# Genome-Wide Identification and Expression Analysis of MYB Transcription Factor Family in Response to Various Abiotic Stresses in Coconut (*Cocos nucifera* L.)

**DOI:** 10.3390/ijms251810048

**Published:** 2024-09-18

**Authors:** Cheng-Cheng Si, Yu-Bin Li, Xue Hai, Ci-Ci Bao, Jin-Yang Zhao, Rafiq Ahmad, Jing Li, Shou-Chuang Wang, Yan Li, Yao-Dong Yang

**Affiliations:** 1 Coconut Research Institute, Chinese Academy of Tropical Agricultural Sciences/Hainan Key Laboratory of Tropical Oil Crops Biology, Wenchang 571300, China; ccsi@hainanu.edu.cn (C.-C.S.); shouchuang.wang@hainanu.edu.cn (S.-C.W.); 2 The Key Laboratory of Plant Resources Conservation and Germplasm Innovation in Mountainous Region (Ministry of Education), Institute of Agro-Bioengineering and College of Life Sciences, Guizhou University, Guiyang 550025, China; 3 School of Tropical Agriculture and Forestry (School of Agricultural and Rural, School of Rural Revitalization), Hainan University, Danzhou 571700, China; 4 School of Breeding and Multiplication (Sanya Institute of Breeding and Multiplication), Hainan University, Sanya 572025, China

**Keywords:** MYB transcription factor, nitrogen deficiency stress, drought stress, salinity stress, gene expression

## Abstract

Abiotic stresses such as nitrogen deficiency, drought, and salinity significantly impact coconut production, yet the molecular mechanisms underlying coconut’s response to these stresses are poorly understood. MYB proteins, a large and diverse family of transcription factors (TF), play crucial roles in plant responses to various abiotic stresses, but their genome-wide characterization and functional roles in coconut have not been comprehensively explored. This study identified 214 *CnMYB* genes (39 1R–MYB, 171 R2R3–MYB, 2 3R–MYB, and 2 4R–MYB) in the coconut genome. Phylogenetic analysis revealed that these genes are unevenly distributed across the 16 chromosomes, with conserved consensus sequences, motifs, and gene structures within the same subgroups. Synteny analysis indicated that segmental duplication primarily drove *CnMYB* evolution in coconut, with low nonsynonymous/synonymous ratios suggesting strong purifying selection. The gene ontology (GO) annotation of protein sequences provided insights into the biological functions of the *CnMYB* gene family. *CnMYB47/70/83/119/186* and *CnMYB2/45/85/158/195* were identified as homologous genes linked to nitrogen deficiency, drought, and salinity stress through BLAST, highlighting the key role of CnMYB genes in abiotic stress tolerance. Quantitative analysis of PCR showed 10 *CnMYB* genes in leaves and petioles and found that the expression of *CnMYB45/47/70/83/85/119/186* was higher in 3-month-old than one-year-old coconut, whereas *CnMYB2/158/195* was higher in one-year-old coconut. Moreover, the expression of *CnMYB70*, *CnMYB2*, and *CnMYB2/158* was high under nitrogen deficiency, drought, and salinity stress, respectively. The predicted secondary and tertiary structures of three key CnMYB proteins involved in abiotic stress revealed distinct inter-proteomic features. The predicted interaction between CnMYB2/158 and Hsp70 supports its role in coconut’s drought and salinity stress responses. These results expand our understanding of the relationships between the evolution and function of *MYB* genes, and provide valuable insights into the *MYB* gene family’s role in abiotic stress in coconut.

## 1. Introduction

Coconut (*Cocos nucifera* L.) is a crucial crop in tropical regions, serving as a significant source of income for farmers and providing diverse products that benefit various industries [1]. Hainan Island is the main coconut producing area in China, where nearly 50% of the annual yield faces numerous challenges, particularly those associated with abiotic stresses such as drought, salinity, and nutrient deficiencies [2]. Hainan Island covers nearly 10% sandy soils, where soil N content is significantly lower than other soils [3]. The lack of N causes older leaves to yellow, reduces the number of female flowers, and decreases the nutritional composition of coconut [4]. Additionally, droughts occur periodically on Hainan Island [5], compounded by seawater intrusion and soil salinization problems [6]. Previous studies showed that drought stress results in withered coconut leaves, dysplasia spathe [7], a decrease in biomass accumulation and seed setting rate, and finally a decrease in both quantity and quality [8]. Likewise, salinity impairs plant growth and development, and decreases the crop yield via water stress [9]. Coconut is tolerant to variable levels of water salinity, but as the mass concentration of sea water increases, the germination rate and germination potential decreases, and growth and development is inhibited [10]. Therefore, understanding the molecular mechanisms underlying stress responses in coconut is essential for developing strategies to enhance its resilience and productivity.

Transcription factors play a crucial role in regulating gene expression by controlling chromatin structure, DNA methylation, dimerization and specific DNA binding. They also affect how plants respond to various stresses and regulate developmental and metabolic processes by either activating or suppressing of target genes [11]. These factors are categorized into different families based on conserved DNA sequences [12]. Among the numerous families of TFs, the MYB (myeloblastosis) family is one of the largest and most functionally diverse groups in the plant kingdom [11]. Based on the number of imperfect tandem repeats in the N-terminus, MYB family members are classified into four subfamilies: 1R-MYB, R2R3-MYB, 3R(R1R2R3)-MYB, and 4R-MYB with one, two, three, and four MYB repeats [12]. Among these, 1R-MYBs are known as MYB-related proteins [13], while R2R3-MYB is the predominant form in higher plants [12]. Each MYB repeat is about 50–53 amino acids long and contains three regularly spaced tryptophan (or aliphatic) residues that form a helix-turn-helix (HTH) structure [13]. During transcription, each repeat’s second and third α-helix connects with the main DNA groove at the particular recognition site C/TAACG/TG [14]. Furthermore, the first MYB protein identified was the C1 protein in maize, which is encoded by the C1 gene [15]. Since then, numerous MYB genes have been discovered in various plant species [15,16,17]. To date, 198, 155, 184, and 179 MYB genes have been reported, respectively, in *Arabidopsis* [18], rice [19], pineapple [20], and coconut [21]. MYB transcription factors is related to abiotic stress responses [22,23,24], such as N deficiency stress [24], drought [25], and salinity [26]. Introducing *OsMYB61* of indica with high N use efficiency into *japonica* with low N use efficiency could increase *japonica* N use efficiency and yield especially under low N condition [27]. Overexpression of *OsMYB6* in leaves did not affect the overall plant growth but reduced wilted leaves under 20% PEG6000 and 150 mM NaCl stress [28], confirming *OsMYB6* is a positive regulator of both drought and salinity stress. Given the established role of MYB transcription factors in enhancing stress tolerance in various plants, OsMYB6 overexpression could be a valuable approach for improving drought and salinity tolerance in coconut.

Although the *MYB* gene family in coconuts was previously identified through genome data analysis [21], a new high-quality reference genome was published by our team in 2021 [29]. This necessitates a more refined genome-wide analysis of the *MYB* gene family in coconuts. Furthermore, the relationship between CnMYBs and stress factors such as N deficiency stress, drought, or salinity remains unclear. To fully understand *CnMYBs*, this study used bioinformatics to identify and analyze the members of the *MYB* gene family in coconut, and performed a comprehensive analysis including phylogenetic tree, gene structure, and motif composition. The findings of this study provide a theoretical basis for further investigation into the function and mechanism of *CnMYB* regulating abiotic stress resistance in coconut, and can be utilized in the genetic improvement.

## 2. Results

### 2.1. CnMYB Genome-Wide Identification and Sequence Analysis

**Figure 1:**
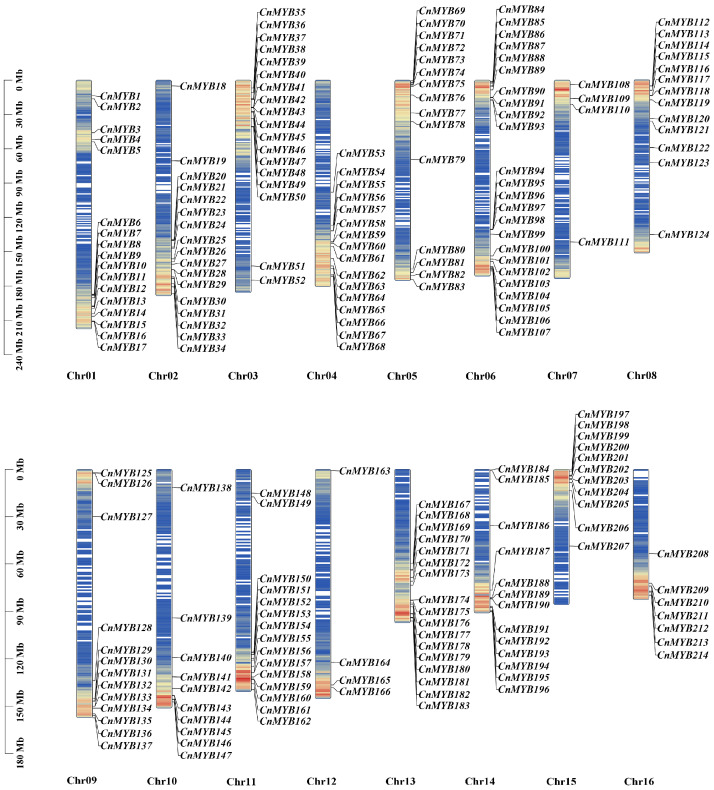
*CnMYB* genes’ chromosomal location. Blue to red on the chromosome indicates gene density.

**Figure 2:**
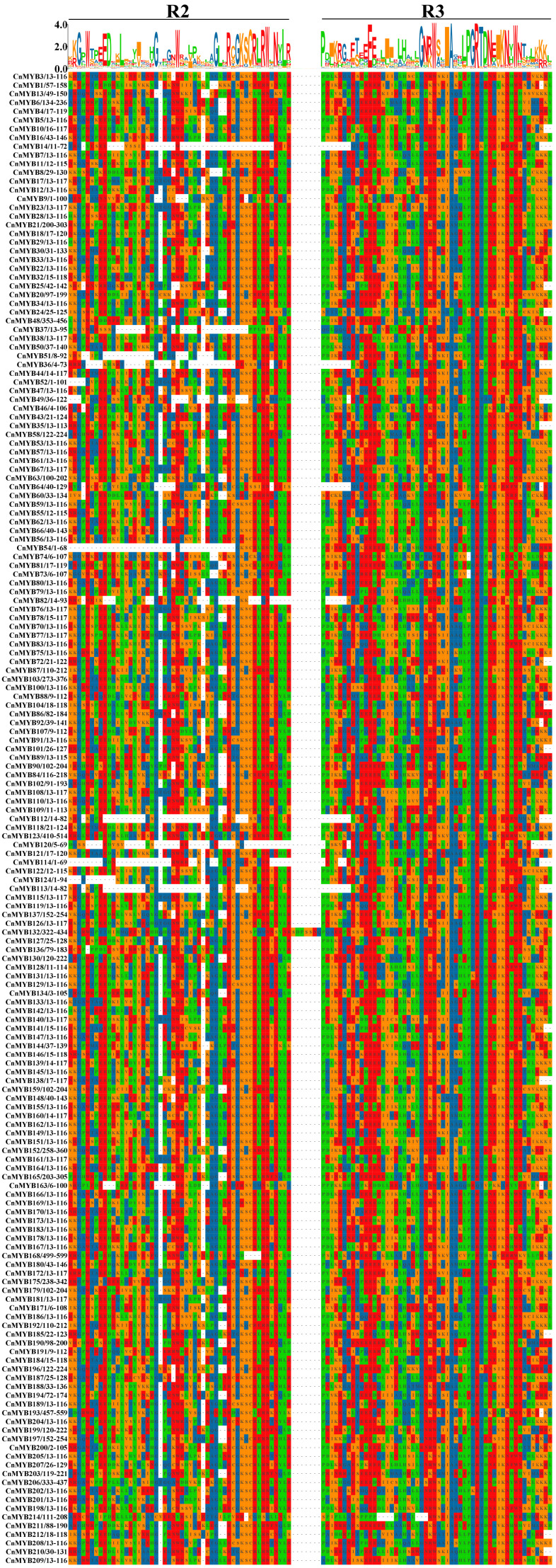
Alignment of the amino acid sequences of R2R3-MYB protein family members in coconut.

From the coconut genome database, 214 MYB genes with the specific domain were identified and named *CnMYB1* to *CnMYB214* based on their chromosomal positions (Figure 1), after removing redundant entries (Figure 1).These *CnMYB* genes are distributed unevenly across 16 chromosomes, with many forming clusters near the ends or tops of the chromosomes. Chromosome 06 contains the highest number of MYB genes, totaling 24, which constitutes 11.21% of the total. In contrast, chromosomes 07 and 12 have the lowest counts, each with only four genes, making up 1.87%. The results of BLAST and sequence multiple comparison showed that there are 39 1R–MYB, 171 R2R3–MYB, 2 3R–MYB, and 2 4R–MYB. R2R3–MYB is the largest group in the coconut *MYB* gene family. In Table S1, the sequences of the *CnMYB* protein ranged from amino acids in *CnMYB120* to 1679 amino acids in *CnMYB213*, with predicted molecular weights spanning from 7.81 kDa for *CnMYB120* to 182.60 kDa for *CnMYB105*. The theoretical isoelectric point (pI) ranged from 4.21 (CnMYB71) to 9.91 (CnMYB117), and there are 127 CnMYB isoelectric points or acidic proteins and the remaining 87 CnMYB are basic proteins. The instability index of *CnMYB* proteins spans from 26.92 in *CnMYB172* to 83.14 in *CnMYB36*. Of the total, only 3 *CnMYB* proteins are stable, while the other 211 are deemed unstable. Additionally, subcellular localization predictions indicated that 192 *CnMYB* members, accounting for 89.72% of the gene family, are located in the nucleus, suggesting that their primary function occurs there. Multiple sequence was performed for the alignment of the domains of 171 R2R3-MYBs to identify conserved aa residues in *CnMYB* genes, and the results showed that there were many highly conserved amino acids in the R2 and R3 domains of CnMYB (Figure 2), especially tryptophan residue (W). However, there were 3 W in R2, but only 2 W residues in R3 domain, because the first W in R3 was usually replaced by leucine (L) or phenylalanine (F) [30]. These conserved aa residues may have great influence on maintaining the HTH structure and function of CnMYBs.

### 2.2. Phylogenetic Relationship Analysis of MYBs in Coconut and Arabidopsis

**Figure 3:**
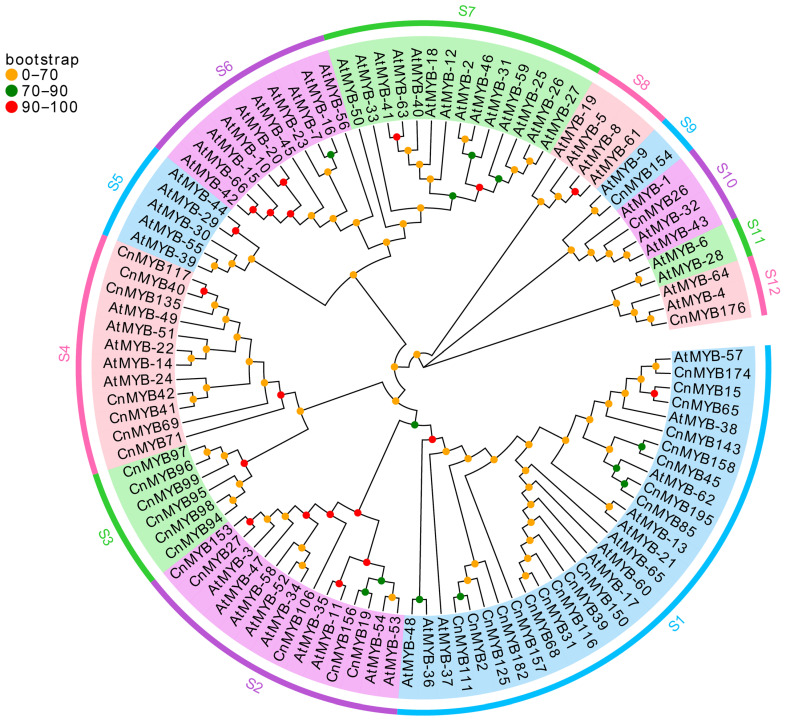
Phylogenetic tree of 1R-MYB protein family members in *Cocos nucifera* and *Arabidopsis thaliana*.

**Figure 4:**
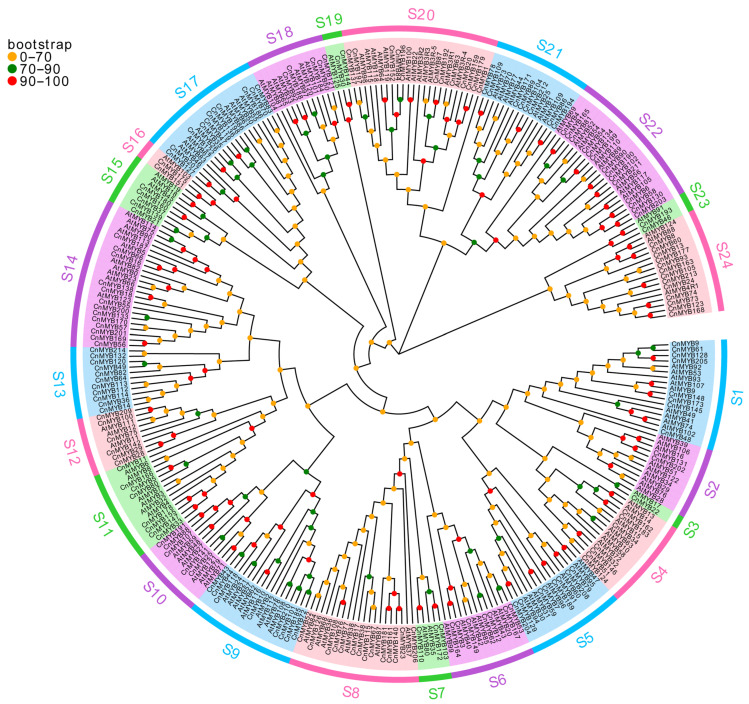
Phylogenetic tree of R2R3-MYB, 3R-MYB, and 4R-MYB protein family members in *Cocos nucifera* and *Arabidopsis thaliana*.

**Figure 5:**
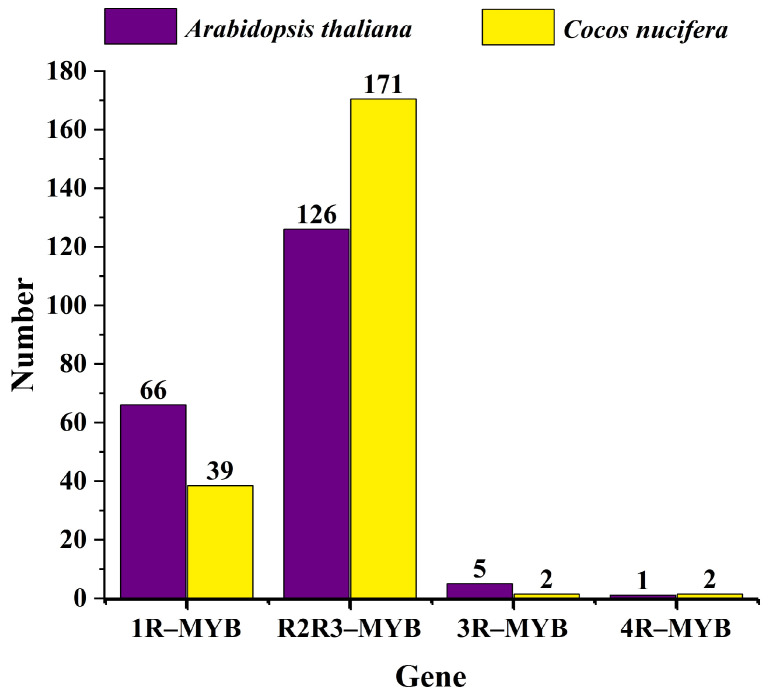
The number distribution of MYB genes in *Cocos nucifera* and *Arabidopsis thaliana*.

To elucidate the evolutionary relationships and potential functions of MYB genes in coconut, we developed phylogenetic trees for 1R-MYB, R2R3-MYB, 3R-MYB, and 4R-MYB genes in both coconut and Arabidopsis (see Figure 3 and Figure 4, Tables S1–S3, Dataset S1 and Dataset S2). The phylogenetic analysis revealed that 1R-MYB genes are categorized into 12 subgroups, with subgroup S1 being the largest, comprising 29 members, and subgroup S9 and S11 being the smallest, each with only 2 members. Notably, some subgroups, such as S3, include only 1R-MYB genes from coconut, indicating a potentially unique function of these genes in coconut. Conversely, subgroups S5, S6, S7, S8, and S11 exclusively contain 1R-MYB genes from Arabidopsis, suggesting that these genes may have different roles or lack similar gene expression functions in coconut. Meanwhile, the phylogenetic trees of R2R3-MYB, 3R-MYB, and 4R-MYB of coconut and Arabidopsis were divided into 24 subgroups, with the smallest subgroup containing only 2 members (S3), the largest subgroup containing 24 members (S20), and the S13 subgroup containing only the MYB members of coconut. These findings indicate the evolutionary divergence of MYB genes across different species over time. Furthermore, a comparison of the number of 1R-MYB, R2R3-MYB, 3R-MYB, and 4R-MYB genes between coconut and Arabidopsis (Figure 5) revealed a similar distribution pattern. In both species, R2R3-MYB constitutes the largest group, while 3R-MYB and 4R-MYB are represented by a smaller number of genes.

### 2.3. Gene Structure and Motif Composition

**Figure 6:**
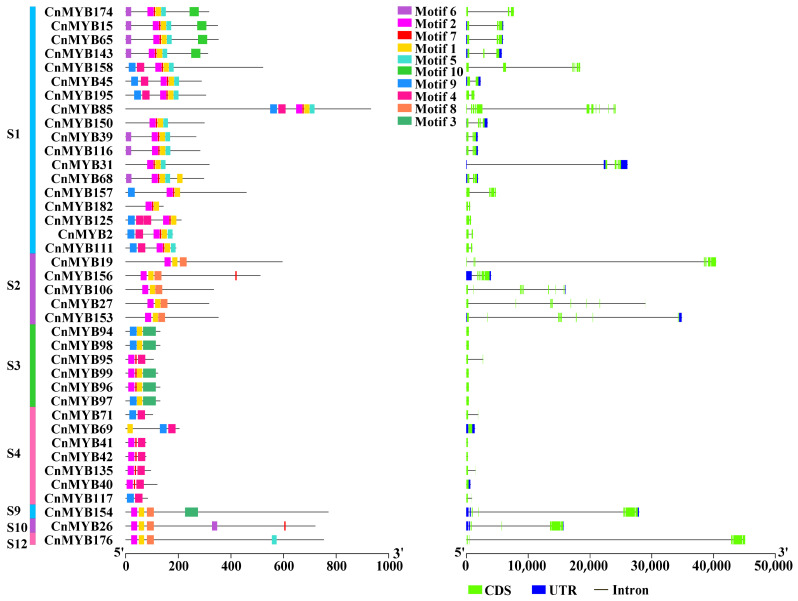
Conserved motifs and gene structure of 1R-MYB family genes in *Cocos nucifera*.

**Figure 7:**
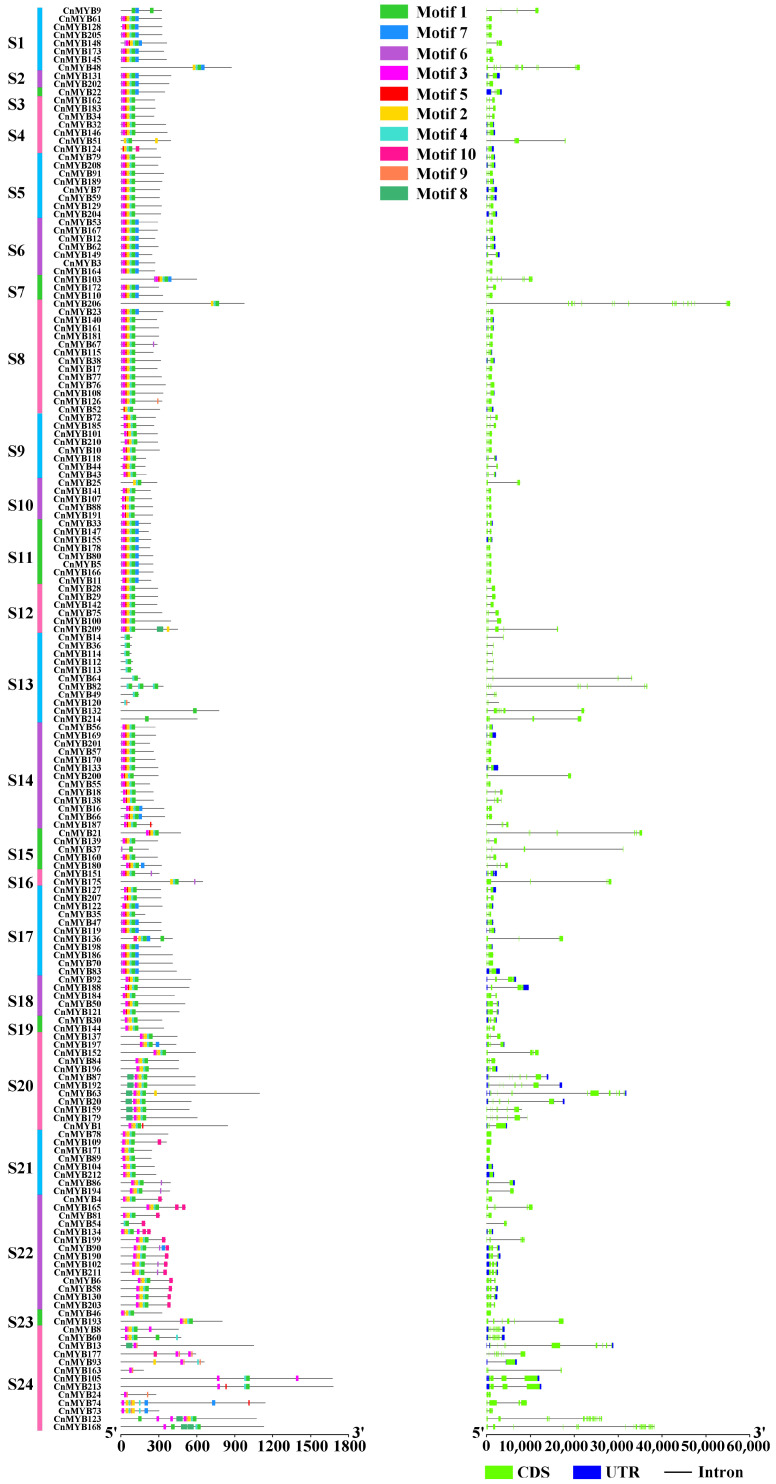
Analysis of conserved motifs and gene structures in the R2R3-MYB, 3R-MYB, and 4R-MYB families of *Cocos nucifera*.

To further explore the characteristics of MYB gene family members in coconut, we conducted an analysis of their conserved motifs and gene structures (Figure 6 and Figure 7). Using the online tool MEME, we identified 10 primary conserved motifs, designated as motifs 1 to 10. The results indicated that genes within the same subgroup often had similar motif compositions, suggesting that CnMYB genes in each subgroup likely have comparable functions. The results of gene structure prediction showed that 199 *CnMYB* contained 1–19 introns, and the remaining 15 *CnMYB* did not contain introns. *CnMYB* genes with two introns had the most, with 107, followed by *CnMYB* genes with one intron, with 27.

### 2.4. Synteny Analysis

**Figure 8:**
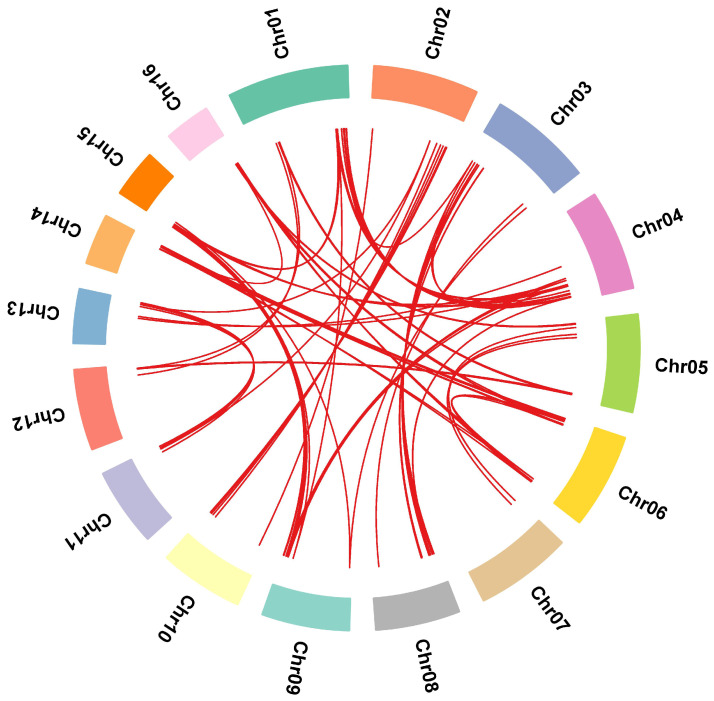
Synteny analysis of MYB gene family in *Cocos nucifera*. The red lines indicate that the repeated *MYB* gene pairs of *Cocos nucifera* are collinear.

**Figure 9:**
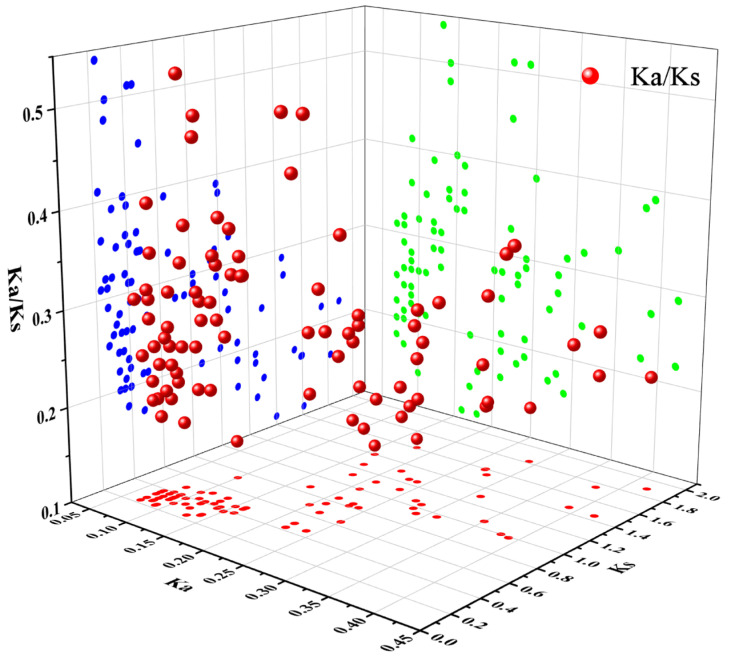
Ka/Ks analysis of 3D scatter plots.

**Figure 10:**
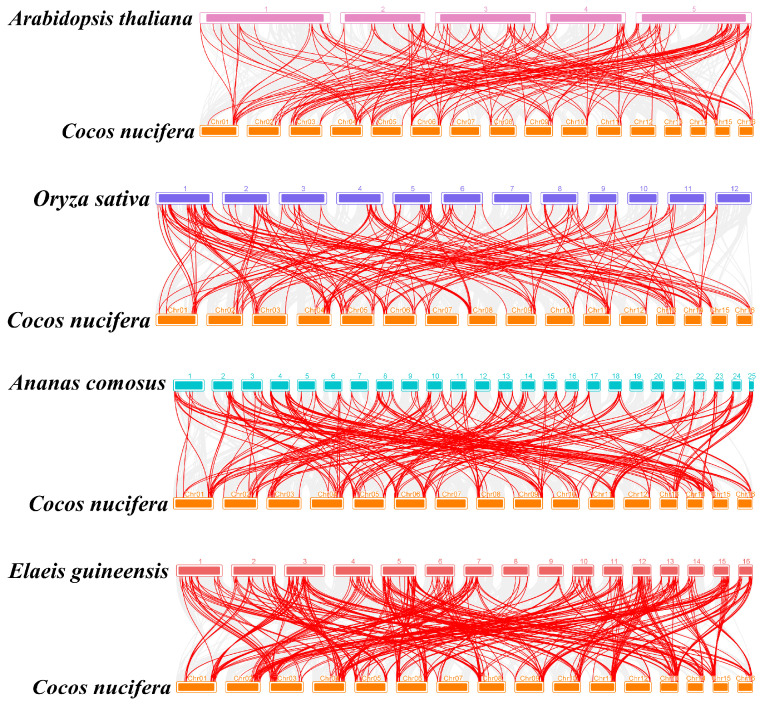
Synteny analysis of MYB gene family in *Cocos nucifera*, *Arabidopsis thaliana*, *Oryza sativa*, *Ananas comosus*, and *Elaeis guineensis*.

Gene duplication events, which significantly contribute to gene family expansion and genomic evolution in plants, were examined in the coconut MYB gene family using TBtools v1.098669 software (Figure 8) [31]. The analysis revealed that all identified genes were segmental duplications, with no tandem duplication events observed. This indicates that segmental duplications have been the primary factor driving the expansion of the *CnMYB* gene family and may play a crucial role in its evolutionary process. The analysis (Table S4 and Figure 9) revealed that the Ka/Ks values of collinear gene pairs in the coconut MYB gene family ranged from 0.11 to 0.54, all below 1, suggesting that these genes have been under strong purifying selection pressure throughout their evolutionary process. The divergent time is between 18.74 and 155.11 Mya. In order to further reveal the evolutionary relationship between the coconut and Arabidopsis, rice, pineapple, and oil palm *MYB* gene families, interspecific collinearity analysis was carried out. The collinearity analysis revealed that coconut and oil palm had a greater number of homologous gene pairs compared to the pairs observed between coconut and Arabidopsis, rice, and pineapple (Figure 10, Tables S5–S8), indicating that coconut shares a closer evolutionary relationship with the *MYB* gene family in oil palm, and these genes may have similar functions.

### 2.5. Gene Ontology Enrichment Analysis

**Figure 11:**
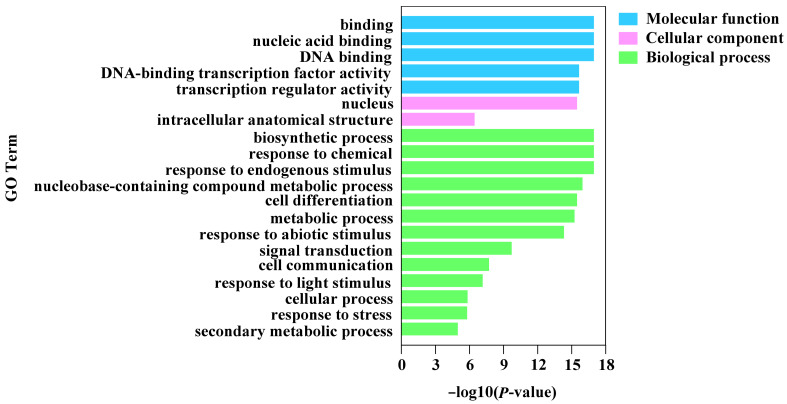
Gene ontology enrichment analyses of 214 *CnMYB* gene family members, with larger −log10 (*p*-value) indicating a more significant pathway.

The GO annotation of the *CnMYB* gene family protein sequences offers insights into their biological roles, which are broadly classified into three categories: molecular function, cellular component, and biological process. The analysis results (Figure 11) revealed that *CnMYB* genes are involved in various molecular functions, including nucleic acid binding, DNA binding, transcription regulator activity, and transcription regulator activity. Cellular component analysis showed that the proteins were mainly enriched in the nucleus, which is consistent with the prediction of the subcellular localization of these proteins (Table S1). In terms of biological process, *CnMYB* genes are mainly concentrated in biosynthetic processes, cell differentiation, metabolic processes, and responses to abiotic stimulus. These results underscore the important role of the CnMYB gene family in regulating plant growth and development.

### 2.6. Key Homologous Genes Analysis

**Figure 12:**
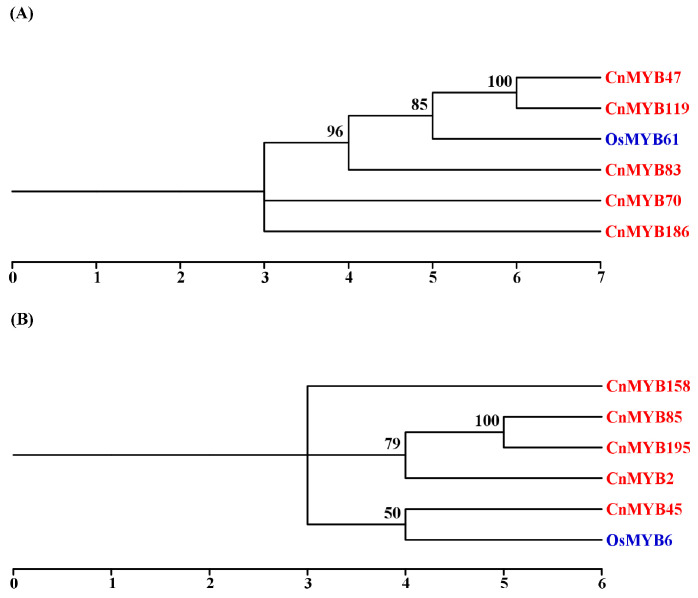
Screening of key homologous genes in coconut genome; (**A**) is the key homologous gene of *OsMYB61* for N deficiency stress, and (**B**) is the key homologous gene of *OsMYB6* for drought and salinity stress. Different colors represent the *MYB* gene of different species. Red represents the *MYB* gene in coconut and blue represents the *MYB* gene in rice.

According to the response of nitrogen (N) deficient stress key gene *OsMYB61* [27], and the key drought and salinity resistance *MYB* gene *OsMYB6* [28] in rice, BLAST was used to screen the possible homologous genes in the coconut genome. A total of 10 homologous genes in coconut were screened, as shown in Figure 12.

### 2.7. Analysis of Cis-Regulatory Elements

**Figure 13:**
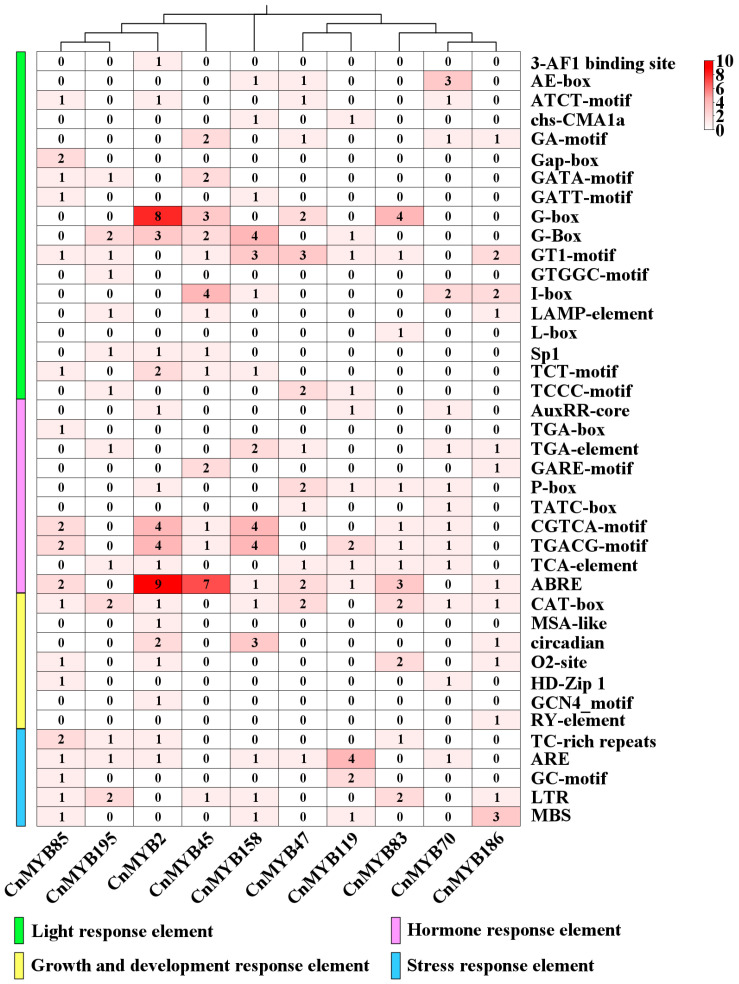
Cis-promoter heatmap of *MYB* genes in *Cocos nucifera*. The columns represent different MYB genes, and the rows represent various cis-regulatory elements identified in their promoter regions. The color gradient indicates the frequency of each cis-regulatory element, with darker red shades representing higher frequencies (up to 10 occurrences) and lighter shades indicating lower frequencies. Green bar: Light response elements. Pink bar: Hormone response elements. Yellow bar: Growth and development response elements. Blue bar: Stress response elements.

To further understand the function of the *CnMYB* family, we first screened the homologous genes of the *CnMYB* family and *OsMYB61*, a key gene of rice nitrogen deficiency stress. We found that *CnMYB47/70/83/119/186* and *OsMYB61* are homologous genes, and they may be the key genes in response to nitrogen deficiency stress. Then, we screened the homologous genes of the *CnMYB* family and *OsMYB6*, a key gene for drought and salinity tolerance in rice, and found that five *CnMYBs* and *OsMYB6* were homologous genes, so *CnMYB2/45/85/158/195* may be the key genes in response to drought and salinity stress. Therefore, we used PlantCare to analyze the 2000 bp sequences upstream of the translation starting sites of 10 selected *CnMYB* homologous key genes to identify cis-acting elements (Figure 13 and Table S9). The analysis revealed various types of cis-acting elements, including those involved in light response. Additionally, elements related to hormone responses were detected, such as those responsive to auxin, gibberellin, jasmonic acid, salicylic acid, and abscisic acid. It is worth noting that *CnMYB2/45* contains multiple abscisic acid response elements (ABRE). This includes the third type of growth and development response elements, except *CnMYB45/119*; all the other genes contained meristem expression elements (CAT-box). Additionally, the analysis identified a cell cycle regulatory element (MSA-like), a seed-specific regulatory element (RY-element), and an endosperm expression element (GCN4_motif) within the *CnMYB* gene. The fourth type of stress response elements includes defense and stress response elements (TC-richrepeats), low temperature response elements (LTR), and drought induced response elements (MBS), among which *CnMYB2/83/85/195* has defense and stress response elements (TC-richrepeats). The multiple functions of these cis-acting elements provide further support for the extensive role of *MYB* genes, indicating that *CnMYB* is closely involved in coconut growth and development, as well as biotic and abiotic stress and other biological processes.

### 2.8. Effects of Abiotic Stress on Growth of Coconut Seedlings

**Figure 14:**
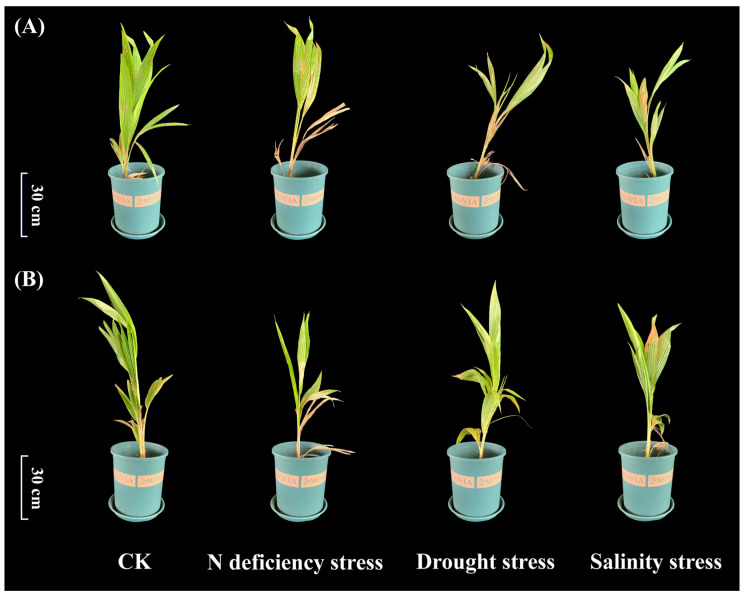
Phenotypic changes in *Cocos nucifera* L. cv. Aromatic Dwarf and *Cocos nucifera* L. cv. *Hainan Tall* under N deficiency, drought, and salinity stress. (**A**) *Cocos nucifera* L. cv. Aromatic Dwarf; (**B**) *Cocos nucifera* L. cv. *Hainan Tall*.

To evaluate the effects of abiotic stress on coconut, *Aromatic Dwarf* and *Hainan Tall* varieties were subjected to N deficiency stress, drought stress, and salinity stress. Compared with the control, these varieties showed reduced plant height with different severity. Under nitrogen deficiency, the upper leaves turned yellow and the lower leaves withered in both varieties. Drought stress caused significant wilting in *Aromatic Dwarf* but minimal wilting in *Hainan Tall*. Salinity stress led to the yellowing and drying of mature leaves in both varieties, with *Hainan Tall* exhibiting greater overall tolerance (Figure 14).

### 2.9. Analysis of Gene Expression

**Figure 15:**
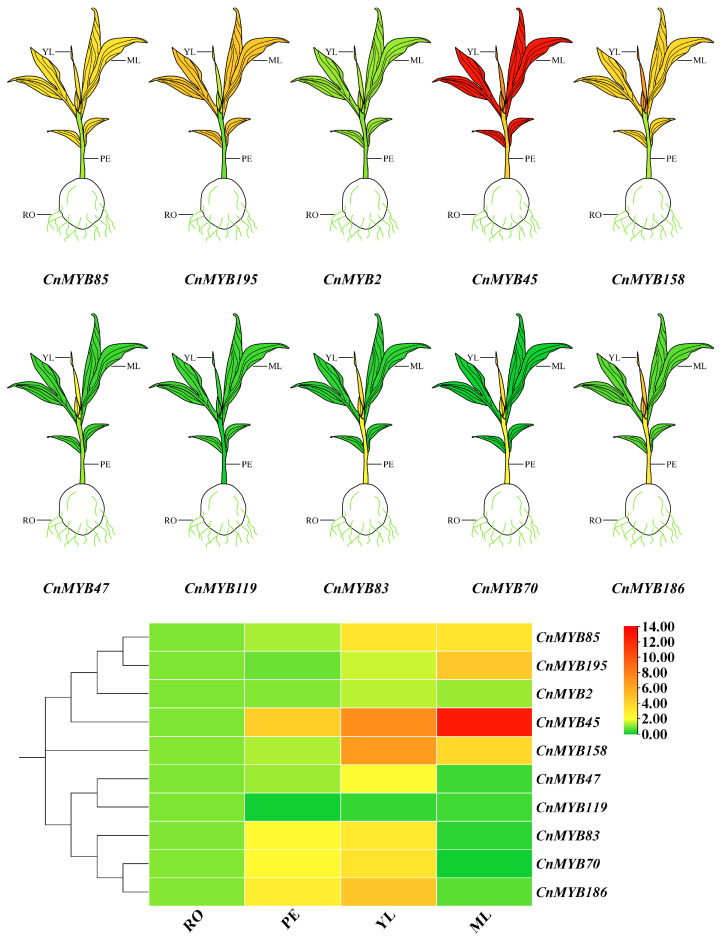
Expression patterns of selected *CnMYBs* across various tissues. RO: roots; PE: petioles; YL: young leaves; ML: mature leaves. The mean expression value was calculated from three independent biological replicates and is expressed in relation to that in the roots. The red represents high expression levels, and the green represents low expression levels.

**Figure 16:**
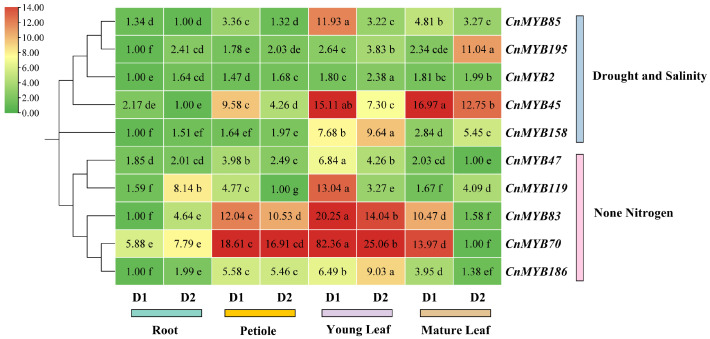
Expression of representative *CnMYBs* in different tissues and growth periods, as determined by qRT-PCR. D1: a dwarf coconut that has been cultivated for three months; D2: a dwarf coconut that has been cultivated for one year. The expression levels are shown relative to the tissue with the lowest expression. Each treatment includes three biological replicates. Red indicates high expression levels, while green indicates low expression levels. There are significant differences in different letter representations in different tissue parts of the same gene, *p* < 0.05.

**Figure 17:**
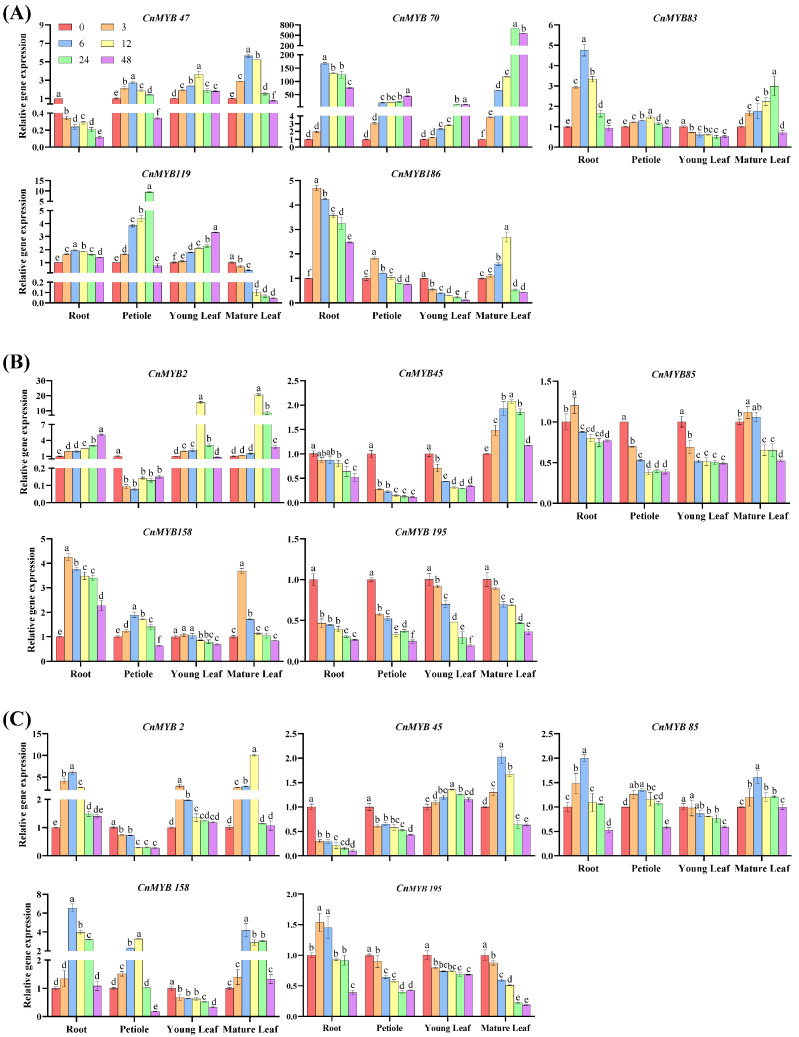
Time-course expression changes in *CnMYBs* under abiotic stress conditions. (**A**): N deficiency stress; (**B**): drought stress; and (**C**): salinity stress. One-year-old coconut plants were subjected to 25% PEG6000, 300 mmol/L NaCl, and Hoagland’s nutrient solution without N, respectively, for 0, 3, 6, 12, 24 and 48 h. The expression levels are shown as values relative to the sample measured at 0 h. There are three biological repeats in each treatment. The values represented by different letters were significantly different at *p* < 0.05.

To investigate the function of 10 *CnMYBs* potentially involved in stress responses during growth and development, we performed qRT-PCR to examine the expression of *MYB* genes in the roots, petioles, young leaves, and mature leaves of one-year-old cultivated dwarf coconut plants (Figure 15). These genes exhibited tissue-specific transcription patterns, with *CnMYB119* showing the highest expression in roots. The other genes displayed higher expression levels in leaves (both young and mature) compared to roots and petioles. Specifically, *CnMYB2*, *CnMYB47*, *CnMYB70*, *CnMYB83*, *CnMYB158*, and *CnMYB186* were predominantly expressed in young leaves, while *CnMYB45*, *CnMYB85*, and *CnMYB195* were highly expressed in mature leaves. It is worth noting that *CnMYB45/70/83/186* is also highly expressed in petioles, indicating that *CnMYBs* are mainly expressed in leaves and petioles. In addition, to understand the 10 *CnMYBs*’ function in coconut development, their expression in four different tissues at two different growth periods were determined by qRT-PCR (Figure 16). All the genes exhibited temporal-specific expression and tissue-specific expression patterns. Among the five genes potentially involved in drought and salinity stress, in all tissues, the expression of *CnMYB45/85* was higher in D1 than D2, while *CnMYB2/158/195* was higher in D2. Meanwhile, across all tissues, the expression of the five genes which might be involved in N deficiency stress were higher in D1 than D2, except *CnMYB186* in young leaf and *CnMYB119* in mature leaf. Furthermore, the expression levels of the 10 CnMYBs (except those related to N stress in D2) were generally higher in the shoots than in the roots. Specifically, *CnMYB2*, *CnMYB47*, *CnMYB85*, *CnMYB119*, and *CnMYB158* were predominantly expressed in young leaves, CnMYB195 showed high expression in mature leaves, and CnMYB45, CnMYB70, CnMYB83, and CnMYB186 were highly expressed in both leaves and petioles. The expression of *CnMYBs* is various in different periods and different tissue parts, indicating that *MYB* may play lots of roles in the growth and development of coconut. The expression level of *CnMYBs* was further studied under nitrogen deficiency, drought, and salinity stress, and the expression of 10 *CnMYBs* in different tissues changed with time. Under nitrogen deficiency stress, the expression levels of most genes increased first and then decreased, while the expression levels of *CnMYB47* in roots, *CnMYB83/186* in young leaves and *CnMYB119* in mature leaves decreased, while the expression levels of *CnMYB70* in petioles and *CnMYB70/119* in young leaves increased. It is clear that all five *CnMYBs* are involved in the response to N deficiency (Figure 17A). Similar changes were observed for *CnMYBs* under drought and salinity stress (Figure 17B,C), indicating that these abiotic stresses either significantly upregulated or downregulated the expression of *CnMYBs*. At the same time, the study showed that under nitrogen deficiency stress, the expression of *CnMYB70* in roots, petioles, young leaves, and mature leaves changed significantly. After six hours of stress exposure, the expression of *CnMYB70* in roots peaked, showing a 167.2-fold increase. After 24 h of stress, the expression level of *CnMYB70* in mature leaves reached the highest level (increased by 692.2 times), and the expression level of *CnMYB70* in petioles and young leaves reached the highest level (increased by 44.6 times and 13.0 times, respectively) after 48 h of stress, suggesting that *CnMYB70* may be the key gene in response to nitrogen deficiency stress. Under drought stress, the expression of *CnMYB2* in petioles, young leaves, and mature leaves changed significantly. After 6 h of stress, the expression of *CnMYB2* in petioles was the lowest (decreased by 12.5 times), and after 12 h of stress, the expression of *CnMYB2* in young and mature leaves reached the highest level (increased by 15.6 times and 20.6 times, respectively). *CnMYB2* is likely a key gene in response to drought, while both *CnMYB2* and *CnMYB158* show significant expression changes in roots and mature leaves under salinity stress. After 6 h of stress, the expression of *CnMYB2/158* in roots reached the highest level (increased by 6.1 times and 6.6 times, respectively), and the expression of *CnMYB158* in mature leaves reached the highest level (increased by 4.2 times). After 12 h of stress, the expression of *CnMYB158* in mature leaves reached the highest level. The expression of *CnMYB2* in mature leaves reached the highest level (increased by 10.1 times). Therefore, these two genes may be key genes in response to salinity stress.

### 2.10. Protein Structure Analysis

**Figure 18:**
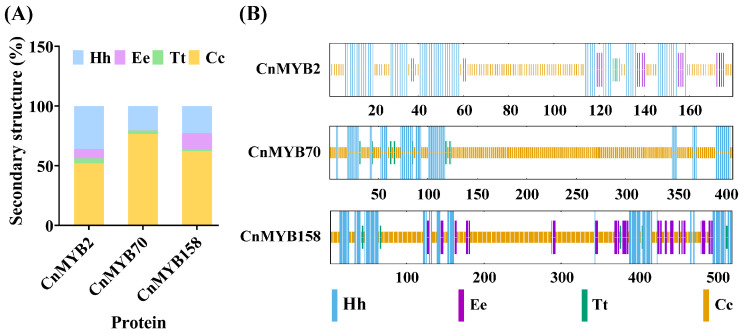
Secondary structure properties of MYB protein in *Cocos nucifera*. Hh: Alpha helix; Ee: extended strand; Tt: Beta turn; Cc: random coil. (**A**) Proportion of secondary structure element. (**B**) Distribution of secondary structure element.

**Figure 19:**
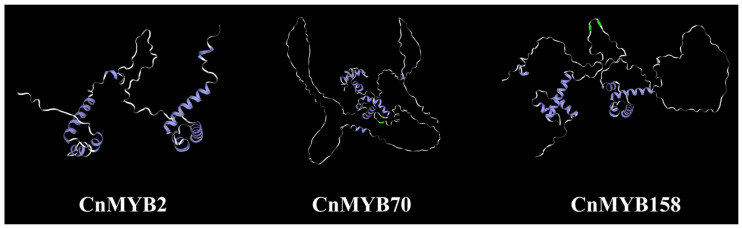
The predicted tertiary structure of MYB proteins in *Cocos nucifera*. Purple represents the Alpha helix, green represents the β-sheet, and gray represents the random coil.

To further explore the function of three key genes of CnMYB2/70/158, we used SWISS-MODEL and SOPMA for the prediction of their secondary and tertiary structures (Figure 18 and Figure 19, Table S10). The secondary structure analysis revealed that the three CnMYB proteins were dominated by random coil and alpha helices, with random coils being the most prevalent, ranging from 51.96% to 76.47%. Additionally, the secondary structures include small amounts of extended strand and beta turn. Notably, there are clear differences in the tertiary structures of these proteins, suggesting they have distinct functions, further illustrating the functional diversity of MYB gene family members.

### 2.11. Protein Interaction Network Prediction

**Figure 20:**
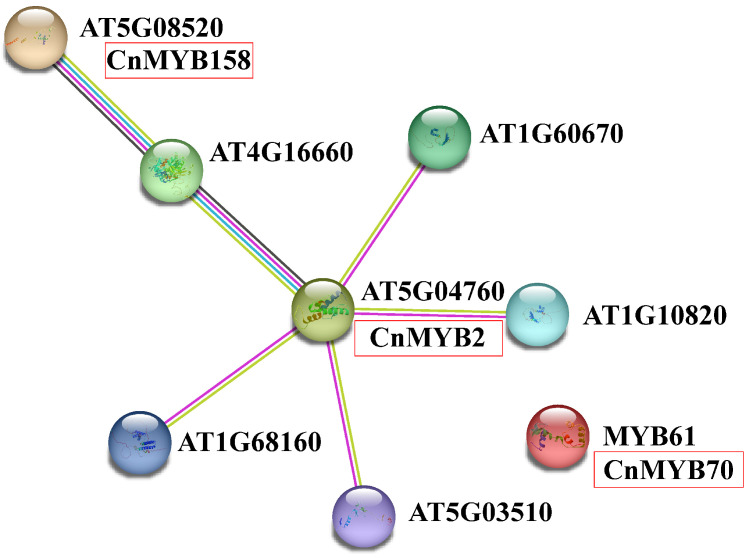
Protein interaction network of MYB in *Cocos nucifera* constructed by referring to AtNLP. The different data sources of predicted interactions are indicated by lines with diverse colors. Blue represents curated databases. Purple represents experimentally determined. Chartreuse represents text mining. Black represents co-expression. Coconut homologous protein names are highlighted in red frame.

To further investigate the protein interactions and potential functions of the three key genes, we constructed a *CnMYB* protein interaction network using the STRING website, with a focus on Arabidopsis (Figure 20). Among them, CnMYB2 corresponds to AT5G04760, CnMYB70 to MYB61, and CnMYB158 to AT5G08520. From the network diagram, it can be seen that CnMYB2 interacts with various proteins such as AT5G03510, AT1G68160, AT4G16660, etc. CnMYB158 interacts with AT4G16660 proteins, while no interacting proteins were found for CnMYB70. It has been reported that AT4G16660 (Hsp70) enhances plant tolerance to various abiotic stresses [32,33], suggesting that CnMYB2 and CnMYB158 may have similar functions and play a crucial role in coconut stress resistance.

## 3. Discussion

### 3.1. MYB Transcription Factor in Coconut

The *MYB* gene family, known for its role in responses to abiotic stress, is among the largest transcription factor families in the plant kingdom [11,23]. In the present study, 214 *MYB* gene family members were identified from the coconut genome, which is greater than the number identified in coconut (179) by Li et al. [21]. This different result is mainly due to genomic differences in accordance with Li et al. [21]. Meanwhile, the total count of *CnMYB* genes exceeded those identified in *Arabidopsis* (198) [18], rice (155) [19], and pineapple (184) [20], yet it was lower than the counts in potato (253) [12] and pepper (235) [34]. This suggests that the *MYB* gene family has undergone patterns of evolutionary expansion across various plant species. The loss or gain of introns is significant for gene amplification [35]. An analysis of the *CnMYB* gene structure indicated that 92.99% of the coding sequences were interrupted by introns. However, 15 genes had no introns and showed a result consistent with pear plant [36], suggesting that the loss of introns may have occurred during evolutionary history of coconut. Furthermore, the sequence analysis revealed that the *R2R3-MYB* gene family members in coconut retain highly conserved R2 and R3 domains, consistent with findings in *Hypericum perforatum* [37]. Furthermore, most *CnMYBs* within the same subgroup exhibited a similar gene structures and motif compositions, indicating a high degree of conservation among *MYB* genes, and the result was similar with previous results concerning pepper [34].

Phylogenetic analysis found that not all CnMYBs and AtMYBs were clustered in the same subfamilies, indicating these genes might have been lost in *Arabidopsis* or coconut gained them from the last common ancestor, and this result was consistent with results obtained in cotton [38]. Previous studies have reported that the gene replication events experienced by a genome in the evolutionary process are the key reasons for the amplification of a gene family and the establishment of new functions [39]. In this study, the main driver of *CnMYBs*’ evolution in coconut was segmental duplication, which is critical for the rapid expansion and evolution of gene families, as well as their environmental adaptability [30,39]. Moreover, many studies showed that the MYB protein can specifically bind to the promoter of related target genes, thus changing their gene expression, leading to the regulation of low nitrogen [24,27], drought [38,40], and salinity [41,42,43] stress tolerance in plants. The gene ontology enrichment analysis of our results showed the involvement of *CnMYB* genes in abiotic stress. In the present research, the prediction of cis-acting regulatory elements revealed that *CnMYB* genes possess numerous elements associated with stress responses, highlighting their crucial roles in managing abiotic stress. However, the *CnMYB* genes related to abiotic stress have been rarely verified, necessitating further exploration.

### 3.2. Analysis of the Expression Pattern and Abiotic Stress Resistance Function of CnMYBs

Coconut is an important source of income for farmers in tropical areas, and has beneficial uses in various aspects of life [1]. Improving abiotic stress resistance is a significant target for coconut breeding. Previous studies related to *MYBs* have shown their positive effects in response to N deficiency [24,44], drought, and salinity stress [45,46]. BLAST is widely used to identify homologous sequences [47]; thus, the *CnMYBs* related to low N (*OsMYB61*) [27], drought, and salinity stress (*OsMYB6*) [28] were screened by BLAST in this study. The results of the BLAST analysis of *CnMYB47/70/83/119/186* and *OsMYB61*, and *CnMYB2/45/85/158/195* and *OsMYB6* were homologous. Tissue expression analysis showed that *CnMYBs* are mainly expressed in leaves and petioles, whereas *OsMYB61* and *OsMYB6* are mainly expressed in leaves [27,28]. The main reason for the difference may be different species. Furthermore, the result of qRT-PCR confirmed that the expression of *CnMYBs* was differentially upregulated under N deficiency, drought, and salinity stress, and the results were consistent with previous studies in plants [48,49]. At the same time, this study showed that the expression of *CnMYB70* in roots, petioles, young leaves, and mature leaves changed significantly under nitrogen deficiency stress, and *CnMYB70* may be a key gene in response to nitrogen deficiency stress. Under drought stress, the expression of *CnMYB2* in petioles, young leaves, and mature leaves changed significantly, and *CnMYB2* may be the key gene in response to drought stress. The expression of *CnMYB2/158* in roots and mature leaves was significantly changed under salinity stress, and these two genes may be the keys in response to salinity stress. In this study, it was found that CnMYB2, CnMYB70, and CnMYB158 had different structures among proteomes. CnMYB158 interacted with Hsp70 protein, while no interacting protein was found in CnMYB70. CnMYB2 interacted with Hsp70 and other proteins, and CnMYB2 was located at the key node of the protein network, suggesting its more important function. In summary, this study identified key genes associated with responses to nitrogen deficiency stress, drought stress, and salinity stress, but their molecular mechanisms still need to be confirmed.

## 4. Materials and Methods

### 4.1. Identification and Analysis of Coconut MYB Family Genes

To obtain the coconut *MYB* gene sequences, one can download the whole dwarf coconut genome data and gene structure annotation file uploaded by Wang et al. from the National Genome Data Center (https://ngdc.cncb.ac.cn/gwh (accessed on 21 March 2023)) [29]. A Hidden Markov Model (HMM) profile representing the MYB DNA-binding domain (accession number PF00249) was acquired from the Pfam protein family database (http://pfam.xfam.org/ (accessed on 7 May 2023)). This model was used to identify MYB proteins using a HMMER search program which is provided by TBtools v1.098669 [50] with an E-value cut of 1 × 10^−5^. AtMYB amino acid sequences were obtained from the PlantTFDB database (http://plantregmap.gao-lab.org/ (accessed on 28 May 2023)) to identify *CnMYB* gene families by BLAST searches. Additional information regarding the number of amino acids, molecular weight (MW), and isoelectric point (pI) of CnMYB proteins of interest was gathered using the ExPASy proteomic website (https://web.expasy.org/compute_pi/ (accessed on 1 June 2023)). The WoLF PSORT Prediction (https://wolfpsort.hgc.jp/ (accessed on 1 June 2023)) was used to predict the *CnMYB* genes intracellular distribution. Multiple sequence alignment of coconut R2R3–MYB was carried out with ClustalW, the R2R3 sequence characteristics conservative domain was analyzed, and the sequence identification was obtained by using the default setting of TBtools v1.098669 [50].

### 4.2. Phylogenetic Analysis

The amino acid sequences of AtMYB and CnMYB proteins were compared using MEGA 11 software (Arizona State University, Tempe, AZ, USA). A phylogenetic tree was constructed with the Neighbor Joining (NJ) method and 1000 bootstrap replications, utilizing MEGA 11 software, and subsequently visualized with the EvolView online tool (http://www.evolgenius.info/evolview, accessed on 12 June 2023).

### 4.3. Analysis of Conserved Motifs, Gene Structures, and Chromosomal Distribution

A search for conserved motifs in CnMYB proteins was conducted using the Multi Em for Motif Elicitation (MEME) tool (http://meme-suite.org/tools/meme, accessed on 13 June 2023), with the default settings and a maximum of 10 motifs. Information on exon/intron structures, including mRNA, coding sequences (CDS), and untranslated regions (UTRs), as well as the chromosomal distribution of CnMYB genes, was extracted from the coconut genome in a general feature format file using a Biolinux system. Visualization of these data was performed using TBtools v1.098669 [50].

### 4.4. Ka/Ks Analyses and Gene Collinearity

Genomic data of *Ananas comosus* and *Elaeis guineensis* were downloaded from JGI–Phytozome database and National Center for Biotechnology Information database (https://phytozome-next.jgi.doe.gov/, https://www.ncbi.nlm.nih.gov/ (accessed on 24 June 2023)). The rates of synonymous (Ks) and nonsynonymous (Ka) nucleotide substitutions in duplicated genes were evaluated using TBtools v1.098669 [30], and the Ka/Ks ratio was subsequently computed [50].

T (divergence time) = Ks/2r (neutral substitution rate, r = 2.6 × 10^−9^) (Mya) (1)

The gene duplication events in coconut MYB and the collinearity analyses with four other species were investigated using MCScanX 1.0, while the Multiple Synteny Plotter in TBtools v1.098669 [50] was employed for the collinearity analysis.

### 4.5. Gene Ontology Enrichment and Cis-Regulatory Elements

Gene ontology data for the CnMYB gene family members were acquired through the eggNOG-mapper website (http://eggnog-mapper.embl.de/, accessed on 29 July 2024). Promoter sequences were extracted from the dwarf coconut genome database [21]. Cis-regulatory elements were analyzed using PlantCARE (http://bioinformatics.psb.ugent.be/webtools/plantcare/html/, accessed on 27 June 2023) and the results were visualized with TBtools v1.098669 [50].

### 4.6. Plant Growth and Treatments

Experiments were conducted using dwarf coconut (*Cocos nucifera* L. cv. *Aromatic Dwarf Lam.*, *Cn. dwarf*) as the experimental material, specifically at 3 months and 1 year after the seed fruit had sprouted. All experiments were performed at the Sanya Nanfan Research Institute of Hainan University (18°30′ N, 109°60′ E). The coconut seedlings with a similar size, height, and number of leaves were transferred to a nutrition bag (28 cm × 28 cm × 25 cm). Substrate cultivation (Shandong Duoka Agricultural Technology Co., Ltd., Weifang, China) was used. The N deficiency stress treatments were treated by Hoagland’s nutrient solution without N, the drought stress treatments were treated by 25% PEG6000 with Hoagland’s nutrient solution, and the salinity stress treatments were treated by 300 mmol/L NaCl with Hoagland’s nutrient solution. Three biological replicates were taken at each sampling point.

The young leaves, mature leaves, petioles, and roots of coconut plants cultivated for 3 months after germination and 1 year after fruit had first sprouted were sampled. In addition, the phenotypes of abiotic stressed plants were observed. Then, samples of young leaves, mature leaves, petioles, and roots of *Cn. dwarf* coconut seedlings cultivated for 1 year after the seed fruit had sprouted were collected at 0 h, 3 h, 6 h, 12 h, 24 h, and 48 h under nitrogen deficiency, drought, and salinity stress, respectively. All samples were immediately frozen in liquid N and stored at −80 °C for further quantitative real-time PCR analysis.

### 4.7. Quantitative Real-Time PCR Analysis

The total RNA was isolated using an RNA extraction kit (Tiangen, Beijing, China, DP441). A quantity of one microgram of RNA was converted into cDNA using HiScript II Q RT SuperMix for qRT-PCR (Vazyme, Nanjing, China, R223). qRT-PCR was used to explore key *MYB* genes expression in coconut. Gene-specific qRT-PCR primers were designed by Primer Primer5 software (Primer Biosoft, Palo Alto, CA, USA) (Table 1). Coconut housekeeper gene β–actin was used as an internal reference gene. The PCR reactions were carried out using the ananlytikjena qTower 2.0 (analytikjena, Jena, Germany). The procedure started with an initial step of 30 s at 95 °C, followed by 40 cycles of 15 s at 95 °C and 30 s at 60 °C. Relative expression levels were assessed using the 2^−∆∆CT^ method, and the experiment included three independent biological replicates.

### 4.8. Protein Secondary Structure Prediction and Tertiary Structure Construction

The secondary structure of MYB proteins was predicted using the SOPMA website (https://npsa.lyon.inserm.fr/cgi-bin/npsa_automat.pl?page=/NPSA/npsa_sopma.html (accessed on 29 July 2024)), and the tertiary structure of MYB proteins was predicted via the SWISS-MODEL online website (https://swissmodel.expasy.org/interactive (accessed on 29 July 2024)).

### 4.9. Construction of the Protein Interaction Network

Through the functional protein association network online website STRING (https://stringdb.org (accessed on 29 July 2024)), we predicted the interaction of MYB family members with related proteins; we selected the model species Arabidopsis as the species source.

### 4.10. Statistical Analysis

The data were analyzed using Microsoft Excel 2003 (Microsoft, Redmond, WA, USA) to determine means, and ANOVA was performed with Statistix 9 (Analytical Software, Tallahassee, FL, USA). Graphs were created and visualized using GraphPad Prism 8.0.2.263 (GraphPad Software Inc., San Diego, CA, USA), Origin 2024 (OriginLab Corporation, Northampton, MA, USA), MedPeer (http://www.medpeer.cn, accessed on 22 July 2024), and TBtools v1.098669 [50].

## 5. Conclusions

This research identified 214 *MYB* genes in coconut and comprehensively analyzed their sequence characteristics, motifs, gene structures, phylogeny, synteny, and cis-acting regulatory elements. The CnMYBs within the same subgroup displayed highly conserved intron structures and motif compositions. Synteny analysis and Ka/Ks ratios indicated that these genes have experienced segmental duplication events and have been subject to purifying selection throughout evolution. The prediction of cis-acting regulatory elements suggests that *CnMYBs* may play crucial roles in response to abiotic stress. Notably, *CnMYB70*, *CnMYB2*, and *CnMYB158*, which are associated with nitrogen deficiency, drought, and salinity stress in coconut, were identified through bioinformatics analysis and qRT-PCR. These findings advance our knowledge of the MYB gene family in coconut and aid in breeding stress-resistant varieties.

## Figures and Tables

**Table 1:** The qRT-PCR primer sequences.

Gene Name	Forward Primer Sequence (5′–3′)	Reverse Primer Sequence (5′–3′)
β–actin	ATAAAGTATGGCTGATGCTGAGG	CAACAATGCTTGGGAACACA
CnMYB2	AAGGGCTGGCTAAGTATGG	GTATTCTGGTTCTGACGGATG
CnMYB45	AAGACGGCGGCTCAGGTAAG	ATGGTGGTGGTCCTTCTCCTTG
CnMYB47	TTCTGATAATAAGGTAGGCTC	GCAGGCTTCCGTTAGGTC
CnMYB70	CAGTCCTTGGCAACAGATGGTC	GGGTCTCGGAAAGTGGTTTGTG
CnMYB83	TGCTGCTCTCGATCCTTCTTCC	CCTGGCTGGCGACGAACC
CnMYB85	TTACAGGGACTTGGAGGACGAC	GACGCAGTAGGATGGCTTGAAC
CnMYB119	TGCCAGGGAGAACAGACAAT	GGCTTCACTGGCTGATGCTCT
CnMYB158	ATCCCTGACGCTCACTTCTCC	TGGTTCGGCTTGTTCGCATTC
CnMYB186	GTCTTGGGCAACAGATGGTCTC	TTGGGTCTCAGAAAGCGGTTTG
CnMYB195	CCAGGACACCTACCCAAGT	AGAACTGGTCAGAGATTGTGGT

## Data Availability

Data will be available upon request.

## Supplementary Materials

The following supporting information can be downloaded at: https://www.mdpi.com/article/10.3390/ijms251810048/s1.
