# Supplementary material for: Genome-Wide Identification and Expression Analysis of MYB Transcription Factor Family in Response to Various Abiotic Stresses in Coconut (Cocos nucifera L.)
Source: Int J Mol Sci. 2024 Sep 18;25(18):10048. doi: 10.3390/ijms251810048 (PMC11432468; doi:10.3390/ijms251810048)
Supplement: Supplementary file 1 [file ijms-25-10048-s001.zip › ijms-3189138-supplementary datesets S1 and S2.pdf]

Dataset S1. Fasta files of MYB proteins in *Cocos nucifera*.

>CnMYB1
MTNDILLSSFLEHIMDSRVNPNLTQAFIAQNNLITGQANEQSPMLEDGANTIIPYKPKRP
NLVTIWTPEDDRLLIKLVKKYGEKNWIIIADKLKKKTSRQCRERWINYLRPNIKRDAWTD
EEERITILAHQELGNKWVEIAKRLPGRTENALKNHWNSTKRKLYSGRPWWSTLAKKTPL
QEYIKCLEDPSSDIASVVAALTGCNNCSMASVNHITMPAVGPRGCNDNHMSFFNHGTQST
LWQNYNKSSSQSSVMAAAMVATPISTWNGGCNSGSMVPLPSSYLPNSTGSLTQPSAVAGSI
MAAPNGNWRSSFNYVSGAPMNLTSIPAIELGKGKDSHVDLFSSDLQSSCLRNYDKSLTQS
SVMATSAMAALTKNLDTGSNSGLVIPNTLTSFDLPLVAATPMGDQESGWNINNVVNDLDS
ISSFDFSRMSDLWSTKVLPNRNEDIDGTSTESIKQQVQAWTSVFVKEPIGNQESGWNSSS
MAPTTATRMPHIGLDMGEDDVMDLFTYSSPRSLLQNNLRNLDQPVALLAPNGSQEIVCNG
SSMATTTLMSVPATEHNFDEGHSKSITTLELDNNPNFMFAEMLADWKEDISFANTEPIRQ
QVQASASSSVMRAPAGDHESGRNIGWMAPMALASMPTSEFNVGENDGTNLFPSEDISCT
NIQQPRKDPWAKKGCGVVVESNTIDWGLDDLPDCMFMEVSPDRDDDIVNTCTLTEKGSG
VEMEMLLQGEDEINCTNIEQSVGDPSTNKGCAMEMVGSSNAANWALDDLPHMVTEVLP
HRENSSWTNIDQPRGDVSKEKGSRMEMVGSNTAEKMACDENEKRNLNLRDMDLLEMIS
FNSFNTRGSMI*
>CnMYB2
MEFFRGWTRVEDKAFERALVAIPEEAPDRWSLIAAQVPGRSPREVWERYQLLVRDLEMIE
RGEVETPGKWDDDDDDDDAGESTDAAGRSDSRGHQISFGRGRGEERRRGIPWTEEEHRL
FLEGLAKYGRGDWRNISRWAVKTRTPTQVASHAQKYFIRQNQNTSNRESKRKSIHDITTP*
>CnMYB3
MGRQPCCDKIGLKRGPWTMEEDQKLINFIMNNGIHCWRLVPKLAGLMRCGKSCRLRWT
NYLRPDLKRGAFSEDEENQIIQLHSCLGNRWSKIASYFPGRTDNEIKNHWNTRVKKRLKL
LGLDPVTHKPIEQPAKYDSSRSDRVSESNTSEVQKKSMEIMDIVSSSQEEHLMNDEKQKG
QEEHLMNDEKQKEVQFGSNDTIVLHRNGMSWESLDVEEMKPRANPSSSFSTSFSIDDISPE
CSNLCWFDTIDSFPSWEALYPLEDVFPFGNFP*
>CnMYB4
MGSSSAATGSDDAKACPRGHWRPGEDEKLRQLVEQYGPQNWNSISEKLQGRSGKSCRLR
WFNQLNPRINKRPFTEEEEERLLAAHRIHGNKWALIARLFPGRTDNAVKNHWHVIMARRY
RERSRLFRKSSGRDQILRSSDVSTANGLFTSCSSAPQEGSSSKHIQFDHGCRLFEFGSSTT
DRFPAASPSSLSWAFSGSTISSSPQIFGAKRRDFFYRSKYNYNVLGRSHFHDQSLRSSYH
PYRSFNALCDRDYTRVVPQSIRFSTTSDGCGSEVMSAGEIIRLGDSSARGMNHRADHNQQ
DDGDESLKRKDVPFIDFLGVGISSSLNS*
>CnMYB5
MGRSPCCEKAHTNKGAWTKEEDQRLIAYIKAHGEGCWRSLPKSAGLLRCGKSCRLRWIN
YLRPDLKRGNFTEEEDELIIKLHALLGNKWSLIAGRLPGRTDNEIKNYWNTHIKRKLLSQG
LDPQTHRPLHGGAPFATPRQQEAPTAIAQPVNSADEGQSCSTNLDEDRFPDLNLDLSISL
PYYSPKSSPPSEGSATAAAAATTSRSSHTQPICFCCHLGFQSNEACSCQTIPNSHVYRYI
RSLEEGQHINYSS*
>CnMYB6
MALKQCNGRQGLRSFPPSSSSSSSSSSSSSSKGLFAGMSSLSINPASSVESYCSTKGSEKN
SRTSSFMRDYIGLEDHPPRNNEDGGVKWSDASDGFGENHVNATGEESPEGENTNEGSLG
GRETAADCGQPKLCARGHWRPAEDSKLRELVALHGPQNWNLIAENLEGRSGKSCRLRWF

NQLDPRINRSAFTEEEEEKLMAAHRLYGNKWAMIARLFPGRTDNAVKNHWHVIMARKYR
EQPNAYRRRKLSQAVPMRLEDTTATSVAYGFNTNLHLPFTHPPLAMCGSNGSHHITTSEGG
EDSVSCYSGFSSDQAPYDFLSGIKAQDKARSFHSTSSWCWQRPRDESFIGSYPQHPIMVAM
QQSRHPSYLSDCSTASGVSAAESSSFKENGENGHFEATASPPFIDFLGVGAT*
>CnMYB7
MGRPPCCDKVGVKKGPWTPEEDIILVSYIQEHGPGNWRAVPTNTGLMRCSKSCRLRWTN
YLRPGIKRGNFTDQEEKLIIHLQALLGNRWAAIASYLPERTDNDIKNYWNTHLKKKLKKL
ETGAEGQTSSGVSSHKSISKGQWERRLQTDIKTAKQALCEALSVEKLNSLSNYSKASSNC
YNWSTTPSTYASSTENISRLLEGWTRNSTRPGACQANSGSIKTETASSQGTPSAADCIPSP
EPLESLFGLNSSSTEASETSLLGEESKPVLEAHEPFSFFESWLLDESVGHGQLSLLDMAL
DDAPEILF*
>CnMYB8
MERRSEMAAAAGMNSDGSVAPAAVAAPPKKDRHIVSWTPKEDDLLREQVALHGTENWT
RIAAQFKDKTGRQCRRRWNTYLNAECKKGGWSAEEDRLLCEAQKVYGNRWTEIAKVVS
GRTDNAVKNRFSTLCKKRAKHEALSKENNGSCLNPNNKRVIIRNGCIPAETRELSLPTKHM
RYHISDLKENKRNEEYLGKHGMNKDQLRSPLAVLAQNCNIADNRTRGTFLKKDDPKLTA
LLQQAELLSSLAQKVNAENTKESLENAWKEIQDYMAQTEESGMPSRKISGTDFVLDDFRD
LIEELMSSDSIGQQSLRQSDLHENSRSSEYSAGSSGHFNNQGNEKDHQTDDCVFSNNTEIS
GPQEDAQCFGMTACQEEVMPPLTKPKENDENSCNISNSEFASPLLTTPPFQSLADGILTPKF
TASERRFLQSVIGLPSPVPSPKSSQPPACKKALLDSL*
>CnMYB9
MENPIAMNNVVEVADYILRTQICLGQTSKVAMIRCRVRSDAEGGRGDIRDIMFTSPSVEG
GPPDKELKPEKQPTEMSKFLAWSKWLLARWSAIATHLPGRTDNEIKNFWNTHLKKKLIRV
GIDPITHRPRTDLFATLPQLMALASLGGLMDYRSLDDQLRSEAVKLQLANLQCLQYLLHS
SAAIGNSPNGNGLGGITGEIDTINFSNQPLATVASNPSHSPFTVVNARTQLHHLSEISCS
LEQPLGNDTKEYCSTFMDFSQGESKGVATTPVVSPHPSTLPPPLADASMGSGDACSSSSH
GGSVGTTPFWPELLFDEPLMSEFA*
>CnMYB10
MSEKRGSNKYEDMEMRRGPWTLEEDTLLIHYIACHGEGRWNLLAKCSGLKRTGKSCRLR
WLNYLKPDIKRGNLSPEEQFLILELHSKWGNRWSRIAQHLPGRTDNEIKNYWRTRVQKQA
RQLKIDANSAMFRDAIRCSWMPRLLEKMGSSQSMPPLIDSNAAATAGMIDQTPQGARPPQ
PFSDPAMQYPFENRSSYEPSGSENHSPSTSSGSMALPQLPYLSDFSSNPANSYINGLTFDP
FMGGHCIDHNGYGLDTMDPTPASASQLGSSISEYNYTMAANNCVDNIGGDGLWNMDEL
WYICKKAT*
>CnMYB11
MRKPCCDKQDTNKGAWSKQEDEKLIDYIRIHGEGSWRSLPKAAGLLRCGKSCRLRWINY
LQPNIKRGNFAEDEEDLIIKLHALLGNRWSLIAGRLPGRTDNEIKNYWNSHLRRKLINMGI
DPNNHHVSQSLSLHRPQISGSATLSGGPVNHERKEWQQLISMTDNDRASDTGSCLPSKKD
AASCLEDNAYVLPDLNLDLTVSVPSPPTTFTEWKQESAELEAPSELGNATNQTLLLFG*
>CnMYB12
MGRQPCCDKVGLKKGPWTADEDKKLVNFILTNGQCCWRAVPKLAGLLRCGKSCRLRWT
NYLRPDLKRGLLSESEEKLVIDLHSRLGNRWSKIASHLPGRTDNEIKNHWNTHIKKKLRK
MGIDPITHKPISSTDEHHRKEERQQEQPDVAADEHGRSENVGLQCLQGQEEEKSVTSSFDV
IETDTLLQKSPDFCTDEVPMIQPHEIILPCASATSFSTSIPSSGYLSGSSVKAEEIQFPCM

EMEWPETTYLCGMDEYFSQWDLISSDGDGT*
>CnMYB13
MMTSDGGKGADKAETSASASNAVQDGRGDESQRQRPLHGRTTGPTSRSTRGQWTSKED
AKLRRAVQKFQGKNWKKIAECFPDRTDVQCLHRWQKVLNPELVKGPWTKEEDEKIIEMV
NKCGPTKWSTIAQALHGCIGKQCRERTDNAIKNHRDSSVKKKLDSHLASGLLAQFQGLPY
VENPAQCISSSVVNQQKSEDSGVKDRQEVEDSSECSKGSSSALVGCSQSDYELAISVRDDV
QLAEAAQKKDIHDSRLSMCSKEYYASMEEIECIVPEIECEPMVSVTAPCQDIHETEISGRLA
SQMVSQELPNYFLEAAQRSPELKRTSEYHTHRVENHKNKSPLFPDSGGLKMPTCITNSGL
ESENQDKISMAEADCCSGSIFGAEMREDITMEDPITAPNIINMDYYLGSGSSQPDFYSSL
AYRNVVSCSQPPYPANSSEMLGNSYCQSLMTIVPPSYICPDDGKPIYRSDDAVARDISVG
TQYSELITCMYDSFAYSNCSSLSPNGGARSKICVHEDKHKENETPEQTYMEMMDSEPPTT
TDNIKSSDENPAIQTEEHQDAGALFYEPPRFPSLEIPFVSCDLISSGDLQQAYSPLGIRQ
LMMSSTPCSLWDSPSHDESPDALLKSAAKSFICTPSIMKKRQRELLSPLQEWRNDKKSGI
DMDPVLFSTPSISRTDNSSMNDEGVAPRISSCSSEVEALYPSNHQKKKHEICLEQKENLD
HTPSYQKDEDASVEAKARISSKESNSSFYQAKKEQCATAISAPTKLDANVAANEPPAGVL
VEHNTNDMLLFSTDQDGHPVNGHMRTAARSLEDQTFRSFEITPNNGNSDAQLALSEFSAP
LSPRVGESKQEQRQVLATSAQLALSVHPLDVSVEKHGSSIDMDLENLNIFADTPGIKRGI
ESPSAWKSPWFMNSLLPGHRIDTEITFEDIGYFMSPGDGSYDAIGLMRQLSEHTASAVAE
AQEVLASGSPGVDFDKRQSGKENFQKENIQSDKELGDHPVTPKVMMEARILDFSACATPV
RKTENKKVGNMGTSVNFSSPSSYLMKGYR*
>CnMYB14
MDKRPLKQARTRGYESEEVSSIEWEFINMTEQEEDLIYRMYRLVGDRWILIAGRIPGRKP
EEIERFWIMRHGEAFAEKRNSKGGLQKCG*
>CnMYB15
MTRRCSHCSNNGHNSRTCPTRAGGVRLFGVRLTEGVGSMKKSASMSSLYSAGSSATGGG
TGGGGSTNPGSPSNDPLRDNPAAAAGYASDNAAHASCSNCRSERKKGVPWTEEEHRMFL
LGLQKLGKGDWRGIARNFVVSRTPTQVASHAQKYFIRQTNASRRKRRSSLFDMVPDMPT
DQFPVLEEHLMHQSPPNEPENTNQLPALNHSQNQGPELAVPLLNNPPQELDESIPYNNTPM
PVPAFYPAFIHLPYAFWPPNLAAPATEEEMGETHEIVKPMPVLRKEPVNMDEVTGMSKLSI
GEHGAGRMEPSALSLKLLGASTSRQSAFHLNPSTARPDLSQSNSNAIHAV*
>CnMYB16
MRNPSSRSGPRPSSSLSSPATGSTSVTKQPQEGEPAPSKMGLKRGPWTPEEDEVLAKFVR
KEGEGRWRTLPHRAGLLRCGKSCRLRWMNYLRPSIKRGPIAPDEEDLILRLHRLLGNRWS
LIAGRIPGRTDNEIKNYWNTHLSKKLISQGIDPRTHKPLISPSSTNPDPPLPEGPEIQPP
ARKTYRNPKYPNPNANPGSTIPSVDRQMSGGHGGVGTHRDRDGFSGSAGLEQDDGWQN
TEGFAAMDLQGSQGEGDTGEDDILSSFLDSLINDDIFLQNHQQNVNYNDDDDDNFNNGQI
RSQDPSMPSAPGSGLGTLWESTYTSPVSRDEEIRGKVVDHAGK*
>CnMYB17
MGRAPCCDRANVKRGPWSPEEDMALKSYIESHGTGGNWIALPQKAGLRRCGKSCRLRW
LNYLRPDIKHGGFTEEEDNIICTLYNKIGSRWSVIASQLPGRTDNDVKNYWNTKLKKKMM
AAQAFLYNNITSSSSSSTTTTTTSNTNLIHHTPAPASLIIPTIKTEIYTCDDFLAPSNEGSS
ASAPVNPGFMFGHNTPFPPAGLVGPPGLNGSAGHYAVSPHDDASTASSSVNMENSSNNYV
NWSANGPGGDDSFFAEFFNDHYGYQEKTGEGAPNQATATVAANMWITF*
>CnMYB18

MKKKAERGGKKVVVVVVNKGAWTPEEDQKLVDYVRAHGDKKWRTLAAKAGLNRSGKS
CRLRWLNYLRPGIKRGNISQDEEDLIIRLHSLLGNRWSLIAGRLPGRTDNEIKNHWNTHLS
KRTLTIHDLNLKQPRKQEVVHGHSHPSNTPPEPSRRFKSGVSEDWFEAWLLDHNAELVNP
WIDLAGSEFNAEHLFDFMSMPGTDRNEGGSSGSELRVEDYGIIGQDGTFGFQGCVCDPSE
FDDLDRFVGCQDHEYYFLH*
>CnMYB19
MPSTGAPSILRGLALPPFPLPSSVDETNDSGWPCFSERACTELHVDFGGFVVNNYSVLEL
IKEEMELEAKVYVKLPTDGASISRFSAYHAEFLIAGKSGTTNPNPACEPNAQMHVDVKTK
STCALQLNEVSSYGDEHIPKVRKPYTLTKQREKWTEEEHKKFLDALQLYGRAWRRIEEHI
GTKTAVQIRSHAQKFFSKVVRESAGNSASTLQPIEIPPPRPKRKPLHPYPRNLGRTSSNG
IPVLKQLEKPSLQIPSPNEQENGSPTSVLSTIGSANLGSTFLNLPDSCTSPVASAAESNE
QDDGDQSPTMSVGEENKSQSLASGAIWLTIGEQPAEELGQHTDGHVHTEEISSMEAPETN
LKLFARTVLVSDSDKPSSSSTGDMVQFVKPSPTVDLEIHHGSSDVNLHAPTELPLQSATH
EDLSEVQGRAWNPWDGGMHPLIYGLPFVGENTTVTTFLSLPWWCFCRNLPFLFIHQQNTS
ITKHPPQSWTGRVDDKEIQRECSWTGSNTASVGEVGIGDQNAVVDSPKADNPMKKLAPT
MGPKPTDHSTFDIKNSGSDKPTRGFVPYKRCAVEREVQHSQTSNEDGEDQMIRLCL*
>CnMYB20
MVAAVKREDAKGEGIQEATPTSNIGSETKPMTIPGRTSGPTRRSTKGGWTDEEDDLLIKA
VRRFNGKNWKKIAELFPDRTDVQCLHRWQKVLNPELVKGTWTKEEDDLIIQLVEKHGCK
KWSVIAKSLPGRIGKQCRERWHNHLNPTIKKDAWTREEEEILVRAHQIYGNKWAEIAKYL
PGRTDNSIKNHWNCSVKKKLDSYLASGFTDQPLETVDHVQNAECPEADPLKRSTNGFISC
DWKLNADGLITSNVSLVHQKTSPKRRCLDLQSTSRGDSKCLRAGSIPTLPVDSKHLSIEVE
SNTEDDSKYTTSAETDYESSKRSQSNLSSSGELDLGDMNKNNRDTSISSLPVCEQSDSDR
TADINRILTTPLHCKNRNLGSSCYAPMQLMNVDGCFSSEKLSRSNNHIQPTPSPIHCSTP
PDLSLSLACIPSSPESILRSAAKDFKKTPSIIRKRGRNSSKPLFLEKLKIETVGAKCPSG
ISNFQWRAKNFKDDLESLYVHQIGSNNQVNFNRSVYLSDDQLCSSNNFLKHQDSVATKSV
EKRLEGEFNAECGLSL*
>CnMYB21
MGVLPVVNTYPLSSYTFGTKEPKMEKDTSVADRLARMKVNYMKEGMRTSVEAILLVQE
HNHPHILLLQIGNTFCKLPGGRLKPGENEIEGLKRKLCSKLAANSPSHQPNWQVGECVAV
WWRPNFETVMYPYCPPHITKPKECKKLFLVHLSEREYFAVPKNLKLLAVPLFELYDNVQL
LAYRHLAAMGCKTCEKPKVGYRKGLWSPDEDQKLRDYILRHGHGCWSTVPAKAGLQRN
GKSCRLRWINYLRPGLKRGFFSAEEEATVIKLHATLGNKWAQIAMHLPGRTDNEVKNYW
NSYLKKKVVKLGGSNSHASPTKSQDSANQSPETRQVPEEVIKQIPGSDFLEPLEPPMQSSM
DQDSFKGTLLPPFPKVLFADWLSMDYNSSQSLLNSNAGMNCQWDSNSNADLFKYGSYQ
VDGPSSSDFLHGFGDSSFCGEFQLQFEHGGQILGGAFYNYLSMDENGGSCGRNHDAIY*
>CnMYB22
MGRAPCCDKQGLKKGPWTPEEDKILVDYIQTNGHGSWRSLPKLAGLLRCGKSCRLRWTN
YLRPDIKRGPFTPEEQKSIIQLHGIVGNKWSTIAAQLPGRTDNEIKNYWNTHLKKRLLRMG
INPDTYAPAASPSSPGATGGGGGGGASSSSSAFPATRHMAQWESARLEAEARLSRESLLF
SSASTSAAAAAAGGGSIHSDSPHPKSEPDFFLRIWNSEVGDAFRKPLSAPRESAPLAGSS
PESSSTKQSSAVTVSPPAMAEAPVVKEEPSTESKSCVVGGGGGGGGDQASAGADSSGSNE
VEDTSEETYQLYLDFVGDDLGLFSGQLGSFSLFSSDLHDASLDTAFK*
>CnMYB23

MGRAPCCDKANVKKGPWSPEEDFKLKEFIEKYGTGGNWIALPHKAGLKRCGKSCRLRW
LNYLRPNIKHGEFSDDEDRIICSLFASIGSRWSIIAAQLPGRTDNDIKNYWNTKLKKKLLGI
APSQRTPHQQPQQQKQLQQQLLFPSSSSSSHGSYYTSPIPPLPVLESLPPISQDAFGTVP
TTTFPALLQGPSYQYQMMKESGHMIMFGSEQSCSSSDGSSTQISHGKEMDYSYIGRGGGA
EQMGLESYLCAAGIEETHRWLLGGGGGGRQVESDNGLSRLWEEAPLEYNYEEIKQLLGS
LNGYTNNNGHNLSVDDPTGSGGRGSFKSQGRVM*
>CnMYB24
MGDFGRRRKYHVFIIVFFFFFDTRIKGSWSLEEDALLMRLVERHGLQNWTLISSEIFGQS
GKSCKLHWSSVMPSTTTHSLTSGTPPSSPPIQSMATSGPPSPASSSAAPTMLSRITGTPP
SATSDSKSDVKWPCKIKDEVSESEVATMVVVLPAPPPPPWPPMAQIPVDLVMLLMLCPPG
ESAGSGDGWDRREGWWDAEMREMCLVPIMWEMIVEEVRSYINREGGGAGAVVMGRDL
RDGVMVEEIFFYYGGRSWEGGCGWGAQVKWACEGSGSVFLLF*
>CnMYB25
MLYISVLTSMVTSTSGWQLQNSRLYSSGFTTSTDRVQLEPASRCWNVRIGTMKWLMRGQ
KRSVYRDSFMMPGKSVRRMRKKPMNVGLTSDFCRSGLRRSGKSCRLRWVNYLRPDLKR
GKITPHEENIILELHALWGNRWSTIARSLPGRTDNEIKNYWRTHFKKSKPSKNIERARARFL
RQQQEQPKLKLEEQRQEEQQQADVRGIMTQVEEATLAPQEMQEMAYMYPLTSMLQWGG
CVGDGSCSVSDGSSEEEVWGSLWNLDDIHDDLDGACRAGVTLQDQVLTFY*
>CnMYB26
MEGSSSGEDLIVKRRKPYTITKRRERWTEEEHNRFLEALKLYGRAWQRIQEHVGTKTAVQ
IRSHAQKFFSKLEKEALAKGIPLGHSHDIEIPPPRPKRKPSFPYPRKVDVGSLSPFEESK
DEKVSNSAASQEKPVATEIQQRTKWTSGSGSCSEVFNLLQDVPSASMSSANKGSSNICTF
QHYVKEVKEKTAQSESSKTVKDNHEEKKEADHTDIDVGRLSRISLHSQSKFAHEERRDAR
KQTENFGPSLEDNQQGGQCYSKLIPGQFIDTNSAEYKQNVCSNEFIMPVTNQDEAHTDVN
SFKNPVTLAGHESHSNCMVSSAHQPVPVFRPCTQFGSNQDAYRSFLSSASSSLMLSTLLQ
NPSIHAVASLAASFWPSVNANAPLDLAAENLNGGMSEKQSNPSPSMAAVAAATVAAASA
WWATNGLLPMFPPFHTSYSFAPPSTVPVPSVDIAQAPKDKKEKKEDRPQNLLQEDHQQIM
DPGHSGALRNTQKASKLLPSSESELDECGRGERSPYAKLKSSAADKFEALPDTRFLDSDKT
QHNKNSNCSTYGSNRLSSSEVEPESMIKKHKEVNDEGEQVHLDNHLCGEAGNHRLGNSR
VTNGSWKEVLGEDGPAFQAHTTRELMPQRFSQLQTNDDEMPNLCKDKEVTGLLVDLNG
KACESTNEFKINSADTLVSEIEHGKLKASRRAFKPYRRCCAEAKDNQGTTDEEKSDQRIR
MEG*
>CnMYB27
MVCVDPMDDGGEGGASGGGGGGEGSGGEGGGFFLGSRQHQQQHGGPVDVALLGAADS
LTTAGSAAATVPEDPNKKVRKPYTITKSRENWTEQEHDKFLEALQLFDRDWKKIEAFVGS
KTVIQIRSHAQKYFLKVQKNGTSEHFPPPRPKRKAAHPYPQKASKNALLLSQTTIPFQTSS
CVLEPGYALGTDTSSILGNSSASATVSSWASGSVQPNASHITKVMPDFAQVYSFIGSVFDPS
TGGHFQKLKEMDPIDVETVLLLMKNLSINLSSPDFEDHRRLLSSSDVGIKELKPGNINNM
LQADKPMSAPSIVKGH*
>CnMYB28
MGRAPCCSKVGLHKGPWSSEEDALLTNYIQCHGEGNWRSLPKNAGLLRCGKSCRLRWM
NYLRPDIKRGNIGRDEEDLIIRLHSLLGNRWSLIAGRLPGRTDNEIKNYWNSHLSKKLKRQ
GIEVREGTYKGTSKRSRISPKRSDSVNNSNNHDSKNQQDKRKKKNEGEDTAEITRTKIYIP
KPTRFTPNLRNCNLIENGGVSEVPKTESAASQEEKGDISMEVQTEDLGVNWYDINQGGFF

FEENDPNHVLAFDFPSKDRLLEGLYQDYYELLNSEGNQAQLNSFPDQSLFLW*
>CnMYB29
MGRAPCCSKVGLHKGPWSPEEDALLANYIQCHGEGNWRSLPRNAGLLRCGKSCRLRWM
NYLRPDIKRGNIGPNEEDLIIRLHSLLGNRWSLIAGRLPGRTDNEIKNYWNSHLSKRLRAQ
GIEVREGTSKGTSKRARISQKRSDNVNNSINNHNSKNQRDKRKKKNDVEDTAEITRTKIYI
PKATRFAPTLRNYDLIENSGVSEVPKTESATSQEEKGDISMEVQTEDLGVNWYDINEGGF
FFEEDDPNHVLAFDFPSKDGLLEGLYQDYYELLNSEGNQAQLNSFPDQSLFLW*
>CnMYB30
MQEARGGSGHASSSSKTNNNGKEEGGGMVMKKGPWMAEEDEILIEYVRKHGPRDWSSI
RSKGLLPRSGKSCRLRWVNKLKPDLKTGCKFSPEEERIVIDLQAQFGNKWARIATYLPGRT
DNDVKNFWSTRQKRLARILRTPLPSRSNKNHGKLPLSSHQVPTLEDPSLDRILLEEDGRCK
QKAAKMTQIPDIMSRVPLHLDTALPLLELELAMEKEPCLSDPAPTSQPPFDHLPHPLVDF
PLPVLPEDAQGLSPGFGDAIFAGGLACPETPPEGLPFLGLDAGHDGIERVDEHPGTSSDF
FDDLPADMFDYLEPPPPPPPASSTSW*
>CnMYB31
MEKGQLERREKGLKLFGVRIVGDGGAGELELEEEEVMRKSSSMGNLASCTADHAGDHV
GGEQGYLSDGGLHQSSRMRRRHERKRGVPWTEEEHRTFLAGLEKLGKGDWRGISRNFVT
TRTPTQVASHAQKYFLRQNNPGKKKRRSSLFDMVIDDEAAVPESASALSPKKPREAKEET
NNLSLDKHMMGSPALVTTQVASRCLDASSVSPLTTSHGSITNFSSANLMVGISGSSVGEQV
IPTLSLISPLDVPEQVATVLQASTTYAPNFPKWSLPHPSSSATSSLIESSDLELSIAPPRPH
SLTKLSSQGVAEAIRVV*
>CnMYB32
MGRGRAPCCEKVGLNKGSWTPEEDMRLMAYIQKYGHGNWRALPKRAGLLRCGKSCRL
RWMNYLRPDIKRGNFSKEEEETIIKLHGLLGNKWSKIASCLPGRTDNEIKNVWNTHLKKR
LLSKERSQKTDNLEEMPSLSSSTTSHSCSDQVEGKSDSEHTNPSVDSINPSEDKIEIPIEPSM
DIWVMLEDAFPSSPGNIETEDKNMLEMQLEHNSFSDSLATNVGTSGGGQALDDIDPKEAE
KDPLEVPDVPIEPELWDMIQDGDAGLFSPEVGSMSELGVHGNPQSLDEDPGRGEGSRIWV
EYLEKELGLWGASDGNLECPMGPWAEMEGDPVSRYFQKGPSTPPSLDLHDLKVS*
>CnMYB33
MGRSPCCEKAHTNKGAWTKEEDDRLIAYIRAHGEGCWRSLPKAAGLLRCGKSCRLRWIN
YLRPDLKRGNFTEEEDELIIKLHSLLGNKWSLIAGRLPGRTDNEIKNYWNTHIRRKLLSRG
IDPATHRPIHAPLSDFTITFAKGEKKDVGILRRDEEKTRSSCSSEESAPWRQYRCPDLNL
ELSISPPFQQPVEPIKGKRRLCFTCSLGLQRTKKCKCNGFLGLGAGVLDYRTVEMK*
>CnMYB34
MVRAPCCEKMGLKKGPWTPEEDQILTAYIQRYGHGNWRALPKQAGLLRCGKSCRLRWV
NYLRPDIKRGNFTEEEEKTIINLHAKLGNRWSAIAARLPGRTDNEIKNVWHTHLKKRVEPN
QVAQEAKKKTQSDAKHTPMQLSSGLMHPSLDDPRLLPASPEQSHSDFSCTATDSSTTSRET
TSNVGVKEESFSSSEEFPVIDESFWLETLSMDNCAEQIDFTGLRGPLGERFGSFGASNND
DVDFWLRVFVEAGDLQTFPPI*
>CnMYB35
MGHHSCCNKQKVRRGLWSPEEDEKLMKYISTNGHGCWSSVPRRAGLQRCGKSCRLRWI
NYLRPDLKRGSFSPQEESLIIELHRVLGNRWAQIAKHLPGRTDNEVKNFWNSTILHSCCSIP
ISSFSFITPLPFIPSCLSRRPLPYSRSIHPWISIIAMLPHVHFCPFQVSTLSILPYSSTW
PSDPHLSFHKAP*

>CnMYB36
MDKRRRQKHSRTCHCDAEEVSSMEWAFINLSEQEEDLVYRMYRLVGDRWTLIAGRIPGR
KPEEIERFWIMRHQEGFAEKRLKRSS*
>CnMYB37
MSGRSSTPEEVHPKGVWGESSSQRSPSEGYRPPLDIINTILQGLQQGEASGSDESNGGVN
LLFEDEVFDEWAQIAMHLPGRTDNEVKNYWNSYLKKKVAKLEGSNSHASPTKSQDSANQ
SPDTRQVPEKVIKQIPGSDSLEPLEPPMQSSMDHDSFKGTPLPRFPKVLFADCLLNSNAEM
NCQWDSNSDADPFKYGSYQVDGPSSSDFLHGFGDSSLF*
>CnMYB38
MGRAPCCDKANVKRGPWSPEEDAVLKDYIERHGTVGNWIALPQKAGLKRCGKSCRLRW
LNYLRPDIKHGGFTEEEDLVICTLYNKIGSRWSVIASKLQGRTDNDVKNYWNTKLKKKVI
TGQISLPTNIATSSSPSPSNSTYSTSTASIHHSPSPAPPLQPPLLPATITQAYGGGADFSVP
LEKRLGTNFGFPQKLYPEMAQFSMMGLMEQRDFSGSVSVSVSPSEDMSAASSSPITVDNS
SSSSNGYVNWSTDGVGGDDAFLSELGFGPTGDFYGGYGSQERMGEVSPYDYSDLATIWA
ANETKPQEFYQGVAYS*
>CnMYB39
MGRRCSHCGNNGHNSRTCSSPRGVVGSGGLRLFGVQLHVASPLKKSFSMECLASSSYLAS
SSPCSSTSSSSSLVSIAETTEKFSNGYLSDGLLGRTPERKKGVPWTEEEHKSFLTGLEKL
GKGDWRGISRNFVTTRTPTQVASHAQKYFLRQNSLNKKKRRSSLFDVVASCDGADQVTD
SSKFKEPCSSSELHVPTLSIKTRDYGASEITAVDLNSSEQEAQLPSSLAPMPKSSFSPLSR
SSLAKKPHVHLSPLNLELSISSARPLD*
>CnMYB40
MASSSLSSSWTPKQNKLFETALAKYDKDTPDRWHNVARAVGGKSAEEVKRHYERLVEDI
RQIESGHVPFPSYRSSGGRGTLFHFLLITVPFPALSSLALPWFSIPLPLSLLLRFHIVFL*
>CnMYB41
MASSSMSSSRTTDTSWTARQNQLFEDALAKYDRDTPDRWQKVADAVGNKSVEEVKRHY
ELLIKDLNDIESGRARYRN*
>CnMYB42
MASGSMSLSRTSDSSWTLRENKRFEEALAKYDKDTPDRWYKVAQAVGSKSVQEVKRHY
ELLIRDINYIETGHVSFPN*
>CnMYB43
MVDEANRMDKRINSSQEAEVRKGPWTKEEDLILINYIANHGEGAWNNLAQSAGLNRTGK
SCRLRWLNYLRPDVRRGNITLEEQLLIMDLHARWGNRWSKIAKQLPGRTDNEIKNYWRT
RVQKRVKHGESFDYHQAMVTDEASASASTSQTNSVEDVGPQPSYIEHQAPNPDALAPHFS
STESSNIFWSVEDFWSMQSLNGD*
>CnMYB44
MDRRINSSQEAAVRKGPWTMEEDLILINYIANHGEGVWNNLARSAGLNRTGKSCRLRWL
NYLRPDVRRGNITPEEQLLIMDLHARWGNRWSKIAKQLPGRTDNEIKNYWRTRIQKKVK
RGVSFDYQHAMVTDEASTSTSTSQTNSVEDVGLQRSYTEHQATNPPPLAPPFSSTESSDNF
WSIEDFWSMQSLNSD*
>CnMYB45
MSSSSAAAAAADGGGAGEASWNREEEKAFENALAMVVRPVKAEEGEDGWWELLAERV
PGKTAAQVRRHYEMLVEDVKAIEAGRVPIPRYIGEDAAAAGKEKDHHHYNHHGFGGDRR
FEMGGQGKSLSKSEQERRKGIPWTEEEHRLFLLGLSKFGKGDWRSISRNFVISRTPTQVAS

HAQKYFIRLNSMNRDRRRSSIHDITSVNGGDVSSPQGGQGNANPAAGGSSMKHPSQPNIS
TVGMYGPSVGHPVAAHMVSAAGARVMLPPHHAPYVMPVAYPVPPPTTMHQ*
>CnMYB46
MKERQRWQPEEDALLRAYVKQYGAKEWGLVSERMGRALHREPKSCQERWKNYLRPGL
KKGSLTPEEQALLVSLQSRYGNKWKAIATHLPGRTPKRLGKWWEVFKDKQLKAAAKASP
DKPHHPGTSSAYEHILETFAEKHVLPPPSHPPLPSLVLPNPLLFAAAAASSCSSSSPSPSSPSP
PSVSFSLSRPEGWLLLLPAVVQLCAEVEEGRQSWGQHRKEAVWRLSRLEQQMETEKARK
KREKVEEVEAKIRRLREEEAAWLDRLEEEWRERVLAVRREAEAEEAEVVEAWTATHAKL
AGLLEKMMLGGADGRGLSLPVAKDYLH*
>CnMYB47
MGRHSCCLKQKLRKGLWSPEEDEKLYNHIMRFGVGCWSSVPKLAGLQRCGKSCRLRWIN
YLRPDLKRGSFSQQEEDLIICLHEILGNRWSQIASQLPGRTDNEIKNYWNSSLKKKLRQRG
IDPSTHKPLSEIEAPVEKPGLNNSSSSGSLEHLPAKPVFDPFPLIDVQAGVDLMETDSNL
CGEIQQTFKPFNQNELLTSSGFCDYSSLLDVSENYGYGESSSNSSNWNRNIGTDTNYVLG
NEVLNWVSDNKVGSLTHTHNNGVETFEHKISPWHEKQHHVQSTEDYNSYPMRSLSRDLT
EACLDIPRGELASEFNVDFF*
>CnMYB48
MGGRLGFLIPVSRFDEFDIWVLKEKDKRCWERKHEVIVEESRDMLDGVTFAGSLRNGEV
VIFKISGRECLIGRDVKHGKEFRVLVSNGCIEGISFLPTSPGKGINQHENGKVHLNLKVSM
LPPSAKVPQVTERQEQSRFQMLTAKRQLINRRDEESTDKEEWLPERWETAHNILPNPRAI
SLRFSITKDSYFGRKLKVLNPSVPLFPEVLVFNELNDEVKVKKSLLSSDGRILEKNDRTP
VMIADFGVQALISLGMLSLKRLFPSIPLVAEEDSGFLRSNYVNAQSGEVRGSNFLVDSVL
SAVADKTSSVDEPLTSDDVLEAIDRGGKDAVSFDAEPSTYWVLDPIDGTRGFLKGSDALY
VVGLPLVVKGEIAIGVMGCPNWKEDNLSTKKPDDNFRPGVIMIAHSGCGTWARRFPDVIS
NYKRIQDSWRRCFVDSCWLVHEARFCIPDSQTWDFMPLSVPFSTSVDCGDIRDKQKVLLL
PTCCGSLCKYLMVASGRASVFILRARAQTVIKVWGHAVGVICVHEAGGQVCEQPRSLSSR
EACLLLAFLMSCVYCLHCACACASIWLARCGKSCRLRWTNYLRPDIKRGRFSFEEEEAII
QLHSMLGNKWSAIAARLPGRTDNEIKNYWNTHIRKRLLRMGIDPVTHSPRLDILDLSSLL
NPSLYNPNQLDYLSGLLGLEPLVNSELVRLATNLLSSQCQNSNLVGQNFQGPALPNPQVQ
DPYPSSPAHHLQNPILQEFPPCTTSTAQFLDESQLMQANEHHFQPEAQNNLWQEIVTQAN
LSENCVPTPNVIYDGLDPSIPQLISENSSFPVISNPNCNITSVLSTPASSQTQLNSSTTY
VNSCSTEDERDSYCSNLFKFHVPELLNVSDYMYRK*
>CnMYB49
MWKEHSSTAFYYDYSFVLGDSSNALTETRCQCGATPIQANEASSLRGSRSCSFGSEGITV
GVCCTYRKMADLDQSSEDVSVDTRDGNSQDIKVDFTEDEERLIARMYGLLGDRWPLIAG
RIPGRTAQEIEKYWTSRYSNSE*
>CnMYB50
MKRGGRAEASSADAAAAAAAKGAGRPPGQAPGTAGLKKGPWTAAEDAILREYVKKHG
EGNWNAVQKNSGLNRCGKSCRLRWANHLRPDLKKGAFTPEEELLILHLHAHVGNKWAR
MAAHLPGRTDNEIKNYWNTRMKRRQRTGLPLYPPEMQRQFLLRHGYHLPPPAASSSASP
DPPPRPPPPPRPPALALPEPTIFPSSPSPTAAFSSSPSAATASIPLLPQDHFSNGLSFQFPLSSPV
SPPPSAPPQQFPLSRPVSPTPPPKFSLSRPVSPTPQSPLFPPQLPQPLRYNLGSFDVSPP
PSFMQMPFVGELPSSQMLGETGGGSGSAGFGLLRSSSTGLLDDIIRDVQRGGEVRRADPL
SELVPSTGGPHGKRDDHLGSNDVSEGSSSFSPAQGSVLESLSMLDGSDCSINGIEMRSKP

TNEIGPAHDDISMLFDMIPVPTFPATTVVPDWYTSDSGEISNGPSSVMTDDDMGLDMHQL
APSYAFAPMSQDWDISPCTWSDMSGIC*
>CnMYB51
MLGNEMAVRSIPTGNPGQLQGLLRCGKSCRLRWINYLRPDIKRGNFTKEEEETIIRLHGL
MGNKWSKIASCLPGRTDNEIKNVWNTHLKKRLAPKGPKSDPSTNKTKDPPSSPSNSNNIS
CDNDGNIKIDEDIKQPNFEPPEPLEENNSFDMEYCCQYSVNIMSELFMELDQCMLIEGFD
YPSGNDRRDSSSISSLESPITSCDFQEKSVHQPLQDDGKDINQDYLNQLEPEIPIHAEIW
SMADDDFCFLTPEVGPVVEGGAHDNSISSEGDSTREVDDKGWVAYLEKELDLGGTMDIV
SAVEQPGPWEYHGGALEEFDPVSCYFQKTHCPSSTTDIISQFVAMAALKGGLQKNDLLFFL
EKKYLRNFTDLLARAKGYARVEEAFKLKDEEAIGER*
>CnMYB52
MVPEEDAKLKAYIEKHGTGGNWITLPQKIGLKRCGKSCRLRWLNYLRPNIKHGGFSEEED
NIICSLYISIGSRWSIIAAQLPGRTDNDIKNYWNTRLKKKLLGKQRKDHQQQQQQARRSG
GMKQQAQRDESGNIRVLGEGGSQSTYWPEPPVPVYSTGQLDRRADDDHASMRQLLMKR
QERFSNDNNASLRSPFIPPSSFTLPQPFDDSASAMTTLVNATTPSFGEGLLQSPNGFAAEPD
ELLHPVKLEGFDWLYGVGGMVSDHHGSTTSESVNWCEISPLICPNAVPSYQGMQQCFFEE
PVHLIMQ*
>CnMYB53
MGRQPCCDKVGLKKGPWTAEEDKKLINFILTNGQCCWRAVPKLAGLLRCGKSCRLRWTN
YLRPDLKRGLLSESEENLVIELHSQLGNRWSKIASHLPGRTDNEIKNHWNTHIKKKLRKM
GIDPVTHKPISSTVDQQQQQQQGCASVDGNDKNEQLCLQSPPIMAQEEAEEKSITSSFDPI
ETDASFSKSPVFCTDEVPVIEPHEFVVPCTSSTPSVSSTSISSSCFSSSSEPKAEEMQLP
CMDFPESLYLWGLDDFMGLEFLNDDGDGKLSLDPSSQYQRTSLDQESWKFELF*
>CnMYB54
MGKSCRLRWYNQLDPRINRNPFTKEEEERLIACHQSHGNRWAVIARLFPGRTDNAVKNH
WHVIMARRRRERSRFHGKRALRSLNKEESSKQNSEEEPELRNMVPFMENYSGRYQNHLA
LNNHHDQYCFDMYEGNGIKLSHRDNSIEFHDFLQVNSESNNTEDTSTRKEEQEEGKEQES
EASVRFIDFFAVGCSQ*
>CnMYB55
MGRTPICSQGLNRGAWSLEEDRILASYIETHGAGKWSNVPKEAGLNRCGKSCRLRWLNY
LRPGLKRGNITQEEEDLIIRLHKLLGNRWSLIAGRLPGRTDNEIKNYWNTILRKKVQQGTL
AQAQELESGTSDQTRAVRTMATRCSGSMLIDLKVDGEMQLEGNAAAGDQNCGGGESVPS
SDNPSLFHEDMVDGWMRGEEIQPSFSWEFSSLACFMGTEDFLWSNDVFP*
>CnMYB56
MGGGACNSKERLNKGPWSATEDKILGNFIKTHGEGRWGKVPKRAGLNRCGRSCRLRWL
NYLRPDIKRGNISQEEEDLIIRLHKLLGNRWSLIAGRLPGRTDNEIKNYWNTVLKKKVQAR
SVSLTKLACLNKDEERESKDETSVQAPLEDPGTNQEEGNQNVHQSGLESLVIEDLGSDGSP
PAPQDYDLPNLWTGLDAEGLSKPCLLDYEFFQVLGGNIQQDMRGCNSSKLSVSSDENVLL
SRVLENGVGTDHIQPYGGLDLGSLATFFESES*
>CnMYB57
MGRKPCCSKEDINRGAWTAEEDRILKAYIKANGEGRWRSLPKKAGLKRCGKSCRLRWLN
YLRPDIKRGNITCEEEDLIIRLHKLLGNKWSLIAGRLPGRTDNEIKNYWNTYLKKKVQGQ
HQAKRCQPAAKELAPELVRTKAIRCATVFIPPQHATKEPAMIGELATEKVANSRSNDHSPI
DPREDAWWDVLVGLDKSNGSTAPGRDLDLMRPWGSESPCSHYTRREMFEDWMSDATLP

FDMELKSLADFLNCEDEWL*
>CnMYB58
MALKQRNGSQGLTSFPPSPSSSTGLFAGMGSLSLNPTSSVGSNCSNKGSEKTFWTSSFMR
NYIGLEDHPPQNSEDEGGKCSDAIDGFGENHIDAVGEESPEGENTNEAGREAADCGQSKL
CARGHWRPAEDSKLRELVALHGPQNWNLIAEKLEGRSGKSCRLRWFNQLDPRINRSAFTE
EEEEKLMAAHRLYGNKWAMIARLFPGRTDNAVKNHWHVIMARKYREQSNAYRRRKLSQ
GVHRRLEDATATSVTCGFNTNLHLPFTHLSLAMGGSNGSYHITTGGGGEDSVSFHSGFCS
DRAPYLFLSGIKAQGKTTSFHSTNNWRWDRPGDEPFIGSYTQHPFMVAMQQPSYPSYISD
CSGASAASGASATESSSSSSFKENGENGQSEATTSPPFIDFLGVGAT*
>CnMYB59
MGRPPCCDKVGVKKGPWTPEEDIILVSYIQEHGPGNWRAVPAKTGLLRCSKSCRLRWTNY
LRPGIKRGNFTDQEEKLIIHLQALLGNRWAAIASYLPERTDNDIKNYWNTHLKKKLKKLE
TGADGLTTSRVSSHQSISKGQWERRLQTDINTAKQALCEALSLEKPNCLSSFKASFSGSN
SSTRPSTYASSTENISRLLEGWMRKSTRPGACQANSGSTQTDSACSQGTPSATNCTMSLE
PLEQLFGPDSSTPEASETSLVGEERKLVLEAQEPLSLIESWLLDESVGQGQLSLLDMALD
DASELF*
>CnMYB60
MEAAGMNSDGAVAPAAAGAVGTEAAPPKKDRRIVSWTPEEDDLLREQVALHGIVNWTKI
SSQFKDKSGRQCRRRWNAYLNSECKKGGWSAEEDKLLCEAQKVYGNRWTEIAKVVSGR
TDNAVKNRFSTLCKKRAKNEALFKENSGSFLNPNKRVIIQNGCITVQTRELSIPTNQMRYN
NSDRKENKISEQYLGNHEMKKDQLRSPLAVLAQNCDIAGGLLTQNHGGNDSRTAVYDDN
KTTGTFLRKDDPKLTALLQQAELLSSLAQKVNAEDSKESLDDAWKEIQDYLAQTEETEILS
RTISGTEFVLDDFTDLIEDLKGTNTIGLQSLRQSELHQNSQGTSEYSIGSAEHFETQGNERD
HRTDNYSSNNNMEVNGPHKDVQLPSIATSQEEVLPPTKKPKENDENSCRVSNSQFASPLP
TTPPFRSLADEIPTPEFTASEKRFLLGLIGLPSPAPSPKSSQQPTCKKALLDSL*
>CnMYB61
MGRSPCCDEIGLKRGPWTPEEDQKLIQYIQKHGHGSWRALPKLAGLNRCGKSCRLRWTN
YLRPDIKRGKFSSEEEQTILHLHSILGNKWSAIATHLPGRTDNEIKNFWNTHLKKKLIRAG
IDPVTHHPRTDLFATLPQLMALASLGALMDYRSFDDQLRAEAVQLANLQCLQHLLQSPAA
INGSAIGNNLGSITSEMDFSTNLPNQPLTTMSANLSQTPFTVVVNAQTQLHHLSEILCSL
EQPLGNDAKESSTSIDFRQRESKSLATPMVSPPPPPALPPLADASMGSGDACSSYSYGGS
SVTASFWPEVLFDEPLLTEFA*
>CnMYB62
MGRQPCCDKVGLKKGPWTAEEDKKLIDFILTNGQCCWRAVPKLAGLLRCGKSCRLRWIN
YLRPDLKRGLLSESEEKLVIELHSRLGNKWSKIASQLPGRTDNEIKNHWNTHIKKKLRQM
GIDPITHKPISSTDEHHHQTGQQQQRQQQEQVGVVAGEDERSDNMILQCSQSQEEEKSAS
VSGSFDFIEADTFLQKSPGFCTDEVPMIQPDEIIVPCASSTSHSSTSLPSSCFSSSPSVKA
EEIQFTCMEWPEPMYFYGMDDFNQWDLISDDRDGKLAFYPLNQLHQRTTRDQESWN*
>CnMYB63
MASDKEKGAKKGETSASPSTAVQDGSSDEIQRQRLLHGRTTGPTRRSTKGQWTPEEDAIL
CRAVQKFRGKNWKKIAECFPDRTDVQCLHRWQKVLNPELVKGPWSKEEDEKIIQMVNKY
GPKKWSTIAQALPGRIGKQCRERWHNHLNPAINKQAWTQEEEIMLIHAHQIYGNKWAELT
KFLPGRTDNAIKNHWNSSVKKKLDSYMASGLLAQFQGLPHVENPAQCISSSVVNQQNSD
NSGFKDRQDVEYSSECSQGPSSALVGCSQSDYELANTVQLHDDIKLGDDAKKEDVQNSQ

LPMCSKEFYTSMEEIAHVLPEIQCGDEVSVTIPCHSVHEVEVSKNLANQMISQELTNSLLES
TQKSPELKRTTEYHAHCVENYESKSGSLPDPVGMEMPTSVTNSGLGSDNQDKFLIAEADC
CSDNLFGAAMHQDITLGDPFTAPSITNIDYNSGSVNSQPDFFSSLDYRDLISCSQPLYLIN
SSDMLGNSYCQGLMTVVPPSFVCPSDGKPTYGSDDVGMREISVGTQDSELIACTYGGFAY
SSCSSLLSPSAGAGSKICVPADRGKETESPKQTYMEMMGSEAPNATGKITSQVENPAIQT
EEHRDSGALFYEPPRFPSLEIPFVSCDLISSGDLQQAYSPLGIRQLMMSSTPYSLWDSPS
HDESPDALLRSAAKSFICTPSIMKKRQRELLSPLQERRADKKSGTDMDRVLFSTPSISRT
DNSSLNTRNDEGGAYRNSSCSIEADAFSPSNHQKKKHEICLEEKENLDHALSCQKDESAS
MEARISSKASNTSNFQSKMEQCASTISVPTKLKVNVRETEQPAGVLVEHNNNDMLLFSTD
RDGHPINGHVSTAARSLKDQTFRSTEATPNKDHSDVPSKSLSDLSAFLSPGVGESELVQH
QVRVTSAQCAPSVHPSEVSIEKHGSSIDMDFENLNIFADTPGIKRGIESPSAWKSPWFMN
SLLPGHRIDTDITFEDIGYFMSPVDRTYDAIGLMRQLSEHTASVVAEAQEVLASGSRGVV
SDKRHSGEENFQKENIQSDKELEDHPMLSKVMTEARVLDFSACGTPVRKTENKKVGNMG
TSVNLSGPSSYLMKGYR*
>CnMYB64
MRKKALVGSLLVPAPLFHSMVQSHTLAHGTFGSRNYGSFRYGFCFCPAVILLTSMLLQWI
GAMDKRPRKQAKTCGYDSEEVSSIEWEFISMTEQEEDLIYRMFRLVGDRWDLIAGRIPGR
KPEEIERFWIMRHGEAFAEKRNTKRRLAEVQLSD*
>CnMYB65
MTRRCSHCSNNGHNSRTCPTRAGGVRLFGVRLTDGVGSMKKSASMGSLYSAGASSTAGG
GGGLGCSPNPGSPSNEPLRHHQAAAAGYASDDAAHASCSSNCRNERKKGVPWTEEEHR
MFLLGLQKLGKGDWRGIARNFVVSRTPTQVASHAQKYFIRQTNASRRKRRSSLFDMVPE
MPMDQLPVLEERFMHQSPPNEPDNTYQQPALNLCQNQEPELAEPLTKNNPPRELNETIRY
GNTLMPAVPAFYSAFIPVPYAFWPPDLTARAKQEMGGMHEIVKPTPVIPKEPVNMDEVAG
MSKLSIGEHVAGRMEPSALSLKLLGASASRQSAFHLNPSVGGPDLSQSSSNAIHAV*
>CnMYB66
MRNPSSSSGPGSSSSLASPPMMKQRQKGLPTPCCSKVGLKRGPWTPEEDEVLAKFVRKEG
EGRWRTLPKRAGLLRCGKSCRLRWMNYLRPSIKRGPIAPDEEDLILRLHRLLGNRWSLIA
GRIPGRTDNEIKNYWNTHLSKKLISQGIDPRTHKPLISSSSTTGPDPPLPQGPKIQPPAA
TMPYRNPNPGANPSPPMPSLLQQTSGDQGGPGIHDDQDGFFGGLSLERDDGWRDSDGGF
AALDLQGSRGGDDGGEDGGIDCCTDDVFSSFLDSLINDEFVLHNQQHNVYYNDNHNNSA
KDRQIQSTDPVTSSEPGFGLGTLWESTFTSPVLGLDEEIHGPLVDHAGK*
>CnMYB67
MGRAPCCDKANVKRGPWSPEEDMVLKSYLERYGTGGNWIALPQKAGLKRCGKSCRLR
WLNYLRPDIKHGGFTEEEDSVICTLYNKIGSRWSVIASQLPGRTDNDVKNYWNTKLKKK
MMAAQAISLSTTTTTTNINLITHSPSPSPPPPLVIPTIKTESYTCDDFFTPFNNDTTSAALAPT
DPSFMFGHNTSFPMAGLPGPPGLNGSVHQYSASPHDDPSTASCSYVNWSANGPGGDDAF
FADFGLESPCEFFNGCYGCQEKIGQSAPNQATLASMLVSSDTKPQITYS*
>CnMYB68
MMARKCSYCGNNGHNSRTCSSPRSLVGAGGLRLFGVQLQVASPIKKSFSTECLSSSSSYP
ASSSSSPSSSSSLASIEENTDKTSNGYLSDGLMDRAQERKKGVPWTEEEHRTFLAGLEKL
GKGDWRGISRHFVTTRTPTQVASHAQKYFLRWNSLHKKKRRSSLFDVVARSVQYNDSSR
LRDQSSSELEIPTRTTCHKVGVNTTTRIRSSAQETQLPSSLNLMSICPVSMSTPPSMMSRS
SSSLMAKPQMQLQEPNLELTIAPTRPLNQTKASPKLCFHGDHQSYLTGWRQDGRFTS*

>CnMYB69
MQPRTGGHDILALTPADVASPFSLHLLLCSHVCLFSHLFAPVTTPSTPHPTSALPTPPAH
HRVSKKPSLSLFLYKYPSRAASPNPRRAQTKPIITKPLPTATPPSPSASLALPPNHLLSV
VVGMASSVSWTATEDKRFERALAIYDQDTPDRWEKVAAMVGGGKSAADIKRRYDLLIED
IQRIESGDVPFPNYRTTNRYANGF*
>CnMYB70
MGRHSCCYKQKLRKGLWSPEEDEKLLKHITKYGHGCWSSVPKLAGLQRCGKSCRLRWI
NYLRPDLKRGSFSQQEEDLIIELHAVLGNRWSQIAAQLPGRTDNEIKNLWNSCIKKKLKQR
GIDPNTHKPLSETQGREDKVPASGDKTAGSSEPKLLAPPSGNLNSVVPEFAPPAMSMVERS
NSENLVASTKDFFLYRFAASHESSSSCQPANSMDYLPLRNSTYSPSSSMDQNLPLWFNQN
SRLPDMNPELTCNMMMTAALPSGSRSTASASMGLKPTADNSSLPGFRYLEAGNSSNNSAS
SGNSTGGGGIELQSNNSIYESGIFQWSDLVSNRQAEIQLGGEPEDLKWSEYLNGTIPSST
DLQNQTQLLNGAVKEENQFPVDGLSSWLQPPEVFGKDFQRISTAFKQI*
>CnMYB71
MEDPDESRNGPSSWSWEDNKLFELALAVVDEGNPDRWEEVASIVGGKKSAEEVKRHYEV
LLEDLDIIESGRLDHAVGAPLSDTQDVRWTDEDQEWLAQLNIR*
>CnMYB72
MEVQGRVLGSATPNEEETDLRRGPWTVEEDLKLINYIAVHGEGRWNTLARCAGLKRTGK
SCRLRWLNYLRPDVRRGNITPEEQLLILELHSRWGNRWSKIAQHLPGRTDNEIKNYWRTR
VQKQAKQLQCDVNSKKFKDAMRYLWMPRLIERIQAASGSSFQASNILSDCPAGQPGQAC
PCQENSASAPAGSNSSDSVITQFSSPPTSDSFTGDYHNYFDCIQRGENNSGDGIQAGFAQLD
DGWYENRGLLDFEQGGWGENLWSLEDIWDMQQQV*
>CnMYB73
MRIMIKGGVWKNTEDEILKAAVMKYGKNQWARISSLLVRKSAKQCKARWYEWLDPSIK
KTEWTREEDEKLLHLAKLMPTQWRTIAPIVGRTPSQCLERYEKLLDAACAKDESYEPSDD
PRKLRPGEIDPNPESKPARPDPVDMDEDEKEMLSEARARLANTRGKKAKRKAREKQLEE
ARRLASLQKRRELKAAGIDTRHRKRKRKGIDYNAEIPFEKDLHQGKRRVDIEAQLRKQDT
ARNKIAQRQDAPAAILQANKLNDPEAVRKRSKLMLPAPQISDQELEEIAKMGYANDLVLE
NEELR*
>CnMYB74
MRIMIKGGVWKNTEDEILKAAVMKYGKNQWARISSLLVRKSAKQCKARWYEWLDPSIK
KTEWTREEDEKLLHLAKLMPTQWRTIAPIVGRTPSQCLERYEKLLDAACAKDESYEPSDD
PRKLRPGEIDPNPESKPARPDPVDMDEDEKEMLSEARARLANTRGKKAKRKAREKQLEE
ARRLASLQKRRELKAAGIDTRHRKRKRKGIDYNAEIPFEKRPPPGFYDVSGEERPVEQPKF
PTTIEELEGKRRVDIEAQLRKQDTARNKIAQRQDAPAAILQANKLNDPEAVRKRSKLMLPA
PQISDQELEEIAKMGYANDLVLENEVLGEGSGATHALLANYSQTPRQGMTPLRTPQRTPA
GKGDAIMMEAVNLARLRESQTPLLGGENPELHPSDFSGVTPRKREIQTPNPMATPLATPGA
VGLTPRIGMTPSRDAYSFGATAKGTPFRDELHINEDMEMQDSAKLELRRQAELKKTLRSG
LTNLPQPRNEYQIVIQPIAEENEETEEKIEEDMSDRIAREKAQDQARQEALLRKRSKVLQ
RELPRPPAASLELIRNSLIRGDEDKSSFVPHTLFEQADEIISKELLALLEHDNAKYPLDG
KQEKEKNKYAKRAANGKAMPVPEIEDFDEDELKEADSLIKEEIEFLRVAMGHENEAFDDF
VKARDACQEDLMYFPSRDSYGLASIAGNSEKLAALQNEFEIMKKRMDDEAKKATRLEQK
VKLLTQGYQTRAGKLWSQIEATCKQMDTAATELECFKALQNQEHLAASFRVSSLMEEVN
KQKVLEQSLQQRYGDLLTEHDRIQRLLEEHKVQLRIQEEIAAKNRALEEEIAARNRALEEE

AAAKHGTLNEKLAENNQHGDTAQLGDPCRPDSGLDETDLPNADHTETSSSGEQPTPSVPG
SQLYAEGSSERDDAFEGQSTQSANVIQEGLNDSEEHLSVVDKNAELSGGVAGFVNEEKQM
VGNMIDAPAPVQDQIGSLQEQEITVAHSGGQVAGASSGSAAEISACLSNQITVSYSAGLET
MVVDSGDTGIDGGLAGCSNISTQVVVPVESGNADPGLAEQIAGDSTNHGVATSAPRSEEK
IHSDFSIRTAVNSADCSPNNPDGGIVCETNVSIPDYRENSMVKLDDKAQWEMASDDTTQI
V*
>CnMYB75
MGRAPCCEKVGLKKGRWSAEEDELLVKYIKAHGEGSWRSLPKNAGLSRCGKSCRLRWV
NYLRTDLKRGNITQEEEETIIKLRTSLGNRWSVIAKHLPGRTDNEIKNYWNSHLSRKIHSFQ
NADGTRVIVALTKIGSGGKQDGSRTRRSATKISSTCWSKRKGDHERNCEHPISFVHGKEG
ENKVYNLDHDKSISIELVNLDEGVAFESGLIDFNEQLSPSIFVDSGLCTNNEIEGELFGA
SHDSGIMNLSEGRDAKDNIPNDDGEGVNLGSKEGRGGEVVGLDWEEMMRLEVDNFLN
WEGMWDGQREMWPCLWECDSGKSGCEEV*
>CnMYB76
MGRAPCCDKANVKKGPWSPEEDAKLKAYIEEHGTGGNWIALPQKIGLKRCGKSCRLRW
LNYLRPNIKHGGFSEQEDQIICSLYISIGSRWSIIAAQLPGRTDNDIKNYWNTRLKKKLLGK
RRDSPQSRRLSINQDTNEETNGISPDASSQALSTSALERMQLHMQLQGLYNPFSFYNNPA
LWPKFHPLGDKVSPIQQTDANTIAATPVQQASDATMIEPSGQNLLPIKPNMPNPMNAYIH
EDRNTSTLGFPSPSSSRTTLSMDNFNTNFNAAAVGLEDELHDLLYGKNECFGRQDKQQQT
AMECFKDMNGEKESMNWWATNGFDDKSSAGTWDFASNLQPDLLQEYGLGYDL*
>CnMYB77
MGRAPCCDKAIVKKGPWSPDEDAKLKAYIEEHGTGGNWIALPHKIGLNRCGKSCRLRWL
NYLRPNIKHGGFSEEEDHIICSLYISIGSRWSIIAAQLPGRTDNDVKNYWHTKLKKKLVGR
RRDRPHSCHLSSTDSKEPSNDIDHNKDLQSLSTSALERLQLHLQLQGLHHPFAFYNNPLL
WPNLLPPGERTLQSQHSNVSNETVVKPPSSTMSPIQPIPLDSMNGDITENFDGATLNGHC
STTSTTVFINEQSNSKLNAVEAELHDLLYGGKDCLKEMDGEAEGMNWWYLDDDGKSQM
SSLGSSYNLHDDLMVQQYVLPYD*
>CnMYB78
MAASMSNRSKDMDRIKGPWSPEEDEALQKLVQKHGPRNWSLISKSIPGRSGKSCRLRWC
NQLSPQVEHRPFTPEEDDTIIRAHRRFGNKWATIARLLSGRTDNAIKNHWNSTLKRKYSSA
AVAPAVDDAALVAADDAAVRPLKRSSSVGPVLSSGGGLCLSPGSPSGSDLSDSSHHMISP
VAAAAATSASHIYRPVPRAGGIVPPTSSTHQHHHTEATPAVSSTLQPNNDDPVTSLSLSL
PGSDKPEPSDHHRANQDQLQLLAPPAPLAQAMDLHPAPPPLAQALPLHQPPPPPTMASPS
AADERRRPSEEERRSAFPFSAEFLVVMQEMIRNEVRNYMSGLEQSGMMCVQPPQESIRNA
AIKRIGVTKIE*
>CnMYB79
MGRPPCCNKVGIKKGPWTPEEDVILVSYIQEHGPGNWRAVPTNTGLLRCSKSCRLRWTNY
LRPGIKRGNFTPLEEGIIIHLQALLGNRWAAIASYLPQRTDNDIKNHWNTHLKKKFKKYQ
TAIDSYMISSDSGPSCNGQTSKGYKMEGIRIPDLTASHSPPSRFYYKSPSTYASNTENIP
GLLEGWMRSTSPKSTPDEHQQSTNIDGNGNELLQGSQAKGDQWSCAVISKEDHDSLLSFE
NLSNIVWDKSSSSSVDSVLQGTQPAPADAEAKHGVADGRQQPPLSLLEKWLLDETSGQVD
GLVELPADCFLNPMF*
>CnMYB80
MGRSPCCEKAHTNKGAWTKEEDQRLIAYIKAHGEGCWRTLPKAAGLLRCGKSCRLRWIN

YLRPDLKRGNFTEEEDELIIKLHGMLGNKWSLIAGRLPGRTDNEIKNYWNTHIKRKLISRG
LDPQTHRPLSGGIQLINTSSRPQLLSMTTEAASKPADSTEEGQSGGISINEDGDRRLDLN
LDLSISLPHPSSKSSTSSEALMPPTAAEARTPTSVVVPPYTQTICLCYHLGFRRSESCSC
QTNSNYQHVLRYY*
>CnMYB81
MELAVATGGAEDTKGCTRGHWRPCEDEKLRRLVEEYGPQNWNLIAEKLQGRSGKSCRLR
WFNQLDPRINKRPFTEEEEERLLAAHRVHGNKWALIARLFPGRTDNAVKNHWHVIMARR
QRERSRIFTRRYCQNPLLDAFSNTNTGREGDSDMRPPTSDRIFGLSPLSSSPSMAFSGSTKS
SSPRDHSGAKRMDYYNHKHGFNILESFHSTNQCLFSYHCSSIYGSNQSFSAVGQVNKKPF
VYNPIGFTSVNDGCVGKDSNESEIIRVGDNMIQFVNIRANDAKEQEDESMRHKDVPFIDF
LGVGIAS*
>CnMYB82
MEAGDFSINREQGNRCWEIKLLVSALSGEIQELDSASHGGKKQWPSMTPEEEDLISRLHH
LLGDRWELIARRLPGRTAEEVESYWKMKEGGELEENKIYKPVHKHVVNSWLIYDIAGEIQ
ELDSASHGGKKQWPSMTPEEEDLISRLHHLLGDRWELIARRLPGRTAEEVESYWKMKEG
GELEENKIYKPTLLDSKRKYNMAIEIEKNNQTTHLQEILPTGIEIEEDGVLLSHGEIQELD
SASHGGKKQWPSMTPEEEDLISRLHHLLGDRWELIARRLPGRTAEEVESYWKMKEGGEL
EENKIYKPVCTRLSPSFKFTMDEPLEDTKPSQTGS*
>CnMYB83
MGRHSCCYKQKLRKGLWSPEEDEKLLKHITKYGHGCWSSVPKQAGLQRCGKSCRLRWI
NYLRPDLKRGTFSQQEEDLIIELHAVLGNRWSQIAAQLPGRTDNEIKNFWNSCIKKKLRQR
GIDPTTHKPLADTQAGDDKVPPNGGQNISGADDLIHPSSAALDPSSIPKPARSLMLTVDKF
AAESSNPKNSAAGAQEFLLDRFVASQESSSSCHPSNSLGFFPLPQLSYASDCGANQTPSV
GLSVAPNPLLWFNQSGRLFDSNPEFSCSGTISTLVPSASGSTLSTWMGLKSVIGLPPDNP
PPSCAALTGISNYWEAGNCSNSSGSSGNTGSGIELRSNGSYFDGGIFPWPDLTPDKDAPM
QLQGEPEDLKWSEYLQGAFSVSAAIQNQSQPLYGDVKTESRVAVDGLGTCYPNQQAQQQ
PQASDMYGKDFQRVSVAFEQI*
>CnMYB84
MDFNNMETVSAGRIDGLVEKSARSLRPAPPLTPLQRFLASSCHGELRLVKSPIDKLERKA
EIPLGVIGVPPDEMLLNDFSFYGDGKNAIIRSNNCGEGNGVKGSSSTTVAAASAVVKGQW
TVEEDRMLVGLVKQHGVRKWSQIAKKLVGRSGKQCRERWHNHLRPDIKKDTWTEEEER
LLVEAHKKVGNRWAEIAKHIPGRSENSIKNHWNATRRRQNAKRRSKRKTAQGGRCRPSIL
HEYVRSKMLDLRDDCATTKITPSAAPPSPPYQHYHITFPNVQEPSISTTTQMEDPMPFMEA
FLDRSATGSAEEGEFHVGDVQERPPGMLDPLDVLDFDDGDYLMAGVDEWQCMSLAPSP
SLDIFQSEANGPSTAVPDGTTSTKSAAPLFSDNYISYLLNGAPPSSADVLGSAANSEILEDQ
TSSSCKRELDLIEMLSLQSSRSRSRSRSLTQTSSSNGSL*
>CnMYB85
MLPFIRALVLVACLLPVLVDGRTRHYKFNVVTKNATRLCSTKPIVTVNGRFPGPTLYARE
GDNVLVKVVNHVKYNVTIHWHGVRQLRTGWADGPAYITQCPIRPGNSYVYNFTLTGQRG
TLFWHAHILWLRVSVHGAIVILPKLHVPYPFPAPHKEEVIILGEWWKSDTEAVINQALKSG
LAPNVSDAHVINGHPGPMSTCPTSKGFQLKVDSGKTYMLRIINAALNEELFFKVAGHQLT
VVEVDATYTKPFKTDTILITPGQTTNVLLTADRGAGRYLVTASPFMDSPIAVDNQTAMAT
VHYTNTVSTTVLTTTKPPPQNATPVASNFIDSLRSLNSKRYPARVPLRVDHSLLFTAGLG
VNPCPTCVNGSRVVADMNNVSFTMPTTAILQAHYFNISGVFTADFPGKPPIAFNYTGSGP

SNLGTMTGTRVYRLPFNSTVHLVLQDTGIIAPENHPIHLHGFNFFAVGRGLGNYNPKKSP
SKFNLVDPVERNTIGVPSGGWTAIRFRADNPGSKELEMVTNSWMQVLTPRAPCFPNSSWL
PGPKRTGNWTQEENKRFEDALAHFDGDTPDRWERVAAMIPGKSVRDIITHYRDLEDDVN
DIEAGRIPCPGYTSSSFTLDWENSHGYEGFKPSYCVGGKRSGGRPDQERKKGVPWTEEEH
KLFLLGLQKYGKGDWRNISRNFVITRTPTQVASHAQKYFIRLNSGGKDKRRSSIHDITTVN
LPNNGPPSPSQASVLAKRSSSAAATGISDQFSVMVDPNQPNEAAGVFSPSPHGTELKNIM
ASRYSLDVTLSSDNSLRVATNLQSTSNSLFIVCSNPQPKFYSNASATAFTVETAIAAVAA
TIRKNGAWYTLPQTHDLETVAKLFPVWRSLLAKRLQSIHFQQFWLKNERPSGYHHHQSEP
LKGMPSSDLSPKDSAQGTTSMATLQMRMVDA*
>CnMYB86
METEPETATATATDSAPPPPPPPRPSPLSSSSPPAPSAAVEVSTSKSPAAEGGAKDPGVG
AAGGSASDGVGGAGAAPADDRVKGPWSPEEDALLSNLVKKFGARNWSLIARGIPGRSGK
SCRLRWCNQLDPYVKRKPFTEEEDRIIIAAHAIHGNKWASIARLLEGRTDNAIKNHWNSTL
RRRRFLAGQYKAAACDAVQDASAEKAKESSEGSPSIGDVNSVRSLEGRDINSRENISDNS
ENMVHVGDGPSEVKDPPYLFRPVARVSAFRPYNHVSGPLLSGPVQMHGLIPRAFQAGGG
VCRLLDGNCSEPQVPPRCGHGCCSIQNIEHPRNSLLGPEFVEFVELPPMSNQELFSAASDL
SSIAWLKSGLQIGSSCIYRGSPGQIAPPRGHVQ*
>CnMYB87
MAAVKIEQSCVENKQSAAASSSSLSEGSYGFSRMSPAVSSLATSSPSHRRTSGPIRRAKG
GWTPQEDETLRKAVEAYKGRCWKKIAEFFPDRTEVQCLHRWQKVLNPELIKGPWTPEED
EKIITLVQKYGPTKWSVIAKSLPGRIGKQCRERWHNHLNPMIKKDAWTAEEELALMNAHR
IHGNKWAEIAKVLPGRTDNSIKNHWNSSLKKKLDFYLATGKLPPVPNPGVHGGSKVMVN
QASGQLFLSLNKGSDTSTEALSETAGSAHSCLPSESCKLEEHRESLEPITVQISPSEAFTGV
QIIGLNTSAAECQARTLQVDNLCNGSDPGVRLENCGNAGEIDKDDKIPGIALNSELSSTF
GSLCYKPPQLEDLGLSVASTLLSTYDPAQQSYSSGTVTSPIGYSTPPFVSGKSSGQHSAE
SVLKSAARSFPNTPSILRRRKREGSTPLPPDITVRTDRLKIQDSSCTPIEGKSRSGPEPL
KSSKSPCGDGGVVPYNSKTFNVSPPYRLRSKRTAIIKSVEKQLDFTSREDNIDGKAKSLG
LAINGSSHSADCNGNLPIMQGRKLSECPVGLESLANDLAHSTKLGVT*
>CnMYB88
MVTVRDETRKGPWTEQEDLQLVCFVGLFGERRWDFIAKVSGLNRTGKSCRLRWVNYLH
PGLKRGRMTPQEERLILELHSRWGNRWSRIARKLPGRTDNEIKNYWRTHMRKKAQELKR
NALSPSSSSSSLTDNSPSASEPPSEAGLEQHELNSGSGCGCIAGGFVENEEGVQGYSMDQI
WNEIATPEAVSELSFGEFKNEVFDTSWPAMPSPMWEYSSESLWKMDYEEFKIFPSVEDLLL
VSNYQHGREFCD*
>CnMYB89
MEGAGVGEEYRRVKGSWRPEEDAKLRELVEIYGPRNWSLISAGIPGRTGKSCRLRWCNQL
SPDIHHRPFTAAEDAIIVAAHARYGNRWAAIARLLPGRTDNAVKNHWNSTLRRRRLRGEK
PFSPADAAAADASGCGGGGGAGSSVVDESDSDSVRKRQCRRDDNGGLVEEVAAVGPATA
LSLRPPGEGGEERRSEVEASVVAVMRQMIAEEVRSYVDGLQSDGGGAVSFSIKSESASSH*
>CnMYB90
MGSLNPKSAVEMSFFPPPPPPQLPPSSSPPYLGASSPRSAERECWPCDMGFLSGEKQGEG
FQGFEGGQGSPGGEKSDGGEGDEGENAGGKRESEGGPAKLCIRGHWRPSEDARLRELVA
QYGPHNWNLIAEKLDGRSGKSCRLRWFNQLDPRINKTSFTEEEEERLLAAHRFHGNKWA
LIARFFPGRTDNAVKNHWHVMMARRQREMCNGYRRRKLSSLTPTNPCVPILPRMMELVG

TGNNNAHSGESTITTNRDESASSSTGLSPNSPNNMVIPGFVNRFGPSELPHHFQLLMGSHE
KLVAARNVCNEKFGTSTRCFYQSASMLMAMGVDQAGHSEATSEASTTESMANPRNNAI
MHAERDNDGEKMTFTFIDFLGVGAT*
>CnMYB91
MGRPPCCDKINIKKGPWTPEEDIILVSYIQEHGPGNWKSVPTNTGLMRCSKSCRLRWTNY
LRPGIKRGNFTPHEEGMIIHLQALLGNRWAAIASYLPQRTDNDIKNYWNTHLKKKIKKYQ
TTIGSYMTPSDSTITCHELISRNYNSETRNSDISTIHSLLPKSSHNLSTYASSTENISRL
LEGWMRSSPKADLKPAQDKQHQNIDAFKSSSSNAFNQAYFEVGQVKSDQECGHPIPQAHI
EGGQIMDDQEYCSRIPHEEVESFLSFENSSGISWEKSCVHSTFYRPQSMVADAEAKQRLD
SEHHPPLSLFEKWLLEEATGQADLMEVSADCCSDVMFQ*
>CnMYB92
MNRTTNDSDNIMVSKDQVDSPSNEGSSGGSLTGGGQVLKKGPWTSAEDAILVDYVKKHG
EGNWNAVQKHSGLSRCGKSCRLRWANHLRPNLKKGAFTPEEEQLIIELHARMGNKWAR
MAAQLPGRTDNEIKNYWNTRIKRLQRAGLPLYPPNLSYQASSENQQSQTATEYGNGEKRP
SELLQGNAFDLPDVIFDNLNAHQGPLSYAPPYPDISVSSMLSQGFGSQNYGFVNPMGNCM
KRFRESETLLPGFHGSVSSALPTLEQFSNEPSEKIQLSFGLGYPYDPDPSSKILAPFGGAVPG
SHALSNGNFSASRPLGGTVKMELPSLQYPETDLSSWFACPSPPPEAIDGYIQSPPATASV
QSECVSPRNSGLLDALLHEAQARKTQSLEKSSSSSVVAPGGMVESSELDLCEAEWEEYDD
PISPLGRPAASVFNECTPPVSGVSLDELQPSKEPSGPDIMLAAAEHVSPNMGERDISACP
DFLRPDALLGGSDWLENSETAKEHSTLNDAIATLLGEDLCSEFKEVPAGTSSALSQGLGL
DSCPWNNMPRACQMS*
>CnMYB93
MDAQESPLLITYSPEILDGEPILISSNCLPVKAFNFEPAGHAFHSAALKFFGFCEEEDTD
TDDQSVLSDDRGQAYIPSSDFYSSRGKKKSVAGSKQQDHYALLGLGHLRFLATEEQIRKS
YRETALKHHPDKQAALILAEETEGAKQAKKDEIETHFKAIQEAYEVLIDPVRRRIYDSTD
EFDDDIPTDCTPQDFFKVFGPAFMRNGRWSINQPIPSLGDENTPLNEVDSFYNFWYTFKS
WREFPHADDFDLEQAESRDHKRWMERQNAKLREKARKEEYARIRALVDNAYKRDPRILK
RKEEEKAEKRRKKEAKFLARKLQEEEAARAAEEERRLKEEEEKRAAEAALNQKKLKEKE
KKLLRKERTRLRTLSAPVVSKNLLDLAEEDVEKVCMALEMEQLRHLCDDLERKEGSERA
QLLKDALHGDNSNAMKQEAKNMQPNGSQDLGTKTNGVVAQVKLDSSLSNYEKKEKPW
EKEEIEMLRKGMQKYPKGTSRRWEVISDYIGTGRSVEEILKATKTILLQKPDSFKAFDSFLE
KRRPAQAISSPLTTRIESEGLPVEGTQDSSPKIAAKQSTNTTNNQKSVDGVVSALNGVSSG
AEQDAWSETQERALIQALKTFPKDINQRWERVAAAVPGKTMIQCRKKFALMKENFRSKK
NAEQ*
>CnMYB94
MASSNKIRSPQLNAEWSRSEDKIFELALVQYPEGTPDRWSLITMKLPGKTSYQVLHHYQV
LIYDLELIESGRVEIPDYKDNELEDEQEGDDYKDNELEGEQVGDNFSEERPPDGQKISSG
SSQRDEEAKV*
>CnMYB95
MASSNKIRSPQLNAEWSRSEDKIFELALVQYLEGTPDRWSLITMKLPGKTSYQVLHHYQV
LIYDLELIESGRVEIPDYKDNELEDEQEEELFLQACKSSYHDIIK*
>CnMYB96
MASSNKIRSPQLNAEWSRSEDKIFELALVQYPEGMPDRWSLITAKLPGKTSYQVLHHYQV
LIYDLELIESGRVEIPDYKDNELEDEQEGDDYNDNELEGEQVGDNFSEERPPDGQKISSG

SSQRDEEAKV*
>CnMYB97
MASSNKIRSPQLNAGWSRSEDKIFELALVQYPEGTPDRWSLITAKLPGKTSYQVLHHYQV
LIYDLELIESGRVEILDYKDNEPEDEQEGDDYKDNELEGEQVGDNFSEERPPDGQKISSG
SSQRDEEAKV*
>CnMYB98
MASSNKIRSPQLNAEWSRSEDKIFELALVQYPEGTPDRWSLITMKLPGKTSYQVLHHYQV
LIYDLELIESGRVEIPDYKDNELEDEQEGDDYKDNELEGEQVGDNFSEERPPDGQKICSG
SSQRDEEAKV*
>CnMYB99
MASSNKIRSPQLNAEWSRSEDKIFELALIQYPEGMPDRWSLITAKLPGKTSYQVLHHYQV
LIYDLELIESGRVEIPDYKDNELEDEQEGDDYNDNELEGEQVGDNFSEELQSDGQDLFDH
HK*
>CnMYB100
MGRAPCCEKVGLKKGRWTAEEDDILIKYIAANGEGSWRSLPKNAGLLRCGKSCRLRWIN
YLRADLKRGNISKEEEEIIIKLHATLGNRWSLIAGHLPGRTDNEIKNYWNSHLSRRVDSIQ
KFVGDGEVMVVDLSKLPGGGKRRGGRTSRPAMKKNTTNGITARREKNEKGVSPPTSSAQ
TQSNEGQSIVLDPDQNQTSCDTFEGPNQGVLGPNEDPDQNQASCITSEGPPNQGALGPNE
DMVSGLLSPSPMESKFWGGNSEMEAMLFGASEGSSGGWGSHDEERESGVMSSSEEREGG
TLVLHGDGGGGTIGSEEEEEEERGGGVIAQVEEEVGSAQADRFMDWELGGIEAKLWDDA
GEMWPWQWDDENRELGLQAIDGCGYQEELLDSWLLSDVL*
>CnMYB101
MEMGIGIGGEVHSSATPPSEEDMDLRRGPWTAEEDLILMNYIAAHGEGRWNSLARCAGL
KRTGKSCRLRWLNYLRPDVRRGNITPEEQLLILELHSRWGNRWSKIAQFLPGRTDNEIKNY
WRTRVQKHAKQLRCDVNSKQFKDIMRYQWMPRLVERIWAASGSSPATVHPNVSVPMAG
DHQPLEPGSESGQVKPSPESTSSAAGSSSDSVGMQFSSPPPVPDCFTDYYGGMQEGENEG
GDWIHDVQMGGWWLESLPSPGGYSNVGLPDFDQNAWGENLWGVEDIWLQQQF*
>CnMYB102
MAPEHLKTTAEMGFFHPPPPPPPFHGGLGTSSGGLECRSSGIGFPMEQKKNKGWGFRGFS
GEKGYHGDEKEDEGEEGEGGSHENDQPKLCARGHWRPAEDAKLKELVSQYGPQNWNLI
AEKLAGRSGKSCRLRWFNQLDPRINRRTFSEEEEERLLAAHRLYGNKWALIARLFPGRTD
NAVKNHWHVIMARKQREQSSSYRRRKTSTSSHNPPSSQAPPNKMEGHTSNNACSGESTIS
STRDESVSTCTDLSLSSFTSGIIPNFFTRYSPSQQPQPYEFLMGSDEKLVAGRNGCCEKFGD
HGGSFPHGAPMMLVPCFDQSGLSDSTTEALATESVANHRSRAWLRGETEHDKEKISLPFF
DFLGVGAT*
>CnMYB103
MLLRASTALSFRSSSSSASPAAALGVVAGSYPSSVLRFPSGRWDGSVVAMANRRPMTGVV
FEPFEELKQELALVPTMPDQSLARQKYSNDCEAALNEQINVEYNISYVYHAMFAYFDRDN
VALKGLAKFFKESSEEEREHAEKLMEYQNKRGGRVKLQSILMPLTEFDHSEKGDALYAME
LALSLEKLTNEKLLNLHSVASRSNDVQMVDFIENQFLGEQVESIKKISEYVAQLRRVGKG
HGRIYMKISSNHSVRSQRAEMGRPPCCDRLNVKRGPWTAEEDAKLLAYTSTHGTGNWTS
VPKKAGLKRCGKSCRLRWTNYLRPNLKHEGFTPQEEELIIALHATIGSRWSIIANQLPGRT
DNDVKNYWNTKLRKKLIQKGIDPVTHRPISEIIQSIGGLPTTTSTSRGKYHSLHGTRISC
FNRDLKNVLLSKSTSIIDPNFHESKPLTSNHFPQFYDVNSTTVATCAPSSGASSSSTVVT

AQVNAPSLSPEIKWSDFLIEDSSMGVSSSQVRYTFLTRSPAWKAKTDIESEQLMVESGVA
NQNDLVDEWTYGALDGEVSNVWGAPCDGSTSFVDAILDRDREMVFQFPEFVDDSYNIL*
>CnMYB104
MVVMEGGGVGRLDWEQRIKGSWSPEEDALLMRLVERHGPRNWTLISSGIPGRSGKSCRL
RWCNQLSPAVHHRPFTPAEDAAIVAAHAKYGNKWATIARLLPGRTDNAIKNHWNSTLRRG
RRAAASAAVPLPIRPPHAADSTDPDESDSESDAKRPCRRDEVSGSEVATVMAGLPAPPPAP
PPMSRIPADPVTSLTLCPPGEGPGGGGGRDRRDGRWDVEMREMCLVSIMREMIAEEVRSY
INSLRSEGGGATPIIVKSDPASNRQD*
>CnMYB105
MPPEPVPWDRKDFVFKERKHERGAGSDALGTGSSFSTFRWREPYHGPRDFPRASPRRPPS
GHYRQGGSSHQIYAEESGGHGCTPSRSDRFWLEDESFRPSPGRYGGGGGRSGSGGSRESR
GSFRRFPYWDSVDFSRQQHHDPNVTAQRSVAVPTPPASQPPLKDPNDKTGGGVDDGSGTG
RRFDQDHSLVSISWKPLKWSRPGSLSSTKIGRSESEETGLEVSLPPGKESPIRSPVTSPA
PSDEGAPRKKPRLGWGQGLAKYEKQKVEGSVDPSGTAAKVAVNDTSPKVVGLAGCPSPA
TPGSFTCSSSPAGIEEKPCIKAVNGENDTSYCSDSLEEFLSRLGYMEGNPINVLNTLLADL
LQPEDATSGDCTFSRQTAMNKLLLLKSGISKELEKTACELDLFENELKSMDGDAENDPCR
SSFTIPANSAPETCIESSDVPSKDSNPSKDHEFSSSACTGNTSLYTNSLNEHNTEIKDGD
VDNPQAASSRFNDSASSSGGVYGDDGEKLAGRFEIFEDRFKIPEVQHFILSDVERPALIC
DRGGGNCVEAGSSSENGNSGASLHGKTDCNLITLILSSNWDAAKKASQVFHKALPTDPPQ
FDIWESGKLLSRRENDLRIKEKLAIHKYLLKFKERVLALKFRALHHLWKEDLRLLSIRKH
RTKSSKRFELSSRASQGGSQKQRCSFRSRFALSAGNLTLVPTTEIVEFTSKLLSDSQIKL
YRNNLKMPALILDEERKQTKFITHNGLIEDPICFEKERAILNPWTQEEKEIFMELLATFG
KDFTKISSSLNHKTTADCIEFYYKNHKSESFKEVKKRLDLKKQRQCAPTSSYLVTSGKKW
NRGINAASLDMLGAASVVAAHSSGNAKNQQRYAGRSIHGTYNGLKVSCGNYRSLERVSS
VEIPGHERETVAADVLAGMCGALSSEAMSSCVTSSIDPVEKMNYMVVERPLTPEVAQNLD
EDTCSSEGCGDLDSFDWTDEEKSVFIQALSMYGKDFARISRCVGTRSRGQCKIFFSKARKC
LGLDVIHQGISNGGMLMGDANGGRGDTDDACAAEMDSAICSTQSCSKMDADVSRSVGK
INIEGCVHALMAPVQAETDRSSEQGVVGGIILEEEDAKVDEHVSVLPENKLVSEGDNPRSF
ITSKKNADAALRSNESVQLHEAVECVDAEMKIGEGIVSPVEPVLTACMEVESKSHIDDVVE
QKDNDGKFSADVLKKEVDVPFLMPETGSCNKQQMDADLTNGGKIFSASDSKADVNSLLP
GKKVVDCPRSTFAPDYQHQIPLDLLQCPGKNPQGISLKQENPHSVPLNSVLPDPFSVCFEG
PHHVASQATLNFEEHGNKQHQNPIARDLYQRYMMRNPSLNQVDQPLHILRGYPLQVINQE
VKREADPLIGEKQVLMDHTKRNGVSQSNQFFISEVHGYHCNHSRLSHPRPGVLFPLRIEA
QPEAQLRHCSQNACSEPEELMHRTSDVKLFGQIICHPSSSQKSNSSPHECNSKPSSPRIN
GSSTWKPSDVVKAGMPFASAPGNGSHVGLEELPARSYGLCDGNRVQTGFSSLPESAIMLA
KYHGSLAGMSFYSAKDGVPCSNGILAQQSYMQHLASDGKRLESFCELQKRNGIEMVSGF
QQQGRVARLGANMVGGGILGSGGVPDPVAALKMHYAARAKVLGSEMESWKGEI*
>CnMYB106
MAFHRNPTLRLLFYLCFPFFLVFRFFRCFSYPLGNSSSSSLHVRSMAAADGSGKKLRKPY
TITKSRDRWTAEEHERFLDALLLFGRDWKKIEDFVGTKTTIQIRSHAQKYFLKVQKIGLG
NHVPPPHPKRKVAHPHPQNSTDNSIIPLHASIPCPSASSSLIPAGAVWDGPSGVKNYSDA
TSLTNCYTVPKVEGDSGLMGKASIHNKNITWTGSSSRSWLTPEATKQKNPQSSYPVVPDF
GQVYTFLGGMFDPDITCPLEVYLEKLKEMDPITVKTVMILMRNLTFNLSSPDFEPLRRWL
STYDANTKTIGVITETAAPVQADRWLLAYDVNSN*

>CnMYB107
MVMVKEDTRKGPWTEQEDLQLVWFVGLFGERRWDYLAKVSGLNRTGKSCRLRWVNYL
HPGLKRGRMTPQEEHLVLELHAKWGNRWSRIARKLPGRTDNEIKNYWRTHMRKKAQER
KRSASSLSPSSSSSNLSSSSLSDPPLEEADVEKHERDGVMEGTSLMTGLEENGEGVKGYPM
DQIWNDIATSELIGDLSFEGYKDEAGDFSCPPVSSPMWDYCSDSLWKIDEGEFKTFAPMAD
NTIRES*
>CnMYB108
MGRTPCCDKANVKKGPWSPEEDVKLKAYIEEHGTGGNWIALPQKIGLKRCGKSCRLRWL
NYLRPDIKHGGFSEEEDQIICSLYISIGSRWSIIAAQLPGRTDNDVKNYWNTRLKKKLFEK
RRDSWQSRRLSNNMSPDASSRALSTSALERIQLHKQLQGLYEPFSFYNSPALWPKFQPLG
DKLFFHIQHPDANTIAATPVQQASDATMIEPSGQNVFNPMDASIQEDLNSSTLGFPSPSS
SHGALSMENSNSNFNAAAVGLQDELHDLLYQEIGCSGGHEQQQQTKLDCFKDLNGGKES
MNWWATDGFEKSSSGSWDSASNLQADLLQEYMG*
>CnMYB109
MSNRVKDMDRIKGPWSPEEDEALHKLVQKHGPRNWSLISKSIPGRSGKSCRLRWCNQLSP
QVEHRPFTAEEDETIIRAHRRFGNKWATIARLLSGRTDNAIKNHWNSTLKRKYSSAAVVP
AVDDVGLPHQQYDAALAAAEDAAVRPLKRSSSVGPVLSSGGGLCLSPGSPSGSDLSDSSR
YSHPMFSPAAAAAVSPSASRIYRPVPRTGGIVPPASSPHHHDATVVAASAINTKNDDPVT
SLSLSLPGSDKPETSDLHPTNQNQLQLLAAPLSQGQAMPLHPPPPMARPSTTDNQRRQPE
EERRPAFPFSAEFLAVMQEMIRNEVRSYMSGLEQSGMMCMQPPQESIHNAVIKRIGITKI
E*
>CnMYB110
MGRIPCCEKDNVKRGQWTPEEDNKLSSYIAQHGTRNWRLIPKNAGLQRCGKSCRLRWTN
YLRPDLKHGEFSESEEQTIVKLHSVVGNRWSLIAAQLPGRTDNDVKNHWNTKLKKKLSG
MGIDPVTHKPFSHLMAEIATTLRPPQVAHLAEAALGCFKDEMLHLLTKKRIDFVGPATPAIS
GDPRVAYNADANVNDDKEDTIQKIKMGLSRAIMQEPNNDKAWTMMGPTGEPSDGLAGM
CDMYPAAGDGFRYGGTAYGNDGEGSAWSQSLCTGSTCTGGAADQHGSGTHEKGEDDDG
EEAEGGKAARKGDTSMFGSDCVLWDLSDDLMNPIV*
>CnMYB111
MEFFSISGEAEARRAWTRVEDKAFEMALVAIPEGTPDRWSLIASHVPGRTPRELWEHYQV
LVQDLVMIERGEVEAPAEWDDSDDEEVARNSDGTRSSDHRAPRGPQMSFGRGRSPSLRRR
GTPWTEEEHRSFLEGLAKYGRGDWRNISRWTVKTRTSTQVASHAQKYFIRQSQNAGNRE
SKRKSIHDITNP*
>CnMYB112
MPFSIRSEAMDNRSRGKQPRTSGQGIRVSSTEWDELINLSEQEEDLVCRMYRLVGDRWTL
IADRIPGRKPEEIERFWMMKHKEGFAENRLRRAG*
>CnMYB113
MPFSIRSEAMDNRSRGKQPRTSGQGIRVSSTEWDELINLSEQEEDLVCRMYRLVGDRWTL
IAGRIPGRKPEEIERFWMMKHKEGFAENRLRRAG*
>CnMYB114
MDNRWRRKQPRTCGQEVSSTEWELVNLSEQEEDLVYRMYRLVGDRWTLIAGRIPGRKPE
EIERFWIMKHAEGFAEKRLRRAD*
>CnMYB115
MGRAPCCDKANVKRGPWSPEEDAVLRNYVEKYGTGGNWIALPQKAGLKRCGKSCRLR

WLNYLRPDIKHGGFTEEEDNVICTFYNKIGSRWSVIASHLPGRTDNDVKNYWNTKLKKK
MMAGSITSTSTPPPQPPVLPTIKIEDDGGGADFSNSLDTDYGFTYDSQKLYSEITQFSMTGL
MQQLDFSGTASISPPEDISATSSSITVDSNSGNGCINWSTNGVGADDAFLFGDCFGGYGFQ
EGMGVVSPYYYSNLNSM*
>CnMYB116
MGRKCSHCGNNGHNSRTCSSPRGAAGGGGLRLFGVQLHITSSPMKKSFSMECLASSSYL
AASSASSSSTSSSSLVSIDETTKRISNGYLSDGLLGRTPERKKGVPWTEEEHRSFLAGLEN
LGKGDWRGISRNFVTTRTPTQVASHAQKYFLRQNSLNKKNRRSSLFDVVASCDKAAQVT
DSSKFKEPSISNELHVPALTIGYGASQITAIDLNSSEQETQLPSSLTLMPNSSACPLPQSS
LVERPCVHLSPLNLELSISSTKPLDQNNSSPDSLFAETIGVT*
>CnMYB117
MASPWTPKQNKLFETALAKYDKDTPDRWHNVARAVGGKSAEEVKRHYEKLVEDVRQIE
SGRVPFPSYRTSGGRGTLLRYLRLH*
>CnMYB118
MVEEANSMDKKINSSEEAEVRKGPWTMEEDLILINYIANHGEGVWNNLARSAGLNRTGK
SCRLRWLNYLRPDVRRGNITPEEQVLIMELHARWGNRWSKIARQLPGRTDNEIKNYWRT
RIQKKVNNSESSDHQHPMHIDEGSTSTSQTNSVEDGGSQPSYTEQQETNPDALAPSFSTES
SDNFWSVEDFWSMQSLNGD*
>CnMYB119
MGRRSCCPKQKLRKGLWSPEEDEKLYNHIIRYGIGCWSSVPKLAGLQRCGKSCRLRWINY
LRPDLKRGSFSQQEEDLIIGLHEILGNRWSQIASHLPGRTDNEIKNFWNSCLKKKLRHKG
IDPSTHKPLSEMEAPVEKPGLNNSSSSGSTEHQPVKPVFDPFPLIDIQVGVDPVETSSNL
YNQIQQAFRPPNQNELLNSSGFCDYGSVLDVSENYGYGESSSNSSNWNCNVGADVNNVL
GTEVLNWASEKKVDSLTQIQTNGVETFEEKISPWLENQHHAQSTQDYNGYLIRSLSRDPSE
ACFDIPRGELANEFNVDFF*
>CnMYB120
MADLDQSSEDVSVDSREANSQDSKVEFTEDEETLIARMYNLLGDRWSLIAGRIPGRTAEE
IEKYWTSR*
>CnMYB121
MKSEGGAEESSAAAALKKGPWTAAEDAILMEYVKKHGEGNWNAVQKNSGLNRCGKSC
RLRWANHLRPNLKKGPFTREEELLILHLHAQLGNKWARMAAQLPGRTDNEIKNYWNTRI
KRRKRAGMPLYPPEFQRQVLLRHGYHLRPHAASSSASPAPPAPLPASVPRARLRPPPLSLM
HPISFPSSPSPTAAFSPSSSATAPIPLVAQNQFSNGLPSQFPLSSTVLPPASPLFQSQQQQRL
QFSVSCPVSPTPPSSLYQSQQPTHCNLGSFDVNPSPSSTQMPFRGELPSSQILGETGGGC
GGGAIGLLRSSSTGLLDDMIRELQRDVEIPRVDLQPQPPPAAGAQGGDGHPGGNDVNKDA
TFSPVRGSVQESLSKLDGSDSSINGIEMRSKASNEISPVHDDISMLLDAIPAPTMPATMV
APDWYNSDSGEISNGPSSVMTDDDIGLEMHQLASSLSIAPVD*
>CnMYB122
MGHHCCSKQKVKRGLWSPEEDEKLIKYITTHGHGCWSSVPKQAGLQRCGKSCRLRWINY
LRPDLKRGSFSAQEERIIIDVHRILGNRWAQIAKHLPGRTDNEVKNFWNSCIKKKLIAQGL
DPKTHNLIPAARPNNSINSANIPQFHHPQSTTTPFTISSPNKSFETTTPMDMNPPLVTLP
PPPTLPLHETIPISTFQYQDANVLMSFKDQNSHASIDFTHASSSLDQTNISCSSFHQPAG
FMDDCMWASTVEPLEAPRQNEVEFQQGQSQAQHLACKMDFNGPNEEKGLEMESSYNSA
TFDIELMESALMPPGVFCGGSSIEQLQWDC*

>CnMYB123
MLSGAKPVAIDDTDSDSDEESSSTDDIDLLRRLQERFYVPSSDADSLPFIKPLSSRPPPD
SDEEDDFETLWAIQKRFTQYESDAVGRNFQNSIQDPEIVVTDMTSEPDTHNILPQNGKSM
VPSEELLASGFTEHAEDGARNVNIGGFMKSRFPKSAKFFVDALKKNRSCQKFIRRKLIEI
EVKIETNKELKERLKCLMDFQVACKRKAGHILSQKKDPRVRLISMRKPGSITDSKVNILT
FMLLTLTPFAFFKSFFKICLGPAENSHVSKYKMVLKTFPVSLRKQKWSNMEKEKLAKGIK
QQYQEMLILNSMNFESDIDSNLISALSSSDLDITPEKIRSFIPLVNWDQLASMYLTGRSG
AQCEARYELGRIFSFSIHLPFKWLNCEDPMINHVPWTIVEDKKLLFIVQERGVYNWIDIS
IVLGSNRTPFQCLARYQRSLNPHILNKDWTEDEDAKLHAAVESFGDNNWQLVASNLEGRT
GPQCSNRWRKSLNPDRRKVGRWSVDEDKRLKVAVMLFGAKNWKKIAQFAPGRTQVQCR
ERWLNRLDPSLNLKAWTEEEDAKLLAAIAEHGYCWSKVATCVPPRTDSQCRRRWKVLLP
HEVPLLQAARKMKRTVLISNFVDRESERPAIGPNDFTPLVNLSETENNDSTRPGKKRSSDN
QPKKSRVKSRRSFKENSIADGVINSSTNAVPADSAIVCSINSDTADGGSSKRSRKKKSSNTT
AESGRIKRTRKKTPRLIDSQTFLSNTTAESGRIKRTRKKTPSDDQPNNSMVKSRGYCKEN
IMADGMVDSSTETAPADLSLVPSASSNAAENGSIEGTGKRTSSNNQLNQSRGKSRGVPAG
LSAVPNVNFCTAECRGNKSSAQRTLSRSHEENKTTVDMVINTYAETIAGDASFIPVINSN
NAKSGNIKSMRKKTLSSPVLRLGSPAVGIRHAKRVRNQTLSKNSRTSYPCPRARRERSLE
ELPLTDSIIKGNIISKEKKGLANLILEKPIFSEKRECLDNQVVVQNAGDAFENNGIDKMT
IKKWLEVRHGMDENTQSADCHKALELLKSSASETADSRGIVVDGDQSTEPC*
>CnMYB124
MKLIAYIQKHGHGNWHALPKRAGLLRCGKSCRLRWINYLRPDIKRGNFTIEEEATIIRLQ
KSIGNKWSKIASCLPGRTDNEIKNVWNTHLKKRLARKESSISSSNCNISYVNDGNLKVDH
EDGEQKDFGSSADLPKNNSFEVECSQDSVNLMTEIFMELDRCNMPIGHDPSLDDGREFSS
ISSSGSHITSCDFQPPHEDGKNYRDYLIQVPEIPIDPEVWSMVGDDSCFLSPGMGPVMGD
GAHNDAPNSIGDSTGDDANKGWLGCLEKEIDDLWGTVDVNAVK*
>CnMYB125
MSPSLQNEWSWNENKIFEVALTEYPEETLNRWSLITAKLPGKNLRQVLDHYRALIHDLEL
IESGKVETPNYKNDEEEEDNSALIHDLKLIESEKMETPNYRNDEEEEDNSNDGSSIKPEQ
QQPTEPEQQQPTVQSGRHREEKRKSVPWTEEEHRSFLKGLAAYGRGDWKNISRNSVVTK
TSIQVASHAQKFFIRQEQNKESKRKSIHDITK*
>CnMYB126
MGRAPCCDKANVRKGPWSPEEDAKLKSYMEQHGTGGNWIALPQKIGLKRCGKSCRLRW
LNYLRPNIKHGGFSEEEDNIICSLYVAIGSRWSIIAAQLPGRTDNDIKNYWNTKLKKKLLGK
QHKNQQQARRVCRRNQGTKESEDGSTMSAKEGNYHSYCPMPTNLTNQFNQVGHHASDD
HESIKKFLMNLDGAFCCHGRAEQGNPLASSLGRHHLNRNSMGSITLPEQANSFMNGYSPS
GYRISQGLNEIAVELDEMFLGNSVKLQGLDCFFGAGNMVAENNGTSTSGSMNWNDISPLI
YPPVVTSYQGMQQQCLPDELVHILGAQI*
>CnMYB127
MRKPDPPAKNNMSNNNSSNGATKLKKGLWSPEEDDKLISYMLSNGQGCWSDVARNAGL
QRCGKSCRLRWINYLRPDLKRGAFSPQEEEVIIHLHSILGNRWSQIAARLPGRTDNEIKNF
WNSTIKKRLKNSSSPSPNHSGSPEPKVGMGGLTTLNDHEMKTMCMDSASSSSSSTMQGLS
ISNQYDLLPLPEVSCVMTALGNSYFHEPTSFPQVGFGSGNYGSHGMMMGGGLMGGGCEL
FVPSLESTSAEENGTLQNTTRNISDPCHSHNNDVNKINSDTAVGNEIYWEGGNIRMEWDL
DDLMKDVSSFPFLDFQAE*

>CnMYB128
MGRSPNENGLKKGPWTPEEDQKLVQYIQNNGHGSWRALPRLAGLNRCGKSCRLRWTNY
LKPDIKRGKFSQEEEQTILHLHSILGNKWSAIATHLPGRTDNEIKNFWNTHLKKKLIQMGID
PMTHRPRTDFFATLPQLIALANLRELIDGHPWDDHTARLQAEAVQASRLQYFQHLLQSAA
AMASNSSTNSLSAITTTNMDPMSLLSPPQMLSFASVPTTSPIESIGCQSQISQLPDLQIP
CSSSDQPMSNDTIQGSDFSLFIGQGDPSNTPRTPLISLHSSLPPLTDISASNPGDACSTS
SCCGSGSPSSFWPELLLDDPFMSELA*
>CnMYB129
MGRPPYCDKGGVKKGPWTPEEDLILVSYIQEHGPGNWKAVPTNTGLMRCSKSCRLRWTN
YLRPGIKRGSFTEPEEKLIIHLQALLGNRWAAIASYLPERTDNDIKNYWNTHLKKKLKKLQ
SSNDSSMSRGLSSNQSISKGQWERRLQTDIHMAKQALREALSLGETNLLTELMKPSNGSY
SSTRPSQPATSSSATTYASSTENISRLLEGWMKNTPKPRAAQANSESTQHSISKAVGTDS
SSSEEETASVANNGRTAVPNLFESLLNFDSSTPELSETSLFQCESRPSSEAQVPLSLLET
WLLDEGFGQGEMELIDMPLGEF*
>CnMYB130
MSLQQCGGSEGMETRCGFRNYSSSPSSSKGVFADISSLSINPTTSAGDHPCTKSSEKTSW
TFPFMQKSEVGHKCSDGSDGSGEKEPSAIGEESPDGENTTNEGSLGGKETVDCGQSKLCA
RGHWRPAEDSKLRELVAIYGPQNWNLIAEKLEGRSGKSCRLRWFNQLDPRINRRAFSEEE
EEKLMAAHRLYGNKWAMIARLFPGRTDNAVKNHWHVIMARKYREQSNAYRRRKLSQAV
QRRLEEGATTTTTAAVCAFGRCPSPSFTFPAAIHGSIGSHHMTLGVGDSLSSHNGFYTQKTP
FDFFSGSRGHDNANFNGNSSWDWPRDDSFMGFYHHPPFMLAMQQPSHASHHSDSTASA
ASRVSALESSSSEENKEGSHFEATVSPTFIDFLGVGAT*
>CnMYB131
MGRSPCCEKVGLKKGPWTPEEDQKLLAYIEEHGHGSWRALPAKAGLQRCGKSCRLRWT
NYLRPDIKRGKFSLQEEQTIIQLHALLGNRWSAIATHLPKRTDNEIKNYWNTHLKKRLAK
MGIDPVTHKPKSDALSSGDGQAKSSANLSHMAQWESARLEAEARLVRESKLRAAAQLQP
QSPSSSAPPLPPALQGNKSVAPPAPPTPSLDVLRAWHGFWSNKPTTGNPGGRIDLESPTSTL
SFSENVLPITGGGLLDGNSTACRQGAAQELEEEQDLEWKCFEKQPLRMLEIKDHRIDSFAS
FSMDTTSVLVGESSWLPESSSIGTGSSGGEFGARFTGMLLDSSDDRNSSDGRGDSDNFEG
GSCVEEEGKEGEENKNYWNSILKLVNSTSPSSSPPVF*
>CnMYB132
MEMSGWLGFSLSSSSKGDVSCCGDEVFGGGADGGGGGGEVGFGHHPLAGMPLRSDGSI
CFMGPPFRPPPPAPHGAPDWRFGGAMATTPASSNREGKGPKLEDFLGGYSENASEENQSQ
QPISNFHDMYYQSSGGPGINVNMPPSFSPVEGGAKEDIRNSCRFFQTCHQSNHSFQEIPTLK
PQFLVPNADPNPNPGPNSMYGGGMDGSTSISGIKSWLRQNQYVPGKQLAESSECNFQSLT
LSMSPVMQSGSSEMVPVGSSAAADGMKYPNAKSTTREPIPRKSIETFGQRTSQYRGVTRH
RWTGRFEAHLWDNSCRKEGQTRKGRQGGYDKEEKAARAYDLAALKYWGPTTHINFPLS
TYEKELEEMKHMTRQEFVAHLRRKSSGFSRGASVYRGVTRHHQHGRWQARIGRVAGNK
DLYLGTFSTQEEAAEAYDIAAIKFRGVNAVTNFDISKYDVKRICSSSHLITGDLAKRSPRDT
PSPSEPTEADHRPTLAITNSGSVDVDDLSPMMWNSYNRSDPSSNQPSNGGVVPFVSSSVSN
PNALDNQAERPRNSSSNEGACGDFPQAFYFTHGMKWSLIAGRLPGRTDNEIKNYWNTKL
GKEVKTIPEQLNEERDHGKLKQRKSKASLRTEAPKELGEQSHPVIRTKAVRCTKVYIPQHQ
DDRMAGQNLEPRSNEQALESMLEDVDPADFFVDFDMQDLLLEVPADDVSRVCRNEPRED
SNGVHGREGSNRMLSPRSGLSCHFHGAMLEEWRDGDGLQSKLASELNALASLLDLEE*

>CnMYB133
MGRKPCCSKKGLKTGAWTAQEDKILVSYIETHGEGKWRSLPNRAGLNRCGKSCRLRWLN
YLRPDIKRGNISEEEEDLIIRLHKLLGNRWSLIAGRLPGRTDNEIKNYWNTTLRRRVHGQP
SLDSKPASTAKSKGKTPMTGPPVAHLPPPTDTPVIRTKAVRCTWSFRAPDRLMSSPPNGD
QRLDLVQRETRTLFEVPQHEPLNEPGMGFRDSDKACPLDLDLFQLQDIGEGIRVGDNGMS
EDGSSTNFDMYSNDLVPFDGTMLESWLANECYQPEVTLDLQPLASLLDSEEFWF*
>CnMYB134
MCTRGHWRPEEDERLRELVARYGPHNWNAIAEKLQGRSGKSCRLRWFNQLDPRINRSPF
TPEEEERLLASHQIHGNRWAIIARLFPGRTDNAVKNHWHVIMARRCRERTRLYSKRPYRSA
IKDEKSRNGKERKSEPRNIVALIEKYYGGYSSDSAANNLQDHKEFYSEIHGYHPHMVADG
DRERENSIEFYDFLQVNTDSNDTKCCSSIEEQEETKENCSKAGVPFIDFLAVGSSS*
>CnMYB135
MASGSLSRSTGSSWTPKQNKLFERALAVYDKDTPDRWQNVARAVGGKSAEEVRRHYEVL
VEDLKHIESGQVPFPNYNIRPSGSDEEQRLRNLKLN*
>CnMYB136
MRFQEKHQFEAAEEGTIWTIAGIPLPAPLRPIKIKPSQNDAEKEEEADMLDMTPRQKEAK
IPERLSCPPAPKKPKPSLRCNPNGVEFFSPPDLESVFIRRVEKAKDEGEEDAIEVVVVEV
EVVTVEFKAMELVRPGLERCGKSCRLRWINYLRPDLKRGNFTQEEEDLIIGLHEVLGNRW
SQIAAQLPGRTDNEIKNLWNSSLKRKLKQRGIDPTTHKPLSETGAEEERPKPCVKPIFNP
FASFEYQAAFDPVETNANFFHQFQQTCRPPEQNEFMVNFDDGFTSRPNSVYCDYGEALDI
SDGCGIQESLNDGSNNWNCNIAAQMSSVLGNEILNWCSSDIKLESPIRTELETHEKKLNP
CPGLQHVESSEEFSSYLTASRSQDLSDACFDISQGEPACELKANFLTS*
>CnMYB137
MDFGWFKTHTGNGLPLEPCVSPDELQDFQHFGDAFDHVPTVADAYPSGFDLKALSSPPVG
LQIGMPVEDFQSMAGLSSGYPQRSLSGRMGPDPSLSSMVLSAEPLMTFGPPDELSFVAGD
NKSCSGGVEMNRKCTPVKKGSGAKIHKKPNVVKGQWTLEEDRLLVKLVGQYGLRKWSH
IAQMLRGRIGKQCRERWHNHLRPNIKKDTWSEEEDRILIQAHSEVGNKWAEIAKRLPGRT
ENSIKNHWNATKRRQFSRRRCRNSKYPKSGSLLQNYIKSLALQGDSAPTIPAASVAATNAP
ADKHLNIIESNDGGSEASMTLTCISAEGPPIVVGADVCGGSDDILTCDFSDMANLLLDDTK
VDVPPERYMGYLFDQLGCSPGMKSCFDIEMAWDEMANPMPMSLPMPCEVDLKVKVKKE
MDLVEMIAQNNNADSTSNSSSSSCNQF*
>CnMYB138
MVKKTERGGKKGVVMVNKGAWTAEEDQKLVDYIRAHGDKKWRTLPAKAGLNRCGKSC
RLRWLNYLRPGIKRGNISEDEEDLIIRLHNLLGNRWSLIAGRLPGRTDNEIKNHWNTHLSK
KPLTIDDLNLKQDTKGHSHPSITPPEPMQGFKPTVSEEHFVAWLPENNDGSWDDLSEFDVE
QLFDFTSMPGVEGNEGGSSSSELGIEDYGMTHQDGCQELIKNDLCRENEASCFSRIEAQEQ
WCFSKTNGAWQLKVGDCR*
>CnMYB139
MGCRTCEKPRVNYRKGLWSPEEDQSLRDYILEHGHGCWSTVAAKAGLQRNGKSCRLRWI
NYLRPGLKRGIFSAEEEETVIKLHAMLGNKWSQIAMHLPGRTDNEVKNYWNSYLKKKVA
KIEGSNSYASGTKSQDTANQSPKMNQILEEVGKQMLGSNFLELPDSSSVDLRPSARCIISHD
SFKGTQHPPLPKVLFADWLSMDHATGQSLPNSNGGMNCQWDFNLHADVSNYGSFQVDG
PSSGDFLRGFGDSSIYGGFQLQFGPGDQIQGGTCYNDLSMDETGGSLDTDHDIIY*
>CnMYB140

MGRSPCCDKANVKKGPWSPEEDSKLKEFIEKHGTAGNWISLPHKAGLKRCGKSCRLRWL
NYLRPNIKHGEFSDDEDRIICSLFASIGSRWSIIAAQLPGRTDNDIKNYWNTRLKKKLLGI
VPSQRKPHQEQQQRNQQQQLFPSPSSSSQGNRYTSPVPPLPVLESFPKISQDAFTIVPTI
TSSTLFQGPSYQYQMKESGNNTIMFGSEHSCSSSDGSSTQISYGKMDHNYICRGGGGEQM
GFESSLCGGVIEDYGPSRLWEEAPLEYNYEEIKQLLRNNNDHFFY*

>CnMYB141

MEGQFGWGTLENGWRKGPWTAQEDEILIEHVNQHGEGRWNCVSKLTGLRRNGKSCRLR
WVNYLRPDLKRGKITPHEENIILELHALWGNRWSTIARSLPGRTDNEIKNYWRTHFKKRK
PSKNIERARARFLKQQKEQQKGEEEQQQQADMRGIMTQVEEATLAQDMQEMKYYMCPL
NYMLQEDGSVVGGGSMSDGSSEEEMWGSLWNLDDIHDDREGVRRGDFTMQDQELAFY
*

>CnMYB142

MGRAPCCSKVGLHKGPWTEREDALLTNYIQCHGEGNWRHLPQKAGLLRCGKSCRLRW
MNYLRPNIKRGNIRPEEEDLIIRLHSLLGNRWSLIAGRLPGRTDNEIKNYWNSHLSKRLRQ
HGIEVREGTSKGTSKRARGTQNKNEETNNKSGSHNRKNQQDRRKKNNGVEGTGEITRTK
IYAPKPTRFTPTLRNHKAMESSSGSDIPQIENTTSEEEKGDIVLEDLEVKWFDINEGGFFFEE
DDPNHVLALDFPSKDRLLERLYQEYSELLNPEGYQVPLNSFPQTFLF*

>CnMYB143

MTRRCSHCYHNGHNSRTCPNRGGVKLFGVRLTDGSIRKSASVGNLSHLATSSASPPDGPD
PGAAASAAAEGYASEDFVQGSSSSCRDRKKGVPWTKDEHKMFLLGLQKLGKGDWRGIA
RNYVVSRTPTQVASHAQKYFVRQTNLTRRKRRSSLFDMVPDEPVEPQPTPMNCQESEMQ
CSKPLPEPLAMDEESKSNHSNNPVVGEAADPKPETLQCSYPVIPPAYFSPFFQFYIPCWPGY
QTDALQRQGHEIVKPTAMHSRTPIDIDELVGMSKLSIEEPMGKRAPLSPSLNLLGGSTRQS
AFHANPPTRTQA*

>CnMYB144

MQGSGGGSRCSATSSNRISSSGKTNGRAEGGGGMVMRKGPWMAEEDEILIEYVRKHGPR
DWSSIRSKGLLPRTGKSCRLRWVNKLKPDLKTGCKFSPEEERIVIDLQARFGNKWARIATY
LPGRTDNDVKNFWSTRQKRLARILRTPLPSRSSKNHGKAPISSHEVAHLEGPSLDLILLE
GDSSNGQCSRDASKMAQTPDIMNSAPLNLDTALPLLEPAMEKEPRLSNNPPPPLLSFDHL
PHPLIDLPLLQEDASELLPGYRDASFPDEFACLEPCQCMPPPAGLPFFGLDAEQNGITKV
DRYPGTPDFFEDLPSDMFDYLEPPPPPPPPPPASSTSW*

>CnMYB145

MVRSPCCDEVGVKKGPWTPEEDTKLVEYIQKHGHGSWRNLPKNAGLNRCGKSCRLRWT
NYLRPGIKRGKFSEDEERLVIHLHSVLGNKWSTIATQLPGRTDNEIKNHWNTHLRKKLLL
MGIDPATHRPRTDLDLLASLPNLLAAAKNLGNLTSPLDNVLRLQADAANLARFQILQSLLQ
LVGSNSPPYMDSRSLLGSASLQNYNFSDLLQMNRQLEGLTNGLLRLPQNQIQTTSGLSDL
GNNPQLPDNFQAYPESSLPLKEQEMVAQIHGSCSTTQHAMDGNGESTNPVLFNSSAIPTPH
STPSLVSASPENTSTDQMRDPINSNASTPLEDWEGLDLGGLDGDTSWKDILDQISWSNAS
*

>CnMYB146

MGRGRAPCCEKVGLNRGSWTPEEDIRLMSYIQKYGHGNWRALPKKAGLLRCGKSCRLR
WINYLRPDIKRGNFTKEEEETIIKLHGLLGNKWSKIASCLPGRTDNEIKNVWNTHLKKRLS
SGDQSRKTDKLEETPSSPSSTTSNSSSDQGEGKSDSEHTNPSVDSTNSSEDKIEIPIEPSM
DMWLMMLEDALPSSPQKTDTEDKNMWELQLEPNSLKVSPCSSPSHSLAMNGNTGGGGG

GVQALNDTDMRETEKDPLEIPDVPIEPELWDMIKDGDAGLFSPGVGAMEELGVHGNPTG
LHEESSREEGSIIWLEYLEKELGLWGASDDNPECPMSPWTEMEGDPVSCYFQKGPSSTSPL
DLHDLKVS*
>CnMYB147
MGRSPCCEKAHTNKGAWTKEEDDRLIAYIRAHGEGCWRSLPKAAGLLRCGKSCRLRWIN
YLRPDLKRGNFTEEEDELIIKLHSLLGNKWSLIAGRLPGRTDNEIKNYWNTYIRRKLLNRG
IDPATHRPIHAPLSDCTVTFAERAKKDVGILQREEEKSNSSSSEESSSWQQYKQPDLNLE
LRHCSYCMSLGLQNSQECKCNGFLGLRTGILDYRTVEMK*
>CnMYB148
MPAKWLLCVLDEELNSHSFSTYGRVGTMGRSPCCDEVGLKKGPWTPEEDKKLMEFIHKH
GHESWRRLPKLAGLNRCGKSCRLRWTNYLRPDIKRGKFSQEEEKLIIDLHSVLGNKWSA
MAARLPGRTDNEIKNYWNTHLRKKLLQMGIDPVTHRPRTDLNLLGGLHDLLPAANFGNL
SHLDHALKLQADAALLARLQLVQNLIQVLTNSPTSNLDLMGLLGSASLGNHQLSYLLQLS
RQYEGLINSPLSPHGSNIQLPSSLPNLGLQNLLNNIQAYSKSSLSLDGGEVAAHLDGVSANS
YSLPTSSSAPSLAPASPEDTHIDQMQGRATSRNVSSTTAPFAPWEDLNLDDISSDFGWKDF
LE*
>CnMYB149
MGRQPCCDKLGVKKGPWTAEEDKKLISFILTNGHCCWRAVPKLAGLLRCGKSCRLRWTN
YLRPDLKRGLLTEAEEQLVIDLHARLGNRWSKIAAKLPGRTDNEIKNHWNTHIKKKLLKM
GIDPVTHEPINRQPSPKSSQSTAATESKSDDHQLQSQGYEEGQNHSSENLSPPEESSNTSS
NDLLVSSLWEEYIPLIDESWTFPCNEEDYSSVVAPLPWEGSSEWLLDYQDFGIGDMGLGS
SAIEN*
>CnMYB150
MEGGRIRQDSRSEKGLKLFGVTILGDGCGGKEAEDVMRKEAAKEVMRKCKSMGNLAAC
GSAPAAESGAGDNGYLSDGGLVQSSTRRGGRGRKRGMPWTEEEHRTFLSGLKMLGKGD
WRGISRNFVTTRTPTQVASHAQKYFLRQTNPTKKKRRSSLFDVVISDPLQAPESETSALPLS
SKKSSEVTEKVDHHLQDSYLQVRISGNSKDQVVPALFLAPPLDISNQTFTSCQASLTACVP
DFLKVSLSPSIQLRPHYPTQFTAASPPQENSNLELSIAPPQQHSLTKLSSPKAAETISVI*
>CnMYB151
MGHHSCCNQQKVKRGLWSPEEDEKLIRYITTHGYGCWSEVPEKAGLQRCGKSCRLRWIN
YLRPDIRRGRFTPEEEKLIISLHSIVGNRWAHIASHLPGRTDNEIKNYWNSWIKKKIRKPT
TPPSTSPSSADLVQPGFNSIDQLDAIINQNLSTKPAPDNIFSMHCPIFMFDTNAGDTRPG
SSAREELVQDVATLNSDMWNPQNQDQALPPLLDFSSSMDTTYLPSLVDGMGNMAPMEA
QPCCVGDDGETSRECFEKQELNDWMDSQQYSSLLIWDQVQGTLGAEGLSTATTSTDSMV
TSFPSSL*
>CnMYB152
MLIILAALFVGLLFAVTLTSAIRFLFCAASRRHPSLADPEKQVAPPSGAEAEGTTLVFSA
ATRLARAEAECAICLAEFVEGDGVKLTLQDECLICLNFSSTNDVLSLLFYCRLSFSPHRL
FPPCSPDLLTLSLLVPMMKEVDKMSDELSEKGSYCLRPLTPLERFLFGERELHVSKKPTP
REANKVVFPSDANAHCGDPLGAIGVLPTGVLFDESGFCEGFVGCSDKEVGEGLKCTEEK
GRTGISSSSRSYAADIKLVKGQWTAEEDSLLIRLVEQHGVGKWSQIAKKLVGRVGKQCRER
WHNHLRPDIKKNPWSEEEERLLVEAHKKVGNRWAEIAKQIPGRTENSIKNHWNATKRRQ
NSKRKTKKNASQAERPQPSILFEYIRSNTSKDTSTTKNTPSTTPPNQLQITFPNISEPMSA
YSFSASTTRHMEGETLAMQNYIDISYGQQLASESALDNGFHDKDIQEPHMIESLEFIGID

GGDDEFMGMDGHTFLATPPAPNFLYEKASPPAGSHYTPMNSGSSLHADAYLAYLLNGAPL
TSLSAVESNTSEFVADDVKREMDLLEMISSSQFSSSQRSCSDCSFTYLY*
>CnMYB153
MVSVNAKPADGGSGGGAAVLFFSDPHHHHQPPSGPPMALPGIAGGAALAAPATAATSSSS
DDPSKKIRKPYTITKSRESWTEQEHDKFLEALQLFDRDWKKIEAFVGSKTVIQIRSHAQK
YFLKVQKNGTSEHVPPPRPKRKAAHPYPQKASKNGIPAAPLTPQATAPLQTSSMLEPGYA
LTTDTSILRNSTTSATVSSWAHSSVQPVSATHAIKDDVGPTGAVVANNCSCSSTESPSIT
WTTCEATDQENHAPSLHVMPDFAQVYSFLGSVFDPNTSGHLQKLKEMDPIDVETALLLM
KNLSINLTSPEFEPHRRLLSSYSADMEDVKPGGANKSLQVSETMNASFMVKGG*
>CnMYB154
MEEDSLGEELVVKTRKPYTITKQRERWTEEEHNRFLEALKLYGRAWQRIEEHIGTKTAVQ
IRSHAQKFFTKLEKEAVMKGIPPGQTHDIDIPPPRPKRKPSSPYPRKTSIVSVSLTGDAM
NNKSSSSVSLVGTSKQILEQGSDAPQKKLAATITLQRKEISVDGSCSEVLNFFQDAPSGS
VTSVKCSSNPNLNLFQDAPSASTSSVKSSSNPNPCTYTEFVPKMKEMKETATDKSSVSIE
ANKDLNINSMSYRDQEIEGLKGLHIDSQTNLAHGEGVDASKQHENLGSFSRDDMQGNQS
NSRHIPVHFSQRSSAEGKQTTCSNGHNFTAVTGQVGADENSKSFINPMIYVAPKLHNSSAI
PCVHQPFPGFPPFTQFHSNHDAYRSFLNISSTFSSLIVYTLLQNPAVHAAASLAASFSPS
ADVDSSTDSTPETLAGGFPARHMNPYPSLASIAAATVAAASAWWTTQGLLPILSPHVGFT
FNPAPATTIPTMATAQGPKDKTDGKEDAFQNLVQGDQAVNPDQSVALNAQGPSPKTSSSE
ADESDDSRSGERSHCTDVKASRINKLKPLAATGLHDSDKTSKKKPDRSSCGSNTPSSSEV
ETDTILDRHEKVNDEAEQAYFSDPSAGETNGRRSRNSGSINESWKAVSEEGRLAFQALFS
REVLPQSFSPPKTEDAMVNAISLPVDLNKKACAATCLDHLQSFAKGMSCRWNDNTGKGS
LTSEIGQGKLKSHRTGFKPYKRCSMEAKESRTAAVEETGNKRIRLEGEAST*
>CnMYB155
MGRSPCCEKAHTNKGAWTKEEDERLIAHIRAHGEGCWRSLPKAAGLLRCGKSCRLRWIN
YLRPDLKRGNFTEDEDELIIKLHSLLGNKWSLIAGRLPGRTDNEIKNHWNTHIKRKLLSRG
VDPATHRPINEHASSNITISFERREEKGAAFGHEERSSSEESSTWRRQQQQQLKCPDLNL
ELCIGPPVQQESLEPVKREGTLCFSCSLGLQKSNECKCNGFLCLSTAVLDYRSLKMN*
>CnMYB156
MDVQDKSGRPSLNPIHIPDRRMCMDIRTTSAIGHQLKESGSSGDEHAIKVRKPYTITKQR
ERWTEEEHEKFLEALQLYGRAWRRIEEHIGTKTAVQIRSHAQKFFSKVFREPGTVKAIEI
PPPRPKRKPLHPYPRKLGNSPSKGLPVMEQPEWSSLPVPSVFEQENSSPVSVLSAVGSDT
IGSTVSNPTHGCTSPMLSAARSDPVGTLSTEQENGCQSPTPSVQDENRSTSPGPASTCLV
TEDKSQMEVDLSSKDETPKEGSQMEVQATCLKLFGRMVFVTDARKPCSSGVGHTVEPPK
SLPAVESSSHTVSTDVDLQTPTKPGLQTPEQGDFCGGPGKSAWSPFNGGSFPMLYCLPPHG
DLMNSTEAACPPPPWWWTFCGTQPALPFINPQNMNSVQIMPQTCMEASDDKDGQKEGC
WTNFNTSACRAGIGDKNSDDVNSQQGVEHLKEPAPTLILEPSENSGFVCLKVNTEKSPPGF
VPYKRCLSEKQAKQHSQMVNEERDAQAVGLCL*
>CnMYB157
MESFLGEEWSPSENEKFEEALAEFDLNGQDWLEQLNETLPATKELTDHCINFSMDLGCIN
NGCNLILPGIAHSNVTKDGDSGILSMDNPLQEMNGIGSSSLNATIAAKTVVMEGGSSSSI
ADEVITLPSSAKPETERLTSPIAATEASMEAIAPTSTKGMPWTEEEHRLFLIGMDVHGRG
DWRNIAKNFVTTRTPTQVASHAQKFFKRQLQFKKRHRPSIHDINNINSSLPTKIPNYSMA
PSQFSISNMMADVGTSNVDNISQFSLPSLPQLPANYIMENPQISISCMKKIGVGTFDANN

VSQFDSPSLLQLPASYTIEHSQFPVSHIMTGVGTSVINNISQFDSPSLLQPSASYTMDHL
QFPASSMMEFGTSNINNVSQFDSLLQPPVLNDIVEHKRFPVSDVVGKEGEGRASLVAPTQ
LPNTFQQPFGNEDYLSSNMMIGPSFTSSFINTSTMWSP*
>CnMYB158
MVMEEANSSSLWSREQEKAFENALVTHPEDCSDRWEKIAADVPGKTLEDIKHHYELLVE
DINAIESGRVPVPCYPSSSDGGDHANEGSSGKKGGHSHGDSAHSGKASRSDQERRKGIAW
TEDEHRLFLLGLDKYGKGDWRSISRNFVISRTPTQVASHAQKYFIRLNSMNKDRRRSSIHD
ITSVGNGDIAAPQGPITGQINGGSTTSGKSAKQSSQPSGGPPGVSVYGTTIGQPVGGPLV
SAVGTPVNMPVPPAPYMAYGVRAPVSGTVVPGAPMNTTPVITTPKVASPYKKPTCWSLD
QRQRNIVEERRRYVAECPGKPQPGSLCLQSTSYGSSHFRQKRVDSVIDWMSRFSKRADSY
AKGIRDHVSLGPNISETVKGKLSLGARILQAGGVKKVFRQNFSVEKGEKLLKAFQCYLST
TAGPIAGLLFISTKKIAFRSDRSLTLTSPRGELARVPYKVLIPLERIKRASQSENANKPNH
KYIQLVTKDEFEFWFMGFVSYQRSFKYLRQAISDSEARFLR*
>CnMYB159
MVEEVKMEDGKNDRIQEISLPSCSPDSDGSCEMTPGTIPGRTSGPTRRSTKGGWTDKEDD
LLVKAVKKFNGKNWKKIAELFPGRSDVQCLHRWQKVLNPELVKGSWTKEEDDCIIELVA
KHGCKKWSAIAKCLPGRIGKQCRERWHNHLNPAIKKDAWTPEEEVTLIHAHQIYGNKWA
EIAKLLPGRTDNSIKNHWNCSVKKKLGSCLASGILSQPFENPSFKLNSHQEKLGSLKSHPT
KLGKELSSDTSLIGNSECLKVEDQHTPADDTECLKLEVKPSPTSDPKCRKSEAIFSANPSD
WQEDRSPCSGGQTSLSLSTVLPSHLTDLWHGNDSAYANLTDDLHGTPTLQSKALPSVFDE
FCSHVPSKEFSNKRRPKSPKMPEEHAFNKNDLTIKNMDLTYVTDTSKMRTFDSSNNDMVD
DHNQVTSTHLSCDGGNIGSLPYPALQLTHEGSSLSGEKKSKTESYVKQTQSPVQYRTPPI
ISPGHTYSPRDPIYYLRAAAKSFKDTPSIFRRRRLHDLIRTDILNGEHFSQSSHDSFVTR
S*
>CnMYB160
MGCRTCEKPKVNYRKGLWSPEEDEKLRDYMLKHGQSCWSAVPVKAGLQRKGKSCRLR
WINYLRPGLKRGFFSQEEEEIIMNLHAKLGNKWSRIAMHLPGRTDNEVKNYWNSYLKKK
VIKAQGSDPYGSATKSRDSTNQSQVTHAKTINKISSSESMEPPESSTTDLSPSMVHRMEFGS
FKGARQQHFPKFFADWPSVDQVIDQSSGSFVHANTPWNSNNSNTDEVLKPGSSQVVADS
KSDLLGGYGDSGIYGELQAQFEPVISQTTGDEFFDLLSLGEISVNLDTNHDVLF*
>CnMYB161
MGRAPCCDKANVKKGPWSPEEDRQLKEYIEKYGTGGNWIALPHKAGLKRCGKSCRLRW
LNYLRPNIKHGDFTDTEDRMICNLYASIGSRWSIIASHLPGRTDNDIKNYWNTKLKKKLFG
FPSSQRKPHQQQQQQQHLFSSNLGTRSVSFSSPLSLEGMQISHYLLNPIATPTFSSPLPQG
SMHQYRVKDNSSLLMFGSEQSCSSSDGSCNQISYGSNVDYELCNVGQQMGSLDSFLHCG
SAEAEKLLLAGGEASLEYNMDEIKPLVSPINWYSNPFGDDLSSPTAKDSSCKSQGRAMYY
Y*
>CnMYB162
MVRSPCCEKMGLKKGPWTPEEDRILVSYIQRYGHGNWRALPKQAGLLRCGKSCRLRWT
NYLRPDIKRGNFTKEEEEIIIKLHEMLGNRWSAIAAQLPGRTDNEIKNVWHTHLKKRVNPN
QSVQNSNRKPMARSDDAMAGMGFGPTTIPAAPNRKPKNPSPGPASPSQSCSDISTNSSTIT
GEINIAGIKEECSDSSEELPEIDESFWSEALSIENCDTPMDYNKIAGPPPNSLGHAFSSL
SPSNYNNDDINFWLKVFMEAGDFQELPDI*
>CnMYB163

MTSIRSRGPLFDELRFDRIRAFWLRSLDDAGSYRMERRSEMAAAVGMNSDGSVAPAAAA
APPKKDRHVVSWTPKEDDLLREQVALHGTENWTRIAAGQDRQTVPKKAEYYEEVLSGIK
PYLMNIKDLTRPLISSHTSTLAYYRPNLRLNEANAYGIGATADIDRMSPIVDKFHFCRSPV*
>CnMYB164
MGRQPCCDKIGLKRGPWTMEEDQKLINFVINNRVHCWRLVPKLAGLMRCGKSCRLRWT
NYLRPDLKRGALSEDEENQIIQLHSRLGNRWSKIASYFPGRTDNEIKNHWNTRIKKKLKLL
GLDPVTHKPLEQPAKCDGIMSDSASESYPSQVQEKGIEIMDLVSSNNQEELLVKNEKQKEV
QFDSNDTEVLLHGNVMPWENLDIEEMKPRPDPSSGSSASFSIDDTLYPSVQRESSLEASN
LCWFDTIDSFPSWEALYPLEDIFPFGKFP*
>CnMYB165
MEGRVEHEERRHLGGSGLVRRQDAAGGGRDRRGDSASHPWPTVDGPLGLSEEESVAYAR
SFFLWGFALLPLLWAVNCFYFWPVLRSRPSSSPSSFSRIRPYVLRSATGFLVVAVLLLSWA
LTFVIGGERLFGPVWQDLVMYDLADRSLFSEALSGASSYSKNGGELPVLEMNEVAAINGG
QGPPRVMVSSSAATGSDDTKACPRGHWRPGEDEKLRQLVEQYGPQNWNSIAEKLQGRSG
KSCRLRWFNQLDPRINKRPFTEEEEERLLTAHRIHGNKWALIARLFPGRTDNAVKNHWHVI
MARRHRERSRLFGKRSNCQDLILSSSDSTPNAPFTSFSAPPEAGSNSKHLRPDTRRLFEF
GSSTNDRIAVSPSSSSLSWAFSAGSTNPSSRQILGVDRRNYYSPKYNYNVLESSRFHDQP
LRPSNYHIFRSLNALCDTDYKRVAPHFIRFSGTGDSCGSEVIMTADKLDSLGDSSARAKM
NHRADNQQEDGDVSLACKDVPFIDFLGVGITS*
>CnMYB166
MGRSPCCEKAHTNKGAWTKEEDQRLIAYIKAHGEGCWRSLPKAAGLLRCGKSCRLRWIN
YLRPDLKRGNFTEEEDELIIKLHGLLGNKWSLIAGRLPGRTDNEIKNYWNTHIKRKLLSRG
LDPQTHRPVNGGATFAGTQQQEAPASVAKPANSGDEGRSSGASVDEDRFPDLNLDLSISL
PCHSPKRSPPSEPLARAAAAATTSANGYTQPICLCCHLGFQSSSDACSCQTNPNSQSHVY
RYIRPLGEGQHINYSS*
>CnMYB167
MGRQPCCDKVGLKKGAWTAEEDKKLINFILTNGQCCWRAVPKLAGLLRCGKSCRLRWT
NYLRPDLKRGLLSESEEKLVIELHSQLGNRWSKIAAHLPGRTDNEIKNHWNTHIKKKLRK
MGIDPLTHKPIAETVDQQQQQGHASVNGNDKNDQLCKQCPPKVAEEEAEEKSITSSSDPA
EADAFLSKSPGFCTDEVPMIEPHEIIVPCASSTLSISSASISSSCSSSSSDAKPEEIQLPCM
EWPESLYLWGLDDFMGLDFLNDDEDGKLGLDPFNQYQRSALDQESWKFALF*
>CnMYB168
MEALRRACMLTGAKPVAIDDSDSDSGGESSSTDDIDLLHRLQERFYIPSSDADSLPFIKP
LCSRPPPDSDEEDDFETLRAIQKRFTRYESDAVGRNSQNSIQDTEMVVGGMTSEQENHNT
LPKNGKSMELSEELLASGFTEHAEDGACNLNIGGFMKSRFPKSAKFFVDALKKNRSCQKF
IRRKLIEIEAKIEANKELKERLKCLMDFQVACKRKAGRILSQKKDPRVRLISMRKPRTIK
DSKAQKTSVNSSAIKVCAVYLYTKQTSSMAAGFLCLVNNVRLCEAVTFNMSFGVIYSVLI
VGLANYKKVSALCLGPAENSHVSKYKMVLKTFPVSLRKQTWSNMEKEKLAKGIKQQYQ
EMLILNSMTFESDIDSNLMSALSSSVLDITPEKIRSFIPLVNWDRLASMYLTGRSGAECEAR
FLSFLYLKLTNHFHKSVHCDLNLNSGEPLLYLNGKVKCIHHPDTCGIQESRWLNCEDPMI
NHNPWTILEDKKLLFIVQERGVYNWIDISIALGSRTPFQCLARYQRSLNPHILNKDWTED
EDAKLRAAVESFGDNNWQLVASNLEGRTVLFSGDRWLNCLDPSLNLKAWTEEEDAKLLA
AIAEHGYCWSKVATCVPPRTDSQCRRRWKVLLPHEVPLLQAARKMKRTVLISNFVDRESE
RPTIGPNDFTPLVNFSEAENNDSTTPGRKRSSDNQPKKSRVKSRRSFKENSTADGVINSST

NAVPTDSALVCGMNSDTAGGGSSKRSRKKKSSDDQPKKLRARLRRSVKENVAADGMVN
SSADKVPSGESLVHIVSTTTAESGRIKRTRRKTPSDNQPNNSMVKSRGYCKENIMADGMV
NSSTESAPAYLSLVPSASSNTAENGSIEGTGKRTSSDDRPKKLRARSRRSVKENVAADGMV
NSSADKVPSGESLVHIVSTTTAESGRIKRTRKKTPSDNQPNNSMVKSRGYCKENIMADGM
VNSSTESAPACLSLVPSASSNTSENGSIEGTGKRTPRFIKLNIETNAIVNSTGDGVPAGLS
VVPNVNFSTAECGANKIDQENTSKAKSRRSHEENKTTVDMVINTYSETIFGDASFVPVIN
SNNAKSGNIKRTRKKTLSDGPLLLPSKKSNARKRRPTENKVTECGELS*
>CnMYB169
MVRSQCGSEEGLNKGPWSAVEDKILTDYIKAHGEGRWGKIPKRAGLNRCGRSCRLRWLN
YLRPDIKRGNISHEEEDLIIRLHKLLGNRWSLIAGRLPGRTDNEIKNYWNTVLKKKVQARS
VPLKNLKCLNHDEERKSKSKTKVQAPSFEKPARCTEAELSSNQHGNQNVHKNEKALEID
DLAPESSLLAPQDYDLLNLLTGFNTDCLCLLDFEFFEHLGDNIQQDMGDCSNGKLCVSSD
DNVFCSRGMFENWIGTDQIQPCGSLDLGSLATFFGSES*
>CnMYB170
MGRKTCCSREDLNRGAWTAEEDGILTAYIKDHGEGRWRSLPKKAGLKRCGKSCRLRWLN
YLRPDIKRGNITAEEEDLIIRLHNLLGNKWSLIAGRLPGRTDNEIKNYWNTCLRKKVQGQH
QLKRCQQPTNETTLLVQTKAIRCAKLFITPQQRIAQNQERNVAIEPAMIGELATSKAPNS
ESGKHSSFTPKEDTWWDMLVGLDKSNASPLEAPDFDLMQLCGSESPRNYYTRREDFKGL
FAEMSDTTLPDFELELKSLANFLGCEEEWLGGDL*
>CnMYB171
MAEERIKGPWSPEEDEALRRLIEIHGPRNWSAISKGVPGRSGKSCRLRWCNQLSPVVEHR
PFTAEEDEAIVRAHRRFGNKWATIARLLAGRTDNAVKNHWNATLKRRLASLSSPSDDDGD
LNKRCRSTPSGSDASDFSGSHSPPPPPSGEDDPMTALTLALPGYGSDCGKEETSAGLGGI
RLPPEMEGAMREMVRREVRSYMEGRGYGVPEGDGVMGQMIWEEVRSYMAELGKTWY
DVPVDWQH*
>CnMYB172
MVRPPSSDKSNGERRPWTAEEDAKLLAYVSTHGSTGNWDSVPEKAGLDRCGNGCGDGW
PNPLTPYVKHERFTPEEEERIITLHAAIGNRWSVIATQFRGRTDNDVKIYWKSKLSKKLLKK
GIDPITHRPISEIMQIINSLPTAASNTARYQFPRGTQTNNLDLEDLNSALLSKSTPKHCD
ASTITIPTDASSNETSNSFSTVVAPSVHSWKDYLINDLFEGDSEPNSMEESTTGADKNGF
VDEWWGAMDGNIQGPSCDDGSSSFVDAMLDRHQETLAQFPELLEFLDDPDYIDPIDERF
K*
>CnMYB173
MGRSPCCDEIGVKKGPWTPEEDKMLTVFIQQHGHRSWRLLPKFAGLNRCGKSCRLRWTN
YLRPDIKRGKFSEEEDKLIVDLHSVLGNKWSAIAARLPGRTDNEIKNYWNTHLRKKLLRI
GIDPVTHTPRTDLNLLGSLSNLLADANFRNWNTLDNVLKLQADAALLARMQLVQKLIQV
LTNPPTSNLDLMGLLGSASLGNHQLSDPLQLSRLYEGLSNSSLSLHGSIQMPSSLPNLSFQN
LLNHNQACTNSSLSLDGEVAASEHGTESSGFSANSYAFPTSNTTSPLLPASPEDAHMDQT
QGPITSSNVSSTSEPFEAWEDLNLDDLSSDFGWKDTLE*
>CnMYB174
MTRRCSHCGHNGHNSRTCPNRGVKLFGVRLTDGSIRKSASMGNLAHYAGSGSGSGSPAD
GPPADHGTADGYASEDFIQGSRERKKGVPWTEHEHRMFLLGLQKLGKGDWRGISRDFVV
TRTPTQVASHAQKYFIRQTNASRRKRRSSLFDMVPDEPANGQLHPMVSDEPEAQSNNPLP
APPAQDEELESMDSNSNDGEGPVPKPESLPCSYPVFFPAYFSPYVSFPFPYWPGYGAADAV

EKRHEIVKPTAMHSKVPINVDELVGMSKLSLGESSGETGSSSLSLKLVGRSDRQSAFHAKT
PTNDTMVDSSASPIHA*
>CnMYB175
MSFRGRSLLRRNQVVSMEQVQEQELHELDLFHSHVADRLLSLLPPDSPKYSIEKPPSSLL
SFAFLSKLLDALLAVEAEFRPLLPLLGSGPPAAAAASRTVADLLDRAVKSLDICNAVSFS
LHSLHHWLRHAQIAASALPSPCPHLGAAHLLRARRALSKLLPSDAASGLRTERTGSFGRR
ETNSRFLSWSMSRNCSAHLAAPRGSEGGAGELAAAVYTMSAVLVFAMWAMGAALPCQE
RGGPPVAPPKHLPWAASMVALQERIAEEWRRRDRKAVAAGLLLELQEVERCGKVLMEVV
EEASGSRAAGGMSLERERAEEVAERAAELAEACRKLEEGLGPLEEQFKMTSGWLWPPWG
TVEEPLGEKDQSSAASVPPLWSNSQFWHWTSSGLQRCGKSCRLRWINYLRPDIRRGRFTP
EEEKLIISLHNIVGNRWAHIASHLPGRTDNEIKNYWNSWIKKKIRKPTTPPSTSPQSIELVQP
RFNSVDQLDAIINQNLSTKPAAENIFHTHCPIFTFDTGAGDSRPGSSARDELDATTLNSSL
WNPSQHQDQALSPLLNFSCGMDSTYLPPLVDGMGNMVPMEAQPCCVGDDGDTSKECFE
KQELNEWVDSQQYSNLLIWDQMQGSLGGEGLLTATTNMDSLVASFPSSL*
>CnMYB176
MEGNSLREELVVKTRKPYTITKLREKWTEEEHNRFLEALKLYGRAWQRIEEHIGTKTAVQ
IRSHAQKFFTKLEKEAVLKDSPPGQVHDIDIPPPRPKRKPSTPYPRKSWVGSVSPTGDAM
NDKSSKSVSLVSTNEQVLDMGNDTPQKKFAATLTLQKKVVSEDGSCSEVLDFFQDAPSTS
ISSVNKSSSIRYMEFVPKMKEIKETTTDKSSVSVEVNKDININVTAYTAQEIQRPKGFHI
DSQANLDHDKCTDTSKQQENLGSLLRDNMQSNYSNSKHVPVHLLDKNNEEVEQTTDSD
VHNFTAVPGQVGGVHENPKSFTNPMVSATPMIHNSSAMPCTHQPFPALPPFTISHGNQDAY
RSFLNIPSTFSSLVMSTLLQNPAVHAAASLAASFWPSADVDSSTDLTPETLSGGFPARHMN
HSPSLASIAAATLAAASAWWATQGLLPLFPPPHIGFTFNPPPTTTIPTVATAQAPEYKTE
GKEGAFQNLVAEDQVVNPDQPMALNTRHPSPKSSSSSEAEDSDNGGSGERSHCTELKASR
NNKSKPLVVTGFNDNDKTTSNKKTDRSSCGSNTPSSSEVETDAILDKHEKVDDEAKQAYF
SNPSGAQINNRRSRSNGSINESWKEVSEEGRLAFQALFSREVLPQSFSPPHAEDTMAKEK
EAITLPVDLNKACETTDLNHLHGFTKEMSNRSDDDTGKGLLTSEIGQGKLKSRRTGFKPY
KRCSMEAKENRTADVEEAGNKRVRLEGEASTR*
>CnMYB177
MVDASKDVNGSFPGEKKITVVFVLGGPGSGKGTQCANIVKHFGFTHLSAGDLLRAEIKSG
SENGTMIQNMIREGKIVPSEVTIKLLQKAMLESGNDKFLIDGFPRNTENRAAFENVTKIE
PKFVLFFDCPEEEMERRLLSRNQGRDDDNIETIRKRFKVFVESSLPVVEYYDAKGKVRKI
DAAKPIDEVFEAVKAIFAPFNAELSNANINRRMGLVNIMSFRDMRRIRTNKLSKITTKLH
CSVGACDKTTLFVYSTPLRAAINRIGMEFVEDEARPRFLFRGGGAAPFAAGDPDAGTLKV
HKLHAAACLSAASLLLFAAVSLSHWQTLSSLLAWASLSLLLAPFAPPSATGGDPSVGRGP
LLPDPPATRATPSEPDELRKRNLGRRPRSQHLPPPSGFPSSPVVPSPPISKKPEERSGDP
NSNGSGLAKEVEAEKEWTDEDFELLKRQISKHPVGEPRRWERIAEALRGRHGLDSVIKTA
KSLSERKPAAGDSFQQFLKQRKPLDKRVDANVGDSKGGILAENGESGKDGGEGSGRWSA
GEDLVLLNALKAFPKDVSMRWEKIAAAVPGKSKACCMKRVAELKRDFRSSKASET*
>CnMYB178
MGRSPCCEKAHTNKGAWTKEEDERLIAHIRVHGEGRWRSLPKAAGLLRCGKSCRLRWIN
YLRPDLKRGNFTEEEDELIIKLHSLLGNKWSLIAARLPGRTDNEIKNYWNTHIRRKLLGRG
LDPTTHRPINERASNTTTISFGRREEKSSDVLGHKEERSSSEESSAWQQQQRLKCPDLNL
ELCIAPPFQQEPVVEPVKRKGNLCFSCSLGLQKSEECRCDRFLGLSTEYY*

>CnMYB179
MVQELKTEGKKDDGTQEVSLPPCIPDSESSCETTPGAIPGRTNGPTRRSTKGGWTDKEDD
ILVKAVKQFNGKKWKRIAELFPGRSDVQCLHRWQKVLNPKLVKGTWTKEEDDRIVKLVA
KHGSKKWSVIAKSLPGRIGKQCRERWHNHLNPAIKKDAWTPEEEVTLIHAHQKYGNKWA
EIAKFLPGRATNSIKNHWNCSLKRKLGSYTASEILTQPFGNPAIKLNNIQEKVGCLKPHYAK
LVTPDPKPSVDNHFVASSGGLDFQNASIGRSDLSMDHSLLGNSKCLNVDHQHTSTDDSEC
SKLEVKLSPTSNPTCRKSEADFSANPSDCQEDRSLCSSGQTNLSCSTEFSCHLTNLLHGD
DSCHANMSDDLHGASTIQSKVQPSVFYELCSHVPPKESSNKRRPPKISEEHAFDKSDLTI
KNVDLNYETDILNMLTFQLSKNDMVDYHNQVTGTPLSCNSGNFGALPYPAMQFTHEGSS
LPGERISKLENYIQQTQNSVRYCTLPNISSNHTYNPRDPISYLRAEAKIFKNTPSIFRRRR
LVDLSRTDIVNVIQFSQSSRDCQKPDVSDTTKYVGRKLEEEFDMEFNYIHVGFFADGYFL
SIG*
>CnMYB180
MHILFRIRMGLGTSLFVHRHLAFLLRNTVANTWTCEKPKVNYRKGLWSPEEDQKLRDYIL
KHGHSCWSAVPVKAGLQRNGKSCRLRWINYLRPGLKRGIFSQEEEEIVINLQARLGNKWS
QIAMHLPGRTDNEVKNYWNSYLKKKVIKAQGSHPHGSATKSLELSNKNPLNHKETNNQI
PTSQSLELPELSTADSGLSLKHSMEFGSFKGTIHHHLPMVLFADWLSVDQVIEQGSGSFGG
SASTPWNSNNSNADEVLRPGSSEVIGQSNGDFLSGYGDTSIYGELQAQFEPVGQLPGDAF
FDLFTMGKFPGSIDINHDVIF*
>CnMYB181
MGRAPCCDKANVKKGPWSPEEDRQLKEYIEKYGTGGNWIALPHKAGLNRCGKSCRLRW
LNYLRPNIKHGDFTDDEDRIICNLYATIGSRWSIIASQLPGRTDNDIKNYWNTKLKKKLLGF
PPSQRKAHQQLQKQQQQQLFPYPFEIRTGSFSSPPSLEGVQNSQYLLSPMAAATFSSPLP
EGSMHQCQVKDSSSLLMFGSEQSCSSSDGSCTQISYGSNVVDYELCNGGQPMRSLESFLY
EETQKLLLGDGEASLDYNIEEIKPFVSPNNWCNSLFGDDLSSATTKDISFKTTGKAMYYY*
>CnMYB182
MASSHDVGSLQTEWRRSVEDVLHPKGTPDYQAPKDDLRDYGNRKEGAENFSAGSPEQR
PQKGQNTSSQSRRHGQERRKAKPWTEEEHRLFLQGLATYGKGDWKSISRHAVITRTPVQV
CSHAQKFFIRQERDMERKRKSMLDT*
>CnMYB183
MVRAPCCEKMGLKKGPWTPEEDRILVSYIHRYGHSNWRALPKQAGLLRCGKSCRLRWIN
YLRPDIKRGNFTKEEEETIIELHEMLGNRWSAIAAKLPGRTDNEIKNVWHTHLKKRVNPN
HSVQNSKPKVQSDVKTVMASRPEKLQSIPVAGHQKLEYPSLDPVSPSQSCSEISSSATNSS
IITGDISNAGIKEGCMDSSEELLGIDASFWSEELPVDDCGTPLMDCSMLAGPPLGDPFPF
SPSNFSNDDANFWLNIFMGAGDFQELPEIREA*
>CnMYB184
MTRSDGGGSRDGGLKKGPWTAAEDAILMEYVRKHGEGNWNAVQKNTGLARCGKSCRL
RWANHLRPNLKKGAFSPDEELLLILRLHAQLGNKWARMAAHLPGRTDNEIKNYWNTRIKR
RQRAGLPLYPPDIQCELALKRQRLHHNHPPSTNPPAPLLTNSTTITHFSSPPSSLLDPTGYFS
TLLPTAFNAPLPLSGHHLPFPSSGMFHTQQQQQQMEFNLGSFGLNSLPLPPPVFPNKMELP
STQMFTELPFSGGLQDTLLQEVQPCGHLQSKGLLLELVSGDQDVKLSHLCGGGGGGVGG
EGGGSKRITSPQDGLMESAAKWADSNWSIGTQMKKEPTENDTSPVEEDISQLLNMIPAVN
AAATTPVSEWYKSDSGEISNGQSSVITEEDIGLEMQQLVSSLSNPPEGQDLSIRSYPWSNI
PGIC*

>CnMYB185
MEVPGRVFGSATQPNVEEMDLRRGPWTVEEDLKLINYIAIHGEGRWNTLAHGAGLKRTG
KSCRLRWLNYLRPDVRRGNISPEEQLLILELHSRWGNRWSKIAQYLPGRTDNEIKNYWRT
RVQKQAKQLNCDVGSKKFKDTLRYFWMPHLMERIRAASGSTPLQGPDPQLPACPAASSST
DFATMRLSSPLPTDSSSGDCYFSGSIPDGERNTSDGIQEARLEMVDGWYDNGGLLDFEQG
GGWGQNSWSLEDIWFAEQQNKE*
>CnMYB186
MGKHSCCYKQKLRKGLWSPEEDEKLLKHITKYGHGCWSSVPKLAGLQRCGKSCRLRWI
NYLRPDLKRGSFSQQEENLIIELHAVLGNRWSQIAGQLPGRTDNEIKNLWNSCIKKKLRQR
GIDPNTHKPLSETQGGEDKALTISDKTSGSSELKPRAPTSGNLNSTVPEPAPPSMSVAEKN
QTEKSSSKNLVAPTKDFFLDRFAASHESSNSVDYFPFRNSAYSPNLSMHQNLPLWFNQNS
RLFNMSTELNCNMISTAVPSGSKSIVISTSMDLKPTVMSIAEDEPLQGFQYLEAGNSSNN
SASSGNASSGTELQSNNSFFDSSIFQWPDLVLNKEDQIQLGGEPEDLKWSEYLNGAISPS
TDFQNQSQLFYGAVKEANQFSIDEFRSWLQPPELFGKDFQKISTTFKQI*
>CnMYB187
MPSLRAQIPLPGLDSGYDATQPGIRKGAWTVEEDALLRRCVEKYGAKEWRHVPRRAGLN
RCRKSCRLRWLNYLRPGIDRGSFGEDETDLIVRLHGLLGNRWSLIAGRLPGRTANDVKNY
WNSHLSKKLDVRDHKERSKHDNKVFKPRPQTVPRTWIWSRDHLSSASEIQQEESDMQAI
QSSSNNHKTWVDAQIIGVPNVENVITKEMQTDNRVIQDSEDNKDDESLTGVGGWEDLLQ
DIGLWGDLGTI*
>CnMYB188
MNRNTNGNDQADSPSIDEGSSGGSLTGGGQVLKKGPWTSAEDAILVDYVKKHGEGNWN
AVQKHSGLFRCGKSCRLRWANHLRPNLKKGAFTQEEEQLIIELHATMGNKWARMAALLP
GRTDNEIKNYWNTRIKRRQRAGLPLYPPNLGLQASSENQQSQTASEYSTGEKRPNEVLQG
NSVDIADAMFDNLNADQGALSYAPPFPDISVNNMLSQGFGSQNNNFMNPMWNSVKRLR
ESESVLLGFHGTVSCGLPTVEQVSHEPSEKIQQTFGLGYLYDPEPSSKIMVPFDGAVPGSHA
LLNGNFSASRPLGGTVKLELPSLQYPETDLNSWLTCPSPPPEEIDTYIQSPPTTVLVRSECVS
PRNSGLLEALLHEAQARKNQQSEKSLSSAVTQSDMVESLGFNLWEAEWEEHNDPISPLGR
PAASVFSECTPPFSGGSLDELQPSKAPSFPAGSDIMVAADKHVLAPNMGDRGILPSPFFL
RPDALLEGSDWLENSEDAKEHSTLNDKVVTLVDEDFCSEYKQLPTGMASALAQRLDLDS
YP*
>CnMYB189
MGRPPCCDKVGIKKGPWTAEEDIILVTYIQEHGPGNWRSVPANTGLMRCSKSCRLRWTNY
LRPGIKRGNFTSHEEGMIIHLQAALGNRWAAIASHLPQRTDNDIKNYWNTHLKKKIKRNP
TTVDTHKTPSNSTITCNELISNGCNSETTNSDISTSHSTSTYAWSTGNISRLLEGWMRSS
PTANLKPTQDEQHQSIRAFNKSSSNAINQAFAREELRSDRERGNPISQAYMKWEQVKNDR
ECCGPIPHKLFSLESSSGNAWEKSSVDSTFYRPQSTLADAEANQRSDGPQRVPLSLFEKW
LLDEAPGQVELVELSADLCSDAMLQ*
>CnMYB190
MGSLNTKSAAEMGFFPPLLPPPPPSGPFLGASSSHSVERECWPGDMGFPSEEKQGEGFQG
FEGGQGSPVDESDGDEGEEGENSGGRRDNEGVHAKISVRCHWRPSEDARLRELVAEYGP
HNWNLIAEKLEGRSGKSCRLRWFNQLDPRINKTAFNEEEEERLLVAHKFYGNKWALIARF
FPGRTDNSVKNHWHVMMARRRREMCNGYRRMKPSATFPNPSVPILPRMVEVGSGNNNA
HSGESTITNNRDESASSSPGLSLTSSNNMVIPGFVSRFGPSELPQHFRFLMGSHEKPVAATN

VCGEKFGTPASCFYNSAPMLMAMGVHQAGHSEATWRASTTESEANPRNNAIIHAEREND
GEKMTLPFIDFLGVGAT*
>CnMYB191
MVTVKEETRKGPWTEQEDQQLVCFVGLFGERRWDFIAKVSGLNRTGKSCRLRWVNYLH
PGLKRGRITPQEERLILDLHSRWGNRWSRIARKLPGRTDNEIKNYWRTHMRKKAQERKRE
TISPSSSSSSLTYQSPSLSEPPSQVGLEQRELNEAGGCCCMTAGFDENEDGVKGYYSMDQI
WDEFAIPEAVSGLSLEECKVEVCDTSWPPMPSPMWEYSSEPLWKMDDEEFKMFPPVDDLL
VSNYQHGREFCN*
>CnMYB192
MAAVKIEQSCVENKQSAAASSSSLSEGSYGFSRMSPAVSSPATSSPSHRRTGGPIRRAKG
GWTPQEDETLRKAVEAYKGRCWKKIAEFFPDRTEVQCLHRWQKVLNPELIKGPWTQEED
EKIINLVQKYGPTKWSVIAKSLPGRIGKQCRERWHNHLNPLIKKDAWTVEEELTLMNAHR
IHGNKWAEIAKLLPGRTDNSIKNHWNSSLKKKLDFYLATGKLPPVPKPGEHSGSKDIGNPA
SGQLFLCLNKGSDTSGKALSESAGSTHSLLPSESCKLEEHRESLGPSTIQISCSETFTGV
PIRELNSSTVEYQAQAMQVDNICNGSDPGERFGNDTNAGEIDKDNKTSGINLLSELSSTF
GSLYYEPPLLEDLGVSLASTLLNMYYPNQQSYNLGMGTSPLGYFTPPFVSGKSSGQHSAE
SILKNAARSFSNTPSILRRRKREANTPLPSDRTVQTDQIKIQDASCTPIEGKYGSDQQPY
KSSNLLGASRCNDCGIVPYNGKIFNVSPPYRLRSKRTSIIKSVEKQLDFTSTEDNIDGNA
KSLSLAVNSSSRSADCNCNLPSIQGRKLNECPVGLEILANDSALIQLS*
>CnMYB193
MKCFHSSAAGDEAGLGPRPSRFSWARSLSVASAGWRAELDHGPQDLPRLEEDLSHPLCRQ
GQLPDGLRAFTFAELKAATRGFSRALMIGEGGFGCVYRGVLTVPGEDDDAETMDVAVKQ
LNRNGLQGHREWVTEVNFLGVVKHPNLVKLVGYCAEDDERGIQRLLVYELMPNKSLED
HLLARVPSTLSWEQRLKIAQDAARGLAYLHEEMDFQLIFRDFKTSNILLDEDFNAKLSDFG
FARHGPAEGVGHVSTSVVGTVGYAAPEYVHTGKLTAKSDVWSYGVVLYELITGRRSVDR
NRPRNEQKLLDWVRPHATDPKKFHIIVDPRLEGQYCVKSIRKLITLANRCLMKQPKSRPR
MSEVVEVLEQIIEMLNGETGGAITAPSVREDTTDATENGNGRSRVFDIRELISLKNRAMQG
RIFSLDFSLRARVPALLPAASVAHAWYQKSSNLPGRIGGDGWWPLQMKDRQRWRAEEDA
VLCAYVKQYGPREWNLVSQRMNVPLNRDAKSCLERWKNYLKPGIKRGSLTEEEQRLVIN
LQAKHGNKWKRIAAEVPGRTAKRLGKWWEVFKEKQQREQRESINPVVSSFEQGKYDRIL
ENFAEKLVRERQITPLLMAAPLLPPWLSNSSSSHRPSSPSVALTLSPSTTPPASIPWLQTDGS
KGLAGSQQNMVSGVPSMGGQMVSELLECCRELDEGHRAWAAHKKEAAWRLKRVELQL
ESEKACKRREKMEEIEAKVRALREEQQLALERIEAEYKEQILGLRRDAEAKEQKLAEQWA
AKHIRLTKFLDQMGCRRWSGTDVDGR*
>CnMYB194
METAADSDPTPLSSSPPPPSPPPPPTAAAVESATKSPAADGGTNEPGAAAAAEGSASGGG
GGGGAAPADDRVKGPWSPEEDAILSNLVNKFGARNWSLIARGIPGRSGKSCRLRWCNQL
DPYVKRKPFTEEEDRIIIAAHAIHGNKWASIARLLEGRTDNAIKNHWNSTLRRRCFQAEQY
KAATCDALQDASAEKAKGSSEESPSIGDVNSFKSLEGRDVNSKENMSDNSEDIVNGRDDT
SEVRDPPYLFRPVARVSAFHPYNHVSGESCGSLLSRPVQMHGLLPQAFQNEAGVSKLLDG
NCSEPQVPPRCGHGCCSIENDCHPPNSLLGPEFVEFVELPAISNQELFSVASELSSIAWL
KSGLQISSSCMYSGSPGPVAPPGGPVQ*
>CnMYB195
MVTNSWMEVLPPTTPYFPNSSWLLGPKRSGNWTQEENKLFEDALAHFDRDTPDRWEMV

AAMIPGKSVQDIITHYKDLEDDVSKIEAGRIPFPGYSSSSFTLDWENSHGYEGSKPSYCVG
AKRSGGRLDQERKKGVPWTEEEHKFFLLGLQKYGKGDWRNISRNFVLTRTPTQVASHAQ
KYFIRLSSGGKDRRRSSIHDITTANLPNNGPPSPSQASVLSMRSSSAVNTTTISDQFSVMVD
SNQPNEAASGHVFSPSAHGNQFMQPPYEVASYGMKLPAQNSQRGNLQDSMISDQNMLFQ
MHAHG*
>CnMYB196
MKTVSAPGRIDGVVEKSPPPISRPAPAPPFTPLKRFLASSCEGELRLEKYPLDKVEQNAE
IPPGATGIPSPNGILSNDFSFYGGLVGCSGSGKDSDMSRENYGERKGMRSSSSTAAAAAP
VVKGQWTAEEDSMLVRLVKQHGVRKWSQIAKKLVGRIGKQCRERWHNHLRPDIKKETW
TEEEERLLVEAHKKVGNRWAEIAKHIPGRSENSIKNHWNATRRRQNAKRKKTAQGGGCR
PSVLQEYINASGRMLDLQDKYCTTKITPPSPPHQFHITFPNVQEPLSAHDSSLSTITHMEDC
MPSIEEFLNQSTTGTTAEEGGGFHVGDVQDPRVPADSLHILDFDRRNYSIAGADQCMPLAP
PPGLDFFHGEANGLTAAASDGTATNSSSTHLYSDGYISYLLNGAPSFSTDAFAGTDTQIA
KDQASSSCKRELDLIEMLSLQLSRSQSQSRTSSSI*
>CnMYB197
MDFGCFNTHTGDGLPFESGVYPDGPQDFHHFDDAFDHVLAVIDGNPSGFDLKALWSPPH
GLQTRMPVEGLQSRTSVSSRYPQRSLSSHTGPDPGQSSVVLNVEPLMVFGPPDELSCVTG
DNKSCRGGVEMNRKCTPVKKSSGRKTHKKPNGVKGQWTPEEDRLLVKLVEQHGLGKW
SHIAQMLRGRIGKQCRERWHNHLRPNIKKDTWSEEEDKILIQAHSEVGNRWAEIAKTLPG
RTENSIKNHWNATKRKQFSRRRCRRNSKHPKPVSLLHNYIKSLALQGSIPPLTVPAASAAA
DDRLKSTESNDGGSKTSMALMTCIPIEGLLSVDVGAHVCGGSNDILACDLGDMANLLLD
NNKVIDVPPERYDMGMGCLFDQLGCGPGIAKKGCLDMEMWWDEMPMPCEANMYAKV
KKEMDLVEMIAQNKNTDGSSRNQL*
>CnMYB198
MGRHPCCTEQKVRKGLWSPEEDEKLYNHITRFGIGCWSSVPKQAGLERCGKSCRLRWIN
YLRPDLKRGNLSQQEEDLIMSLHKVLGNRWSQIAAQLPGRTDNEIKNFWNSSLKKKLKQ
RGINPITHKQLRETGAQGEMPRPAVKPIFNPFPSFECQAATDPTGTNANLYHQLQQTYRPLE
QNEFMKNFDNNLVSRSSSGYWNYGEVLNVSDTYAIQESLNGSIDWNCGIAAQMDSMLGK
EVLGWSSLEIKLESSLQTEIKTYHQRLNPCQGLQHVESSEEFSSYLTTSLSQDLSDACLDV
SQDELARELNGNLLSS*
>CnMYB199
MFQSLVFISTLRKVSELCMWIYGGEVSELCSLLLIKELLLLPPSNTDVSEAICSMELPPF
SAPNAQPLQCPNTPSPAVLLLLLLIEICVDQYPSSVVLKISFLAREPPCSSLYSSDKMCS
RGHWRPAEDEKLKELVARYGPHNWNAIAEKLQGRSGKSCRLRWFNQLDPRINRSPFAPEE
EERLLASHRIHGNRWAIIARLFPGRTDNAVKNHWHVIMARRCRERSRLHSKRAYRSAVKE
EKSKEGKERKSEARNIVTLMEKYCRGCSDSIANNHQNHKEFHSEIHGYYPSMVADGHRH
RESSIEFYDFLQVNTDHSNDTTCCSTIEEQEETKENCSEAVVPFIDFLAVGSSS*
>CnMYB200
MNRGAWTALEDKILVSYIKTHGEGKWRSLPNRAGLKRCGKICRHRWLNYLRPDSERGNIS
EDEEELVIRLHKLLRNRWSLIAGRLPGRTDDEIKNYWDTTSGKRVRGQPSEDSKPRPVAR
SKGKSLETGPPVASVPSPTGTPVILTKALRCTKTKTTCTADQFMSSLPNTDQHPDSELRE
TRNLSEVPQYESLDDVGMGSVDTDMAYPLMGLDLWQDIGEGTLVGNNGMIQGDGSTSF
DMPSKYSGTFDGAMLKNQPANECFHPDFMTSDISSIICFPFGFRRALVLKNIQMMTLA*
>CnMYB201

MCRKPCCSKEGLNRGAWSAHEDKMLADYVKAHGEGKWTDLPKRAGLKRCGKSCRLR
WLNYLRPGIKRGNISHEEEDLIIRLHKLLGNRWSLIAGRLPGRTDNEIKNYWNTKLGKEVK
KAILERFNGEKVQSKSKEEAVASLKAKAPIEALDKQSHPVIRPKAVRCTKVYIPPHRDDDK
MANGNLEPHSNDEDALESILEDVEPSNFFVDFDMEKLLSELYDDDFLLHG*
>CnMYB202
MGRSPCCEKEGLKKGPWTAEEDQKLLAYIEENGHGSWRALPVKAGLQRCGKSCRLRWT
NYLRPDIKRGKFSMQEEQTIIQLHALLGNRWSAIATHLPKRTDNEIKNYWNTHLKKRLAK
MGIDPVTHKPKSDALSSSDGQSKSAANLSHMAQWESARLEAEVRLARESRLRTTAAPFQP
QSPSSSAPPPPGSDSVAPPAPPLDVLRAWQGVWSSKPATGNPGGRIDLESPTSTVSFSDNAL
PITSSRVMDANSTACHQGPAQELEEEQDLEWKCFEKQPLTKDHRMDSFASFSVDTASVFG
GESSSWLPETSGGEFDAGFTGMLLDGSDDRNSSDGCGDSNYNAEGGSCVEEDGEEGDEN
KDYWNRILNLVNSASPSRSPPMF*
>CnMYB203
MALQQRGGSEGMETRCGFRSYSSSPSSSIGVFADIGPLSINPTTSVGSYRCTKSSDMTSW
TFQFMQKSQVGNKCFDGIDGSGEEEPDAIGEESPDGENTNESSQGGKEIVDCGQSKLCTR
GHWRPVEDSKLRELVAIYGPQNWNLIADKLEGRSGKSCRLRWFNQLDPRINRRAFSEEEE
EKLMAAHRLYGNKWAMIARLFPGRTDNAIKNHWHVIMARKYRDQSNAYRRRKLSHDVQ
RRPEEGATTTAAAAVCVFGHCPSPSSTFAAAISGSVGLHHMTPGAGDSVSSHNGFYTKRTP
FDFLSGKERHDKANFRSRSSWDRPRDDSMCFYHHPPLMLAMQQSSHASHYSDSTALSAS
RVSAIESSSSEENRESSHFEATITPPFIDFLGVGAT*
>CnMYB204
MGRPPCCDKVGVKKGPWTPEEDLILVSYIQEHGPGNWRAVPTNTGLMRCNKSCRLRWTN
YLRPGIKRGNFTEEEEKLIIHLQALLGNRWAAIASYIPERTDNDIKNYWNTHLKKKLKKLQ
AGDGSPTSGGPSVSKGQWERRLQTDIHMAKQALREALSLGNPNCQTELKSFKQLSQPATS
SSTTNYASSTENISRWLQGWMKNPPKPIAAQSNSEYKLHSIKTMVGTDSSSSEESASAAN
INGGTVSPTVLESLLNVGSSTPEMSETSLFQGESKPTSEAQVQLSLLETWLLDEVLVPVK
VELVDMPVGDTTELI*
>CnMYB205
MGRSPCCDENGLKKGPWTPEEDQKLVHYIQEHGHGSWRALPKLAGLNRCGKSCRLRWT
NYLRPDIKRGKFSQEEEQTILNLHSILGNKWSAIATHLPGRTDNEIKNFWNTHLKKKLIQM
GFDPMTHRPRTDFFATLPQLIALANLRELVDGRPWDDHAARLQAEAVQAAKLQYLEYLL
QSAATVANTSNTNSLSTITTTEMDPVSLLSPPQMPSFPSVPSPSLIESISGQNQISQLPDFQ
FPCSFSDQPMSNETNQGSDFSVLSQGDQNSTPGMILMSPHSSLPPLTDVSAGNPGDACST
SSCGGSGTPSFWPELLLDDPFMTEFA*
>CnMYB206
MRLAFIHLVPYMYALAGKGNLDTPWEASDIIDLNWENIGFGLVPTDYMYIMKSSEDGTFA
KGELQRFGPIELNPASGVLNYGQGLFEGLKAYRKEDGSILLFRPEENALRMRMGAERMC
MPAPTIEQFMDAVKLTVLANKRWVPPIGKGSLYIRPLLMGSGAVLGVAPAPEYTFLIFVSP
VGNYFKEGLAPINLIVENDFHRATPGGTGGVKNIGNYASVLKAQKLAKEKGYSDVLYLDS
VHKKYLEEVSSCNIFVVKDKIISTPATRGTILPGITRKSIIDVARGLGYQVEERLVSVEE
LVDANEVFCTGTAVVVSPVGSVTYLGNRVEYGNRGVGAVSQQLYSALTRLQMGLSEDKM
GWTVQALISFPGIARSDEDNADINWDDLGFGLVPTDYMYVMKCSEADTFSFGELNRYGNI
ELSPSSGVLNYGQALFEGLKAYRKEDGCGFLLFRPEENAWRMQMGAKRMCMPAPSVDL
FVNAVKQTVLANKRWVPPQGKGSLYLRPLLMGSGPVLGLAPAPEYMFLVYASPVGTYFK

EGLAPIHLVIEDEIHRATPGGTGGVKTITNYAPVLKAQVQAKAKGFTDVLFLDSVQKRYLE
EASSCNLFIIKDNVISTPITRGTILAGITRKSIMDIAWDHGYQVEERLVSVEDLIDADEVFCT
GTAVVVAPVGSITYQGERYAYKTGSETVSQQLYNALTAMQMGLVKDEKGLKRCGKSCRL
RWLNYLRPNIKHGGFSEEEDNIICSLYVAIGSRWSLIAARLPGRTDNDIKNYWNTKLKKKL
LGGKQHKNQQRAHRVCHKNQEAKESKDGSSFWPTPPNIPTNQFKYQVDGDGHASIRKFS
TNVDGRFSCNYGAGHETPLASFLEQQHLCTGSIELSEQVTSFINGCSPSGYHMPQGPNEIP
VELDEMFCGNSVKLEGLDSFFGVGNMVGESNGTSTSGSMNWNEVSPLIHPPVVTSYQGM
QQQYCLPDELVHLLGAQ*
>CnMYB207
MMRKPDPPAKINNPTNNNSSGATKLRKGLWSPEEDDKLMSYMLSNGQGCWSDVARNAG
LQRCGKSCRLRWINYLRPDLKRGAFSPKEEELILHLHSILGNRWSQIAARLPGRTDNEIKNF
WNSTIKKRLKNSSSPSPNHSGSPEPKVGMGSLASLNEQEIKTMCIDSASSSSLMQGLSIS
NQYDLLPLPDVSSGMNGLGTSYFHEPTSFPQVGFGNGYYGSHDVMMEGDLMGGGGCEL
FIPPLESASTEENGTSNNNTRNISEPYHSSNNDVNKINSDNAVGSGIYWQGGNMKMEEWD
FEDLMKDVTSFPFLDFHAE*
>CnMYB208
MGRSPCCDKVGIKKGPWTPEEDIILVSYIQEHGPGNWRAVPANTGLLRCSKSCRLRWTNY
LRPGIKRGNFTPHEEGMIIHLQALLGNRWAAIASYLPQRTDNDIKNYWNTHLKKKIKKYQ
TAIGNYMISTDSGSTRHDIISKGYHKDAIRNPDLAGSHSPPSRFHQSPSTYASSTENISR
LLEGWMRPSPKSTQDKNQRSNVMDNEDNESLGDQGRHAAATKENLDSLISLENLSSIVLH
GPELAPADAETKHGVVEDQQQPPLFLLEKWLLDEATGQVDGLMELPADCCSNPML*
>CnMYB209
MGRAPCCAKVGLKKGRWTAEEDEKLVKYITANGEGSWRSLPKNAGLLRCGKSCRLRWI
NYLRADLKRGNISKEEEEIIIKLHAAVGNRWSLIAGHLPGRTDNEIKNYWNSHLSRRIYSFR
TAAGDVQAVPVDLSKLPTGGKQRRGRVSRSAMRTNIIMNRIARREKNEKGVGLPRSSAET
PSDEGRSMALDPEQQQASCVTFEGLKQGALGPMEEMVSGLLSPGPIESGLWGANTEMEA
MHHGATEESSVGWGSHEERETGVMSSSEEREGRALALNGNGDGGDMGLEEGRGSGIVT
KGEEEVGSNQVDKFLDWDLGGLEAKLWDETGEMWPWQWDGPVTVVSGTIAFLLLTHG
YGTSIYANERNGFCCRCKWHGKKNECFDKESETECSMDGGEGGRDNDDDEELMGVEEE
QGIDSKADEFIAKFYGQMKLQRQVSLLQYNEMLYRSTS*
>CnMYB210
MEMGRGLGVGVHGRTWSSANPPSEEDMDLRRGPWTVDEDLILMNYIAAHGEGRWNSLA
RCAGLKRTGKSCRLRWLNYLRPDVRRGNITPEEQLLILELHSRWGNRWSKIAQHLPGRTD
NEIKNYWRTRVQKHAKQLQCDVNSKQFKDVMRYLWMPRLVERIRAASGSSPAAIYQNAT
VPMAGGDQLVEMGSEVGQVKPSPETSSAAGSSLDSVGLQFSSPPISDCFSDCYGGVQGGEI
RNGDGIPDVQIGGWWPESLPSPGGDSNLGLPDFEQGAWGESCLWSVEDIWLQQQF*
>CnMYB211
MAPQHLRIAPEMGFFHPPPPPPPPFHGGLGSPSGGMECRSSGMGFSMEKEQNKDCGFIGG
QVYHGDEKEQSEQGEDGTHENGQTKLCARGHWRPAEDDKLKELVSQYGPQNWNLIAEK
LEGRSGKSCRLRWFNQLDPRINRRAFSEEEEERLLAAHRLYGNKWALIARLFPGRTDNAV
KNHWHVIMARKHREQSSSYRRRKSSTSSPNPPSSQALPNRMEVNTSNIACSGESTITSARD
ESASTCTGLSLNSFTSGTIPNFISRYSPPQQPQRLEFLMGSDEKLVAGRNGCYEKFGDRHG
SSFPHVMMVPCFDQSGFSNSNYKALATESVTNHRSRAWVHGETDHKREKISLPFIDFLGV
GAT*

>CnMYB212
MVVMEGGGEGRVDGEQRIKGSWSPEEDAQLTRLVERHGPRNWTLISSGIPGRSGKSCRLR
WCNQLSPAIHHRPFTPAEDAVIVAAHAKYGNKWATIARLLPGRTDNAIKNHWNSTLRRRR
RAAEAASAIFSPATPAAAPTSVPLPLAADSTDPGESDSESAGKRPCRRDEVGGSEVATVV
AAALPPLLPRPPRAPIAADPATSLTLSPPGEGGATGSGGGGERREARWDAEMREMCLVSI
MREMIAEEVRSYINRLRSEGGGAIPIITTSESASNRQD*
>CnMYB213
MPPEPVPWDRKDFVFKDRKHERGAGSDALGGGSSSSTTRWREPYHGPRDFPRASPRRPPP
GQYRQSGGYHQLYPENSGAHGCTPSRSDRFWLEDEGCRPSSGRYGGGGGGRSSSGGSRE
SRGSFRRSPYWDSSDFSRQQHHDPPVTAQRSVVVPISPASQPPLKDQNDKTGGGVDDGSG
TGHRFDRDHSLGSISWKPLKWSRAGSLSSTKAGRSELEDTGLEVLIPPGKETPIRSPVASP
APSDEGASKKKPRLGWGQGLAKYEKQKVEGSVDASGTAAKEVLNETSPKVVGLAGCPSP
ATPGSVTCSSSPAGIEEKPCIKVVNGDNDSSHYRDSPEEFSIKLGHMEGDPVNILTALLAD
LWQPDDASVGDSTFSRQTAMNRLLLLKEDISRELEKTECEIDLFENELKSLDSDPENDPH
QSSFTSPANTAPEPCIESSNVASKDFNPLKGHEFTSSVVILVENNALPTNACDAEIKGIN
VNSPQTVSSRFNNSASYRKGVCDHETEQLAECSEIVENDRLKVPEIQHFVLSDDVERPAT
LCDNGDGSRGEAGSSSDNGNSEASLCGKTDCNLITLIMASNRDAANKASQVFHKALLTSP
PQLDVWGSDKLLSYRQNDFKIKEKLAIHKRLLKFKERVLTLKFRALHHLWKEDLRLLSIR
KHRTKSSRRFELSSRASQCGSQKQRCSIRSRFALPAGNLTLVPTTEIVEFTSKLLSDSQT
KLYRNNLKMPALILDENGRKQTKFITHNGLMEDPISFEKERAMINPWMQGEKEVFMEML
ATFGKDFTKISSFLNHKTTADCIEFYYKNHKSESFREVKKRLNLKKQWQRLPTSSYLGTSG
KKWNREANAASLDMLGAASVVAAHSNGNVTSQQRYSGHGAHDGLKVSCGSYGSLERV
GNVETPEHERETIAADVLAGICSALEAVSSCVTSSIDPVEKMNYMAKEWPLTLEVTQNFD
EDDTFSDEGCGELDSADWTDEEKSMFIRAWSMYGKDFAMISRCVGTRSREQCKIFFSKAR
KCLGLDVIHQRTSNGGMPMSDTNGGRSDTDDACAAEMDSAICSTQSCSKMDADVSQSVA
NISSEGLIHAASTPLQAEADKSSEQDVVGGISLEEDEGKVDKQASVLHDNKLVSEGDNPQS
VQDADAALRCNASIPHEAVGCVDAEMKMEGGSPIVSSVEPVFTVCMEVGSKSCIDGVVE
KKDTGGSADVSKKEVDVSLLVPETVSRNRQQMVDLGATSGGTICSASDSKADPNALHPG
NKVDDCPRSTCAPIYPHQMPLDLLPCLQNKPQGISSTQENAHSVPSNSVLPDPSSACFEGPL
VVAPQATLNFEEHGNKWHKNPVARDPYQVDQPLHMMRNPSLNQVDQPVCILTGYPLQVL
NQEVKKAADPVIGENAVFMESHPKRNGVSQSNQFFISEMYGDHCNGSSLSHSRPGLLFPP
RNEAQPEARLKHSSQNSCSEPEEQAHRTGDVKLFGQIICHPSSSQKSNSSSHECNSKPSSPK
MNGSSALKSSSGGKAGTLFASRPGSSGHGGLGELPLRSYGLWDGNRMQGGFSSLPESAV
MLAKYQGSLAGMSFYSAKDGVPSRNRILTDYQQSYMQHLSSDEKRLQNFCELQKRNGIE
AVSGFQQQGRVPRLGSNMVGGGILGGGGGGVSDPVAALKLHYAARAKVLGGELESWRG
DIGGR*
>CnMYB214
MSHEMMMLITKIPIAFHDCSDRLMWGTNFSKYIRIKDIYPLLFCDGHTETIPMSWMWEMK
VPPRIKYFWWKVPCKALLARRRIIDDSVAACDLCPGMQEDLNHVLISCSFARNCWALIQN
MIPDELLFDSMLGLLDFLSATCVEENYRSIGIEETTGHTILPVRRCGDSMLLNLSSPIPL
LVAWSPPPPPMEALRWSRIARRLPGRTDNEIKNYWRTHMRKKAQERKGSSPPLSPSSSSS
NPSSSLGDPPLLEAVVGKLHEVDGVVEGTSSMIGFQGNDEEDVKGYPPMDQIWSEIASLE
LIFEGYKNEACINVSCPTSTVELLHKLFYATWELEALQSSIEQERREREENINQLIQILK
ATTQERDEAREQLQVFLRKISEPNFLAELLHSLTHLHPHSPQIWEPRANSMVIDSEGLSQ

IRNHHSYGSSSVESFFVTSMNGAGSGLPQQPLFLCENNHSSSMMISPGTAKYDRADAIIG
ILSAKRPLPDKGKLLETMLKIGPTLETLMVAGPIPHWRNPPPLQSFQVPPVTIKACYAQL
LNRAAILNSNHSIQSCLYEKCDGLSRMHGASILECSGKVSLCRRSLPGVSSNHSFEAKRE
FTQ*

Dataset S2. Fasta files of MYB proteins in *Arabidopsis thaliana.*
>AtMYB-1
MDTNTSGEELLAKARKPYTITKQRERWTEDEHERFLEALRLYGRAWQRIEEHIGTKTAVQ
IRSHAQKFFTKLEKEAEVKGIPVCQALDIEIPPPRPKRKPNTPYPRKPGNNGTSSSQVSS
AKDAKLVSSASSSQLNQAFLDLEKMPFSEKTSTGKENQDENCSGVSTVNKYPLPTKQVSG
DIETSKTSTVDNAVQDVPKKNKDKDGNDGTTVHSMQNYPWHFHADIVNGNIAKCPQNH
PSGMVSQDFMFHPMREETHGHANLQATTASATTTASHQAFPACHSQDDYRSFLQISSTFSN
LIMSTLLQNPAAHAAATFAASVWPYASVGNSGDSSTPMSSSPPSITAIAAATVAAATAWWA
SHGLLPVCAPAPITCVPFSTVAVPTPAMTEMDTVENTQPFEKQNTALQDQNLASKSPASS
SDDSDETGVTKLNADSKTNDDKIEEVVVTAAVHDSNTAQKKNLVDRSSCGSNTPSGSDAE
TDALDKMEKDKEDVKETDENQPDVIELNNRKIKMRDNNSNNNATTDSWKEVSEEGRIAF
QALFARERLPQSFSPPQVAENVNRKQSDTSMPLAPNFKSQDSCAADQEGVVMIGVGTCKS
LKTRQTGFKPYKRCSMEVKESQVGNINNQSDEKVCKRLRLEGEAST
>AtMYB-2
MNTQRKSKHLKTNPTIVASSSEEVSSLEWEEIAMAQEEEDLICRMYKLVGERWDLIAGRI
PGRTAEEIERFWVMKNHRRSQLR
>AtMYB-3
MVTVNPSQAHCLPMKMSLPGFNTLPHTATTIPVSIRSNRTMSFFEDPTKKVRKPYTITKS
RENWTEQEHDKFLEALHLFDRDWKKIKAFVGSKTVIQIRSHAQKYFLKVQKNGTKEHLP
PPRPKRKANHPYPQKAPKFTLSSSNALFQHDYLYNTNSHPVISTTRKHGLVHCDVSIPSSV
IKEEFGVSENCCSTSSSRDKQRTRIVTETNDQESCGKPHRVAPNFAEVYNFIGSVFDPKT
TGHVKRLKEMDPINLETVLLLMKNLSVNLTSPEFDEQRKLISSYNAS
>AtMYB-4
MALSQWKEAILEGIFMEIEDGIVEEKNLERLENLVEILHKEGSKVPKSVTEAYCKVAVEC
TVKCLAYEKDAKKAYTEAIKTIWLGRIMPLCDKVSCLVTLDLLKCCRRLWKAHTDDKAC
KTLMDEDTRDKALVCLRKVVLDLNPNLVLENLNMDESDEIESSEESEETESMVEAREGVG
NQNSQASEAMEEDDQESLLDTELERPTSGGSKAVYVPSQFNPIPSAVVDRALRKLRASKIE
LMKALEKGRPSNLNNETITEQENDVANPSATNAAPRPSLMEPRSTAHTYEWNDSIDDSDG
EMGDDIERINKSKRKRIVVSPLKRNRCSEGARRPKLPWSTAETLAVLKGYEKYGANWKRI
KDENPVLVRRTNGDIKDKFRVEMRREERRH
>AtMYB-5
MVLQKRPDYGFNGYEVPHTPRAARSPRKSAFKKKSENHQISSFDLLAAVAGKLLLEGGNS
SSSSNNTSGNNEDQCAVKKEPLNGGDIMVEEETTNSDHDNNNAERSFFVSEILQKSHEMQ
SFNRSPSPLKEFHFGSSSGITSDSSEKFETQELAYDESKINNGDCYRSESNDKKSMLGGL
NFEAKLSRNVVGKDEKHIGSGFRKPIPQNPSTCSDDVDLHGKENDDGENFSACYRTKSFR
STLRIGDRRIRKVWASKYCKVPPKLKDTTVTNSDLDLKSDYYSKKHCLKSLRSERNYPIK
KRRYFDGYTASQSEETNKNEGQSGSPRKASAFLSSIACQKQPAAFQSPRDSNNVKLGIKS
FRVPELFIEIPETATVGSLKRTVLEAVTSILGGGLRIGVLVHGKKVRDDSKMLLQTGLSL

DTLSDTLGFCLEPNPPQSTKPLSPEDSDFARPCNVPHTLTRCLPSPGKHAKPSNSVESDL
DSKPSAPNRGKTIYSRALIPVSPLHAQALTVVPPRKTKRSEVAQRRIRRPFSVAEVEALV
QAVERLGTGRWRDVKLRAFDNAKHRTYVDLKDKWKTLVHTARISPQQRRGEPVPQELLD
RVLTAHAYWSQQQGKHQLLEGPQQLETSLGL
>AtMYB-6
MVVVRWASIASYLPQRTDNDIKNYWNTHLKKKLNKSDSDERSRSENIALQTSSTRNTINH
RSTYASSTENISRLLEGWMRASPKSSTSTTFLEHKMQNRTNNFIDHHSDQFPYEQLQGSW
EEGHSKGINGDDDQGIKNSENNNGDDVHHEDGDHEDDDDHNATPPLTFIEKWLLEETSTT
GGQMEEMSHLMELSNML
>AtMYB-7
MVANNNTSSNRRKRIITEGDIATLLLRYDMETILRMLQEISYCSETKMDWNALVKKTTTG
ITNAREYQLLWRHLSYRHPLLPVEDDALPLDDDSDMECELEASPAVSHEASVEAIAHVKV
MAASYVLSESDILDDSTVEAPLTINIPYALPEGSQEPSESPWSSRGMNINFPVCLQKVTS
TEGMNGNGSAGISMAFRRKRKRWSAEEDEELFAAVKRCGEGNWAHIVKGDFRGERTASQ
LSQRWALIRKRCHTSTSVSQCGLQGTEAKLAVNHALSLALGNRPPSNKLAIGTSSRRSFPA
NSSIYVITEDALVWLPLACLNQKLAYLFNCGLMPTTSSCTITETEANGGSSSQGQQQSKP
IVQALPRAGTSLPAAKSRVVKKTTASSTSRSDLMVTANSVAAAACMGDVLTAASGRKVEP
GKTDAPRVPKTKPVKHASTVCMPQPSGSLSMPKVEPGTSVAASIRSLANGKLKPVMASSS
SNKPPLIAPRSEGSSMLSASAPLASLSRIVSNQRVFAGSVPATEIVTCKPDGGQKGQARG
NEASSSAAIQPHQITSRNLEISQGKQATQAQSPNLLPRKVPVVRTAVHCATNQKLMDKPS
DQTVVPIRGAGSQSKAKGEVNSKVGPVIKVSSVCGKPLEVATVAGTGQGV
>AtMYB-8
MEVMDKWVAEFFLRRQLNPRVYAFPLLSALKPVDSDDCVKLKLTAVLRDISNSMIQGTVD
EGMLDLLEILEKLLLQEHSVIMGSLKSAYCWTAVECTLRFMWPVNASDGFFGDALERIWR
NRIGTLKEKESDLVTRELLKWESDLNKAFEEPEIYQKIRETNIRYNAISHLNQLLKEQWA
LLGCSSLESEARKRFLKRKDSPYASRRGGNREKANDVEEVGGVENPDGVGKVNEHEQEH
EPSLNKGEMLVARELKDFLLEIQRLIDPITRQDQEPNNAMEHSVDVTPQPDGANRTDAEDS
EGTSSSRRVRPHLPTPEPLNVSPLKKGRLERPRPRRPMKFWTSEEVAALREGVKEYGKSW
KDIKNSYPVVFADRSEVDLKDKWRNLLGRQ
>AtMYB-9
MFGNVKDYGETFQDSIFQTRNGSLSSTNQIGNPVTYKLVRVAGDGSLVPATDEEMLEVKN
LLEKNEQDMPVLPDPIQTEEYIPDEGSPSQFLQLENFEGKTYCIHAKTDRISLGLHDKGF
FQSETAGPYTENLNSRHESKEELMNGSQMLFVLPDTKFQISTELSGNVELVPSKVLLQEP
ILFSSNGCSINQSTDVLNATASPKEPALSTAASKPDFSRVPGEISLANLSIKELQETFRA
TFGRETTSKDKRWLKRRIKMGLINSCVVPTTTLTINDSKLIGGDQDAIDAFSKGTVDEET
ATESIDTPASPDGIKGHSNDFGHSPVETFVDHYSGNEDFEGEDGSAKRVRKPTRRYIEET
NEKQQIDGSMIPSKDPSSIQAVSSEGRVVVTRMVSLAGSRIQVPYVSHVRRSRPRENIMA
LGEFRSKSWEVKAAPEEGNLNLSPPQLSNDVNRVPGVKSASRCVQKESDKDHLKPIFTDV
DQEMMEPELLDSSGDSSDDNFVDAPITQSASGRKLHRAWTISEVEKLVEGVSKYGVGKW
TEIKKLSFSPYTHRTTVDLKDKWRNLQKASSSNRMEGGLKKHGSMAIPTHIMLQVRELAQ
KQSPISRVVSKARVVKRSRSRNGFL
>AtMYB-10
MGNQKLKWTAEEEEALLAGVRKHGPGKWKNILRDPELAEQLSSRSNIDLKDKWRNLSV
APGIQGSKDKIRTPKIKAAAFHLAAAAAAAIVTPTHSGHSSPVATLPRSGSSDLSIDDSFNI

VVDPKNAPRYDGMIFEALSNLTDANGSDVSAIFNFIEQRQEVPPNFRRMLSSRLRRLAAQ
GKLEKVSHLKSTQNFYKMNDNSLVQRTPHVARPKESNTKSRQQTNSQGPSISQQIVEASI
TAAYKLVEVENKLDVSKGAAEEIERLMKLAEEADEMLVIAREMHEECSQGKIMYLN
>AtMYB-11
MLCFVRFQAGFVRIIVAARKRFRYFLMAAEDRSEELSSNVENGSCNSNEGINPETSSHWI
ENVVKVRKPYTVTKQREKWSEEEHDRFLEAIKLYGRGWRQIQEHIGTKTAVQIRSHAQKF
FSKMAQEADSRSEGSVKAIVIPPPRPKRKPAHPYPRKSPVPYTQSPPPNLSAMEKGTKSP
TSVLSSFGSEDQVNRCSSPNSCTSDIQSIGATSIDKKNNYTTSKQPFKDDSDIGSTPISS
ITLFGKIVLVAEESHKPSSYNDDDLKQMTCQENHYSGMLVDTNLSLGVWETFCTGSNAFG
SVTEASENLEKSAEPISSSWKRLSSLEKQGSCNPVNASGFRPYKRCLSEREVTSSLTLVA
SDEKKSQRARIC
>AtMYB-12
MVRSCSSKSKNPWTNEEDTTQKFVFASASKNGCAAPKKIGLRRCGKSCRVRKTDHSGTK
HESFTSEDEDLIIKMHAAMGSRWQLIAQHLPGKTEEEVKMFWNTKLKKKLSEMGIDHVT
HRPFSHVLAEYGNINGGGNLNPNPSNQAGSLGRNHSLNDDGHQQQPNDSGDLMFHLQAI
KLMTDSSNQVKPESTFVYASSSSSNSSPPLFSSTCSTIAQENSEVNFTWSDFLLDQETFHEN
QQNHPQELDSLFGNDFSEVTAATMANTSTVPSQIEEESLSNGFVESIIAKEKEFFLGFPSY
LEQPFHF
>AtMYB-13
MAAVSSSSETGDCGVTGKRDEIMLFGVRVVVDPMRKCVSLNNLSDYEKSSPEDEIPKIVT
AGAGDGEDKNETDATVIVADGYASANDAVQISSSSGGRKRGVPWTENEHKRFLIGLQKV
GKGDWKGISRNFVKSRTPTQVASHAQKYFLRRTNLNRRRRRSSLFDITTETVTEMAMEQD
PTQENSPLPETNISSGQQAMQVFTDVPTKTENAPETFHLNDPYLVPVTFQAKPTFNLNTDA
APLSLNLCLASSFNLNEQPNSRHSAFTMMPSFSDGDSNSSIIRVA
>AtMYB-14
MASSSMSSSSSWTSKQNKMFERALAVYDKDTPDRWQNVAKAVGSKSAEEVKRHYDILVE
DLMNIEQDLVPLPKYKTVDVGSKSRGIDDFDLRLMKNMRIQ
>AtMYB-15
MGAPKQKWTQEEESALKSGVIKHGPGKWRTILKDPEFSGVLYLRSNVDLKDKWRNMSV
MANGWGSREKSRLAVKRTFSLPKQEENSLALTNSLQSDEENVDATSGLQVSSNPPPRRPN
VRLDSLIMEAIATLKEPGGCNKTTIGAYIEDQYHAPPDFKRLLSTKLKYLTSCGKLVKVKR
KYRIPNSTPLSSHRRKGLGVFGGKQRTSSLPSPKTDIDEVNFQTRSQIDTEIARMKSMNVH
EAAAVAAQAVAEAEAAMAEAEEAAKEAEAAEAEAEAAQAFAEEASKTLKGRNICKMMI
RA
>AtMYB-16
MVDNSNNKKRKEFISEADIATLLQRYDTVTILKLLQEMAYYAEAKMNWNELVKKTSTGIT
SAREYQLLWRHLAYRDSLVPVGNNARVLDDDSDMECELEASPGVSVDVVTEAVAHVKV
MAASYVPSESDIPEDSTVEAPLTINIPYSLHRGPQEPSDSYWSSRGMNITFPVFLPKAAEGH
NGNGLASSLAPRKRRKKWSAEEDEELIAAVKRHGEGSWALISKEEFEGERTASQLSQRWG
AIRRRTDTSNTSTQTGLQRTEAQMAANRALSLAVGNRLPSKKLAVGMTPMLSSGTIKGAQ
ANGASSGSTLQGQQQPQPQIQALSRATTSVPVAKSRVPVKKTTGNSTSRADLMVTANSVA
AAACMSGLATAVTVPKIEPGKNAVSALVPKTEPVKTASTVSMPRPSGISSALNTEPVKTA
VAASLPRSSGIISAPKVEPVKTAASAASLPRPSGMISAPKVEPVKTTASVASLPRPSGII
SAPKAEPVKTAASAASSPRPSGMISAPKVESVKTTASMPRPSGIISAPKAELVKSAASAA

SLPCTSGIISSPKAELVKSAASAASFPRPSSMLSAPKADPVKIVPAAATNTKSVGPLNLR
HAVNGSPNHTIPSSPFTKPLHMAPLSKGSTIQSNSVPPSFASSRLVPTQRAPAATVVTPQ
KPSVVAAATVVTPQKPSVGAAATVVTPQKPSVGAAANVVTPQKPSVGSAATVVTPQKPS
VGAAVTVTSKPVGVQKEQTQGNRASPLVTATLPPNKTIPANSVIGTAKAVAAKVETPPSLM
PKKNEVVGSCTDKSSLDKPPEKESTTTVSPLAVAATKSKPKDEATVTGTGLKEL
>AtMYB-17
MSRSCSQCGNNGHNSRTCPTDITTTGDNNDKGGGEKAIMLFGVRVTEASSSCFRKSVSMN
NLSQFDQTPDPNPTDDGGYASDDVVHASGRNRERKRGTPWTEEEHRLFLTGLHKVGKGD
WRGISRNFVKTRTPTQVASHAQKYFLRRTNQNRRRRRSSLFDITPDSFIGSSKEENQLQTP
LELIRPVPIPIPIPPSRKMADLNLNKKKTPATTEMFPLSLNLQRPSSSTSSSSNEQKARG
SRASSGFEAMSSNGDSIMGVA
>AtMYB-18
MNKTRLRALSPPSGMQHRKRCRLRGRNYVRPEVKQRNFSKDEDDLILKLHALLGNRWSL
IAGRLPGRTDNEVRIHWETYLKRKLVKMGIDPTNHRLHHHTNYISRRHLHSSHKEHETKII
SDQSSSVSESCGVTILPIPSTNCSEDSTSTGRSHLPDLNIGLIPAVTSLPALCLQDSSES
STNGSTGQETLLLFR
>AtMYB-19
MGTVVGTVEDRRDMFEGNAQTRITPFSSTNQIGNPVAYKLVRVSGDGSLVPATDEEILEV
NDTDMHIPSDTCQTIGYLATDEENVEVDETDMHIASDACQTIGYLPAEGIPSRLSQIESS
EAINSGLLHSDNVQPYTDQVKSRSEYNEEMLQKVEQEERLENVHGSQMPSTPADANIQCS
NENNFFEEDQVHHEALLQDECKMNESDMMERCSNAVASPKETALSAAAQKPDFSRVRGE
ICLDNLPIKALQETFRATFGRDTTVKDKTWLKRRIAMGLINSCDVPTTNLRVKDNKLIGNQ
EKSNDVTNAIRKEMGDDVRATKMKDAPSSTDHVNGHSNGGNHYYASEDYSSEQRAAKR
VRKPTRRYIEELSETDDKQQNDKSVIPSKDQRLSEKSEVRSISVSSGKRVTVTRMVSLAGS
EIEVPYVSHVRRSRPRENIMALLGCHSSYLEDKASAAESNLNLSPSQLSSEVVNRDSVEKS
ASRPVQNEFATSDENNVEHILSEVDQEMEPEHIDSSGNSSDENNIGVPIMQGGALRRKHH
RAWTLSEIAKLVEGVSKYGAGKWSEIKKHLFSSHSYRTSVDLKDKWRNLLKTSFAQSPSN
SVGSLKKHGSMHIPTQILLRVRELAEKQSQ
>AtMYB-20
MGNQKLKWTAEEEEALLAGIRKHGPGKWKNILRDPEFADQLIHRSNIDLKDKWRNLSVP
PGTQSLTNKARPAKVKEEGDTPAADANDAVTIPRPIPTIPPPPGRRTLPSELIPDENTKNA
PRYDGVIFEALSALADGNGSDVSSIYHFIEPRHEVPPNFRRILSTRLRRLAAQSKLEKVS
TFKSIQNFYKIPDPSGTKIGVPKPKETHTKLRQANNQTSADSQQMIEEAAITAACKVVEA
ENKIDVAKLAAEEFEKMTKIAEENRKLLVIATEMHELCSCGETMLLA
>AtMYB-21
MADGSTSSSESTTACAGSGTRREIMLFGVRVVLDPMRKCVSLNNLSDYEQTAETPKIDGE
DRDEQDMNKTPAGYASADEALPMSSSNGKIERKRGVPWTEEEHKLFLLGLQRVGKGDW
KGISRNFVKTRTSTQVASHAQKYFLRRSNLNRRRRRSSLFDMTTDTVIPMEEDHQVLIQEN
TSQSSSPVPEINNFSIHPVMQVFPEFPVPTGNQSYGQLTSSNLINLVPLTFQSSPAPLSLN
LSLASSNLNEPSPSMHPAFNTIGVA
>AtMYB-22
MASNSRSSISPWTFSQNKMFERALAVYDKDTPDRWHNVAKAVGGKTVEEVKRHYDILVE
DLINIETGRVPLPNYKTFESNSRSINDFDTRYITKYLYMMLSIYFDNHSSDFEKFSQKVLV
SYISLV

>AtMYB-23
MSSSSNPPVCSPEKEERSEMKIEIQCMENKQPLAASCSSASEGSGCFFLKSPEIATPATV
SSFPRRTSGPMRRAKGGWTPEEDETLRRAVEKYKGKRWKKIAEFFPERTQVQCLHRWQK
VLNPELVKGPWTQEVLLSFSCSETFFGFHFT
>AtMYB-24
MASSSMSTSSWTAREDKQFEMALAKFDKDTPDRWQKIARAVGGKSTEEVKRHYELLLRD
VNDIESGRYPQPRYRNTN
>AtMYB-25
MDNTNRLRLRRGPSLRQTKFTRSRYDSEEVSSIEWEFISMTEQEEDLISRMYRLVGNRWD
LIAGRVVGRKANEIERYWIMRNSDYFSHKRRRLNNSPFFSTSPLNLQENLKL
>AtMYB-26
MDNTNRLRHLRSRKQSKFTLGDTAEVNSVKWEFINMTEQEEDLIFRMHRLVGDRWDLIA
GRVVGREAKDIERYWIMRNCDHCSHKRRRVHKFYRFSISPP
>AtMYB-27
MDNTNRLRRLHCHKQPKFTHSSQEVSSMKWEFINMTEQEEDLIFRMYRLVGDRWDLIAR
RVVGREAKEIERYWIMRNCDYFSHK
>AtMYB-28
MLKQFTHYCEMQAELIPEGPNGEGRLSNQNSNPNLLSSASISITQFPAKKPTRQWAAWTH
QEEESFFTALRQVGKNFEKITSRVQSKNKDQVRHYYYRLVRRMNKLLGPDLSLDAKNPK
DTNAAMLRWWSLLEKYSCKASKLHLKPRRFKLFIEALEHQLLKDRRKSIRKRTCQGENLS
SASLGNISSHSRERGLDNRPFKLILSDGQNVKKLGPGRASTKHGESLSVNLGDEKEDTAFG
RGGRQRRKQAGYRKWEKAAIDGVSLVADAAEHLERTSIDKDMDDQTDLGPTRYLTGKSP
LSLCSAGDVPLSDANMQFSAKLKLQLFPIDECTRRSLEMDKHNPHLELTLSNRKKISSVLE
HLNRKWGSSSCATGELLLFPYNARKETVTCHQRWTHDSFLSAAEVHSMVGSPSVFRLRY
GWFVHDASGSIISQVPTSDPCPSLEDDMNVDRLNEVNMLLTESGPLSVHSTAEQTTSVEPS
QGLVCASGVHDRPARSRDDYEPASTSITPLEHLSGGNAQSPGEWADSLTNISIGDLLSEV
PDDIDSDGVDPPATEGSHYLLRDVPFTSDSFDAAIAAHILRHQNKPSAQLPLTSGSSSLW
DDEETRDAFSFQKNRFANSTELASVASPKGVGRVNGEPSQLVEASSGDEGSYNPHDDGDP
MEEGPADPHTMDSPGKTPCGLADVYWPDSLGPLDLDIRSSKYTDDLILSESLGGLSRLIA
TSLDAFQNCSLFGFDNKKDKSNMV
>AtMYB-29
MAKPENDVTLTEKQTWSTWEELLLACAVHRHGTESWNSVSAEIQKLSPNLCSLTASACRH
KYFDLKSRFTQELPVPESVAEISTAPWLEELRKLRVDELRREVEQYDLSISTLQSKVKQL
EEEREMSFIKPDTETENLDLERKKERSDSGEPVPNPPVQLMNETISPDPKEIGSENTERE
EEMAGSGGGESKLAGEDSCRGSCESVEKEPTTNSERVEPVSVTELIESEDGASRGEEITS
DVQSSASLPRKGTSEPDKEDQSPTSAKDFTVESQPLISFVEILLSHPCGSHFSRRLERQE
TIEYGTIIREHVDFEIIRKRVEGGLYKSWRINFFRDLLLLVNNARVFYHRGSSEFKFAEQ
LHQLVKKQMTTTLKGLSNRDEISISPPKEEVVAIPSSKPVSSKPRMSVPNIVACRKRSAL
AAKPLLLLPPGPDKKAKKTDHVVDYDEKPVSDKDGEASGKDDDDSLIVKIMTRGRTSST
GKVANRNDKNRDSSLNVDDSKDKVKKTDEEKKGGSKKKRAASFLRRMKVGSSDDTLKR
SSAADSSTTGKGGGAEQRKNNSNKADNKKTPIPKIRQTNKKASPVKRSNNGRNSEREAAP
SSSSYPILAKRSRDAGEKEEASSYSPRLKKRARR
>AtMYB-30
MRKEEEEEAGSYNMNVDDTNNNNNNYICTDNDYIEEDEDNSNTKKSQTQAWGTWEELLL

ACAVKRHGFGDWDSVATEVRSRSSLSHLLASANDCRHKYRDLKRRFHEQEKTDVTATVE
EEEEEEERVGNNIPWLEQLRNLRVAELRREVERYDCSILSLQLKVKKLEEEREVGEEKPDL
ENERKEERSENDGSESEHREKAVSAAEESDRENRSMNESNSTATAGEEERVCGDEPSQTRE
DDSGNDKNPDPDPVNKDATAAEEEEGSVSRGSEASHSDELGESGTSESKWKRKRRKQGG
AGEIRSAESKSQPLISLLDLIRSHPRGSLFERRLRSQEAKDYKSMVKQHLDIETIQRKLKQG
SYDSSSLIFYRDLQLLFTNAIVFFPLSSSESMAAHELRAVVSQEMRKETGKAGPRLIKQE
ASGMRSGKADAETSDSSLSRQKSSGPLVVCKKRRSVSAKASPSSSSFSQKDDTKEETLSE
EKDNIATGVRSSRRANKVAAVVANNTKTGKGRNKQKQTESKTNSSNDNSSKQDTGKTEK
KTVSADKKKSVADFLKRLKKNSPQKEAKDQNKSGGNVKKDSKTKPRELRSSSVGKKKA
EVENTPVKRAPGRPQKKTAEATASASGKRGRDTGSTGKDNKQPKKRIRK
>AtMYB-31
MFRSDKAEKMDKRRRRQSKAKASCSEEVSSIEWEAVKMSEEEEDLISRMYKLVGDRWEL
IAGRIPGRTPEEIERYWLMKHGVVFANRRRDFFRK
>AtMYB-32
METNSSGEDLVIKTRKPYTITKQRERWTEEEHNRFIEALRLYGRAWQKIEEHVATKTAVQ
IRSHAQKFFSKVEKEAEAKGVAMGQALDIAIPPPRPKRKPNNPYPRKTGSGTILMSKTGV
NDGKESLGSEKVSHPEMANEDRQQSKPEEKTLQEDNCSDCFTHQYLSAASSMNKSCIETS
NASTFREFLPSREEGSQNNRVRKESNSDLNAKSLENGNEQGPQTYPMHIPVLVPLGSSIT
SSLSHPPSEPDSHPHTVAGDYQSFPNHIMSTLLQTPALYTAATFASSFWPPDSSGGSPVP
GNSPPNLAAMAAATVAAASAWWAANGLLPLCAPLSSGGFTSHPPSTFGPSCDVEYTKAST
LQHGSVQSREQEHSEASKARSSLDSEDVENKSKPVCHEQPSATPESDAKGSDGAGDRKQ
VDRSSCGSNTPSSSDDVEADASERQEDGTNGEVKETNEDTNKPQTSESNARRSRISSNITD
PWKSVSDEGRIAFQALFSREVLPQSFTYREEHREEEQQQQEQRYPMALDLNFTAQLTPVD
DQEEKRNTGFLGIGLDASKLMSRGRTGFKPYKRCSMEAKESRILNNNPIIHVEQKDPKRM
RLETQAST
>AtMYB-33
MGGTDAKDILGLPKTPLSLTQEKKSRPQKESHRKPDGISREVYALTGGVAPLMPSIDLKR
RPPADEKVAWKWLSFTNSARKDDLQLYHWVRVVNDVPPTGDYSFAKYNKSVDILKYTDE
EYENHLTDSVWTKEETDQLFEFCQNFDLRFVVIADRFPVSRTVEELKDRYYSVNRALLRA
RAQSPADVANHPLMKEPYDITRDRERKRALSMVLSQSRHQEKKDAEILAEAKRITEMRLA
ARRAEEPDVSANENAGLDKADGVVPGRSVSPTSNSQLPATAVAPSTLTMADYASTLASLR
MLHVYLRTYGLEQMVQAASSAVGLRTIKRVEQTLQDLGVNLKPKVPTKTVCDEHLELRK
EILTLLNLQKQLQYKESEGSSHREGSYAAMPDTPKDRVFAPDPFSFGAERPIKKEQKRKGP
GRQADTPSPAHKRPRKLKASDL
>AtMYB-34
MSSSPSRNPTNAEAPPPPPTSTDAVAEGSSKKVRKPYTITKSRESWTEEEHDKFLEALQL
FDRDWKKIEDFVGSKTVIQIRSHAQKYFLKVQKNGTLAHVPPPRPKRKAAHPYPQKASK
NAQMPLQVSTSFTTTRNGDMPGYASWDDASMLLNRVISPQHELATLRGAEADIGSKGLL
NVSSPSTSGMGSSSRTVSGSEIVRKAKQPPVLHGVPDFAEVYNFIGSVFDPETRGHVEKLK
EMDPINFETVLLLMRNLTVNLSNPDLESTRKVLLSYDNVTTELPSVVSLVKNSTSDKSA
>AtMYB-35
MVMMIIIYTEPEISLFPLQDRSEELSSNVENGSCNSNEGINPETSSHWIENVVKVRKPYT
VTKQREKWSEEEHDRFLEAIKLYGRGWRQIQEHIGTKTAVQIRSHAQKFFSKMAQEADSR
SEGSVKAIVIPPPRPKRKPAHPYPRKSPVPYTQSPPPNLSAMEKGTKSPTSVLSSFGSED

QNNYTTSKQPFKDDSDIGSTPISSITLFGKIVLVAEESHKPSSYNDDDLKQMTCQENHYS
GMLVDTNLSLGVWETFCTGSNAFGSVTEASENLEKSAEPISSSWKRLSSLEKQGSCNPVN
ASGFRPYKRCLSEREVTSSLTLVASDEKKSQRARIC
>AtMYB-36
MDAAIPIWKRDDDKRFELALVRFPAEGSPDFLENIAQFLQKPLKEVYSYYQALVDDVTLI
ESGKYPLPKYPEDDYVSLPEATKSKTQGTGKKKGIPWSPEEHRLFLDGLNKYGKGDWKSI
SRECVTSRSPMQVASHAQKYFLRQKNKKGKRFSIHDMTLGDAENVTVPVSNLNSMGQQP
HFDDQSPPDHYQDYFSQSNVTIPGCNMHFMGQQPRFGDQIPPGEYHPYSRDNVTVTGSNL
NSIGQQPHFNDQISPDQYGRYLQENFGFFDDDGEDDGSLASFQQLYKA
>AtMYB-37
MASSPRWTEDDNRRFKSALSQFPPDNKRLVNVAQHLPKPLEEVKYYYEKLVNDVYLPKP
LENVTQHLQKPMEMEEMKYMYEKMANDVNQMPEYVPLAESSQSKRRKKDTPNPWTEE
EHRLFLQGLKKYGEGASTLTSTNFVKTKTPRQVSSHAQYYKRQKSDNKKEKRRSIFDITL
ESTEGNPDSGNQNPPDDDDPSQGQGTCLGV
>AtMYB-38
MTRRCSHCSNNGHNSRTCPTRGGGTCGGSGGGGGGGGGGGSGSSSAVKLFGVRLTDGSII
KKSASMGNLSALAVAAAAATHHRLSPSSPLATSNLNDSPLSDHARYSNLHHNEGYLSDDP
AHGSGSSHRRGERKRGVPWTEEEHRLFLVGLQKLGKGDWRGISRNYVTSRTPTQVASHA
QKYFIRHTSSSRRKRRSSLFDMVTDEMVTDSSPTQEEQTLNGSSPSKEPEKKSYLPSLELS
LNNTTEAEEVVATAPRQEKSQEAIEPSNGVSPMLVPGGFFPPCFPVTYTIWLPASLHGTE
HALNAETSSQQHQVLKPKPGFAKERVNMDELVGMSQLSIGMATRHETETSPSPLSLRLEP
SRPSAFHSNGSVNGADLSKGNSAIQAI
>AtMYB-39
MAPSRSKKSKYKKKPRAKAVSPHKDEESMSKTKQRKRKLSDMLGPQWSKEELERFYEG
YRKFGKEWKKVAGFVHSRSAEMVEALYTMNKAYLSLPEGTASVVGLTAMMTDHYSVLH
GGSDSEQENNEGIETPRSAPKRSRVKSSDHPSIGLEGLSDRLQFRSSSGFMPSLKKRRTETM
PRAVGKRTPRIPISYTLEKDTRERYLSPVKRGLNQKGDDTDDDMEHEIALALAEASQRGGS
TKNSHTPNRKAKMYPPDKKGERMRADIDLAIAKLHATDMEDVRCEPSLGSTEADNADYS
GGRNDLTHGEGSSAVEKQQKGRTYYRRRVGIKEEDAKEACSGTDEAPSLGAPDEKFEQER
EGKALKFTYKVSRRKSKKSLFTADEDTACDALHTLADLSLMMPETATDTESSVQAEEKKA
GEAYVSDFKGTDPASMSKSSSLRNSKQRRYGSNDLCNPELERKSPSSSLIQKRRQKALPAK
VRENVLKDELAASSQVIEPCNSKGIGEEYKPVGRGKRSASIRNSHEKKSAKSHDHTSSSNN
IVEEDESAPSNAVIKKQVNLPTKVRSRRKIVTEKPLTIDDGKISETIEKFSHCISSFRAR
RWCIFEWFYSAIDYPWFARQEFVEYLDHVGLGHVPRLTRVEWGVIRSSLGKPRRFSEQFL
KEEKEKLYLYRDSVRKHYDELNTGMREGLPMDLARPLNVSQRVICLHPKSREIHDGNVLT
VDHCRYRIQFDNPELGVEFVKDTECMPLNPLENMPASLARHYAFSNYHIQNPIEEKMHER
AKESMLEGYPKLSCETGHLLSSPNYNISNSLKQEKVDISSSNPQAQDGVDEALALQLFNS
QPSSIGQIQAREADVQALSELTRALDKKELVLRELKCMNDEVVESQKDGHNNALKDSESF
KKQYAAVLFQLSEINEQVSLALLGLRQRNTYQENVPYSSIRRMSKSGEPDGQLTYEDNNA
SDTNGFHVSEIVESSRIKARKMVYRAVQALELLRKDENNNVNMEEAIDFVNNQLSIDQTE
GSSVQQTQGGQDQRLPSTPNPPSSTPANDSHLNQPDQNDLQVPSDLVSRCIATLLMIQKC
TERQFPPSEVAQVLDSAVASLQPCCSQNLPIYTEIQKCMGIIRNQILALVPS
>AtMYB-40
MNYLKPDIKRGNFTKEEEDAIISLHQILGNRWSAIAAKLPGRTDNEIKNVWHTHLKKRLE

DYQPAKPKTSNKKKGTKPKSESVITSSNSTRSESELADSSNPSGESLFSTSPSTSEVSSM
TLISHDGYSNEINMDNKPGDISTIDQECVSFETFGADIDESFWKETLYSQDEHNYVSNDL
EVAGLVEIQQEFQNLGSANNEMIFDSEMDFWFDVLARTGGEQDLLAGL
>AtMYB-41
MGFYSKSIRFEGGGRKIRIGLNRTGKSCRLRWVNYLHPGLKRGKMTPQEERLVLELHAK
WGNRWSKIARKLPGRTDNEIKNYWRTHMRKKAQEKKRPVSPTSSFSNCSSSSVTTTTTNT
QDTSCHSRKSSGEVSFYDTGGSRSTREMNQENEDVYSLDDIWREIDHSAVNIIKPVKDIYS
EQSHCLSYPNLASPSWESSLDSIWNMDADKSKISSYFANDQFPFCFQHSRSPWSSG
>AtMYB-42
MGAPKLKWTPEEETALKAGVLKHGTGKWRTILSDPVYSTILKSRSNVDLKDKWRNISVT
ALWGSRKKAKLALKRTPLSGSRQDDNATAITIVSLANGDVGGQQIDAPSPPAGSCEPPRPS
TSVDKIILEAITSLKRPFGPDGKSILMYIEENFKMQPDMKRLVTSRLKYLTNVGTLVKKK
HKYRISQNYMAEGEGQRSPQLLLEGNKENTPKPEENGVKNLTKSQVGGEVMIMGMTEKE
AAAAAARAVAEAEFAMAEAEEAAREADKAEAEAEAAHIFAKAAMKAVKYRMHSQTR
>AtMYB-43
MVDCGVDIYQLPVVPRACRSPRGRRLKIMRKQRSEFEVLAQVAGRFSGERKNRIVIGSLA
HETKETKDDNVVVNNFLETMEVEVKPQPGLENLSQVLLSKDWLALGPSMPNSPITQEEN
FDSRSKIVKFGIKSLNISELLVDVPESATVGSLKLAVLEAVTQILKGGLNIGVLFQGKTIV
DDSKTLLQIGIPYDDDDDENLGSLGFMLEPQKSETTTITTLTTVSPRTRLRQNQVLGSVD
STEAVAAKSVVPVRMKPAWQPEMVQRRIRRPFTVSEVEALVQAVERLGTGRWRDVKSHA
FNHVNHRTYVDLKDKWKTLVHTAKISARQRRGEPVPQDLLDRVLAAHAFWSDRTG
>AtMYB-44
MEELLLACAVHRHGTDSWDSVASEVHKQNSTFRTLTAIDCRHKYNDLKRRFSRNLVSPGS
ADEETLAAEISSVPWLEELRKLRVDELRREVERYDLSISSLQLKVKTLEDEREKSLKTEN
SDLDRIAETKENHTESGNNSGVPVTELKNSPDPNDNSPGTGSENTNRAVKIAEPVDEEPN
RIGGEDNDEKPAREDSGRGSCESVAKESDRAEPKREGNDSPELVESMDESKGEEDTKETS
DGQSSASFPRKETVDQDQPDNKDQSLTVNKIFVESQPLSDFIEILQSHPIGSHFSRRLET
QETSDYYRIIRQHIDFEMIRSRVEEGYYKTARTKFFRDLLLLINNVRVFYGEPSPEFNAA
KQLYQLIKKQMSFKIPKQTLPPPKEDALVTSKEEVKVSSLKPTLSVPIIACRKRSSLAVR
SPASVTETLKKKTRVVPTVDEKQVSEEEEGRPSDKDEKPIVSKKMARGAAPSTAKKVGSR
NVKTSLNAGISNRGRSPNGSSVLKKSVQQKKGINTSGGSKKQSAASFLKRMKGVSSSETV
VETVKAESSNGKRGAEQRKSNSKSEKVDAVKLPAGQKRLTGKRPTIEKGSPTKKNSGVAS
KRGTASLMAKRDSETSEKETGSSTRPKKRSKR
>AtMYB-45
MKEDEEEEAVISNGMNDDDDTNNINNSSTEFDNNRIGDGDDNSNLNQIKQVWGTWEELV
LTCAVKRHAFSDWDSVAKEVQARSRSSLIVSAVNCRLKYQDLKRRFQDSVDVGDENTEA
AANEEDEVGEISWLEQLRSLHMAELRREVQRCDDSILSLQLKVKKLEEEKDGDDGDNKP
DLKNDETKPVRVNRETTESDRDDNRSMNESNSTASVDKIADHDRLDGDKMVKANENSR
NPDPDPVNKAETPEEEERTVSKRSEMSNSGELDESGTSNCLGKRKGQKYRSGGGGGGVK
SAGDKSQPLIDIIKLIRSHPRGSVFESRLRSQDTKDYKRLIRQHLDMKTIEKKMEKGSYVSS
SLSFYRDLKLLFTNAIVFFPTSSSESIAAQELRTLVSNEMKKRTGKLGHCVIKSEAESSVSR
QKSSVLSLVPCKKKSSALKKTSPSSSSRQKDEKKSQEVSEEKIVTTTATTSARSSRRTSKEI
AVVAKDTKTGRAKNNIKKQTDTKTESSDDDDDEKEENSKTEKKTVADKKKSVADFLKRI
KKNSPQKGKETTSKNQKKNDGNVKKENDHQKKSDGNVKKENSKVKPRELRSSTGKKK

VEVENNNSKSSSKRKQTKETAEVATGKRGRESGKDDKQPRKRSRR
>AtMYB-46
MDNHRRTKQPKTNSIVTSSSEGTEVSSLEWEVVNMSQEEEDLVSRMHKLVGDRWELIAG
RIPGRTAGEIERFWVMKN
>AtMYB-47
MVSVNPRPKGFPVFDSSNMSLPSSDGFGSIPATGRTSTVSFSEDPTTKIRKPYTIKKSRE
NWTDQEHDKFLEALHLFDRDWKKIEAFVGSKTVVQIRSHAQKYFLKVQKSGANEHLPPP
RPKRKASHPYPIKAPKNVAYTSLPSSSTLPLLEPGYLYSSDSKSLMGNQAVCASTSSSWNH
ESTNLPKPVIEVEEPGVSATAPLPNNRCRQEDTERVRAVTKPNNEESCEKPHRVMPNFAE
VYSFIGSVFDPNTSGHLQRLKQMDPINMETVLLLMQNLSVNLTSPEFAEQRRLISSYSAK
ALK
>AtMYB-48
MAAFPQWTRVDDKRFELALLQIPEGSPNFIENIAYYLQKPVKEVEYYYCALVHDIERIES
GKYVLPKYPEDDYVKLTEAGESKGNGKKTGIPWSEEEQRLFLEGLNKFGKGDWKNISRY
CVKSRTSTQVASHAQKYFARQKQESTNTKRPSIHDMTLGVAVNVPGSNLESTGQQPHFGD
QIPSNQYYPSQENFRGFDQRW
>AtMYB-49
MASNSMSSSASWTRKENKLFERALATYDQDTPDRWHNVARAVGGKSAEEVRRHYEPPH
>AtMYB-50
MDLDFDDQPSDHAAPAVRAGARFKPKGRPQPKKKQVSLSTTQTTLSPDVAQEKLSTQSED
LVPLDGSSEIPSNALPSETNVPDSGSINKSTIGTLSEENEDAFPRGVHWSVKPSILRACN
NVNLVGNRRDDGIEATTSFPDDPRTQDSAIFGDYVTPETGADEGRVDMETLDIVQEEGTT
SSYVQHTGKLQPKPRLLETVVEEPEPHYSAGDTGYFPMGTNESEFMANVESRNGFSTYED
LQEEELNIPEAPRETVGEMEAQNASGGWEQEEQGVSPCINNTVTGEEENCMGNTVEEQS
KRESKTGKSKRATSRKRKKTSEEPNKSSEKTEQKKFKHSSRRQKRTLEKELLETPDHEIRS
LPLRDMLRLVEYKEWMQKKEAKGAGVQPSQESNNMNGSGSQYHSQGFDEEDEFGDFGI
ESSEYQENNVVKPDSPVNYQTYMNKTSRTRWSKEDTELFYEGIQEFGSNLSMIQQLFPERT
REQMKLKFKLEERRNPLKLNDALSSRSKHFTHFKNVIKKLQQEAAAAKEGEEEEEAGAE
AETTDVPENEEPEKSEETERASDGVAAGVKESDGGDVENGVRSDGGDECDDDEDFWNSY
KSDIICKRHAKLAMNTFVIFTNSKTRP
>AtMYB-51
MASSSMSSQSSGSWTAKQNKAFEQALATYDQDTPNRWQNVAKVVGGKTTEEVKRHYEL
LVQDINSIENGHVPFPNYRTSGGCTNGRLSQEEKRMRNMRLQ
>AtMYB-52
MTSTNPVVAEVIPAETSTDATETTIATTEAGEAPEKKVRKAYTITKSRESWTEGEHDKFL
EALQLFDRDWKKIEDFVGSKTVIQIRSHAQKYFLKVQKNGTLAHVPPPRPKRKAAHPYPQ
KASKNAQMSLHVSMSFPTQINNLPGYTPWDDDTSALLNIAVSGVIPPEDELDTLCGAEVD
VGSNDMISETSPSASGIGSSSRTLSDSKGLRLAKQAPSMHGLPDFAEVYNFIGSVFDPDS
KGRMKKLKEMDPINFETVLLLMRNLTVNLSNPDFEPTSEYVDAAEEGHEHLSS
>AtMYB-53
MASSPLTANVQGTNASLRNRDEETADKQIQFNDQSFGGNDYAPKVRKPYTITKERERWTD
EEHKKFVEALKLYGRAWRRIEEHVGSKTAVQIRSHAQKFFSKVAREATGGDGSSVEPIVI
PPPRPKRKPAHPYPRKFGNEADQTSRSVSPSERDTQSPTSVLSTVGSEALCSLDSSSPNR
SLSPVSSASPPAALTTTANAPEELETLKLELFPSERLLNRESSIKEPTKQSLKLFGKTVL

VSDSGMSSSLTTSTYCKSPIQPLPRKLSSSKTLPIIRNSQEELLSCWIQVPLKQEDVENR
CLDSGKAVQNEGSSTGSNTGSVDDTGHTEKTTEPETMLCQWEFKPSERSAFSELRRTNSE
SNSRGFGPYKKRKMVTEEEEHEIHLHL
>AtMYB-54
MAMQERCESLCSDELISSSDAFYLKTRKPYTITKQREKWTEAEHEKFVEALKLYGRAWRR
IEEHVGTKTAVQIRSHAQKFFTKVARDFGVSSESIEIPPPRPKRKPMHPYPRKLVIPDAK
EMVYAELTGSKLIQDEDNRSPTSVLSAHGSDGLGSIGSNSPNSSSAELSSHTEESLSLEA
ETKQSLKLFGKTFVVGDYNSSMSCDDSEDGKKKLYSETQSLQCSSSTSENAETEVVVSEF
KRSERSAFSQLKSSVTEMNNMRGFMPYKKRVKVEENIDNVKLSYPLW
>AtMYB-55
MAEEKKNKKKKSDAKVDSEETGEEFISEHSSMKDKEKKRKKNKRENKDGFTGEDMEITG
RESEKLGDEVFIVKKKKKSKKPIRIDSEAVDAVKKKSKKRSKETKADSEAEDDGVEKKSK
EKSKETKVDSEAHDGVKRKKKKSKKESGGDVIENTESSKVSDKKKGKRKRDDTDLGAE
ENIDKEVKRKNNKKKPSVDSDVEDINLDSTNDGKKKRKKKKQSEDSETEENGLNSTKDA
KKRRKKKKKKKQSEVSEAEEKSDKSDEDLTTPSTSSKRVKFSDQVEFFPSDDDEGTEDDD
EEEVKVVRGKRFTKEEDEMVKNAVLEYIDNHALGDEGIKMVMECKAYPQLKGCWKEIT
SALPWRTYNSVYHRAHTIFEAGSQGIWTKEDIELVMEFQKTHGNDWKTLADAMGKHRK
HVKDAWRRGRLAGKKKGHWMREEYQNLFDLVNKDLRMKAFKEKHSKHGMLKDNIPW
MAISDVLETRDHVTCCQKWYEQLISPMVAKGMWANVDDYRLLEELLKLDAACIDDVDW
DNLLENRDGEACRKRWNQMIIHIGVPKSKTFAEQVEILSDRYCPDIAEDREDFDNRPYDPE
D
>AtMYB-56
MDFFDEDRPRFVFQSRPSSSHTAEEEEEARIPNKLFISISVSISLIILSLSFFYFESEPA
KSLLLWLSLSFLVGPFAPSSLTGGKIRVGYGQILEPEQIHDESSTDNERESRRKSVNKRS
KGSTKSDNPPENASAVTEVSRKVVIPQSKESGSVNETKDWTAEEIEILKKQLIKHPAGKP
GRWETVASAFGGRYKTENVIKKAKEIGEKKIYESDDYAQFLKNRKASDPRLVDENEENSG
AGGDAEGTKEIWSNGEDIALLNALKAFPKEAAMRWEKIAAAVPGKSKAACMKRVTELK
KGFRSSKTPAN
>AtMYB-57
MTRRCSHCNHNGHNSRTCPNRGVKLFGVRLTEGSIRKSASMGNLSHYTGSGSGGHGTGS
NTPGSPGDVPDHVAGDGYASEDFVAGSSSSRERKKGTPWTEEEHRMFLLGLQKLGKGDW
RGISRNYVTTRTPTQVASHAQKYFIRQSNVSRRKRRSSLFDMVPDEVGDIPMDLQEPEEDN
IPVETEMQGADSIHQTLAPSSLHAPSILEIEECESMDSTNSTTGEPTATAAAASSSSRLEE
TTQLQSQLQPQPQLPGSFPILYPTYFSPYYPFPFPIWPAGYVPEPPKKEETHEILRPTAV
HSKAPINVDELLGMSKLSLAESNKHGESDQSLSLKLGGGSSSRQSAFHPNPSSDSSDIKS
VIHAL
>AtMYB-58
MVSRNSDGYFLDPTGMTVPGLGPSFTAAVSSSSSPTTSSTAVAVADVTAMVSSSEEDLSK
KIRKPYTITKSRESWTEPEHDKFLEALQLFDRDWKKIEAFIGSKTVIQIRSHAQKYFLKV
QKSGTGEHLPPPRPKRKAAHPYPQKAHKNVQLQVPGSFKSTSEPNDPSFMFRPESSSMLM
TSPTTAAAAPWTNNAQTISFTPLPKAGAGANNNCSSSSENTPRPRSNRDARDHGNVGHSL
RVLPDFAQVYGFIGSVFDPYASNHLQKLKKMDPIDVETVLLLMRNLSINLSSPDFEDHRR
LLSSYDIGSETATDHGGVNKTLNKDPPEIST
>AtMYB-59

MDNTDRRRRRKQHKIALHDSEEVSSIEWEFINMTEQEEDLIFRMYRLVGDRWDLIAGRVP
GRQPEEIERYWIMRNSEGFADKRRQLHSSSHKHTKPHRPRFSIYPS
>AtMYB-60
MGRRCSHCGNVGHNSRTCSSYQTRVVRLFGVHLDTTSSSPPPPPPPSILAAAIKKSFSMD
CLPACSSSSSSFAGYLSDGLAHKTPDRKKGVPWTAEEHRTFLIGLEKLGKGDWRGISRNF
VVTKSPTQVASHAQKYFLRQTTTLHHKRRRTSLFDMVSAGNVEENSTTKRICNDHIGSSS
KVVWKQGLLNPRLGYPDPKVSVSGSGNSGGLDLELKLASIQSPESNIRPISVT
>AtMYB-61
MEVIDQWVAEFFLLRQHNPRASPINLISALKLGDSSDCIKLKISSVLRDISNSLIRGTID
EGMLDLLEILEKLLLQQHSLLMDSHKSAYCWTATECTLRFMWPMFASDGLFTDALERIWT
KRIGILKESGSDLVTCDLLKWESDLKKALGDPELYQRIRETNIRYTAISFLTQLLKEQWA
LLGSSSLESVAQRRFLKRKAVNVEGDVVDNRGDQSDVDESTRRFGSDTIDIANEARGERE
DGNGIGRDNANDGEGMECLENDGIDNVNAADEEHTVSAQDQEHEPSLDKGDEMAAREL
KEYLVEIQGHIDPSTRQGEEPNSAIDHSVDVTPPPTRVNRTGTGCQDHNEASDNVNEKGSD
SQETWSSRVRPRRPTPVTLSVSPLKKGGLAKPHVRRPKKFWKPEEVEALREGVKEYGKS
WKDIKNGNPTVFAERTEVDLKDKWRNLVGG
>AtMYB-62
MEVMRPSTSHVSGGNWLMEETKSGVAASGEGATWTAAENKAFENALAVYDDNTPDRW
QKVAAVIPGKTVSDVIRQYNDLEADVSSIEAGLIPVPGYITSPPFTLDWAGGGGGCNGFKP
GHQVCNKRSQAGRSPELERKKGVPWTEEEHKLFLMGLKKYGKGDWRNISRNFVITRTPT
QVASHAQKYFIRQLSGGKDKRRASIHDITTVNLEEEASLETNKSSIVVGDQRSRLTAFPWN
QTDNNGTQADAFNITIGNAISGVHSYGQVMIGGYNNADSCYDAQNTMFQL
>AtMYB-63
MGFCSESFRFEGGGRNIRIGLNRTGKSCRLRWVNYLHPGLKRGKMTPQEERLVLELHAK
WGNRWSKIARKLPGRTDNEIKNYWRTHMRKKAQEKKRPMSPTSSSSNCCSSSMTTTTSQ
DTGGSNGKMNQECEDGYYSMDDIWREIDQSGANVIKPVKDNYYSEQSCYLNFPPLASPT
WESSLESIWNMDADESKMSSFAIDQFPLSFEHGSGRL
>AtMYB-64
MLHASRGNKWSVIARHLPRRTDNEIKNYWNTHLKKRLMEQGIDPVTHKPLASSSNPTVD
ENLNSPNASSSDKQYSRSSSMPFLSRPPPSSCNMVSKVSELSSNDGTPIQGSSLSCKKRFK
KSSSTSRLLNKVAAKATSIKDILSASMEGSLSATTISHASFFNGFTEQIRNEEDSSNTSL
TNTLAEFDPFSPSSLYPEHEINATSDLNMDQDYDFSQFFEKFGGDNHNEENSMNDLLMSD
VSQEVSSTSVDDQDNMVGNFEGWSNYLLDHTNFMYDTDSDSLEKHFI
>AtMYB-65
MVKETVTVAKTCSHCGHNGHNARTCLNGVNKASVKLFGVNISSDPIRPPEVTALRKSLSL
GNLDALLANDESNGSGDPIAAVDDTGYHSDGQIHSKKGKTAHEKKKGKPWTEEEHRNFL
IGLNKLGKGDWRGIAKSFVSTRTPTQVASHAQKYFIRLNVNDKRKRRASLFDISLEDQKE
KERNSQDASTKTPPKQPITGIQQPVVQGHTQTEISNRFQNLSMEYMPIYQPIPPYYNFPPI
MYHPNYPMYYANPQVPVRFVHPSGIPVPRHIPIGLPLSQPSEASNMTNKDGLDLHIGLPP
QATGASDLTGHGVIHVK
>AtMYB-66
MGAPKQKWTPEEEAALKAGVLKHGTGKWRTILSDTEFSLILKSRSNVDLKDKWRNISVT
ALWGSRKKAKLALKRTPPGTKQDDNNTALTIVALTNDDERAKPTSPGGSGGGSPRTCASK
RSITSLDKIIFEAITNLRELRGSDRTSIFLYIEENFKTPPNMKRHVAVRLKHLSSNGTLVK

IKHKYRFSSNFIPAGARQKAPQLFLEGNNKKDPTKPEENGANSLTKFRVDGELYMIKGMT
AQEAAEAAARAVAEAEFAITEAEQAAKEAERAEAEAEAAQIFAKAAMKALKFRIRNHPW

>AtMYB0
MRIRRRDEKENQEYKKGLWTVEEDNILMDYVLNHGTGQWNRIVRKTGLKRCGKSCRLR
WMNYLSPNVNKGNFTEQEEDLIIRLHKLLGNRWSLIAKRVPGRTDNQVKNYWNTHLSKK
LVGDYSSAVKTTGEDDDSPPSLFITAATPSSCHHQQENIYENIAKSFNGVVSASYEDKPKQE
LAQKDVLMATTNDPSHYYGNNALWVHDDDFELSSLVMMNFASGDVEYCL*
>AtMYB1
MEAEIVRRSEVTGLRREVEESSIGRGDCDGDGGDVGEDAAGFVGTSGRGRRDRVKGPWS
KEEDDVLSELVKRLGARNWSFIARSIPGRSGKSCRLRWCNQLNPNLIRNSFTEVEDQAIIA
AHAIHGNKWAVIAKLLPGRTDNAIKNHWNSALRRRFIDFEKAKNIGTGSLVVDDSGFDRT
TTVASSEETLSSGGGCHVTTPIVSPEGKEATTSMEMSEEQCVEKTNGEGISRQDDKDPPT
LFRPVPRLSSFNACNHMEGSPSPHIQDQNQLQSSKQDAAMLRLLEGAYSERFVPQTCGGG
CCSNNPDGSFQQESLLGPEFVDYLDSPTFPSSELAAIATEIGSLAWLRSGLESSSVRVME
DAVGRLRPQGSRGHRDHYLVSEQGTNITNVLST*
>AtMYB2
MEDYERINSNSPTHEEDSDVRKGPWTEEEDAILVNFVSIHGDARWNHIARSSGLKRTGKS
CRLRWLNYLRPDVRRGNITLEEQFMILKLHSLWGNRWSKIAQYLPGRTDNEIKNYWRTRV
QKQAKHLRCDVNSNLFKETMRNVWMPRLVERINAQSLPTTCEQVESMITDPSQPVNEPSP
VEPGFVQFSQNHHQQFVPATELSATSSNSPAETFSDVRGGVVNGSGYDPSGQTGFGEFND
WGCVGGDNMWTDEESFWFLQDQFCPDTTSYSYN*
>AtMYB3
MGRSPCCEKAHMNKGAWTKEEDQLLVDYIRKHGEGCWRSLPRAAGLQRCGKSCRLRW
MNYLRPDLKRGNFTEEEDELIIKLHSLLGNKWSLIAGRLPGRTDNEIKNYWNTHIKRKLLS
RGIDPNSHRLINESVVSPSSLQNDVVETIHLDFSGPVKPEPVREEIGMVNNCESSGTTSEKD
YGNEEDWVLNLELSVGPSYRYESTRKVSVVDSAESTRRWGSELFGAHESDAVCLCCRIGL
FRNESCRNCRVSDVRTH*
>AtMYB4
MGRSPCCEKAHTNKGAWTKEEDERLVAYIKAHGEGCWRSLPKAAGLLRCGKSCRLRWIN
YLRPDLKRGNFTEEEDELIIKLHSLLGNKWSLIAGRLPGRTDNEIKNYWNTHIRRKLINRG
IDPTSHRPIQESSASQDSKPTQLEPVTSNTINISFTSAPKVETFHESISFPGKSEKISML
TFKEEKDECPVQEKFPDLNLELRISLPDDVDRLQGHGKSTTPRCFKCSLGMINGMECRCG
RMRCDVVGGSSKGSDMSNGFDFLGLAKKETTSLLGFRSLEMK*
>AtMYB5
MMSCGGKKPVSKKTTPCCTKMGMKRGPWTVEEDEILVSFIKKEGEGRWRSLPKRAGLLR
CGKSCRLRWMNYLRPSVKRGGITSDEEDLILRLHRLLGNRWSLIAGRIPGRTDNEIKNYW
NTHLRKKLLRQGIDPQTHKPLDANNIHKPEEEVSGGQKYPLEPISSSHTDDTTVNGGDGD
SKNSINVFGGEHGYEDFGFCYDDKFSSFLNSLINDVGDPFGNIIPISQPLQMDDCKDGIVG
ASSSSLGHD*
>AtMYB6
MGRSPCCEKAHTNKGAWTKEEDQRLVDYIRNHGEGCWRSLPKSAGLLRCGKSCRLRWIN
YLRPDLKRGNFTDDEDQIIIKLHSLLGNKWSLIAGRLPGRTDNEIKNYWNTHIKRKLLSHG
IDPQTHRQINESKTVSSQVVVPIQNDAVEYSFSNLAVKPKTENSSDNGASTSGTTTDEDL

RQNGECYYSDNSGHIKLNLDLTLGFGSWSGRIVGVGSSADSKPWCDPVMEARLSLL*
>AtMYB7
MGRSPCCEKEHMNKGAWTKEEDERLVSYIKSHGEGCWRSLPRAAGLLRCGKSCRLRWIN
YLRPDLKRGNFTHDEDELIIKLHSLLGNKWSLIAARLPGRTDNEIKNYWNTHIKRKLLSKG
IDPATHRGINEAKISDLKKTKDQIVKDVSFVTKFEETDKSGDQKQNKYIRNGLVCKEERV
VVEEKIGPDLNLELRISPPWQNQREISTCTASRFYMENDMECSSETVKCQTENSSSISYS
SIDISSSNVGYDFLGLKTRILDFRSLEMK*
>AtMYB8
MGRSPCCEKAHMNKGAWTKEEDQRLIDYIRNHGEGSWRSLPKSVGLLRCGKSCRLRWIN
YLRPDLKRGNFTDGEEQIIVKLHSLFGNKWSLIAGKLPGRTDNEIKNYWNTHIKRKLLNR
GIDPKTHGSIIEPKTTSFHPRNEDLKSTFPGSVKLKMETSCENCASTSGTTTDEDLRLSVD
CDYRYDHLDKELNLDLTLGYSPTRFVGVGSCY*
>AtMYB9
MGRSPCCDENGLKKGPWTQEEDDKLIDHIQKHGHGSWRALPKQAGLNRCGKSCRLRWT
NYLRPDIKRGNFTEEEEQTIINLHSLLGNKWSSIAGNLPGRTDNEIKNYWNTHLRKKLLQM
GIDPVTHRPRTDHLNVLAALPQLIAAANFNSLLNLNQNVQLDATTLAKAQLLHTMIQVLS
TNNNTTNPSFSSSTMQNSNTNLFGQASYLENQNLFGQSQNFSHILEDENLMVKTQIIDNPL
DSFSSPIQPGFQDDHNSLPLLVPASPEESKETQRMIKNKDIVDYHHHDASNPSSSNSTFT
QDHHHPWCDTIDDGASDSFWKEIIEQTCSEPWPFPE*
>AtMYB10
MGNRRAPCCDKSQVKRGPWSDEESERLRSFILKNGHQNWRSLPKLAGLMRCGKSCRLR
WINYLRPGLKRGNFTKEEEDTIIHLHQAYGNKWSKIASNFPGRTDNEIKNVWNTHLKKRL
VKRSISSSSSDVTNHSVSSTSSSSSSISSVLQDVIIKSERPNQEEEFGEILVEQMACGFEVD
APQSLECLFDDSQVPPPISKPDSLQTHGKSSDHEFWSRLIEPGFDDYNEWLIFLDNQTC*
>AtMYB11
MGRAPCCEKVGIKKGRWTAEEDRTLSDYIQSNGEGSWRSLPKNAGLKRCGKSCRLRWIN
YLRSDIKRGNITPEEEDVIVKLHSTLGTRWSTIASNLPGRTDNEIKNYWNSHLSRKLHGYF
RKPTVANTVENAPPPPKRRPGRTSRSAMKPKFILNPKNHKTPNSFKANKSDIVLPTTTIE
NGEGDKEDALMVLSSSSLSGAEEPGLGPCGYGDDGDCNPSINGDDGALCLNDDIFDSCFL
LDDSHAVHVSSCESNNVKNSEPYGGMSVGHKNIETMADDFVDWDFVWREGQTLWDEK
EDLDSVLSRLLDGEEMESEIRQRDSNDFGEPLDIDEENKMAAWLLS*
>AtMYB12
MGRAPCCEKVGIKRGRWTAEEDQILSNYIQSNGEGSWRSLPKNAGLKRCGKSCRLRWIN
YLRSDLKRGNITPEEEELVVKLHSTLGNRWSLIAGHLPGRTDNEIKNYWNSHLSRKLHNFI
RKPSISQDVSAVIMTNASSAPPPPQAKRRLGRTSRSAMKPKIHRTKTRKTKKTSAPPEPN
ADVAGADKEALMVESSGAEAELGRPCDYYGDDCNKNLMSINGDNGVLTFDDDIIDLLLD
ESDPGHLYTNTTCGGDGELHNIRDSEGARGFSDTWNQGNLDCLLQSCPSVESFLNYDHQ
VNDASTDEFIDWDCVWQEGSDNNLWHEKENPDSMVSWLLDGDDEATIGNSNCENFGEP
LDHDDESALVAWLLS*
>AtMYB13
MGRRPCCEKIGLKKGPWSAEEDRILINYISLHGHPNWRALPKLAGLLRCGKSCRLRWINY
LRPDIKRGNFTPHEEDTIISLHQLLGNRWSAIAAKLPGRTDNEIKNVWHTHLKKRLHHSQ
DQNNKEDFVSTTAAEMPTSPQQQSSSSADISAITTLGNNNDISNSNKDSATSSEDVLAII
DESFWSEVVLMDCDISGNEKNEKKIENWEGSLDRNDKGYNHDMEFWFDHLTSSSCIIGE

MSDISEF*
>AtMYB14
MGRAPCCEKMGVKRGPWTPEEDQILINYIHLYGHSNWRALPKHAGLLRCGKSCRLRWIN
YLRPDIKRGNFTPQEEQTIINLHESLGNRWSAIAAKLPGRTDNEIKNVWHTHLKKRLSKNL
NNGGDTKDVNGINETTNEDKGSVIVDTASLQQFSNSITTFDISNDNKDDIMSYEDISALI
DDSFWSDVISVDNSNKNEKKIEDWEGLIDRNSKKCSYSNSKLYNDDMEFWFDVFTSNRRI
EEFSDIPEF*
>AtMYB15
MGRAPCCEKMGLKRGPWTPEEDQILVSFILNHGHSNWRALPKQAGLLRCGKSCRLRWM
NYLKPDIKRGNFTKEEEDAIISLHQILGNRWSAIAAKLPGRTDNEIKNVWHTHLKKRLEDY
QPAKPKTSNKKKGTKPKSESVITSSNSTRSESELADSSNPSGESLFSTSPSTSEVSSMTLI
SHDGYSNEINMDNKPGDISTIDQECVSFETFGADIDESFWKETLYSQDEHNYVSNDLEVA
GLVEIQQEFQNLGSANNEMIFDSEMDFWFDVLARTGGEQDLLAGL*
>AtMYB16
MGRSPCCDKLGLKKGPWTPEEDQKLLAYIEEHGHGSWRSLPEKAGLHRCGKSCRLRWTN
YLRPDIKRGKFNLQEEQTIIQLHALLGNRWSAIATHLPKRTDNEIKNYWNTHLKKRLVKM
GIDPVTHKPKNETPLSSLGLSKNAAILSHTAQWESARLEAEARLARESKLLHLQHYQTKTS
SQPHHHHGFTHKSLLPNWTTKPHEDQQQLESPTSTVSFSEMKESIPAKIEFVGSSTGVTL
MKEPEHDWINSTMHEFETTQMGEGIEEGFTGLLLGGDSIDRSFSGDKNETAGESSGGDCN
YYEDNKNYLDSIFNFVDPSPSDSPMF*
>AtMYB17
MGRTPCCDKIGLKKGPWTPEEDEVLVAHIKKNGHGSWRTLPKLAGLLRCGKSCRLRWTN
YLRPDIKRGPFTADEEKLVIQLHAILGNRWAAIAAQLPGRTDNEIKNLWNTHLKKRLLSMG
LDPRTHEPLPSYGLAKQAPSSPTTRHMAQWESARVEAEARLSRESMLFSPSFYSGVVKTE
CDHFLRIWNSEIGEAFRNLAPLDESTITSQSPCSRATSTSSALLKSSTNSWGGKEVTVAI
HGSDYSPYSNDLEDDSTDSALQLLLDFPISDDDMSFLEENIDSYSQAPPIGLVSMVSKF*
>AtMYB18
MAKTKYGERHRKGLWSPEEDEKLRSFILSYGHSCWTTVPIKAGLQRNGKSCRLRWINYL
RPGLKRDMISAEEEETILTFHSSLGNKWSQIAKFLPGRTDNEIKNYWHSHLKKKWLKSQSL
QDAKSISPPSSSSSSLVACGKRNPETLISNHVFSFQRLLENKSSSPSQESNGNNSHQCSS
APEIPRLFFSEWLSSSYPHTDYSSEFTDSKHSQAPNVEETLSAYEEMGDVDQFHYNEMMI
NNSNWTLNDIVFGSKCKKQEHHIYREASDCNSSAEFFSPSTTT*
>AtMYB19
MTKSGERPKQRQRKGLWSPEEDQKLKSFILSRGHACWTTVPILAGLQRNGKSCRLRWINY
LRPGLKRGSFSEEEEETILTLHSSLGNKWSRIAKYLPGRTDNEIKNYWHSYLKKRWLKSQ
PQLKSQISDLTESPSSLLSCGKRNLETETLDHVISFQKFSENPTSSPSKESNNNMIMNNS
NNLPKLFFSEWISSSNPHIDYSSAFTDSKHINETQDQINEEEVMMINNNNYSSLEDVMLR
TDFLQPDHEYANYYSSGDFFINSDQNYV*
>AtMYB20
MGRQPCCDKVGLKKGPWTAEEDRKLINFILTNGQCCWRAVPKLSGLLRCGKSCRLRWTN
YLRPDLKRGLLSDYEEKMVIDLHSQLGNRWSKIASHLPGRTDNEIKNHWNTHIKKKLRK
MGIDPLTHKPLSIVEKEDEEPLKKLQNNTVPFQETMERPLENNIKNISRLEESLGDDQFMEI
NLEYGVEDVPLIETESLDLICSNSTMSSSTSTSSHSSNDSSFLKDLQFPEFEWSDYGNSN
NDNNNGVDNIIENNMMSLWEISDFSSLDLLLNDESSSTFGLF*

>AtMYB21
MEKRGGGSSGGSGSSAEAEVRKGPWTMEEDLILINYIANHGDGVWNSLAKSAGLKRTGK
SCRLRWLNYLRPDVRRGNITPEEQLIIMELHAKWGNRWSKIAKHLPGRTDNEIKNFWRTRI
QKYIKQSDVTTTSSVGSHHSSEINDQAASTSSHNVFCTQDQAMETYSPTPTSYQHTNMEF
NYGNYSAAAVTATVDYPVPMTVDDQTGENYWGMDDIWSSMHLLNGN*
>AtMYB22
MGEPQLFDVPVLEGIKNTTNEIMNQLEDDKMKKTYENKKEASTSKYLKKSDITKKRWTE
SEDIKLKEMVALEPKKWTKVAKHFEGRTPKQCRERWHNHARPNVKKTTWSEEEDQILIE
VHKVIGAKWIQISEQLPGRSYNNVKNHWNTTKRRVQNKSGRTVNRVGNNILENYIRSITIN
NDDESDGEPTNIENYHDDSEDMLYGEMNLSPEAITQTTKPLTDASTISPYIPMPKENYTLE
VCESLEDYLELLRWWD*
>AtMYB23
MRMTRDGKEHEYKKGLWTVEEDKILMDYVRTHGQGHWNRIAKKTGLKRCGKSCRLRW
MNYLSPNVNRGNFTDQEEDLIIRLHKLLGNRWSLIAKRVPGRTDNQVKNYWNTHLSKKL
GLGDHSTAVKAACGVESPPSMALITTTSSSHQEISGGKNSTLRFDTLVDESKLKPKSKLVH
ATPTDVEVAATVPNLFDTFWVLEDDFELSSLTMMDFTNGYCL*
>AtMYB24
MEKRESSGGSGSGDAEVRKGPWTMEEDLILINYIANHGEGVWNSLAKSAGLKRTGKSCR
LRWLNYLRPDVRRGNITPEEQLTIMELHAKWGNRWSKIAKHLPGRTDNEIKNFWRTKIQK
YIIKSGETTTVGSQSSEFINHHATTSHVMNDTQETMDMYSPTTSYQHASNINQQLNYGNY
VPESGSIMMPLSVDQSEQNYWSVDDLWPMNIYNGN*
>AtMYB25
MNGEISRPPELISSRNPCKSFENAIHKAVEAELAELAKSDANGGGKSKVKGPWLPEQDEA
LTRLVKMCGPRNWNLISRGIPGRSGKSCRLRWCNQLDPILKRKPFSDEEEHMIMSAQAVL
GNKWSVIAKLLPGRTDNAIKNHWNSNLRRKPAEQWKIPLLMSNTEIVYQLYPSMVRRISN
ASPKEHLPQEEETGVLSDDKMDDEAKEPPREQNSKTGVYRPVARMGAFSVCKPGYMAPC
EGPLVQASRPDSLAGKFLQSLCYDPIIPSKCGHGCCNHQDSTTLSSSSVLGSEFVDYEEHS
SAELDKELISISNDLNNTAWIRSGKEAEQSLKADDQFRREYAHSKFSGMVNNGVSSQMVR
QDLRALS*
>AtMYB26
MGHHSCCNKQKVKRGLWSPEEDEKLINYINSYGHGCWSSVPKHAGTYTHIHGFCLQRCG
KSCRLRWINYLRPDLKRGSFSPQEAALIIELHSILGNRWAQIAKHLPGRTDNEVKNFWNSS
IKKKLMSHHHHGHHHHHLSSMASLLTNLPYHNGFNPTTVDDESSRFMSNIITNTNPNFIT
PSHLSLPSPHVMTPLMFPTSREGDFKFLTTNNPNQSHHHDNNHYNNLDILSPTPTINNHH
QPSLSSCPHDNNLQWPALPDFPASTISGFQETLQDYDDANKLNVFVTPFNDNAKKLLCGE
VLEGKVLSSSSPISQDHGLFLPTTYNFQMTSTSDHQHHHRVDSYINHMIIPSSSSSSPIS
CGQYVIT*
>AtMYB27
MDFKKEETLRRGPWLEEEDERLVKVISLLGERRWDSLAIVSGLKRSGKSCRLRWMNYLN
PTLKRGPMSQEEERIIFQLHALWGNKWSKIARRLPGRTDNEIKNYWRTHYRKKQEAQNY
GKLFEWRGNTGEELLHKYKETEITRTKTTSQEHGFVEVVSMESGKEANGGVGGRESFGV
MKSPYENRISDWISEISTDQSEANLSEDHSSNSCSENNINIGTWWFQETRDFEEFSCSLWS*
>AtMYB28
MSRKPCCVGEGLKKGAWTTEEDKKLISYIHDHGEGGWRDIPQKAGLKRCGKSCRLRWT

NYLKPEIKRGEFSSEEEQIIIMLHASRGNKWSVIARHLPRRTDNEIKNYWNTHLKKRLMEQ
GIDPVTHKPLASSSNPTVDENLNSPNASSSDKQYSRSSSMPFLSRPPPSSCNMVSKVSELS
SNDGTPIQGSSLSCKKRFKKSSSTSRLLNKVAAKATSIKDILSASMEGSLSATTISHASF
FNGFTEQIRNEEDSSNTSLTNTLAEFDPFSPSSLYPEHEINATSDLNMDQDYDFSQFFEK
FGGDNHNEENSMNDLLMSDVSQEVSSTSVDDQDNMVGNFEGWSNYLLDHTNFMYDTD
SDSLEKHFI*
>AtMYB29
MSRKPCCVGEGLKKGAWTAEEDKKLISYIHEHGEGGWRDIPQKAGLKRCGKSCRLRWAN
YLKPDIKRGEFSYEEEQIIIMLHASRGNKWSVIARHLPKRTDNEIKNYWNTHLKKLLIDKG
IDPVTHKPLAYDSNPDEQSQSGSISPKSLPPSSSKNVPEITSSDETPKYDASLSSKKRCF
KRSSSTSKLLNKVAARASSMGTILGASIEGTLISSTPLSSCLNDDFSETSQFQMEEFDPF
YQSSEHIIDHMKEDISINNSEYDFSQFLEQFSNNEGEEADNTGGGYNQDLLMSDVSSTSV
DEDEMMQNITGWSNYLLDHSDFNYDTSQDYDDKNFI*
>AtMYB30
MVRPPCCDKGGVKKGPWTPEEDIILVTYIQEHGPGNWRAVPTNTGLLRCSKSCRLRWTN
YLRPGIKRGNFTEHEEKMIVHLQALLGNRWAAIASYLPQRTDNDIKNYWNTHLKKKLNK
VNQDSHQELDRSSLSSSPSSSSANSNSNISRGQWERRLQTDIHLAKKALSEALSPAVAPIIT
STVTTTSSSAESRRSTSSASGFLRTQETSTTYASSTENIAKLLKGWVKNSPKTQNSADQI
ASTEVKEVIKSDDGKECAGAFQSFSEFDHSYQQAGVSPDHETKPDITGCCSNQSQWSLFE
KWLFEDSGGQIGDILLDENTNFF*
>AtMYB31
MGRPPCCEKIEVKKGPWTPEEDIILVSYIQQHGPGNWRSVPANTGLLRCSKSCRLRWTNY
LRPGIKRGNFTQPEEKMIIHLQALLGNRWAAIASYLPQRTDNDIKNYWNTHLKKKLVMM
KFQNGIINENKTNLATDISSCNNNNNGCNHNKRTTNKGQWEKKLQTDINMAKQALFQAL
SLDQPSSLIPPDPDSPKPHHHSTTTYASSTDNISKLLQNWTSSSSSKPNTSSVSNNRSSSPG
EGGLFDHHSLFSSNSESGSVDEKLNLMSETSMFKGESKPDIDMEATPTTTTTDDQGSLSL
IEKWLFDDQGLVQCDDSQEDLIDVSLEELK*
>AtMYB32
MGRSPCCEKDHTNKGAWTKEEDDKLISYIKAHGEGCWRSLPRSAGLQRCGKSCRLRWIN
YLRPDLKRGNFTLEEDDLIIKLHSLLGNKWSLIATRLPGRTDNEIKNYWNTHVKRKLLRK
GIDPATHRPINETKTSQDSSDSSKTEDPLVKILSFGPQLEKIANFGDERIQKRVEYSVVEE
RCLDLNLELRISPPWQDKLHDERNLRFGRVKYRCSACRFGFGNGKECSCNNVKCQTEDSS
SSSYSSTDISSSIGYDFLGLNNTRVLDFSTLEMK*
>AtMYB33
MSYTSTDSDHNESPAADDNGSDCRSRWDGHALKKGPWSSAEDDILIDYVNKHGEGNWN
AVQKHTSLFRCGKSCRLRWANHLRPNLKKGAFSQEEEQLIVELHAKMGNRWARMAAHL
PGRTDNEIKNYWNTRIKRRQRAGLPLYPPEMHVEALEWSQEYAKSRVMGEDRRHQDFLQ
LGSCESNVFFDTLNFTDMVPGTFDLADMTAYKNMGNCASSPRYENFMTPTIPSSKRLWES
ELLYPGCSSTIKQEFSSPEQFRNTSPQTISKTCSFSVPCDVEHPLYGNRHSPVMIPDSHTPTD
GIVPYSKPLYGAVKLELPSFQYSETTFDQWKKSSSPPHSDLLDPFDTYIQSPPPPTGGEESD
LYSNFDTGLLDMLLLEAKIRNNSTKNNLYRSCASTIPSADLGQVTVSQTKSEEFDNSLKS
FLVHSEMSTQNADETPPRQREKKRKPLLDITRPDVLLASSWLDHGLGIVKETGSMSDALA
VLLGDDIGNDYMNMSVGASSGVGSCSWSNMPPVCQMTELP*
>AtMYB34

MVRTPCCKEEGIKKGAWTPEEDQKLIAYLHLHGEGGWRTLPEKAGLKRCGKSCRLRWAN
YLRPDIKRGEFSPEEDDTIIKLHALKGNKWAAIATSLAGRTDNEIKNYWNTNLKKRLKQK
GIDAITHKPINSTGQTGFEPKVNKPVYSSGSARLLNRVASKYAVELNRDLLTGIISGNSTV
AEDSQNSGDVDSPTSTLLNKMAATSVLINTTTTYSGFSDNCSFTDEFNEFFNNEEISDIY
TTVDNFGFMEELKSILSYGDASAGVIENSPEVNVADAMEFIDSWNEDDNMVGVFV*
>AtMYB35
MGRPPCCDKSNVKKGLWTEEEDAKILAYVAIHGVGNWSLIPKKAGLNRCGKSCRLRWTN
YLRPDLKHDSFSTQEEELIIECHRAIGSRWSSIARKLPGRTDNDVKNHWNTKLKKKLMKM
GIDPVTHKPVSQLLAEFRNISGHGNASFKTEPSNNSILTQSNSAWEMMRNTTTNHESYYTN
SPMMFTNSSEYQTTPFHFYSHPNHLLNGTTSSCSSSSSSTSITQPNQVPQTPVTNFYWSD
FLLSDPVPQVVGSSATSDLTFTQNEHHFNIEAEYISQNIDSKASGTCHSASSFVDEILDK
DQEMLSQFPQLLNDFDY*
>AtMYB36
MGRAPCCDKANVKKGPWSPEEDVKLKDYIDKYGTGGNWIALPQKIGLKRCGKSCRLRW
LNYLRPNIKHGGFSEEEDRIILSLYISIGSRWSIIAAQLPGRTDNDIKNYWNTKLKKKLLGR
QKQMNRQDSITDSTENNLSNNNNNKSPQNLSNSALERLQLHMQLQNLQSPFSSFYNNPIL
WPKLHPLLQSTTTNQNPKLASQESFHPLGVNVDHQHNNTKLAQINNGASSLYSENVEQSQ
NPAHEFQPNFGFSQDLRLDNHNMDFMNRGVSKELFQVGNEFELTNGSSWWSEEVELERK
TTSSSSWGSASVLDQTTEGMVMLQDYAQMSYHSV*
>AtMYB37
MGRAPCCDKTKVKRGPWSPEEDSKLRDYIEKYGNGGNWISFPLKAGLRRCGKSCRLRWL
NYLRPNIKHGDFSEEEDRIIFSLFAAIGSRWSIIAAHLPGRTDNDIKNYWNTKLRKKLLSS
SSDSSSSAMASPYLNPISQDVKRPTSPTTIPSSSYNPYAENPNQYPTKSLISSINGFEAG
DKQIISYINPNYPQDLYLSDSNNNTSNANGFLLNHNMCDQYKNHTSFSSDVNGIRSEIMM
KQEEIMMMMMIDHHIDQRTKGYNGEFTQGYYNYYNGHGDLKQMISGTGTNSNINMGGS
GSSSSSISNLAENKSSGSLLLEYKCLPYFYS*
>AtMYB38
MSLSLIITEPENSFSFPFFSFKKQNKDISTKTLRPRETDREMGRAPCCDKANVKRGPWSP
EEDAKLKDYIEKQGTGGNWIALPHKAGLRRCGKSCRLRWLNYLRPNIRHGDFTEEEDNII
YSLFASIGSRWSVIAAHLQGRTDNDIKNYWNTKLKKKLIATMAPPPHHHLAIATSSSSAS
PSSSSHYNMINSLLPYNPSTNQLLTPHQGIMMTMMGQQQQLFYQEDMGNLVNSPNRNNLI
MSHQEDNQEQSTNKGIMLLSDVRSGSSTTSTVTRVKMEHRDHDDHHHHHEEDERSMTSV
VMEDYGMEEIKQLISSSCTSSNNSLWFDENKTEDKFMLYY*
>AtMYB39
MGRSPCCDQDKGVKKGPWLPEEDDKLTAYINENGYGNWRSLPKLAGLNRCGKSCRLRW
MNYLRPDIRRGKFSDGEESTIVRLHALLGNKWSKIAGHLPGRTDNEIKNYWNTHMRKKL
LQMGIDPVTHEPRTNDLSPILDVSQMLAAAINNGQFGNNNLLNNNTALEDILKLQLIHKM
LQIITPKAIPNISSFKTNLLNPKPEPVVNSFNTNSVNPKPDPPAGLFINQSGITPEAASDFIP
SYENVWDGFEDNQLPGLVTVSQESLNTAKPGTSTTTKVNDHIRTGMMPCYYGDQLLETP
STGSVSVSPETTSLNHPSTAQHSSGSDFLEDWEKFLDDETSDSCWKSFLDLTSPTSSPVPW*
>AtMYB40
MCDLLACLRKISMGRKPCCDKIGLKRGPWTIEEDHRLMNFILNNGIHCWRIVPKLAGLLR
CGKSCRLRWINYLRPDLKRGGFTDAEEDRIMELHSQLGNRWSKIASHFSGRTDNEIKNHW
NTKIKKKMKHLGLDPATHKPMNDITHQTDPNQDKKPNMCSTINEGEEIKDQTPKDDVITE

TTKTLMLSDNDEELVAKNCKILCAEEVDLESLFETQCNEISSSSFSSLCSNISRSESSSY
LAEDSISLEQWDLDMTDPFVPWDLFANLDDNLFLL*
>AtMYB41
MGRSPCCDKNGVKKGPWTAEEDQKLIDYIRFHGPGNWRTLPKNAGLHRCGKSCRLRWT
NYLRPDIKRGRFSFEEEETIIQLHSVMGNKWSAIAARLPGRTDNEIKNHWNTHIRKRLVRS
GIDPVTHSPRLDLLDLSSLLSALFNQPNFSAVATHASSLLNPDVLRLASLLLPLQNPNPVY
PSNLDQNLQTPNTSSESSQPQAETSTVPTNYETSSLEPMNARLDDVGLADVLPPLSESFD
LDSLMSTPMSSPRQNSIEAETNSSTFFDFGIPEDFILDDFMF*
>AtMYB42
MGRQPCCDKLMVKKGPWTAEEDKKLINFILTNGHCCWRALPKLAGLRRCGKSCRLRWT
NYLRPDLKRGLLSDAEEQLVIDLHALLGNRWSKIAARLPGRTDNEIKNHWNTHIKKKLLK
MEIDPSTHQPLNKVFTDTNLVDKSETSSKADNVNDNKIVEIDGTTTNTIDDSIITHQNSSND
DYELLGDIIHNYGDLFNILWTNDEPPLVDDASWSNHNVGIGGTAAVAASDKNNTAAEEDF
PERSFEKQNGESWMFLDYCQEFGVEDFGFECYHGFGQSSMKTGHKD*
>AtMYB43
MGRQPCCDKVGLKKGPWTIEEDKKLINFILTNGHCCWRALPKLSGLLRCGKSCRLRWINY
LRPDLKRGLLSEYEEQKVINLHAQLGNRWSKIASHLPGRTDNEIKNHWNTHIKKKLRKM
GIDPLTHKPLSEQEASQQAQGRKKSLVPHDDKNPKQDQQTKDEQEQHQLEQALEKNNTS
VSGDGFCIDEVPLLNPHEILIDISSSHHHHSNDDNVNINTSKFTSPSSSSSSTSSCISSVVP
GDEFSKFFDEMEILDLKWLSSDDSLGDDISKDGKFNNSTVDTMNLWDINDLSSLDMFMN
EHDDGFIGNGNGCSRMVLDQDSWTFDLL*
>AtMYB44
MADRIKGPWSPEEDEQLRRLVVKYGPRNWTVISKSIPGRSGKSCRLRWCNQLSPQVEHRP
FSAEEDETIARAHAQFGNKWATIARLLNGRTDNAVKNHWNSTLKRKCGGYDHRGYDGSE
DHRPVKRSVSAGSPPVVTGLYMSPGSPTGSDVSDSSTIPILPSVELFKPVPRPGAVVLPLP
IETSSSSDDPPTSLSLSLPGADVSEESNRSHESTNINNTTSSRHNHNNTVSFMPFSGGFR
GAIEEMGKSFPGNGGEFMAVVQEMIKAEVRSYMTEMQRNNGGGFVGGFIDNGMIPMSQI
GVGRIE*
>AtMYB45
MVFKSEKSNREMKSKEKQRKGLWSPEEDEKLRSHVLKYGHGCWSTIPLQAGLQRNGKS
CRLRWVNYLRPGLKKSLFTKQEETILLSLHSMLGNKWSQISKFLPGRTDNEIKNYWHSNL
KKGVTLKQHETTKKHQTPLITNSLEALQSSTERSSSSINVGETSNAQTSSFSPNLVFSEWLD
HSLLMDQSPQKSSYVQNLVLPEERGFIGPCGPRYLGNDSLPDFVPNSEFLLDDEISSEIE
FCTSFSDNFLFDGLINELRPM*
>AtMYB46
MRKPEVAIAASTHQVKKMKKGLWSPEEDSKLMQYMLSNGQGCWSDVAKNAGLQRCGK
SCRLRWINYLRPDLKRGAFSPQEEDLIIRFHSILGNRWSQIAARLPGRTDNEIKNFWNSTIK
KRLKKMSDTSNLINNSSSSPNTASDSSSNSASSLDIKDIIGSFMSLQEQGFVNPSLTHIQT
NNPFPTGNMISHPCNDDFTPYVDGIYGVNAGVQGELYFPPLECEEGDWYNANINNHLDEL
NTNGSGNAPEGMRPVEEFWDLDQLMNTEVPSFYFNFKQSI*
>AtMYB47
MGRTTWFDVDGMKKGEWTAEEDQKLGAYINEHGVCDWRSLPKRAGLQRCGKSCRLRW
LNYLKPGIRRGKFTPQEEEEIIQLHAVLGNRWAAMAKKMQNRTDNDIKNHWNSCLKKRL
SRKGIDPMTHEPIIKHLTVNTTNADCGNSSTTTSPSTTESSPSSGSSRLLNKLAAGISSRQHS

LDRIKYILSNSIIESSDQAKEEEEKEEEEEERDSMMGQKIDGSEGEDIQIWGEEEVRRLME
IDAMDMYEMTSYDAVMYESSHILDHLF*
>AtMYB48
MKMMQEEGNRKGPWTEQEDILLVNFVHLFGDRRWDFIAKVSGLNRTGKSCRLRWVNYL
HPGLKRGKMTPQEERLVLELHAKWGNRWSKIARKLPGRTDNEIKNYWRTHMRKKAQEK
KRPVSPTSSFSNCSSSSVTTTTTNTQDTSCHSRKSSGEVSFYDTGGSRSTREMNQENEDVY
SLDDIWREIDHSAVNIIKPVKDIYSEQSHCLSYPNLASPSWESSLDSIWNMDADKSKISSYF
ANDQFPFCFQHSRSPWSSG*
>AtMYB49
MGKSSSSEESEVKKGPWTPEEDEKLVGYIQTHGPGKWRTLPKNAGLKRCGKSCRLRWTN
YLRPDIKRGEFSLQEEETIIQLHRLLGNKWSAIAIHLPGRTDNEIKNYWNTHIKKKLLRMG
IDPVTHCPRINLLQLSSFLTSSLFKSMSQPMNTPFDLTTSNINPDILNHLTASLNNVQTE
SYQPNQQLQNDLNTDQTTFTGLLNSTPPVQWQNNGEYLGDYHSYTGTGDPSNNKVPQA
GNYSSAAFVSDHINDGENFKAGWNFSSSMLAGTSSSSSTPLNSSSTFYVNGGSEDDRESFG
SDMLMFHHHHDHNNNALNLS*
>AtMYB50
MKRHSCCYKQKLRKGLWSPEEDEKLLNYITKHGHGCWSSVPKLAGLERCGKSCRLRWIN
YLRPDLKRGAFSSEEQNLIVELHAVLGNRWSQIAARLPGRTDNEIKNLWNSCIKKKLMKK
GIDPITHKPLSEVGKETNRSDNNNSTSFSSETNQDLFVKKTSDFAEYSAFQKEESNSVSLR
NSLSSMIPTQFNIDDGSVSNAGFDTQVCVKPSIILLPPPNNTSSTVSGQDHVNVSEPNWE
SNSGTTSHLNNPGMEEMKWSEEYLNESLFSTQVYVKSETDFNSNIAFPWSQSQACDVFPK
DLQRMAFSFGGQTL*
>AtMYB51
MVRTPCCKAELGLKKGAWTPEEDQKLLSYLNRHGEGGWRTLPEKAGLKRCGKSCRLRW
ANYLRPDIKRGEFTEDEERSIISLHALHGNKWSAIARGLPGRTDNEIKNYWNTHIKKRLIK
KGIDPVTHKGITSGTDKSENLPEKQNVNLTTSDHDLDNDKAKKNNKNFGLSSASFLNKVA
NRFGKRINQSVLSEIIGSGGPLASTSHTTNTTTTSVSVDSESVKSTSSSFAPTSNLLCHGT
VATTPVSSNFDVDGNVNLTCSSSTFSDSSVNNPLMYCDNFVGNNNVDDEDTIGFSTFLND
EDFMMLEESCVENTAFMKELTRFLHEDENDVVDVTPVYERQDLFDEIDNYFG*
>AtMYB52
MMCSRGHWRPAEDEKLRELVEQFGPHNWNAIAQKLSGRSGKSCRLRWFNQLDPRINRNP
FTEEEEERLLASHRIHGNRWSVIARFFPGRTDNAVKNHWHVIMARRGRERSKLRPRGLGH
DGTVAATGMIGNYKDCDKERRLATTTAINFPYQFSHINHFQVLKEFLTGKIGFRNSTTPIQ
EGAIDQTKRPMEFYNFLQVNTDSKIHELIDNSRKDEEEDVDQNNRIPNENCVPFFDFLSV
GNSASQGLC*
>AtMYB53
MGRSPSSDETGLKKGPWLPEEDDKLINYIHKHGHSSWSALPKLAGLNRCGKSCRLRWTN
YLRPDIKRGKFSAEEEETILNLHAVLGNKWSMIASHLPGRTDNEIKNFWNTHLKKKLIQM
GFDPMTHQPRTDDIFSSLSQLMSLSNLRGLVDLQQQFPMEDQALLNLQTEMAKLQLFQYL
LQPSPAPMSINNINPNILNLLIKENSVTSNIDLGFLSSHLQDFNNNNLPSLKTLDDNHFSQ
NTSPIWLHEPPSLNQTMLPTHDPCAQSVDGFGSNQASSSHDQEVAVTDSVDWPDHHLFDD
SMFPDISYQS*
>AtMYB54
MIMCSRGHWRPAEDEKLKDLVEQYGPHNWNAIALKLPGRSGKSCRLRWFNQLDPRINRN

PFTEEEEEERLLAAHRIHGNRWSIIARLFPGRTDNAVKNHWHVIMARRTRQTSKPRLLPSTT
SSSSLMASEQIMMSSGGYNHNYSSDDRKKIFPADFINFPYKFSHINHLHFLKEFFTGKIA
LNHKANQSKKPMEFYNFLQVNTDSNKSEIIDQDSGQSKRSDSDTKHESHVPFFDFLSVGN
SAS*
>AtMYB55
MGRHSCCYKQKLRKGLWSPEEDEKLLRYITKYGHGCWSSVPKQAGTFLFIQIHLLFGLQR
CGKSCRLRWINYLRPDLKRGAFSQDEENLIIELHAVLGNRWSQIAAQLPGRTDNEIKNLW
NSCLKKKLRLRGIDPVTHKLLTEIETGTDDKTKPVEKSQQTYLVETDGSSSTTTCSTNQN
NNTDHLYTGNFGFQRLSLENGSRIAAGSDLGIWIPQTGRNHHHHVDETIPSAVVLPGSMF
SSGLTGYRSSNLGLIELENSFSTGPMMTEHQQIQESNYNNSTFFGNGNLNWGLTMEENQN
PFTISNHSNSSLYSDIKSETNFFGTEATNVGMWPCNQLQPQQHAYGHI*
>AtMYB56
MNPNLLEKDLRGKETTNGSIRYKEANNFRSLPNSHTAACKTSLNNPSISRNHPHNKSASV
LESEDEHGNERGENEKSLRMRGKSGINTKVCSRGHWRPTEDAKLKELVAQFGPQNWNLI
SNHLLGRSGKSCRLRWFNQLDPRINKRAFTEEEEFRLLAAHRAYGNKWALISRLFPGRTD
NAVKNHWHVIMARRTRESQRQRQQPPPTLSRDAEMTVSSSCRYNQGKFINEEDDDDDVS
AVSTCTTELSLTPPSSAYQPRFFNYDSTLASGKDGQCVQRAEVNGIYGKKMDHQNHHTIS
VSERKVEMKMRSGYYYFDFLGVGAS*
>AtMYB57
METTMKKKGRVKATITSQKEEEGTVRKGPWTMEEDFILFNYILNHGEGLWNSVAKASGL
KRTGKSCRLRWLNYLRPDVRRGNITEEEQLLIIQLHAKLGNRWSKIAKHLPGRTDNEIKNF
WRTKIQRHMKVSSENMMNHQHHCSGNSQSSGMTTQGSSGKAIDTAESFSQAKTTTFNVV
EQQSNENYWNVEDLWPVHLLNGDHHVI*
>AtMYB58
MGKGRAPCCDKTKVKRGPWSHDEDLKLISFIHKNGHENWRSLPKQAGLLRCGKSCRLR
WINYLRPDVKRGNFSAEEEDTIIKLHQSFGNKWSKIASKLPGRTDNEIKNVWHTHLKKRL
SSETNLNADEAGSKGSLNEEENSQESSPNASMSFAGSNISSKDDDAQISQMFEHILTYSEFT
GMLQEVDKPELLEMPFDLDPDIWSFIDGSDSFQQPENRALQESEEDEVDKWFKHLESELG
LEENDNQQQQQQHKQGTEDEHSSSLLESYELLIH*
>AtMYB59
MKLVQEEYRKGPWTEQEDILLVNFVHLFGDRRWDFVAKVSGLNRTGKSCRLRWVNYLH
PGLKRGKMTPQEERLVLELHAKWGNRWSKIARKLPGRTDNEIKNYWRTHMRKKAQEKK
RPMSPTSSSSNCCSSSMTTTTSQDTGGSNGKMNQECEDGYYSMDDIWREIDQSGANVIKP
VKDNYYSEQSCYLNFPPLASPTWESSLESIWNMDADESKMSSFAIDQFPLSFEHGSGRL*
>AtMYB60
MGRPPCCDKIGIKKGPWTPEEDIILVSYIQEHGPGNWRSVPTNTGLLRCSKSCRLRWTNY
LRPGIKRGNFTPHEEGMIIHLQALLGNKWASIASYLPQRTDNDIKNYWNTHLKKKLNKSD
SDERSRSENIALQTSSTRNTINHRSTYASSTENISRLLEGWMRASPKSSTSTTFLEHKMQ
NRTNNFIDHHSDQFPYEQLQGSWEEGHSKGINGDDDQGIKNSENNNGDDVHHEDGDHED
DDDHNATPPLTFIEKWLLEETSTTGGQMEEMSHLMELSNML*
>AtMYB61
MGRHSCCYKQKLRKGLWSPEEDEKLLTHITNHGHGCWSSVPKLAGLQRCGKSCRLRWIN
YLRPDLKRGAFSPEEENLIVELHAVLGNRWSQIASRLPGRTDNEIKNLWNSSIKKKLKQRG
IDPNTHKPISEVESFSDKDKPTTSNNKRSGNDHKSPSSSSATNQDFFLERPSDLSDYFGF

QKLNFNSNLGLSVTTDSSLCSMIPPQFSPGNMVGSVLQTPVCVKPSISLPPDNNSSSPIS
GGDHVKLAAPNWEFQTNNNNTSNFFDNGGFSWSIPNSSTSSSQVKPNHNFEEIKWSEYLN
TPFFIGSTVQSQTSQPIYIKSETDYLANVSNMTDPWSQNENLGTTETSDVFSKDLQRMAV
SFGQSL*
>AtMYB62
MENSMKKKKSFKESEDEELRRGPWTLEEDTLLTNYILHNGEGRWNHVAKCAGLKRTGKS
CRLRWLNYLKPDIRRGNLTPQEQLLILELHSKWGNRWSKIAQYLPGRTDNEIKNYWRTRV
QKQARQLNIESNSDKFFDAVRSFWVPRLIEKMEQNSSTTTTYCCPQNNNNNSLLLPSQSH
DSLSMQKDIDYSGFSNIDGSSSTSTCMSHLTTVPHFMDQSNTNIIDGSMCFHEGNVQEFGG
YVPGMEDYMVNSDISMECHVADGYSAYEDVTQDPMWNVDDIWQFRE*
>AtMYB63
MGKGRAPCCDKTKVKRGPWSPEEDIKLISFIQKFGHENWRSLPKQSGMSLLLSSQSKQKP
LQLFFLFFMILNVYICKNEGLLRCGKSCRLRWINYLRPDLKRGNFTSEEEETIIKLHHNY
GNKWSKIASQLPGRTDNEIKNVWHTHLKKRLAQSSGTADEPASPCSSDSVSRGKDDKSSH
VEDSLNRETNHRNELSTSMSSGGSNQQDDPKIDELRFEYIEEAYSEFNDIIIQEVDKPDL
LEIPFDSDPDIWSFLDTSNSFQQSTANENSSGSRATTEEESDEDEVKKWFKHLESELGLE
EDDNQQQYKEEESSSSSLLKNYELMIH*
>AtMYB64
MEEQKIQEKSLAHGAAPPLTAVERFLNGQKNEALCFKKQERSIDRPIVKTTRAIEIRNEN
KENMMFGPRKEKNLAVIGEIVVKGAAKDYTCKDITKKQPYKNIIKGQWTADEDRKLIKLV
MQHGERKWAVISEKLEGRAGKQCRERWHNHLRPDIKKDSWSEEEERLLVEAHTRIGNKW
AEIAKLIQGRTENSIKNHWNATKRRQNSKRKHKRSKNADSNSDIDDLSPSAKRPRILEDYI
KNIENNDKNNGENIMTTSGNNVLSTSNYDQFNSEDSTSSLLDDPYDEELVFLKNIFENHS
LENINLSQGTEITQSSSSGFMIENPKPKPNLYNNTFGTHLGAMVTEPANSSHLASDIYLS
DLLNGTASSSSSLTFLSSNNNEHAGENELLLPQANSTSERREMDLIEMLSGSTQGSNIWF
PLF*
>AtMYB65
MSYTTATADSDDGMHSSIHNESPAPDSISNGCRSRGKRSVLKKGPWTSTEDGILIDYVKK
HGEGNWNAVQKHTSLARCGKSCRLRWANHLRPNLKKGAFSQEEEQLIVEMHAKMGNK
WAQMAEHLPGRTDNEIKNYWNTRIKRRQRAGLPLYPPEIYVDDLHWSEEYTKSNIIRVDR
RRRHQDFLQLGNSKDNVLFDDLNFAASLLPAASDLSDLVACNMLGTGASSSRYESYMPPI
LPSPKQIWESGSRFPMCSSNIKHEFQSPEHFQNTAVQKNPRSCSISPCDVDHHPYENQHSSH
MMMVPDSHTVTYGMHPTSKPLFGAVKLELPSFQYSETSAFDQWKTTPSPPHSDLLDSVD
AYIQSPPPSQVEESDCFSSCDTGLLDMLLHEAKIKTSAKHSLLMSSPQKSFSSTTCTTNVTQ
NVPRGSENLIKSGEYEDSQKYLGRSEITSPSQLSAGGFSSAFAGNVVKTEELDQVWEPKR
VDITRPDVLLASSWLDQGCYGIVSDTSSMSDALALLGGDDIGNSYVTVGSSSGQAPRGVG
SYGWTNMPPVWSL*
>AtMYB66
MRKKVSSSGDEGNNEYKKGLWTVEEDKILMDYVKAHGKGHWNRIAKKTGLKRCGKSC
RLRWMNYLSPNVKRGNFTEQEEDLIIRLHKLLGNRWSLIAKRVPGRTDNQVKNYWNTHL
SKKLGIKDQKTKQSNGDIVYQINLPNPTETSEETKISNIVDNNNILGDEIQEDHQGSNYLSS
LWVHEDEFELSTLTNMMDFIDGHCF*
>AtMYB67
MREKWEMKRDEMGHRCCGKHKVKRGLWSPEEDEKLLRYITTHGHPSWSSVPKLAGLQR

CGKSCRLRWINYLRPDLRRGSFNEEEEQIIIDVHRILGNKWAQIAKHLPGRTDNEVKNFWN
SCIKKKLLSQGLDPSTHNLMPSHKRSSSSNNNNIPKPNKTTSIMKNPTDLDQSTTAFSITN
INPPTSTKPNKLKSPNQTTIPSQTVIPINDNMSSTQTMIPINDPMSSLLDDENMIPHWSD
VDGMAIHEAPMLPSDKAVVGVDDDDLNMDILFNTPSSSAFDPDFASIFSSAMSIDFNPMD
DLGSWTF*
>AtMYB68
MGRAPCCDKANVKKGPWSPEEDAKLKDYIENSGTGGNWIALPQKIGLRRCGKSCRLRW
LNYLRPNIKHGGFSEEEDNIICNLYVTIGSRWSIIAAQLPGRTDNDIKNYWNTRLKKKLLN
KQRKEFQEARMKQEMVMMKRQQQGQGQGQSNGSTDLYLNNMFGSSPWPLLPQLPPPH
HQIPLGMMEPTSCNYYQTTPSCNLEQKPLITLKNMVKIEEEQERTNPDHHHQDSVTNPFD
FSFSQLLLDPNYYLGSGGGGEGDFAIMSSSTNSPLPNTSSDQHPSQQQEILQWFGSSNFQTE
AINDMFINNNNNIVNLETIENTKVYGDASVAGAAVRAALGGGTTSTSADQSTISWEDITSL
VNSEDASYFNAPNHV*
>AtMYB69
MEMSRGSNSFDNKKPSCQRGHWRPVEDDNLRQLVEQYGPKNWNFIAQHLYGRSGKSCR
LRWYNQLDPNITKKPFTEEEEERLLKAHRIQGNRWASIARLFPGRTDNAVKNHFHVIMAR
RKRENFSSTATSTFNQTWHTVLSPSSSLTRLNRSHFGLWRYRKDKSCGLWPYSFVSPPTNG
QFGSSSVSNVHHEIYLERRKSKELVDPQNYTFHAATPDHKMTSNEDGPSMGDDGEKNDV
TFIDFLGVGLAS*
>AtMYB70
MSGSTRKEMDRIKGPWSPEEDDLLQSLVQKHGPRNWSLISKSIPGRSGKSCRLRWCNQLS
PEVEHRGFTAEEDDTIILAHARFGNKWATIARLLNGRTDNAIKNHWNSTLKRKCSGGGGG
GEEGQSCDFGGNGGYDGNLTDEKPLKRRASGGGGVVVVTALSPTGSDVSEQSQSSGSVL
PVSSSCHVFKPTARAGGVVIESSSPEEEEKDPMTCLRLSLPWVNESTTPPELFPVKREEEE
EKEREISGLGGDFMTVVQEMIKTEVRSYMADLQLGNGGGAGGGASSCMVQGTNGRNVG
FREFIGLGRIE*
>AtMYB71
MSLWGGMGGGWGMVEEGWRKGPWTAEEDRLLIDYVQLHGEGRWNSVARLAGLKRNG
KSCRLRWVNYLRPDLKRGQITPHEETIILELHAKWGNRWSTIARSLPGRTDNEIKNYWRT
HFKKKTKSPTNSAEKTKNRILKRQQFQQQRQMELQQEQQLLQFNQIDMKKIMSLLDDDN
NNGDNTFSSSSSGESGALYVPHQITHSTTTSGCEPNSNGYYPVVPVTIPEANVNEDNAIWD
GLWNLDFEGQGSFGGAACAPRKHYFQNMVIPFC*
>AtMYB72
MGKGRAPCCDKNKVKRGPWSPQEDLTLITFIQKHGHQNWRSLPKLAGLLRCGKSCRLRW
INYLRPDVKRGNFSKKEEDAIIHYHQTLGNKWSKIASFLPGRTDNEIKNVWNTHLKKRLTP
SSSSSSLSSTHDQSTKADHDKNCDGAQEEIHSGLNESQNSATSSHHQGECMHTKPELHEV
NGLNEIQFLLDHDDFDDITSEFLQDNDILFPLDSLLHNHQTHISTQEMTREVTKSQSFDH
PQPDIPCGFEDTNEESDLRRQLVESTTPNNEYDEWFNFIDNQTYFDDFNFVGEVCL*
>AtMYB73
MSNPTRKNMERIKGPWSPEEDDLLQRLVQKHGPRNWSLISKSIPGRSGKSCRLRWCNQLS
PEVEHRAFSQEEDETIIRAHARFGNKWATISRLLNGRTDNAIKNHWNSTLKRKCSVEGQS
CDFGGNGGYDGNLGEEQPLKRTASGGGGVSTGLYMSPGSPSGSDVSEQSSGGAHVFKPT
VRSEVTASSSGEDPPTYLSLSLPWTDETVRVNEPVQLNQNTVMDGGYTAELFPVRKEEQV
EVEEEEAKGISGGFGGEFMTVVQEMIRTEVRSYMADLQRGNVGGSSSGGGGGGSCMPQS

VNSRRVGFREFIVNQIGIGKME*
>AtMYB74
MGRSPCCEKKNGLKKGPWTPEEDQKLIDYINIHGYGNWRTLPKNAGLQRCGKSCRLRWT
NYLRPDIKRGRFSFEEEETIIQLHSIMGNKWSAIAARLPGRTDNEIKNYWNTHIRKRLLKM
GIDPVTHTPRLDLLDISSILSSSIYNSSHHHHHHHQQHMNMSRLMMSDGNHQPLVNPEIL
KLATSLFSNQNHPNNTHENNTVNQTEVNQYQTGYNMPGNEELQSWFPIMDQFTNFQDL
MPMKTTVQNSLSYDDDCSKSNFVLEPYYSDFASVLTTPSSSPTPLNSSSSTYINSSTCSTED
EKESYYSDNITNYSFDVNGFLQFQ*
>AtMYB75
MEGSSKGLRKGAWTTEEDSLLRQCINKYGEGKWHQVPVRAGLNRCRKSCRLRWLNYLK
PSIKRGKLSSDEVDLLLRLHRLLGNRWSLIAGRLPGRTANDVKNYWNTHLSKKHEPCCKI
KMKKRDITPIPTTPALKNNVYKPRPRSFTVNNDCNHLNAPPKVDVNPPCLGLNINNVCDN
SIIYNKDKKKDQLVNNLIDGDNMWLEKFLEESQEVDILVPEATTTEKGDTLAFDVDQLWS
LFDGETVKFD*
>AtMYB76
MSKRPYCIGEGLKKGAWTTEEDKKLISYIHDHGEGGWRDIPEKAGLKRCGKSCRLRWTN
YLKPDIKRGEFSYEEEQIIIMLHASRGNKWSVIARHLPKRTDNEVKNYWNTHLKKRLIDD
GIDPVTHKPLASSNPNPVEPMKFDFQKKSNQDEHSSQSSSTTPASLPLSSNLNSVKSKISS
GETQIESGHVSCKKRFGRSSSTSRLLNKVAARASSIGNILSTSIEGTLRSPASSSGLPDS
FSQSYEYMIDNKEDLGTSIDLNIPEYDFPQFLEQLINDDDENENIVGPEQDLLMSDFPST
FVDEDDILGDITSWSTYLLDHPNFMYESDQDSDEKNFL*
>AtMYB77
MADRVKGPWSQEEDEQLRRMVEKYGPRNWSAISKSIPGRSGKSCRLRWCNQLSPEVEHR
PFSPEEDETIVTARAQFGNKWATIARLLNGRTDNAVKNHWNSTLKRKCSGGVAVTTVTET
EEDQDRPKKRRSVSFDSAFAPVDTGLYMSPESPNGIDVSDSSTIPSPSSPVAQLFKPMPIS
GGFTVVPQPLPVEMSSSSEDPPTSLSLSLPGAENTSSSHNNNNNALMFPRFESQMKINVE
ERGEGRRGEFMTVVQEMIKAEVRSYMAEMQKTSGGFVVGGLYESGGNGGFRDCGVITP
KVE*
>AtMYB78
MGDKGRSLKINKNMEEFTKVEEEMDVRRGPWTVEEDLELINYIASHGEGRWNSLARCAE
LKRTGKSCRLRWLNYLRPDVRRGNITLEEQLLILELHTRWGNSNEFDHKFSSSCEDRWSKI
AQYLPGRTDNEIKNYWRTRVQKHAKQLKCDVNSQQFKDTMKYLWMPRLVERIQAASIGS
VSMSSCVTTSSDQFVINNNNTNNVDNLALMSNPNGYITPDNSSVAVSPVSDLTECQVSSEV
WKIGQDENLVDPKMTSPNYMDNSSGLLNGDFTKMQDQSDLNWFENINGMVPNYSDSFW
NIGNDEDFWLLQQHQQVHDNGSF*
>AtMYB79
MVEEVWRKGPWTAEEDRLLIEYVRVHGEGRWNSVSKLAGLKRNGKSCRLRWVNYLRPD
LKRGQITPHEESIILELHAKWGNRWSTIARSLPGRTDNEIKNYWRTHFKKKAKPTTNNAEK
IKSRLLKRQHFKEQREIELQQEQQLFQFDQLGMKKIISLLEENNSSSSDGGGDVFYYPDQ
ITHSSKPFGYNSNSLEEQLQGRFSPVNIPDANTMNEDNAIWDGFWNMDVVNGHGGNLGV
VAATAACGPRKPYFHNLVIPFC*
>AtMYB80
MGRIPCCEKENVKRGQWTPEEDNKLASYIAQHGTRNWRLIPKNAGLQRCGKSCRLRWT
NYLRPDLKHGQFSEAEEHIIVKFHSVLGNRWSLIAAQLPGRTDNDVKNYWNTKLKKKLS

GMGIDPVTHKPFSHLMAEITTTLNPPQVSHLAEAALGCFKDEMLHLLTKKRVDLNQINFS
NHNPNPNNFHEIADNEAGKIKMDGLDHGNGIMKLWDMGNGFSYGSSSSSFGNEERNDGS
ASPAVAAWRGHGGIRTAVAETAAAEEEERRKLKGEVVDQEEIGSEGGRGDGMTMMRNHH
HHQHVFNVDNVLWDLQADDLINHMV*
>AtMYB81
MGKVRQDSGSDDDNSIKKSFTKGPWTQAEDNLLIAYVDKHGDGNWNAVQNNSGLSRCG
KSCRLRWVNHLRPDLKKGAFTEKEEKRVIELHALLGNKWARMAEELPGRTDNEIKNFWN
TRLKRLQRLGLPVYPDEVREHAMNAATHSGLNTDSLDGHHSQEYMEADTVEIPEVDFEH
LPLNRSSSYYQSMLRHVPPTNVFVRQKPCFFQPPNVYNLIPPSPYMSTGKRPREPETAFPCP
GGYTMNEQSPRLWNYPFVENVSEQLPDSHLLGNAAYSSPPGPLVHGVENFEFPSFQYHEE
PGGWGADQPNPMPEHESDNTLVQSPLTAQTPSDCPSSSLYDGLLESVVYGSSGEKPATDTD
SESSLFQSFTPANENITGKTCFLTLYALHALHCLCNQFKKSPLLHLHDKLNWCNKFRFNSF
KSGTHIL*
>AtMYB82
MECKREEGKSYVKRGLWKPEEDMILKSYVETHGEGNWADISRRSGLKRGGKSCRLRWK
NYLRPNIKRGSMSPQEQDLIIRMHKLLGNRWSLIAGRLPGRTDNEVKNYWNTHLNKKPNS
RRQNAPESIVGATPFTDKPVMSTELRRSHGEGGEEESNTWMEETNHFGYDVHVGSPLPLI
SHYPDNTLVFDPCFSFTDFFPLL*
>AtMYB83
MMMRKPDITTIRDKGKPNHACGGNNNKPKLRKGLWSPDEDEKLIRYMLTNGQGCWSDI
ARNAGLLRCGKSCRLRWINYLRPDLKRGSFSPQEEDLIFHLHSILGNRWSQIATRLPGRTD
NEIKNFWNSTLKKRLKNNSNNNTSSGSSPNNSNSNSLDPRDQHVDMGGNSTSLMDDYHH
DENMMTVGNTMRMDSSSPFNVGPMVNSVGLNQLYDPLMISVPDNGYHQMGNTVNVFS
VNGLGDYGNTILDPISKRVSVEGDDWFIPPSENTNVIACSTSNNLNLQALDPCFNSKNLCH
SESFKVGNVLGIENGSWEIENPKIGDWDLDGLIDNNSSFPFLDFQVD*
>AtMYB84
MGRAPCCDKANVKKGPWSPEEDAKLKSYIENSGTGGNWIALPQKIGLKRCGKSCRLRWL
NYLRPNIKHGGFSEEEENIICSLYLTIGSRWSIIAAQLPGRTDNDIKNYWNTRLKKKLINK
QRKELQEACMEQQEMMVMMKRQHQQQQIQTSFMMRQDQTMFTWPLHHHNVQVPALF
MNQTNSFCDQEDVKPVLIKNMVKIEDQELEKTNPHHHQDSMTNAFDHLSFSQLLLDPNH
NHLGSGEGFSMNSILSANTNSPLLNTSNDNQWFGNFQAETVNLFSGASTSTSADQSTISWE
DISSLVYSDSKQFF*
>AtMYB85
MGRQPCCDKLGVKKGPWTVEEDKKLINFILTNGHCCWRALPKLAGLRRCGKSCRLRWT
NYLRPDLKRGLLSHDEEQLVIDLHANLGNKWSKIASRLPGRTDNEIKNHWNTHIKKKLLK
MGIDPMTHQPLNQEPSNIDNSKTIPSNPDDVSVEPKTTNTKYVEISVTTTEEESSSTVTDQN
SSMDNENHLIDNIYDDDELFSYLWSDETTKDEASWSDSNFGVGGTLYDHNISGADADFPI
WSPERINDEKMFLDYCQDFGVHDFGF*
>AtMYB86
MGRHSCCFKQKLRKGLWSPEEDEKLLNYITRHGHGCWSSVPKLAGLQRCGKSCRLRWIN
YLRPDLKRGAFSQDEESLIIELHAALGNRWSQIATRLPGRTDNEIKNFWNSCLKKKLRRKG
IDPTTHKPLITNELQSLNVIDQKLTSSEVVKSTGSINNLHDQSMVVSSQQGPWWFPANTT
TTNQNSAFCFSSSNTTTVSDQIVSLISSMSTSSSPTPMTSNFSPAPNNWEQLNYCNTVPS
QSNSIYSAFFGNQYTEASQTMNNNNPLVDQHHHHQDMKSWASEILHYTEHNQSSETVIEA

EVKPDIANYYWRSASSSSSPNQEAATLLHDANVEVYGKNLQKLNNMVFDQSL*
>AtMYB87
MGRAPCCDKMAVKKGPWSTEEDAVLKSYIEKHGTGNNWISLPQRIGIKRCGKSCRLRWL
NYLRPNLKHGGFTDEEDYIICSLYITIGSRWSIIASQLPGRTDNDIKNYWNTRLKKKLLSK
QGKAFHQQLNVKFERGTTSSSSSQNQIQIFHDENTKSNQTLYNQVVDPSMRAFAMEEQSM
IKNQILEPFSWEPNKVLFDVDYDAAASSYHHHASPSLNSMSSTSSIGTNNSSLQMSHYTV
NHNDHDQPDMFFMDGFENFQAELFDEIANNNTVENGFDGTEILINNNYLDHDISSFIDYP
LYDNE*
>AtMYB88
MEETTKQNNMKKKKKILLHSDDSKKKERHIVTWSPEEDDILRKQISLQGTENWAIIASKF
NDKSTRQCRRRWYTYLNSDFKRGGWSPEEDTLLCEAQRLFGNRWTEIAKVVSGRTDNAV
KNRFTTLCKKRAKHEAMAKENRIACCVNSDNKRLLFPDGISTPLKAESESPLTKKMRRSHI
PNLTEIKSYGDRSHIKVESTMNQQRRHPFSVVAHNATSSDGTEEQKQIGNVKESDGEDKS
NQEVFLKKDDSKVTALMQQAELLSSLAQKVNADNTDQSMENAWKVLQDFLNKSKEND
LFRYGIPDIDFQLDEFKDLVEDLRSSNEDSQSSWRQPDLHDSPASSEYSSGSGSGSTIMTHP
SGDKTQQLMSDTQTTSHQQNGGELLQDNGIVSDATVEQVGLLSTGHDVLKNSNETVPIPG
EEEFNSPVQVTPLFRSLAAGIPSPQFSESERNFLLKTLGVESPSPYPSANPSQPPPCKRVL
LDSL*
>AtMYB89
MYLFMYKCNIVLEETHVFQNTPCDVSLQRPFNGFGENNALPLRKMHQEEKKKKHRGGH
WTLSEDLKLKELVAVFGPQNWKFIGEKMEPRTSLSCRQRWFNQLDPKINKRNFTDEEEEK
LLRAHILYGNKWSKIAKLFNRRTDHAVKNHWHSLMNRIIRKQSASDIRSFDNIQNYQTSNF
LPGLCLLNTKQ*
>AtMYB90
MEGSSKGLRKGAWTAEEDSLLRLCIDKYGEGKWHQVPLRAGLNRCRKSCRLRWLNYLK
PSIKRGRLSNDEVDLLLRLHKLLGNRWSLIAGRLPGRTANDVKNYWNTHLSKKHESSCCK
SKMKKKNIISPPTTPVQKIGVFKPRPRSFSVNNGCSHLNGLPEVDLIPSCLGLKKNNVCENS
ITCNKDDEKDDFVNNLMNGDNMWLENLLGENQEADAIVPEATTAEHGATLAFDVEQLW
SLFDGETVELD*
>AtMYB91
MKERQRWSGEEDALLRAYVRQFGPREWHLVSERMNKPLNRDAKSCLERWKNYLKPGIK
KGSLTEEEQRLVIRLQEKHGNKWKKIAAEVPGRTAKRLGKWWEVFKEKQQREEKESNKR
VEPIDESKYDRILESFAEKLVKERSNVVPAAAAAATVVMANSNGGFLHSEQQVQPPNPVIP
PWLATSNNGNNVVARPPSVTLTLSPSTVAAAAPQPPIPWLQQQQPERAENGPGGLVLGSM
MPSCSGSSESVFLSELVECCRELEEGHRAWADHKKEAAWRLRRLELQLESEKTCRQREKM
EEIEAKMKALREEQKNAMEKIEGEYREQLVGLRRDAEAKDQKLADQWTSRHIRLTKFLE
QQMGCRLDRP*
>AtMYB92
MGRSPISDDSGLKKGPWTPDEDEKLVNYVQKHGHSSWRALPKLAGLNRCGKSCRLRWT
NYLRPDIKRGRFSPDEEQTILNLHSVLGNKWSTIANQLPGRTDNEIKNFWNTHLKKKLIQM
GFDPMTHRPRTDIFSGLSQLMSLSSNLRGFVDLQQQFPIDQEHTILKLQTEMAKLQLFQYL
LQPSSMSNNVNPNDFDTLSLLNSIASFKETSNNTTSNNLDLGFLGSYLQDFHSLPSLKTL
NSNMEPSSVFPQNLDDNHFKFSTQRENLPVSPIWLSDPSSTTPAHVNDDLIFNQYGIEDV
NSNITSSSGQESGASASAAWPDHLLDDSIFSDIP*

>AtMYB93
MGRSPCCDENGLKKGPWTPEEDQKLIDYIHKHGHGSWRALPKLADLNRCGKSCRLRWT
NYLRPDIKRGKFSAEEEQTILHLHSILGNKWSAIATHLQGRTDNEIKNFWNTHLKKKLIQM
GIDPVTHQPRTDLFASLPQLIALANLKDLIEQTSQFSSMQGEAAQLANLQYLQRMFNSSAS
LTNNNGNNFSPSSILDIDQHHAMNLLNSMVSWNKDQNPAFDPVLELEANDQNQDLFPLGF
IIDQPTQPLQQQKYHLNNSPSELPSQGDPLLDHVPFSLQTPLNSEDHFIDNLVKHPTDHE
HEHDDNPSSWVLPSLIDNNPKTVTSSLPHNNPADASSSSSYGGCEAASFYWPDICFDESL
MNVIS*
>AtMYB94
MGRPPCCDKIGVKKGPWTPEEDIILVSYIQEHGPGNWRSVPTHTGLRRCSKSCRLRWTNY
LRPGIKRGNFTEHEEKMILHLQALLGNRWAAIASYLPERTDNDIKNYWNTHLKKKLKKM
NDSCDSTINNGLDNKDFSISNKNTTSHQSSNSSKGQWERRLQTDINMAKQALCDALSIDK
PQNPTNFSIPDLGYGPSSSSSSTTTTTTTTRNTNPYPSGVYASSAENIARLLQNFMKDTPK
TSVPLPVAATEMAITTAASSPSTTEGDGEGIDHSLFSFNSIDEAEEKPKLIDHDINGLIT
QGSLSLFEKWLFDEQSHDMIINNMSLEGQEVLF*
>AtMYB95
MGRTTWFDVDGLRKGEWTAEEDRKLVVYINEHGLGEWGSLPKRAGLQRCGKSCRLRWL
NYLRPGIKRGKFTPQEEEEIIKYHALLGNRWAAIAKQMPNRTDNDIKNHWNSCLKKRLAK
KGIDPMTHEPTTTTSLTVDVTSSSTTSSPTPSPTSSSFSSCSSTGSARFLNKLAAGISSRKH
GLESIKTVILAEQPREAVDEEKMMTINMKEKELISCYMEIDETMSIDELPCDDSTSGFVA
FDDYSLIDPYRDGVYYVSDFYDETEHLDLFLL*
>AtMYB96
MGRPPCCEKIGVKKGPWTPEEDIILVSYIQEHGPGNWRSVPTHTGLRRCSKSCRLRWTNY
LRPGIKRGNFTEHEEKTIVHLQALLGNRWAAIASYLPERTDNDIKNYWNTHLKKKLKKIN
ESGEEDNDGVSSSNTSSQKNHQSTNKGQWERRLQTDINMAKQALCEALSLDKPSSTLSSS
SSLPTPVITQQNIRNFSSALLDRCYDPSSSSSSTTTTTTSNTTNPYPSGVYASSAENIAR
LLQDFMKDTPKALTLSSSSPVSETGPLTAAVSEEGGEGFEQSFFSFNSMDETQNLTQETS
FFHDQVIKPEITMDQDHGLISQGSLSLFEKWLFDEQSHEMVGMALAGQEGMF*
>AtMYB97
MIVYGGGASEDGEGGGVVLKKGPWTVAEDETLAAYVREYGEGNWNSVQKKTWLARCG
KSCRLRWANHLRPNLRKGSFTPEEERLIIQLHSQLGNKWARMAAQLPGRTDNEIKNYWNT
RLKRFQRQGLPLYPPEYSQNNHQQQMYPQQPSSPLPSQTPASSFTFPLLQPPSLCPKRCYNT
AFSPKASYISSPTNFLVSSPTFLHTHSSLSSYQSTNPVYSMKHELSSNQIPYSASLGVYQV
SKFSDNGDCNQNLNTGLHTNTCQLLEDLMEEAEALADSFRAPKRRQIMAALEDNNNNN
NFFSGGFGHRVSSNSLCSLQGLTPKEDESLQMNTMQDEDITKLLDWGSESEEISNGQSSVI
TTENNLVLDDHQFAFLFPVDDDTNNLPGIC*
>AtMYB98
MENFVDENGFASLNQNIFTRDQEHMKEEDFPFEVVDQSKPTSFLQDFHHLDHDHQFDHH
HHHGSSSSHPLLSVQTTSSCINNAPFEHCSYQENMVDFYETKPNLMNHHHFQAVENSYFT
RNHHHHQEINLVDEHDDPMDLEQNNMMMMRMIPFDYPPTETFKPMNFVMPDEISCVSA
DNDCYRATSFNKTKPFLTRKLSSSSSSSSWKETKKSTLVKGQWTAEEDRVLIQLVEKYGLR
KWSHIAQVLPGRIGKQCRERWHNHLRPDIKKETWSEEEDRVLIEFHKEIGNKWAEIAKRLP
GRTENSIKNHWNATKRRQFSKRKCRSKYPRPSLLQDYIKSLNMGALMASSVPARGRRRES
NNKKKDVVVAVEEKKKEEEVYGQDRIVPECVFTDDFGFNEKLLEEGCSIDSLLDDIPQPDI

DAFVHGL*
>AtMYB99
MGGRKPCCDEVGLRKGPWTVEEDGKLVDFLRARGNCGGGGGGWCWRDVPKLAGLRR
CGKSCRLRWTNYLRPDLKRGLFTEEEIQLVIDLHARLGNRWSKIAVELPGRTDNDIKNYW
NTHIKRKLIRMGIDPNTHRRFDQQKVNEEETILVNDPKPLSETEVSVALKNDTSAVLSGNL
NQLADVDGDDQPWSFLMENDEGGGGDAAGELTMLLSGDITSSCSSSSSLWMKYGEFGYE
DLELGCFDV*
>AtMYB100
MKKNYQKKNIKVVSTSKYLKKSDIDKVNWTESEDIKLKEIMALGPKNKWTKVAKKFEGR
TGKQCRERWYNHARPNIKKTAWSEEEDQILIEAHKVLGTKWVEIAQQLPGRSDNNIKNH
WNTTKRRVQNKRGGTVNPVGNNILENYIRCITINNEDFLKTDGSYGEPTNIESDDDSKDM
LYGEMNLSLETITTQTTKPLTNASTTSPYVQMPEDNYTMEDCESLEDILELLRWWE*
>AtMYB101
MDGGGETTATATMEGRGLKKGPWTTTEDAILTEYVRKHGEGNWNAVQKNSGLLRCGKS
CRLRWANHLRPNLKKGSFTPDEEKIIIDLHAKLGNKWARMASQLPGRTDNEIKNYWNTR
MKRRQRAGLPLYPHEIQHQGIDIDDEFEFDLTSFQFQNQDLDHNHQNMIQYTNSSNTSSSS
SSFSSSSSQPSKRLRPDPLVSTNPGLNPIPDSSMDFQMFSLYNNSLENDNNQFGFSVPLSSS
SSSNEVCNPNHILEYISENSDTRNTNKKDIDAMSYSSLLMGDLEIRSSSFPLGLDNSVLE
LPSNQRPTHSFSSSPIIDNGVHLEPPSGNSGLLDALLEESQALSRGGLFKDVRVSSSDLC
EVQDKRVKMDFENLLIDHLNSSNHSSLGANPNIHNKYNEPTMVKVTVDDDDELLTSLLN
NFPSTTTPLPDWYRVTEMQNEASYLAPPSGILMGNHQGNGRVEPPTVPPSSSVDPMASLG
SCYWSNMPSIC*
>AtMYB102
MARSPCCEKNGLKKGPWTSEEDQKLVDYIQKHGYGNWRTLPKNAGLQRCGKSCRLRWT
NYLRPDIKRGRFSFEEEETIIQLHSFLGNKWSAIAARLPGRTDNEIKNFWNTHIRKKLLRMG
IDPVTHSPRLDLLDISSILASSLYNSSSHHMNMSRLMMDTNRRHHQQHPLVNPEILKLAT
SLFSQNQNQNLVVDHDSRTQEKQTVYSQTGVNQYQTNQYFENTITQELQSSMPPFPNEAR
QFNNMDHHFNGFGEQNLVSTSTTSVQDCYNPSFNDYSSSNFVLDPSYSDQSFNFANSVLN
TPSSSPSPTTLNSSYINSSSCSTEDEIESYCSNLMKFDIPDFLDVNGFII*
>AtMYB103
MGHHSCCNQQKVKRGLWSPEEDEKLIRYITTHGYGCWSEVPEKAGLQRCGKSCRLRWIN
YLRPDIRRGRFSPEEEKLIISLHGVVGNRWAHIASHLPGRTDNEIKNYWNSWIKKKIRKPH
HHYSRHQPSVTTVTLNADTTSIATTIEASTTTTSTIDNLHFDGFTDSPNQLNFTNDQETN
IKIQETFFSHKPPLFMVDTTLPILEGMFSENIITNNNKNNDHDDTQRGGRENVCEQAFLT
TNTEEWDMNLRQQEPFQVPTLASHVFNNSSNSNIDTVISYNLPALIEGNVDNIVHNENSN
VQDGEMASTFECLKRQELSYDQWDDSQQCSNFFFWDNLNINVEGSSLVGNQDPSMNLGS
SALSSSFPSSF*
>AtMYB104
MIQDQANDLLAMKKTFTKSKWKPEEDRILKDYVIQYGDRTWTHVPKRTGLPHNPASCRF
RWMNHLKPSLKKGPFTDEEEKRVLQLHAVLGNKWSQMAREFPGRTDNEIKNFWNARRM
RLKGKGLPVYPDEVREQAIRTAAQYGVKVELLNAHYSQDSLMAGNVEKPQELNNLALN
QCSPYYQSTLANVQPSRNRVMEPETTFPFTGGSAMNEQNPTLLCNPYVESTQEQLPDSHL
FGNVTYSSPPMPLIHEVENLELPSFQGFDFHEEPSSFGAEQYNPMLNLEPHNTLVQSPLIGQ
TPTDFPSSFYDELLDELLESVVNGSLGEIPKTDTSSESQLFQSSLRSHTDATPDIANTTGYVG

SNERNTTNDDDWIRLLLDEGFI*
>AtMYB105
MMFKVIFNICSSPPMRMEMVHADVASLSITPCFPSSLSSSSHHHYNQQQHCIMSEDQHHS
MDQTTSSDYFSLNIDNAQHLRSYYTSHREEDMNPNLSDYSNCNKKDTTVYRSCGHSSKA
SVSRGHWRPAEDTKLKELVAVYGPQNWNLIAEKLQGRSGKSCRLRWFNQLDPRINRRAFT
EEEEERLMQAHRLYGNKWAMIARLFPGRTDNSVKNHWHVIMARKFREQSSSYRRRKTM
VSLKPLINPNPHIFNDFDPTRLALTHLASSDHKQLMLPVPCFPGYDHENESPLMVDMFETQ
MMVGDYIAWTQEATTFDFLNQTGKSEIFERINEEKKPPFFDFLGLGTV*
>AtMYB106
MPIHVRDREKGRLQNLNRDIFCCVSPSIYQSDAKRAAFVIILIMIISPCCDKAGLKKGPW
TPEEDQKLLAYIEEHGHGSWRSLPEKAGLQRCGKSCRLRWTNYLRPDIKRGKFTVQEEQT
IIQLHALLGNRWSAIATHLPKRTDNEIKNYWNTHLKKRLIKMGIDPVTHKHKNETLSSST
GQSKNAATLSHMAQWESARLEAEARLARESKLLHLQHYQNNNNLNKSAAPQQHCFTQK
TSTNWTKPNQGNGDQQLESPTSTVTFSENLLMPLGIPTDSSRNRNNNNNESSAMIELAVSS
STSSDVSLVKEHEHDWIRQINCGSGGIGEGFTSLLIGDSVGRGLPTGKNEATAGVGNESEY
NYYEDNKNYWNSILNLVDSSPSDSATMF*
>AtMYB107
MGRSPCCDESGLKKGPWTPEEDQKLINHIRKHGHGSWRALPKQAGLNRCGKSCRLRWT
NYLRPDIKRGNFTAEEEQTIINLHSLLGNKWSSIAGHLPGRTDNEIKNYWNTHIRKKLIQM
GIDPVTHRPRTDHLNVLAALPQLLAAANFNNLLNLNQNIQLDATSVAKAQLLHSMIQVLS
NNNTSSSFDIHHTTNNLFGQSSFLENLPNIENPYDQTQGLSHIDDQPLDSFSSPIRVVAYQ
HDQNFIPPLISTSPDESKETQMMVKNKEIMKYNDHTSNPSSTSTFTQDHQPWCDIIDDEA
SDSYWKEIIEQTCSEPWPFRE*
>AtMYB108
MDEKGRSLKNNNMEDEMDLKRGPWTAEEDFKLMNYIATNGEGRWNSLSRCAGLQRTG
KSCRLRWLNYLRPDVRRGNITLEEQLLILELHSRWGNRWSKIAQYLPGRTDNEIKNYWRT
RVQKHAKQLKCDVNSQQFKDTMKYLWMPRLVERIQSASASSAAAATTTTTTTGSAGTS
SCITTSNNQFMNYDYNNNNMGQQFGVMSNNDYITPENSSVAVSPASDLTEYYSAPNPNPE
YYSGQMGNSYYPDQNLVSSQLLPDNYFDYSGLLDEDLTAMQEQSNLSWFENINGAASSS
DSLWNIGETDEEFWFLQQQQQFNNNGSF*
>AtMYB109
MEGETHQSEPLPLASGDSDEGISAAIEAELAELAAGDSSGGGGCGGGGGGIRSKVKGPWS
TEEDAVLTKLVRKLGPRNWSLIARGIPGRSGKSCRLRWCNQLDPCLKRKPFSDEEDRMII
SAHAVHGNKWAVIAKLLTGRTDNAIKNHWNSTLRRKYADLWNNGQWMANSVTTASVKN
ENVDETTNPPSSKQQLPQGDINSSPPKPPQVSDVVMEEAANEPQEPQEQQEQAPPVVSNV
PTENNVFRPVARVGAFSIYNPTSQKNGYRDYNIVPCEGPLIQAAKPDSLAGKFLQSLCDEP
QIPSKCGHGCSTLPAETKFSRNSVLGPEFVDYEEPSAVFNQELISIATDLNNIAWIKSGLD
NAVVREAEQSLKMDNYNYNDPRIKFTGMMPRQDFFCARS*
>AtMYB110
MKMDFSCFQEYPFEFHCRGTTFNGFRENNAVSETVEEFCNKRRMQKKSDDLKTKKKKKQ
SVSRVCSRGHWRISEDTQLMELVSVYGPQNWNHIAESMQGRTGKSCRLRWFNQLDPRIN
KRAFSDEEEERLLAAHRAFGNKWAMIAKLFNGRTDNALKNHWHVLMARKMRQQSSSY
VQRFNGSAHESNTDHKIFNLSPGNVDDDEDVNLKKCSWEMLKEGTTNLKAQYLQEEYSS
SRMPMQGPHHHYSTFPADSLALTLHVSIQEPSSSSSLSLPSSSTTGEHTMVTRYFETIKPPAF

IDFLGVGH*
>AtMYB111
MGRAPCCEKIGLKRGRWTAEEDEILTKYIQTNGEGSWRSLPKKAGLLRCGKSCRLRWINY
LRRDLKRGNITSDEEEIIVKLHSLLGNRWSLIATHLPGRTDNEIKNYWNSHLSRKIYAFT
AVSGDGHNLLVNDVVLKKSCSSSSGAKNNNKTKKKKKGRTSRSSMKKHKQMVTASQCF
SQPKELESDFSEGGQNGNFEGESLGPYEWLDGELERLLSSCVWECTSEEAVIGVNDEKVC
ESGDNSSCCVNLFEEEQGSETKIGHVGITEVDHDMTVEREREGSFLSSNSNENNDKDWW
VGLCNSSEVGFGVDEELLDWEFQGNVTCQSDDLWDLSDIGEITLE*
>AtMYB112
MNISRTEFANCKTLINHKEEVEEVEKKMEIEIRRGPWTVEEDMKLVSYISLHGEGRWNSL
SRSAGLNRTGKSCRLRWLNYLRPDIRRGDISLQEQFIILELHSRWGNRWSKIAQHLPGRT
DNEIKNYWRTRVQKHAKLLKCDVNSKQFKDTIKHLWMPRLIERIAATQSVQFTSNHYSPE
NSSVATATSSTSSSEAVRSSFYGGDQVEFGTLDHMTNGGYWFNGGDTFETLCSFDELNKW
LIQ*
>AtMYB113
MGESPKGLRKGTWTTEEDILLRQCIDKYGEGKWHRVPLRTGLNRCRKSCRLRWLNYLKP
SIKRGKLCSDEVDLVLRLHKLLGNRWSLIAGRLPGRTANDVKNYWNTHLSKKHDERCCK
TKMINKNITSHPTSSAQKIDVLKPRPRSFSDKNSCNDVNILPKVDVVPLHLGLNNNYVCES
SITCNKDEQKDKLININLLDGDNMWWESLLEADVLGPEATETAKGVTLPLDFEQIWARFD
EETLELN*
>AtMYB114
MEGSSKGLRKGAWTAEEDSLLRQCIGKYGEGKWHQVPLRAGLNRCRKSCRLRWLNYLK
PSIKRGKFSSDEVDLLLRLHKLLGNRWSLIAGRLPGRTANDVKNYWNTHLSKKHEPCCKT
KIKRINIITPPNTPAQKVDIF*
>AtMYB115
MYHQNLISSTPNQNSNPHDWDIQNPLFSIHPSAEIPSKYPFMGITSCPNTNVFEEFQYKI
TNDQNFPTTYNTPFPVISEGISYNMHDVQENTMCGYTAHNQGLIIGCHEPVLVHAVVESQ
QFNVPQSEDINLVSQSERVTEDKVMFKTDHKKKDIIGKGQWTPTEDELLVRMVKSKGTK
NWTSIAKMFQGRVGKQCRERWHNHLRPNIKKNDWSEEEDQILIEVHKIVGNKWTEIAKR
LPGRSENIVKNHWNATKRRLHSVRTKRSDAFSPRNNALENYIRSITINNNALMNREVDSIT
ANSEIDSTRCENIVDEVMNLNLHATTSVYVPEQAVLTWGYDFTKCYEPMDDTWMLMNG
WN*
>AtMYB116
MSNITKKKCNGNEEGAEQRKGPWTLEEDTLLTNYISHNGEGRWNLLAKSSGKSCRLRWL
NYLKPDIKRGNLTPQEQLLILELHSKWGNRWSKISKYLPGRTDNDIKNYWRTRVQKQARQ
LNIDSNSHKFIEVVRSFWFPRLINEIKDNSYTNNIKANAPDLLGPILRDSKDLGFNNMDCS
TSMSEDLKKTSQFMDFSDLETTMSLEGSRGGSSQCVSEVYSSFPCLEEEYMVAVMGSSDI
SALHDCHVADSKYEDDVTQDLMWNMDDIWQFNEKILIFEDQTVLVLVSLDLLRRTNLISL
LHVKESKR*
>AtMYB117
MFITEKQVWMDEIVARRASSSWDFPFNDINIHQHHHRHCNTSHEFEILKSPLGDVAVHEE
ESNNNNPNFSNSESGKKETTDSGQSWSSSSSKPSVLGRGHWRPAEDVKLKELVSIYGPQN
WNLIAEKLQGRSGKSCRLRWFNQLDPRINRRAFTEEEEERLMQAHRLYGNKWAMIARLF
PGRTDNSVKNHWHVVMARKYREHSSAYRRRKLMSNNPLKPHLTNNHHPNPNPNYHSFIS

TNHYFAQPFPEFNLTHHLVNNAPITSDHNQLVLPFHCFQGYENNEPPMVVSMFGNQMMV
GDNVGATSDALCNIPHIDPSNQEKPEPNDAMHWIGMDAVDEEVFEKAKQQPHFFDFLGL
GTA*
>AtMYB118
MEFESVFKMHYPYLAAVIYDDSSTLKDFHPSLTDDFSCVHNVHHKPSMPHTYEIPSKETI
RGITPSPCTEAFEACFHGTSNDHVFFGMAYTTPPTIEPNVSHVSHDNTMWENDQNQGFIF
GTESTLNQAMADSNQFNMPKPLLSANEDTIMNRRQNNQVMIKTEQIKKKNKRFQMRRIC
KPTKKASIIKGQWTPEEDKLLVQLVDLHGTKKWSQIAKMLQGRVGKQCRERWHNHLRPD
IKKDGWTEEEDIILIKAHKEIGNRWAEIARKLPGRTENTIKNHWNATKRRQHSRRTKGKDE
ISLSLGSNTLQNYIRSVTYNDDPFMTANANANIGPRNMRGKGKNVMVAVSEYDEGECKYI
VDGVNNLGLEDGRIKMPSLAAMSASGSASTSGSASGSGSGVTMEIDEPMTDSWMVMHG
CDEVMMNEIALLEMIAHGRL
>AtMYB119
MEDRRLVHGAAPPLTAVERFLYGQKNDALCSKKQESSRDQPIVKTKISIETRSDNKENTT
FGPTREKHLVLNGGNRNPTGEVVARSAARDYQNSTKKRSSKNLIKGQWTAEEDRKLIRLV
RQHGERKWAMISEKLEGRAGKQCRERWHNHLRPDIKKDGWSEEEERVLVESHMRIGNK
WAEIAKLIPGRTENSIKNHWNATKRRQNSKRKHKRESNADNNDRDASPSAKRPCILQDYI
KSIERNNINKDNDEKKNENTISVISTPNLDQIYSDGDSASSILGGPYDEELDYFQNIFANHP
ISLENLGLSQTSDEVTQSSSSGFMIKNPNPNLHDSVGIHHQEATITAPANTPHLASDIYL
SYLLNGTTSSYSDTHFPSSSSSTSSTTVEHGGHNEFLEPQANSTSERREMDLIEMLSGSI
QGSNICFPLV*
>AtMYB120
MIMYGGGGAGKDGGSTNHLSDGGVILKKGPWTAAEDEILAAYVRENGEGNWNAVQKNT
GLARCGKSCRLRWANHLRPNLKKGSFTGDEERLIIQLHAQLGNKWARMAAQLPGRTDNE
IKNYWNTRLKRLLRQGLPLYPPDIIPNHQLHPHPHHQQQQQHNHHHHHHQQQQQHQQM
YFQPQSSQRNTPSSSPLPSPTPANAKSSSSFTFHTTTANLLHPLSPHTPNTPSQLSSTPPPPPL
SSPLCSPRNNQYPTLPLFALPRSQINNNNNGNFTFPRPPPLLQPPSSLFAKRYNNANTPLN
CINRVSTAPFSPVSRDSYTSFLTLPYPSPTAQTATYHNTNNPYSSSPSFSLNPSSSSYPT
STSSPSFLHSHYTPSSTSFHTNPVYSMKQEQLPSNQIPQIDGFNNVNNFTDNERQNHNLN
SSGAHRRSSSCSLLEDVFEEAEALASGGRGRPPKRRQLTASLPNHNNNTNNNDNFFSVSF
GHYDSSDNLCSLQGKTKTTYNTSNLNYSSLQVKCKMFMIKTQI*
>AtMYB121
MLDWGVQGHHQKHDHDIYQQQHQQQGCRKGPWTLEEDKLLAEYVTSHGEGRWSTVAK
CAGLNRSGKSCRLRWVNYLRPGLKRGQITPQEEGIILELHSLWGNKWSTIARYLPGRTDN
EIKNYWRTHYKKNQKSSSKQDKVKKSLSRKQQQVDLKPQPQAQSENHQSQLVSQDHMN
IDNDHNIASSLYYPTSVFDDKLYMPQSVATTSSDHSMIDEGHLWGSLWNLDEDDPHSFGG
GSGQGTAADIDEKFPDSGIEAPSCGSGDYSYTGVYMGGYIF*
>AtMYB122
MVRTPCCRAEGLKKGAWTQEEDQKLIAYVQRHGEGGWRTLPDKAGLKRCGKSCRLRWA
NYLRPDIKRGEFSQDEEDSIINLHAIHGNKWSAIARKIPRRTDNEIKNHWNTHIKKCLVKK
GIDPLTHKSLLDGAGKSSDHSAHPEKSSVHDDKDDQNSNNKKLSGSSSARFLNRVANRFG
HRINHNVLSDIIGSNGLLTSHTTPTTSVSEGERSTSSSSTHTSSNLPINRSITVDATSLSS
STFSDSPDPCLYEEIVGDIEDMTRFSSRCLSHVLSHEDLLMSVESCLENTSFMREITMIF
QEDKIETTSFNDSYVTPINEVDDSCEGIDNYFG*

>AtMYB123
MKTRSLEITSPLTAKANGALSLTKLVLISLSTDPSLYLLRTIIVLAHIKGLKRCGKSCRL
RWKNYLRPGIKRGNISSDEEELIIRLHNLLGNRWSLIAGRLPGRTDNEIKNHWNSNLRKR
LPKTQTKQPKRIKHSTNNENNVCVIRTKAIRCSKTLLFSDLSLQKKSSTSPLPLKEQEMD
QGGSSLMGDLEFDFDRIHSEFHFPDLMDFDGLDCGNVTSLVSSNEILGELVPAQGNLDLN
RPFTSCHHRGDDEDWLRDFTC*
>AtMYB124
MEDTKKKKKKNINNNQDSKKKERHIVTWSQEEDVILREQITLHGTENWAIIASKFKDKST
RQCRRRWYTYLNSDFKRGGWSPEEDMLLCEAQRVFGNRWTEIAKVVSGRTDNAVKNRF
TTLCKKRAKHEAMTKDSNSNTKRMLFLDGISTPRKSENETPIAKKLKRSHILDLTEISNYG
RAEACVNQQIRSPFSVLARNATGIDSLEEQNQTSNVNESDGEGMFLKKDDPKVTALMQQ
AELLSSLAQKVNADNTEQSMENAWKVLQDFLNKGKENDLFRYGIPDIDFKIEEFKDLIED
LRSGYEDNQLSWRQPDLHDSPASSEYSSGSTIMVDQSGDKTQPFSADTQTEHKQVGEELL
VPKNPDENMPISGEEKFSSPIQVTPLFRSLADGIPSPQFSESERSFLLKTLGIESSSPCPSA
NPSKPPPCKRVLLHSL*
>AtMYB125
MRKMEAKKEEIKKGPWKAEEDEVLINHVKRYGPRDWSSIRSKGLLQRTGKSCRLRWVN
KLRPNLKNGCKFSADEERTVIELQSEFGNKWARIATYLPGRTDNDVKNFWSSRQKRLARI
LHNSSDASSSSFNPKSSSSHRLKGKNVKPIRQSSQGFGLVEEEVTVSSSCSQMVPYSSDQV
GDEVLRLPDLGVKLEHQPFAFGTDLVLAEYSDSQNDANQQAISPFSPESRELLARLDDPFY
YDILGPADSSEPLFALPQPFFEPSPVPRRCRHVSKDEEADVFLDDFPADMFDQVDPIPSP
>AtMYB3R1
MKREMKAPTTPLESLQGDLKGKQGRTSGPARRSTKGQWTPEEDEVLCKAVERFQGKNW
KKIAECFKDRTDVQCLHRWQKVLNPELVKGPWSKEEDNTIIDLVEKYGPKKWSTISQHLP
GRIGKQCRERWHNHLNPGINKNAWTQEEELTLIRAHQIYGNKWAELMKFLPGRSDNSIKN
HWNSSVKKKLDSYYASGLLDQCQSSPLIALQNKSIASSSSWMHSNGDEGSSRPGVDAEES
ECSQASTVFSQSTNDLQDEVQRGNEEYYMPEFHSGTEQQISNAASHAEPYYPSFKDVKIV
VPEISCETECSKKFQNLNCSHELRTTTATEDQLPGVSNDAKQDRGLELLTHNMDNGGKNQ
ALQQDFQSSVRLSDQPFLSNSDTDPEAQTLITDEECCRVLFPDNMKDSSTSSGEQGRNMV
DPQNGKGSLCSQAAETHAHETGKVPALPWHPSSSEGLAGHNCVPLLDSDLKDSLLPRNDS
NAPIQGCRLFGATELECKTDTNDGFIDTYGHVTSHGNDDNGGFPEQQGLSYIPKDSLKLVP
LNSFSSPSRVNKIYFPIDDKPAEKDKGALCYEPPRFPSADIPFFSCDLVPSNSDLRQEYSP
FGIRQLMISSMNCTTPLRLWDSPCHDRSPDVMLNDTAKSFSGAPSILKKRHRDLLSPVLD
RRKDKKLKRAATSSLANDFSRLDVMLDEGDDCMTSRPSESPEDKNICASPSIARDNRNCA
SARLYQEMIPIDEEPKETLESGGVTSMQNENGCNDGGASAKNDQETSGSFFELRLCSPGM
TRARPDNKVNASAKDLSNQHKISLGDFPTEEMSSEPLCTVDSIPLSAIDKTNTAETSFDI
ENFNIFDGTPFRKLLDTPSPWKSPLLFGSFLQSPKLPPEITFEDIGCFMSPGERSYDAIG
LMKHLSEHSATAYADALEVLGNDTPESILKKRQLNKSIQGKENQHQPHDQLGNRSQVECR
ALDFSDCGTPGKAKVPSASPGGYSSPSSYLLKSCR*
>AtMYB3R2
MTESIDLNRSESESDNNTDDVTPIFAIDDSSKGRVSGPTRRSTKGGWTAEEDQILTNVVK
KYQGRNWKRIAECLPGSEENRRNDVQCQHRWLKVLDPSLQKGAWKKEEDELLSELVKD
YMENDRPPWSKISKELPGRIGKQCRERWHNHLNPTIIKSPWTREEELILVQAQRGNGNKW
AEIAKLLPGRTENNIKNHWNCSVKKRLEQFPSNLFSGVVYGSKPSSGFEYNFFNQRNTMV

ESCITSQIKEAAKSPQRDFLDLTLGLNWRSISSSTSSLRGEESVSSSVDSVCARLNACLETP
QNSNNDTVCVKEVREMKERLRMAARTFDTPSIISKTSSPASGLKRLRQKYDTPFPTDARS
HMSSEEDHSVSASPSSKYRFVKRNTCSGSKPLERRLDFDFLLWDEHGRRNGIVNFSVRIL
PQKSDLKSGLVRPFWLR*
>AtMYB3R3
MIAFLGFLCFLSKSGSFFSFIEMMDLQEETGEVKIEDQCVENKQSTPASCSSVSEGSAGS
SHKSPTIASPATVSPTHRYLGRTSGPIRRAKGGWTPEEDETLRQAVDTFKGKSWKNIAKS
FPDRTEVQCLHRWQKVLNPDLIKGPWTHEEDEKIVELVEKYGPAKWSIIAQSLPGRIGKQ
CRERWHNHLNPDINKDAWTTEEEVALMNAHRSHGNKWAEIAKVLPGRTDNAIKNHWNS
SLKKKSEFYLLTGRLPPPTTTRNGVPDSVTKRSSSAQKRVFGSVAQTSSVTTDVNNLAEDG
NGQINSSVPVEEVVAASRMTSLNEYARSPQLPNPEPLPENGGAANNGYHLYYTPQIDYYR
ASEVDTQRMYGNECGCSPSASPVSFFTPPPCRNVHSNGSTPRSPESYLREAGRTYPNTPSI
FRKRRPRVVVQDNNNAKKTDEAKEVDQKVNDGKDSSEIQNNGSNAYNLSPPYRIRSKRT
AVFKSRQLEFISREEEKADDETKSSEKDMLIDGDSQLLG*
>AtMYB3R-4
MEAESSTPQERIPKLRHGRTSGPARRSTRGQWTAEEDEILRKAVHSFKGKNWKKIAEYFK
DRTDVQCLHRWQKVLNPELVKGPWTKEEDEMIVQLIEKYGPKKWSTIARFLPGRIGKQC
RERWHNHLNPAINKEAWTQEEELLLIRAHQIYGNRWAELTKFLPGRSDNGIKNHWHSSVK
KKLDSYMSSGLLDQYQAMPLAPYERSSTLQSTFMQSNIDGNGCLNGQAENEIDSRQNSS
MVGCSLSARDFQNGTINIGHDFHPCGNSQENEQTAYHSEQFYYPELEDISVSISEVSYDME
DCSQFPDHNVSTSPSQDYQFDFQELSDISLEMRHNMSEIPMPYTKESKESTLGAPNSTLNI
DVATYTNSANVLTPETECCRVLFPDQESEGHSVSRSLTQEPNEFNQVDRRDPILYSSASD
RQISEATKSPTQSSSSRFTATAASGKGTLRPAPLIISPDKYSKKSSGLICHPFEVEPKCT
TNGNGSFICIGDPSSSTCVDEGTNNSSEEDQSYHVNDPKKLVPVNDFASLAEDRPHSLPK
HEPNMTNEQHHEDMGASSSLGFPSFDLPVFNCDLLQSKNDPLHDYSPLGIRKLLMSTMTC
MSPLRLWESPTGKKTLVGAQSILRKRTRDLLTPLSEKRSDKKLEIDIAASLAKDFSRLDV
MFDETENRQSNFGNSTGVIHGDRENHFHILNGDGEEWSGKPSSLFSHRMPEETMHIRKSL
EKVDQICMEANVREKDDSEQDVENVEFFSGILSEHNTGKPVLSTPGQSVTKAEKAQVSTP
RNQLQRTLMATSNKEHHSPSSVCLVINSPSRARNKEGHLVDNGTSNENFSIFCGTPFRRG
LESPSAWKSPFYINSLLPSPRFDTDLTIEDMGYIFSPGERSYESIGVMTQINEHTSAFAA
FADAMEVSISPTNDDARQKKELDKENNDPLLAERRVLDFNDCESPIKATEEVSSYLLKGC
R*
>AtMYB3R-5
MSSSSNPPVCSPEKEERSEMKIEIQCMENKQPLAASCSSASEGSGCFFLKSPEIATPATV
SSFPRRTSGPMRRAKGGWTPEEDETLRRAVEKYKGKRWKKIAEFFPERTEVQCLHRWQK
VLNPELVKGPWTQEEDDKIVELVKKYGPAKWSVIAKSLPGRIGKQCRERWHNHLNPGIRK
DAWTVEEESALMNSHRMYGNKWAEIAKVLPGRTDNAIKNHWNSSLKKKLEFYLATGNL
PPPASKFIVLKDIADGDRDSKQSSATKPFKDSDSLTQTSSGNTDSNEVGRDHFDSSSALLEE
VAASRRIGVNEYACSPVEYKPQLPNLEPISEEVRINSKAYFERSIQRKVENGFGTPKHGNL
YYKSPLDYYFPSEADLQHMYGYECGCSPGAASPVSLMTTPCNKDSGLTATRSPESFLREA
ARTFPNTPSIFRKRRKVVLAAKTDAVVVVNGVVKEVDRKEESKDMRKSLLLETTDNCSD
DEELGLNGNAFNLSPPYRLRAKRTAVIKSRQLEFTSEKEKQPDNEIEFTSAKEKQPDNEIK
TSEEDKPV*
>AtMYB4R1

MNRNSLYEADDDDDDDEEDDIGEDLEDLRRACMVSDVNSDQFASKTGSIEPEGVGGGEIP
SDSENEDDFEMLRTIKSQLASSKDAGRSSGPPMGLSLLSDSESEDDFEMIRSIKSQLSLS
MDVSLPPIGLSDDEEDDAFETLRAIRRRFSAYKNFDSEGKFMNDSHGKKKQVHNSDNEPS
SEILSRSNTCESFPDHGKSVVTVPDSEDVQDGHMPAASSSFPEAARAFVDAIRRNRAYQK
FLRGKLAEIEATIEQNEKHKKNVRIVKDFQASCKRITKLALCQRKDPRVELISTRKSGPC
DSSEVIGPCDSFEGNDKKISPLTLGPAENPCVENYRMALEKYPISVKRRKWSTEENKNLA
KGLKQEVQKILLSEAIERSSDLEGATYDIDTINESIGNLEITPEMIRQFLPKINWDSLDI
KDRSAAECEARWMSSEDPLINHGPWTAAEDKNLLRTIEQTSLTDWVDIAVSLGTNRTPFQ
CLARYQRSLNPSILKKEWTAEEDDQLRTAVELFGEKDWQSVANVLKGRTGTQCSNRWKK
SLRPTRKGTWSLEEDKRVKVAVTLFGSQNWHKISQFVPGRTQTQCRERWLNCLDPKVNR
GKWTEEEDEKLREAIAEHGYSWSKVATNLSCRTDNQCLRRWKRLYPHQVALLQEARRLQ
KEASVGNFVDRESERPALVTSPILALPDISLEPEPDSVALKKKRKAKQKKSDAERQPKRRR
KGLKNCSGDVCRQENETVCENEPNNGGEERMLALECHDEIQDNAKEKPKQRRKSVAETV
CENEPNNGGEERMLALECDNEIQDNAKEKRKRRRKSVAETSNNTTGLKKLTPRRRKISAV
VPIKNQDAPN*
