# Supplementary material for: Genome-Wide Identification and Expression Analysis of MYB Transcription Factor Family in Response to Various Abiotic Stresses in Coconut (Cocos nucifera L.)
Source: Int J Mol Sci. 2024 Sep 18;25(18):10048. doi: 10.3390/ijms251810048 (PMC11432468; doi:10.3390/ijms251810048)
Supplement: Supplementary file 1 [file ijms-25-10048-s001.zip › ijms-3189138-supplementary tables S1-S10.pdf]

Genome-wide identification, and expression analysis of MYB transcription factor family in response to various abiotic stresses in Coconut (*Cocos nucifera* L.)

Table S1 Physicochemical properties and subcellular localization of *MYB* family in *Cocos nucifera*.

Gene name	Gene ID	Chromosome location	Amion acid length	Molecular weight (kDa)	Isoelectric point	Instability index	Grand average of hydropathicity	Subcellular localization
CnMYB1	*AZ01G0003390.1*	Chr01(-):13833330-13838018	845	93.07	4.86	51.15	-0.466	nucleus
CnMYB2	*AZ01G0003870.1*	Chr01(-):16464252-16465288	179	20.87	5.89	44.01	-1.126	nucleus
CnMYB3	*AZ01G0007770.1*	Chr01(-):45932408-45933777	271	31.42	5.87	55.23	-0.792	nucleus
CnMYB4	*AZ01G0009680.1*	Chr01(+):52018608-52019888	328	37.07	9.69	67.53	-0.813	nucleus
CnMYB5	*AZ01G0010240.1*	Chr01(-):53881191-53882298	253	28.19	8.46	60.58	-0.675	nucleus
CnMYB6	*AZ01G0016690.1*	Chr01(+):187112576-187114640	411	45.08	6.57	52.82	-0.747	nucleus
CnMYB7	*AZ01G0016790.1*	Chr01(+):187486089-187488486	308	34.00	6.34	44.00	-0.584	nucleus
CnMYB8	*AZ01G0016910.1*	Chr01(+):188327264-188331367	456	50.89	6.77	66.83	-0.816	nucleus
CnMYB9	*AZ01G0017520.1*	Chr01(-):190390123-190401964	324	35.22	5.72	44.89	-0.263	nucleus
CnMYB10	*AZ01G0019330.1*	Chr01(-):197399720-197400938	306	34.52	7.04	61.98	-0.708	nucleus
CnMYB11	*AZ01G0019540.1*	Chr01(-):198025931-198026918	238	26.77	7.04	52.20	-0.687	nucleus
CnMYB12	*AZ01G0020080.1*	Chr01(+):199479780-199481766	270	30.58	5.76	50.51	-0.684	nucleus
CnMYB13	*AZ01G0021730.1*	Chr01(-):204301640-204330550	1049	115.74	5.08	54.78	-0.668	nucleus
CnMYB14	*AZ01G0022480.1*	Chr01(+):206502002-206505879	89	10.65	7.87	64.77	-0.896	cytosol/nucleus
CnMYB15	*AZ01G0024000.1*	Chr01(-):210652163-210658159	349	37.73	8.58	61.80	-0.622	chloroplast
CnMYB16	*AZ01G0024010.1*	Chr01(+):210676845-210678076	341	37.53	6.27	58.67	-0.991	nucleus
CnMYB17	*AZ01G0024190.1*	Chr01(-):211226853-211228174	288	31.21	6.44	57.85	-0.523	nucleus
CnMYB18	*AZ02G0026630.1*	Chr02(+):5079306-5082960	256	29.24	6.87	48.62	-0.764	nucleus

CnMYB19	*AZ02G0029800.1*	Chr02(-):70429517-70469906	595	65.35	5.49	53.05	-0.578	nucleus
CnMYB20	*AZ02G0032720.1*	Chr02(-):139339495-139357236	556	62.28	8.61	50.54	-0.752	nucleus
CnMYB21	*AZ02G0032900.1*	Chr02(+):140237541-140273009	472	53.24	8.65	46.83	-0.516	nucleus
CnMYB22	*AZ02G0032920.1*	Chr02(+):140409484-140412952	347	36.51	6.02	56.00	-0.505	nucleus
CnMYB23	*AZ02G0034130.1*	Chr02(+):145967480-145969051	332	36.62	8.08	57.91	-0.667	nucleus
CnMYB24	*AZ02G0034430.1*	Chr02(-):147247693-147248619	279	30.69	5.21	58.14	-0.106	nucleus
CnMYB25	*AZ02G0036210.1*	Chr02(-):156222417-156230015	287	33.27	9.49	65.84	-0.713	chloroplast
CnMYB26	*AZ02G0037050.1*	Chr02(-):158833961-158849713	720	79.86	6.44	50.00	-0.848	nucleus
CnMYB27	*AZ02G0037500.1*	Chr02(+):160996432-161025399	316	33.78	6.8	37.54	-0.448	nucleus
CnMYB28	*AZ02G0038740.1*	Chr02(-):165717461-165719432	292	33.49	8.62	53.98	-0.966	nucleus
CnMYB29	*AZ02G0038750.1*	Chr02(-):165752959-165755001	293	33.50	7.64	54.39	-0.888	nucleus
CnMYB30	*AZ02G0041060.1*	Chr02(+):172138365-172140693	326	36.01	5.66	63.27	-0.628	cytosol
CnMYB31	*AZ02G0041470.2*	Chr02(+):173228682-173254761	317	34.28	7.85	50.98	-0.494	nucleus
CnMYB32	*AZ02G0043140.1*	Chr02(+):178009182-178010912	354	39.39	4.78	52.85	-0.760	nucleus
CnMYB33	*AZ02G0044370.1*	Chr02(-):180677807-180679239	236	27.01	9.54	56.77	-0.683	nucleus
CnMYB34	*AZ02G0046620.1*	Chr02(-):186504346-186506185	261	29.47	6.19	53.92	-0.680	nucleus
CnMYB35	*AZ03G0049700.1*	Chr03(-):6788696-6789703	192	22.10	9.71	74.03	-0.346	nucleus
CnMYB36	*AZ03G0051100.1*	Chr03(-):10622201-10623825	85	10.40	9.13	83.14	-1.036	nucleus
CnMYB37	*AZ03G0053140.1*	Chr03(-):16070691-16101884	218	23.96	4.65	51.68	-0.806	nucleus
CnMYB38	*AZ03G0053510.1*	Chr03(+):16909721-16911582	315	34.22	5.87	59.88	-0.495	nucleus
CnMYB39	*AZ03G0053570.1*	Chr03(-):17078566-17080421	267	28.80	9.45	57.60	-0.499	nucleus
CnMYB40	*AZ03G0056230.1*	Chr03(+):23677396-23678062	119	13.47	9.69	69.59	-0.100	nucleus
CnMYB41	*AZ03G0056240.1*	Chr03(+):23725442-23725675	77	8.91	6.56	54.95	-1.084	nucleus
CnMYB42	*AZ03G0056250.1*	Chr03(+):23738324-23738557	77	8.93	8.03	60.77	-0.819	cytosol
CnMYB43	*AZ03G0056400.1*	Chr03(+):24114072-24116239	201	23.06	6.08	53.32	-0.863	nucleus
CnMYB44	*AZ03G0056410.1*	Chr03(+):24146805-24149377	194	22.37	8.73	59.12	-0.863	nucleus

CnMYB45	*AZ03G0057640.1*	Chr03(-):27221728-27224040	287	31.13	9.05	60.55	-0.631	cytosol
CnMYB46	*AZ03G0057760.1*	Chr03(-):27573199-27574176	325	36.90	9.27	65.42	-0.705	nucleus
CnMYB47	*AZ03G0058150.1*	Chr03(+):28556914-28558454	319	36.27	5.90	58.59	-0.693	nucleus
CnMYB48	*AZ03G0059780.1*	Chr03(+):33336703-33357983	875	98.03	6.19	45.40	-0.240	nucleus
CnMYB49	*AZ03G0062230.1*	Chr03(-):40424310-40426616	141	15.80	4.70	50.93	-0.613	chloroplast
CnMYB50	*AZ03G0062380.1*	Chr03(+):40985587-40988374	507	54.10	6.50	69.95	-0.498	nucleus
CnMYB51	*AZ03G0071770.1*	Chr03(+):162565632-162583667	396	44.54	4.68	54.94	-0.662	nucleus
CnMYB52	*AZ03G0073080.1*	Chr03(+):174787899-174789486	307	34.52	7.08	68.13	-0.673	nucleus
CnMYB53	*AZ04G0079220.1*	Chr04(-):97993932-97995421	293	32.97	5.19	64.07	-0.603	nucleus
CnMYB54	*AZ04G0080380.1*	Chr04(-):126777406-126782031	194	23.10	6.80	76.03	-1.213	nucleus
CnMYB55	*AZ04G0081030.1*	Chr04(-):131727558-131728430	228	25.49	5.52	47.63	-0.563	nucleus
CnMYB56	*AZ04G0081040.1*	Chr04(+):131731643-131733072	272	30.15	5.55	58.75	-0.681	nucleus
CnMYB57	*AZ04G0081050.1*	Chr04(+):131783632-131784610	257	29.51	8.90	50.19	-0.758	nucleus
CnMYB58	*AZ04G0082970.1*	Chr04(+):141249977-141252541	405	44.12	6.40	49.70	-0.695	nucleus
CnMYB59	*AZ04G0083170.1*	Chr04(+):141571882-141574172	306	33.74	6.90	48.11	-0.562	nucleus
CnMYB60	*AZ04G0083490.1*	Chr04(+):142634277-142638377	474	52.62	5.72	50.68	-0.800	nucleus
CnMYB61	*AZ04G0084410.1*	Chr04(-):145197993-145199241	321	35.34	6.59	50.52	-0.359	nucleus
CnMYB62	*AZ04G0088200.1*	Chr04(+):155949457-155951501	296	33.84	5.76	60.34	-0.752	nucleus
CnMYB63	*AZ04G0090630.1*	Chr04(-):162405299-162437181	1096	120.83	5.38	49.91	-0.627	nucleus
CnMYB64	*AZ04G0091560.1*	Chr04(+):164641437-164674575	154	17.90	9.10	55.40	-0.332	chloroplast
CnMYB65	*AZ04G0093340.1*	Chr04(-):169823425-169829367	352	38.10	9.18	58.96	-0.556	chloroplast
CnMYB66	*AZ04G0093350.1*	Chr04(+):169869099-169870361	347	37.86	6.15	59.74	-0.772	nucleus
CnMYB67	*AZ04G0093700.1*	Chr04(-):171333802-171335225	288	31.33	6.51	50.47	-0.468	nucleus
CnMYB68	*AZ04G0093750.1*	Chr04(-):171477740-171479633	297	32.84	9.89	75.08	-0.670	nucleus
CnMYB69	*AZ05G0096990.1*	Chr05(+):597449-598832	203	22.16	9.61	54.11	-0.280	nucleus
CnMYB70	*AZ05G0097330.1*	Chr05(-):2039321-2040840	408	45.05	7.56	58.78	-0.663	nucleus

CnMYB71	*AZ05G0097510.1*	Chr05(+):2546589-2548499	102	11.65	4.21	56.69	-0.784	cytosol
CnMYB72	*AZ05G0098140.1*	Chr05(+):3887463-3890020	274	31.12	5.68	57.74	-0.775	nucleus
CnMYB73	*AZ05G0098330.1*	Chr05(+):4239558-4240982	302	35.09	9.59	56.19	-1.064	nucleus
CnMYB74	*AZ05G0098350.1*	Chr05(+):4286500-4295662	1141	126.92	5.10	51.98	-0.788	nucleus
CnMYB75	*AZ05G0098830.1*	Chr05(+):5354278-5357045	325	36.50	5.71	42.45	-0.747	nucleus
CnMYB76	*AZ05G0101920.1*	Chr05(+):13070896-13072696	352	39.53	6.33	52.20	-0.741	nucleus
CnMYB77	*AZ05G0107400.1*	Chr05(+):28734846-28736088	321	36.12	6.09	57.81	-0.663	nucleus
CnMYB78	*AZ05G0109860.1*	Chr05(-):36421559-36422674	371	40.14	8.90	74.14	-0.579	nucleus
CnMYB79	*AZ05G0115030.1*	Chr05(-):69447503-69449434	315	34.89	6.71	49.36	-0.637	nucleus
CnMYB80	*AZ05G0119730.1*	Chr05(+):165160917-165162020	253	28.31	9.05	56.76	-0.642	nucleus
CnMYB81	*AZ05G0120530.1*	Chr05(-):167968900-167970088	307	34.94	8.94	55.46	-0.764	nucleus
CnMYB82	*AZ05G0121500.1*	Chr05(+):170905297-170941923	334	38.54	5.14	70.15	-0.843	cytosol
CnMYB83	*AZ05G0122770.1*	Chr05(+):174323871-174326893	440	47.72	6.19	54.19	-0.535	nucleus
CnMYB84	*AZ06G0123300.1*	Chr06(-):1336509-1338506	458	50.50	6.85	67.54	-0.618	nucleus
CnMYB85	*AZ06G0123480.1*	Chr06(-):1971046-1995195	931	102.62	9.45	40.11	-0.302	chloroplast
CnMYB86	*AZ06G0123810.1*	Chr06(+):2714743-2721185	393	41.54	7.10	60.02	-0.478	nucleus
CnMYB87	*AZ06G0124680.1*	Chr06(-):4842222-4856279	587	63.53	9.00	54.27	-0.553	nucleus
CnMYB88	*AZ06G0125260.1*	Chr06(+):5863887-5864985	251	28.88	5.54	59.90	-0.731	nucleus
CnMYB89	*AZ06G0126460.1*	Chr06(-):8640687-8641406	239	25.64	7.72	63.04	-0.578	nucleus
CnMYB90	*AZ06G0128770.1*	Chr06(-):15014058-15017020	381	41.83	6.65	53.01	-0.721	nucleus
CnMYB91	*AZ06G0128900.1*	Chr06(-):15357825-15359269	338	38.42	6.27	51.04	-0.699	nucleus
CnMYB92	*AZ06G0129250.2*	Chr06(-):17159175-17165894	555	59.79	4.83	59.61	-0.528	nucleus
CnMYB93	*AZ06G0129420.1*	Chr06(-):17633731-17640626	660	75.72	6.67	58.59	-0.919	cytosol
CnMYB94	*AZ06G0133920.1*	Chr06(-):130418539-130418931	130	14.95	4.36	56.16	-1.050	peroxisome
CnMYB95	*AZ06G0133930.1*	Chr06(-):130463410-130466100	105	12.35	4.69	66.98	-0.594	cytosol/nucleus
CnMYB96	*AZ06G0133940.1*	Chr06(-):130555070-130555462	130	14.91	4.30	60.19	-1.028	peroxisome

CnMYB97	*AZ06G0133950.1*	Chr06(-):130576679-130577071	130	14.82	4.39	63.37	-1.027	peroxisome
CnMYB98	*AZ06G0133960.1*	Chr06(-):130582911-130583303	130	14.97	4.36	54.68	-1.025	peroxisome
CnMYB99	*AZ06G0134240.1*	Chr06(+):134701095-134701463	122	14.19	4.25	65.73	-0.962	cytosol/nucleus
CnMYB100	*AZ06G0137370.1*	Chr06(-):153771471-153774795	396	43.15	4.67	55.02	-0.756	nucleus
CnMYB101	*AZ06G0138250.1*	Chr06(+):156356691-156357949	290	32.61	5.33	63.77	-0.691	nucleus
CnMYB102	*AZ06G0138850.2*	Chr06(+):158355970-158358575	368	40.82	6.95	56.95	-0.858	nucleus
CnMYB103	*AZ06G0140810.1*	Chr06(+):162984687-162995131	598	66.82	6.93	50.92	-0.432	nucleus
CnMYB104	*AZ06G0141040.1*	Chr06(+):163454326-163455808	266	28.82	8.68	65.19	-0.583	nucleus
CnMYB105	*AZ06G0141580.2*	Chr06(+):164759343-164771387	1672	182.60	6.22	53.70	-0.588	nucleus
CnMYB106	*AZ06G0141920.1*	Chr06(+):165453989-165470060	334	37.54	9.38	47.31	-0.321	chloroplast
CnMYB107	*AZ06G0142560.1*	Chr06(-):166804719-166805763	243	27.85	5.67	48.51	-0.823	nucleus
CnMYB108	*AZ07G0145430.1*	Chr07(+):3668895-3670718	332	37.18	6.09	60.31	-0.679	nucleus
CnMYB109	*AZ07G0150740.1*	Chr07(-):16063935-16065020	361	39.36	9.30	69.46	-0.580	nucleus
CnMYB110	*AZ07G0152280.1*	Chr07(+):21110607-21111981	331	36.12	5.83	29.93	-0.669	nucleus
CnMYB111	*AZ07G0156840.1*	Chr07(+):141365413-141366339	191	22.01	7.04	59.57	-1.002	nucleus
CnMYB112	*AZ08G0163640.1*	Chr08(-):5517439-5519078	94	11.14	9.03	70.89	-0.954	nucleus
CnMYB113	*AZ08G0163650.1*	Chr08(-):5549377-5550910	94	11.08	9.50	72.60	-0.921	nucleus
CnMYB114	*AZ08G0163660.1*	Chr08(-):5577347-5578801	82	10.01	8.97	72.60	-1.051	nucleus
CnMYB115	*AZ08G0165150.1*	Chr08(+):9186527-9187832	256	28.04	5.27	49.70	-0.511	nucleus
CnMYB116	*AZ08G0165210.1*	Chr08(-):9405439-9407326	282	30.17	8.98	57.77	-0.432	nucleus
CnMYB117	*AZ08G0167160.1*	Chr08(+):13739489-13740373	83	9.60	9.91	65.30	-0.924	cytosol
CnMYB118	*AZ08G0167270.1*	Chr08(+):14146026-14148331	198	22.73	5.19	58.87	-1.022	nucleus
CnMYB119	*AZ08G0168470.1*	Chr08(+):17758666-17760646	319	36.08	5.81	55.13	-0.703	nucleus
CnMYB120	*AZ08G0171230.1*	Chr08(-):34145883-34148668	68	7.81	4.29	70.10	-0.824	nucleus
CnMYB121	*AZ08G0171390.1*	Chr08(+):36351014-36353747	462	49.43	7.20	73.54	-0.484	nucleus
CnMYB122	*AZ08G0172050.1*	Chr08(+):59539092-59540635	328	36.86	6.39	60.47	-0.590	nucleus

CnMYB123	*AZ08G0172480.1*	Chr08(+):72473110-72499411	1071	120.26	9.36	51.56	-0.651	nucleus
CnMYB124	*AZ08G0174510.1*	Chr08(+):135383183-135384866	283	31.54	5.29	52.46	-0.635	nucleus
CnMYB125	*AZ09G0179830.1*	Chr09(-):2280513-2281255	211	24.71	5.30	76.41	-1.248	nucleus
CnMYB126	*AZ09G0179960.1*	Chr09(-):2620195-2621373	326	36.29	8.69	47.32	-0.648	nucleus
CnMYB127	*AZ09G0184720.1*	Chr09(+):29901896-29904065	316	34.74	5.77	55.73	-0.643	nucleus
CnMYB128	*AZ09G0187760.1*	Chr09(+):133903359-133904524	326	35.86	6.19	48.52	-0.452	nucleus
CnMYB129	*AZ09G0189840.1*	Chr09(-):145193973-145195556	322	35.69	6.56	54.75	-0.678	nucleus
CnMYB130	*AZ09G0190250.1*	Chr09(-):146272409-146274872	396	43.51	6.79	54.61	-0.727	nucleus
CnMYB131	*AZ09G0190430.1*	Chr09(+):146855793-146858799	397	43.41	6.34	62.29	-0.699	nucleus
CnMYB132	*AZ09G0191600.1*	Chr09(+):149947388-149969648	775	85.23	6.25	53.29	-0.780	nucleus
CnMYB133	*AZ09G0191610.1*	Chr09(+):149992112-149994766	294	33.26	6.96	57.54	-0.695	nucleus
CnMYB134	*AZ09G0192100.1*	Chr09(+):151208680-151210200	236	27.63	8.49	68.29	-1.042	nucleus
CnMYB135	*AZ09G0193600.1*	Chr09(-):154854788-154856254	95	10.81	9.10	73.36	-0.946	cytosol
CnMYB136	*AZ09G0194060.1*	Chr09(-):155956867-155974279	408	46.15	4.88	51.93	-0.651	nucleus
CnMYB137	*AZ09G0194450.1*	Chr09(-):157433338-157436501	445	48.89	5.97	55.07	-0.474	nucleus
CnMYB138	*AZ10G0195710.1*	Chr10(+):11543821-11547228	258	29.28	5.94	43.29	-0.821	nucleus
CnMYB139	*AZ10G0197820.1*	Chr10(-):94077340-94079704	293	32.58	6.14	42.96	-0.646	nucleus
CnMYB140	*AZ10G0198830.1*	Chr10(-):119122076-119123742	285	32.26	8.54	66.01	-0.789	nucleus
CnMYB141	*AZ10G0200550.1*	Chr10(-):131365966-131366969	233	27.41	6.12	66.29	-1.057	nucleus
CnMYB142	*AZ10G0202500.1*	Chr10(-):138669327-138670955	287	33.17	9.12	53.56	-1.021	nucleus
CnMYB143	*AZ10G0203990.1*	Chr10(-):143211773-143217503	312	34.28	8.98	55.84	-0.681	chloroplast
CnMYB144	*AZ10G0204040.1*	Chr10(+):143284720-143286626	338	37.23	6.12	56.99	-0.627	chloroplast
CnMYB145	*AZ10G0205290.1*	Chr10(-):145582180-145583799	360	39.74	6.06	41.48	-0.573	nucleus
CnMYB146	*AZ10G0205400.2*	Chr10(+):145763948-145765904	366	40.48	4.86	57.09	-0.813	nucleus
CnMYB147	*AZ10G0206030.1*	Chr10(-):147248691-147249845	219	25.20	8.99	59.58	-0.760	nucleus
CnMYB148	*AZ11G0208510.1*	Chr11(+):15098848-15102339	362	40.06	6.92	49.03	-0.412	nucleus

CnMYB149	*AZ11G0208640.1*	Chr11(-):17494222-17497193	245	27.57	5.56	61.04	-0.711	nucleus
CnMYB150	*AZ11G0211820.1*	Chr11(+):115710008-115713446	299	32.41	9.43	56.89	-0.539	nucleus
CnMYB151	*AZ11G0211900.1*	Chr11(+):115967108-115969473	304	34.11	5.20	51.68	-0.604	nucleus
CnMYB152	*AZ11G0212140.1*	Chr11(+):117347773-117359654	589	65.06	5.69	47.39	-0.346	nucleus
CnMYB153	*AZ11G0212760.2*	Chr11(-):119850555-119885427	352	37.59	6.92	41.81	-0.432	nucleus
CnMYB154	*AZ11G0212950.2*	Chr11(+):120766790-120794757	770	83.45	6.76	53.34	-0.669	nucleus
CnMYB155	*AZ11G0214040.1*	Chr11(-):125038269-125039612	237	27.02	8.93	65.18	-0.756	nucleus
CnMYB156	*AZ11G0215700.1*	Chr11(-):128797370-128801360	511	55.90	5.96	58.39	-0.667	nucleus
CnMYB157	*AZ11G0215810.1*	Chr11(-):128904617-128909383	458	50.09	4.76	56.22	-0.294	nucleus
CnMYB158	*AZ11G0217040.1*	Chr11(-):131537295-131555690	521	57.26	9.68	54.89	-0.566	nucleus
CnMYB159	*AZ11G0217800.1*	Chr11(-):132791543-132799587	541	60.54	8.23	56.77	-0.804	nucleus
CnMYB160	*AZ11G0217970.1*	Chr11(+):133026304-133028512	291	32.74	7.69	48.65	-0.726	nucleus
CnMYB161	*AZ11G0219380.1*	Chr11(+):135840780-135842444	300	33.61	8.59	56.51	-0.663	nucleus
CnMYB162	*AZ11G0220750.1*	Chr11(-):139336380-139338259	269	30.08	6.25	58.88	-0.728	nucleus
CnMYB163	*AZ12G0221060.1*	Chr12(+):900550-917656	179	20.21	9.04	45.97	-0.515	nucleus
CnMYB164	*AZ12G0227370.1*	Chr12(-):122235422-122236771	269	30.97	5.67	50.92	-0.680	nucleus
CnMYB165	*AZ12G0231220.1*	Chr12(+):136489013-136499477	512	57.13	9.10	66.28	-0.514	nucleus
CnMYB166	*AZ12G0231930.1*	Chr12(-):138276298-138277445	256	28.14	8.96	58.57	-0.685	nucleus
CnMYB167	*AZ13G0238110.1*	Chr13(-):59570836-59572305	291	32.58	5.48	52.91	-0.594	nucleus
CnMYB168	*AZ13G0239080.1*	Chr13(-):63422872-63461155	1128	124.97	9.30	54.04	-0.591	nucleus
CnMYB169	*AZ13G0239190.1*	Chr13(+):63823414-63825580	277	31.28	6.66	48.53	-0.649	nucleus
CnMYB170	*AZ13G0239200.1*	Chr13(+):63841499-63842546	273	31.18	8.32	48.17	-0.714	nucleus
CnMYB171	*AZ13G0240920.1*	Chr13(-):68546637-68547371	244	27.11	5.93	67.97	-0.777	nucleus
CnMYB172	*AZ13G0241860.1*	Chr13(-):70763899-70766059	300	33.41	4.76	26.92	-0.705	nucleus
CnMYB173	*AZ13G0242460.1*	Chr13(-):73263643-73264817	338	37.40	5.93	40.82	-0.452	nucleus
CnMYB174	*AZ13G0244680.1*	Chr13(-):82728938-82736618	316	34.29	7.23	51.06	-0.693	mitochondrion

CnMYB175	*AZ13G0244820.1*	Chr13(+):83141677-83170137	647	71.25	6.02	53.23	-0.315	nucleus
CnMYB176	*AZ13G0245410.1*	Chr13(-):84647521-84692624	752	82.65	6.18	52.82	-0.751	nucleus
CnMYB177	*AZ13G0245720.1*	Chr13(+):85691424-85700262	594	64.94	9.43	48.71	-0.434	nucleus
CnMYB178	*AZ13G0246320.1*	Chr13(-):86990450-86991272	230	26.54	9.12	70.13	-0.838	nucleus
CnMYB179	*AZ13G0248380.1*	Chr13(-):91397797-91407100	603	67.72	8.29	42.53	-0.718	nucleus
CnMYB180	*AZ13G0248440.1*	Chr13(+):91502517-91507371	321	35.99	8.64	43.00	-0.475	nucleus
CnMYB181	*AZ13G0249410.1*	Chr13(+):93639461-93640867	300	33.75	7.48	62.09	-0.653	nucleus
CnMYB182	*AZ13G0249550.1*	Chr13(-):94046956-94047549	143	16.58	9.75	56.26	-1.341	nucleus
CnMYB183	*AZ13G0250330.1*	Chr13(-):95909862-95911937	272	30.38	5.85	59.72	-0.532	nucleus
CnMYB184	*AZ14G0250600.1*	Chr14(+):299571-301917	424	46.22	6.36	58.08	-0.539	nucleus
CnMYB185	*AZ14G0250630.1*	Chr14(-):861533-863697	261	29.67	6.14	57.47	-0.758	nucleus
CnMYB186	*AZ14G0251180.1*	Chr14(-):35553804-35555334	409	45.77	7.58	57.46	-0.682	nucleus
CnMYB187	*AZ14G0255610.1*	Chr14(-):76416250-76421243	248	28.37	7.77	50.26	-0.825	nucleus
CnMYB188	*AZ14G0257350.1*	Chr14(+):81032743-81042351	541	59.14	4.94	56.29	-0.552	nucleus
CnMYB189	*AZ14G0257640.1*	Chr14(+):81759712-81761369	325	36.51	8.33	45.21	-0.764	nucleus
CnMYB190	*AZ14G0257750.1*	Chr14(+):82032143-82035309	375	41.34	6.04	55.50	-0.692	nucleus
CnMYB191	*AZ14G0259720.1*	Chr14(-):86585103-86586180	251	29.18	5.58	64.83	-0.813	nucleus
CnMYB192	*AZ14G0260160.1*	Chr14(+):87398423-87415624	588	64.46	8.75	54.75	-0.617	nucleus
CnMYB193	*AZ14G0260170.1*	Chr14(-):87417035-87434551	801	90.67	9.06	46.26	-0.552	nucleus
CnMYB194	*AZ14G0260820.1*	Chr14(-):89254262-89260443	387	40.82	5.22	62.61	-0.515	nucleus
CnMYB195	*AZ14G0261090.1*	Chr14(+):89823630-89824923	304	34.04	6.80	50.30	-0.723	nucleus
CnMYB196	*AZ14G0261200.1*	Chr14(+):90238943-90241408	455	49.73	7.65	66.86	-0.639	nucleus
CnMYB197	*AZ15G0261350.1*	Chr15(+):422231-426277	436	48.30	7.92	53.87	-0.608	nucleus
CnMYB198	*AZ15G0261610.1*	Chr15(+):1217810-1219220	316	35.94	6.41	57.21	-0.696	nucleus
CnMYB199	*AZ15G0262750.1*	Chr15(-):3747031-3755695	353	40.35	7.20	61.95	-0.469	nucleus
CnMYB200	*AZ15G0263060.1*	Chr15(-):4314312-4333571	296	33.07	8.17	51.78	-0.593	nucleus

CnMYB201	*AZ15G0263070.1*	Chr15(-):4402340-4403420	228	26.10	8.63	42.85	-0.770	nucleus
CnMYB202	*AZ15G0263830.1*	Chr15(-):5736140-5737655	382	41.87	5.42	63.69	-0.771	nucleus
CnMYB203	*AZ15G0263930.1*	Chr15(+):5903045-5905004	394	43.70	7.24	60.80	-0.709	nucleus
CnMYB204	*AZ15G0264170.1*	Chr15(+):6479956-6482359	315	34.77	7.63	53.82	-0.557	nucleus
CnMYB205	*AZ15G0265110.1*	Chr15(-):8591989-8593195	326	36.00	5.58	52.31	-0.493	nucleus
CnMYB206	*AZ15G0266690.1*	Chr15(-):13565385-13620908	976	107.46	6.14	44.49	-0.181	nucleus
CnMYB207	*AZ15G0270340.1*	Chr15(-):48651131-48652764	317	35.03	5.56	51.85	-0.690	nucleus
CnMYB208	*AZ16G0272220.1*	Chr16(+):53406624-53408629	295	32.98	6.83	48.76	-0.689	nucleus
CnMYB209	*AZ16G0276570.1*	Chr16(-):72075986-72092267	450	49.83	5.35	50.46	-0.700	nucleus
CnMYB210	*AZ16G0277170.1*	Chr16(-):73657590-73658796	293	32.61	5.80	61.66	-0.565	nucleus
CnMYB211	*AZ16G0277470.2*	Chr16(+):74320657-74323262	363	40.59	8.58	59.76	-0.831	nucleus
CnMYB212	*AZ16G0278830.1*	Chr16(+):77125101-77126831	278	29.66	8.79	65.21	-0.508	nucleus
CnMYB213	*AZ16G0279130.3*	Chr16(+):77697772-77710261	1679	182.27	6.04	51.60	-0.629	nucleus
CnMYB214	*AZ16G0279940.1*	Chr16(-):79532041-79553655	603	67.93	6.19	57.01	-0.265	nucleus

Table S2. 1R–MYB members of rename in *Arabidopsis thaliana*.

Gene ID	Rename
AT1G01060.1	*AtMYB-1*
AT1G01380.1	*AtMYB-2*
AT1G01520.1	*AtMYB-3*
AT1G06910.1	*AtMYB-4*
AT1G07540.1	*AtMYB-5*
AT1G08810.2	*AtMYB-6*
AT1G09710.2	*AtMYB-7*
AT1G15720.1	*AtMYB-8*
AT1G17460.2	*AtMYB-9*
AT1G17520.1	*AtMYB-10*
AT1G18330.2	*AtMYB-11*
AT1G18960.1	*AtMYB-12*
AT1G19000.1	*AtMYB-13*
AT1G19510.1	*AtMYB-14*
AT1G49950.1	*AtMYB-15*
AT1G58220.1	*AtMYB-16*
AT1G70000.1	*AtMYB-17*
AT1G71030.1	*AtMYB-18*
AT1G72650.2	*AtMYB-19*
AT1G72740.1	*AtMYB-20*
AT1G74840.1	*AtMYB-21*
AT1G75250.1	*AtMYB-22*
AT2G13960.1	*AtMYB-23*
AT2G18328.1	*AtMYB-24*
AT2G30420.1	*AtMYB-25*
AT2G30424.1	*AtMYB-26*
AT2G30432.1	*AtMYB-27*
AT2G36960.3	*AtMYB-28*
AT2G42150.1	*AtMYB-29*
AT2G44430.1	*AtMYB-30*
AT2G46410.1	*AtMYB-31*
AT2G46830.1	*AtMYB-32*
AT2G47210.1	*AtMYB-33*
AT3G09600.1	*AtMYB-34*
AT3G10113.1	*AtMYB-35*
AT3G10580.1	*AtMYB-36*
AT3G10590.1	*AtMYB-37*
AT3G16350.1	*AtMYB-38*
AT3G21430.2	*AtMYB-39*
AT3G23250.2	*AtMYB-40*
AT3G46130.4	*AtMYB-41*
AT3G49850.1	*AtMYB-42*

AT3G53790.1	*AtMYB-43*
AT3G57980.1	*AtMYB-44*
AT3G60110.1	*AtMYB-45*
AT4G01060.1	*AtMYB-46*
AT4G01280.2	*AtMYB-47*
AT4G09450.1	*AtMYB-48*
AT4G36570.1	*AtMYB-49*
AT4G39160.2	*AtMYB-50*
AT4G39250.1	*AtMYB-51*
AT5G02840.1	*AtMYB-52*
AT5G17300.1	*AtMYB-53*
AT5G37260.1	*AtMYB-54*
AT5G41020.1	*AtMYB-55*
AT5G45420.1	*AtMYB-56*
AT5G47390.1	*AtMYB-57*
AT5G52660.2	*AtMYB-58*
AT5G53200.1	*AtMYB-59*
AT5G56840.1	*AtMYB-60*
AT5G58340.1	*AtMYB-61*
AT5G58900.1	*AtMYB-62*
AT5G59780.2	*AtMYB-63*
AT5G61420.1	*AtMYB-64*
AT5G61620.1	*AtMYB-65*
AT5G67580.1	*AtMYB-66*

Table S3. R2R3–MYB, 3R–MYB and 4R–MYB members of rename in *Arabidopsis thaliana*.

Gene ID	Rename
At3g27920	*AtMYB0*
At3g09230	*AtMYB1*
At2g47190	*AtMYB2*
At1g22640	*AtMYB3*
At4g38620	*AtMYB4*
At3g13540	*AtMYB5*
At4g09460	*AtMYB6*
At2g16720	*AtMYB7*
At1g35515	*AtMYB8*
At5g16770	*AtMYB9*
At3g12820	*AtMYB10*
At3g62610	*AtMYB11*
At2g47460	*AtMYB12*
At1g06180	*AtMYB13*
At2g31180	*AtMYB14*
At3g23250	*AtMYB15*
At5g15310	*AtMYB16*
At3g61250	*AtMYB17*
At4g25560	*AtMYB18*
At5g52260	*AtMYB19*
At1g66230	*AtMYB20*
At3g27810	*AtMYB21*
At5g40430	*AtMYB22*
At5g40330	*AtMYB23*
At5g40350	*AtMYB24*
At2g39880	*AtMYB25*
At3g13890	*AtMYB26*
At3g53200	*AtMYB27*
At5g61420	*AtMYB28*
At5g07690	*AtMYB29*
At3g28910	*AtMYB30*
At1g74650	*AtMYB31*
At4g34990	*AtMYB32*
At5g06100	*AtMYB33*
At5g60890	*AtMYB34*
At3g28470	*AtMYB35*
At5g57620	*AtMYB36*
At5g23000	*AtMYB37*
At2g36890	*AtMYB38*
At4g17785	*AtMYB39*
At5g14340	*AtMYB40*
At4g28110	*AtMYB41*

At4g12350	*AtMYB42*
At5g16600	*AtMYB43*
At5g67300	*AtMYB44*
At3g48920	*AtMYB45*
At5g12870	*AtMYB46*
At1g18710	*AtMYB47*
At3g46130	*AtMYB48*
At5g54230	*AtMYB49*
At1g57560	*AtMYB50*
At1g18570	*AtMYB51*
At1g17950	*AtMYB52*
At5g65230	*AtMYB53*
At1g73410	*AtMYB54*
At4g01680	*AtMYB55*
At5g17800	*AtMYB56*
At3g01530	*AtMYB57*
At1g16490	*AtMYB58*
At5g59780	*AtMYB59*
At1g08810	*AtMYB60*
At1g09540	*AtMYB61*
At1g68320	*AtMYB62*
At1g79180	*AtMYB63*
At5g11050	*AtMYB64*
At3g11440	*AtMYB65*
At5g14750	*AtMYB66*
At3g12720	*AtMYB67*
At5g65790	*AtMYB68*
At4g33450	*AtMYB69*
At2g23290	*AtMYB70*
At3g24310	*AtMYB71*
At1g56160	*AtMYB72*
At4g37260	*AtMYB73*
At4g05100	*AtMYB74*
At1g56650	*AtMYB75*
At5g07700	*AtMYB76*
At3g50060	*AtMYB77*
At5g49620	*AtMYB78*
At4g13480	*AtMYB79*
At5g56110	*AtMYB80*
At2g26960	*AtMYB81*
At5g52600	*AtMYB82*
At3g08500	*AtMYB83*
At3g49690	*AtMYB84*
At4g22680	*AtMYB85*

At5g26660	*AtMYB86*
At4g37780	*AtMYB87*
At2g02820	*AtMYB88*
At5g39700	*AtMYB89*
At1g66390	*AtMYB90*
At2g37630	*AtMYB91*
At5g10280	*AtMYB92*
At1g34670	*AtMYB93*
At3g47600	*AtMYB94*
At1g74430	*AtMYB95*
At5g62470	*AtMYB96*
At4g26930	*AtMYB97*
At4g18770	*AtMYB98*
At5g62320	*AtMYB99*
At2g25230	*AtMYB100*
At2g32460	*AtMYB101*
At4g21440	*AtMYB102*
At1g63910	*AtMYB103*
At2g26950	*AtMYB104*
At1g69560	*AtMYB105*
At3g01140	*AtMYB106*
At3g02940	*AtMYB107*
At3g06490	*AtMYB108*
At3g55730	*AtMYB109*
At3g29020	*AtMYB110*
At5g49330	*AtMYB111*
At1g48000	*AtMYB112*
At1g66370	*AtMYB113*
At1g66380	*AtMYB114*
At5g40360	*AtMYB115*
At1g25340	*AtMYB116*
At1g26780	*AtMYB117*
At3g27780	*AtMYB118*
At5g58850	*AtMYB119*
At5g55020	*AtMYB120*
At3g30210	*AtMYB121*
At1g74080	*AtMYB122*
At5g35550	*AtMYB123*
At1g14350	*AtMYB124*
At3g60460	*AtMYB125*
At4g32730	*AtMYB3R1*
At4g00540	*AtMYB3R2*
At3g09370	*AtMYB3R3*
At5g11510	*AtMYB3R-4*

At5g02320	*AtMYB3R-5*
At3g18100	*AtMYB4R1*

Table S4. Gene replication events, selection pressure and divergence time.

Gene Name	Gene Name	Ka	Ks	Ka/Ks	Types of selection	Mya (million years ago)
CnMYB14	*CnMYB36*	0.18	1.61	0.11	Purifying	131.92
CnMYB6	*CnMYB58*	0.08	0.26	0.32	Purifying	20.95
CnMYB7	*CnMYB59*	0.08	0.27	0.29	Purifying	22.00
CnMYB8	*CnMYB60*	0.12	0.38	0.32	Purifying	30.78
CnMYB9	*CnMYB61*	0.25	0.64	0.39	Purifying	52.79
CnMYB12	*CnMYB62*	0.10	0.48	0.21	Purifying	39.52
CnMYB13	*CnMYB63*	0.13	0.24	0.54	Purifying	19.42
CnMYB14	*CnMYB64*	0.10	0.73	0.14	Purifying	59.51
CnMYB15	*CnMYB65*	0.08	0.33	0.24	Purifying	26.80
CnMYB16	*CnMYB66*	0.14	0.28	0.48	Purifying	23.28
CnMYB17	*CnMYB67*	0.12	0.40	0.31	Purifying	32.84
CnMYB4	*CnMYB81*	0.28	1.54	0.18	Purifying	126.07
CnMYB5	*CnMYB80*	0.18	1.12	0.16	Purifying	91.56
CnMYB9	*CnMYB128*	0.39	1.46	0.27	Purifying	119.55
CnMYB5	*CnMYB166*	0.08	0.30	0.26	Purifying	24.68
CnMYB3	*CnMYB164*	0.09	0.24	0.36	Purifying	19.96
CnMYB6	*CnMYB203*	0.24	0.82	0.30	Purifying	67.12
CnMYB7	*CnMYB204*	0.24	1.12	0.21	Purifying	92.06
CnMYB9	*CnMYB205*	0.42	1.45	0.29	Purifying	118.67
CnMYB25	*CnMYB141*	0.22	0.49	0.45	Purifying	40.10
CnMYB28	*CnMYB142*	0.14	0.41	0.35	Purifying	33.41
CnMYB30	*CnMYB144*	0.13	0.46	0.29	Purifying	37.72
CnMYB32	*CnMYB146*	0.10	0.30	0.32	Purifying	24.98
CnMYB33	*CnMYB147*	0.07	0.38	0.18	Purifying	31.06
CnMYB18	*CnMYB138*	0.16	0.48	0.34	Purifying	39.59
CnMYB20	*CnMYB159*	0.38	1.00	0.38	Purifying	81.98
CnMYB20	*CnMYB179*	0.39	0.99	0.39	Purifying	81.20
CnMYB39	*CnMYB68*	0.21	0.85	0.25	Purifying	69.95
CnMYB36	*CnMYB64*	0.16	1.40	0.11	Purifying	114.65
CnMYB36	*CnMYB112*	0.16	0.47	0.34	Purifying	38.31
CnMYB39	*CnMYB116*	0.08	0.26	0.32	Purifying	21.26
CnMYB40	*CnMYB117*	0.11	0.51	0.21	Purifying	41.78
CnMYB43	*CnMYB118*	0.09	0.40	0.22	Purifying	32.79
CnMYB49	*CnMYB120*	0.07	0.23	0.31	Purifying	19.15
CnMYB50	*CnMYB121*	0.15	0.46	0.34	Purifying	37.60
CnMYB51	*CnMYB124*	0.22	0.66	0.33	Purifying	54.26
CnMYB52	*CnMYB126*	0.31	1.30	0.24	Purifying	106.45
CnMYB67	*CnMYB115*	0.27	1.59	0.17	Purifying	130.54
CnMYB68	*CnMYB116*	0.23	0.84	0.27	Purifying	68.90
CnMYB54	*CnMYB134*	0.27	1.60	0.17	Purifying	130.91

CnMYB55	*CnMYB133*	0.38	1.75	0.22	Purifying	143.60
CnMYB58	*CnMYB130*	0.20	0.70	0.28	Purifying	57.26
CnMYB59	*CnMYB129*	0.21	1.00	0.21	Purifying	82.36
CnMYB53	*CnMYB167*	0.08	0.37	0.21	Purifying	30.40
CnMYB56	*CnMYB169*	0.16	0.40	0.39	Purifying	32.86
CnMYB57	*CnMYB200*	0.42	1.89	0.22	Purifying	155.11
CnMYB58	*CnMYB203*	0.21	0.76	0.28	Purifying	62.51
CnMYB59	*CnMYB204*	0.23	1.25	0.18	Purifying	102.70
CnMYB61	*CnMYB205*	0.24	1.25	0.19	Purifying	102.31
CnMYB76	*CnMYB108*	0.09	0.36	0.24	Purifying	29.67
CnMYB78	*CnMYB109*	0.07	0.36	0.20	Purifying	29.54
CnMYB77	*CnMYB108*	0.27	1.06	0.25	Purifying	86.98
CnMYB80	*CnMYB166*	0.17	1.26	0.14	Purifying	103.22
CnMYB81	*CnMYB165*	0.29	1.82	0.16	Purifying	149.30
CnMYB72	*CnMYB210*	0.17	0.87	0.20	Purifying	71.20
CnMYB75	*CnMYB209*	0.35	1.07	0.33	Purifying	87.92
CnMYB88	*CnMYB107*	0.21	1.31	0.16	Purifying	107.05
CnMYB89	*CnMYB104*	0.24	0.82	0.29	Purifying	66.94
CnMYB90	*CnMYB102*	0.28	1.04	0.27	Purifying	85.45
CnMYB102	*CnMYB190*	0.28	0.98	0.29	Purifying	80.45
CnMYB107	*CnMYB191*	0.21	1.12	0.19	Purifying	91.78
CnMYB84	*CnMYB196*	0.16	0.46	0.36	Purifying	37.63
CnMYB86	*CnMYB194*	0.10	0.38	0.26	Purifying	30.98
CnMYB87	*CnMYB192*	0.09	0.23	0.41	Purifying	18.74
CnMYB88	*CnMYB191*	0.09	0.39	0.23	Purifying	31.64
CnMYB90	*CnMYB190*	0.09	0.34	0.28	Purifying	27.50
CnMYB91	*CnMYB189*	0.14	0.39	0.36	Purifying	32.19
CnMYB92	*CnMYB188*	0.11	0.33	0.35	Purifying	26.83
CnMYB102	*CnMYB211*	0.08	0.27	0.31	Purifying	22.27
CnMYB104	*CnMYB212*	0.07	0.33	0.22	Purifying	27.21
CnMYB105	*CnMYB213*	0.12	0.31	0.39	Purifying	25.41
CnMYB89	*CnMYB212*	0.23	0.81	0.28	Purifying	65.99
CnMYB90	*CnMYB211*	0.31	0.97	0.32	Purifying	79.11
CnMYB126	*CnMYB206*	0.12	0.41	0.29	Purifying	33.78
CnMYB128	*CnMYB205*	0.08	0.48	0.17	Purifying	39.01
CnMYB129	*CnMYB204*	0.11	0.42	0.26	Purifying	34.33
CnMYB130	*CnMYB203*	0.09	0.32	0.27	Purifying	26.01
CnMYB131	*CnMYB202*	0.07	0.33	0.20	Purifying	27.23
CnMYB133	*CnMYB200*	0.22	0.43	0.51	Purifying	35.38
CnMYB134	*CnMYB199*	0.07	0.27	0.25	Purifying	22.44
CnMYB136	*CnMYB198*	0.24	0.46	0.51	Purifying	37.82
CnMYB137	*CnMYB197*	0.13	0.42	0.31	Purifying	34.19
CnMYB155	*CnMYB178*	0.08	0.40	0.20	Purifying	32.59
CnMYB159	*CnMYB179*	0.14	0.29	0.50	Purifying	23.61

CnMYB160	*CnMYB180*	0.15	0.38	0.40	Purifying	31.33
CnMYB161	*CnMYB181*	0.09	0.35	0.26	Purifying	28.94
CnMYB162	*CnMYB183*	0.13	0.51	0.27	Purifying	41.59
CnMYB190	*CnMYB211*	0.29	0.94	0.31	Purifying	76.72

Table S5. Synteny analysis of MYB gene family in *Arabidopsis thaliana* and *Cocos nucifera*.

At Gene Name	Cn Gene ID
AT5G14340.1	*AZ01G0007770.1*
AT1G26780.2	*AZ01G0016690.1*
AT1G69560.2	*AZ01G0016690.1*
AT3G29020.2	*AZ01G0016690.1*
AT3G47600.1	*AZ01G0016790.1*
AT1G14350.2	*AZ01G0016910.1*
AT2G02820.2	*AZ01G0016910.1*
AT5G10280.1	*AZ01G0017520.1*
AT5G65230.1	*AZ01G0017520.1*
AT1G25340.3	*AZ01G0019330.1*
AT1G68320.1	*AZ01G0019330.1*
AT1G01380.1	*AZ01G0022480.1*
AT2G46410.1	*AZ01G0022480.1*
AT4G01060.1	*AZ01G0022480.1*
AT5G53200.1	*AZ01G0022480.1*
AT5G52260.1	*AZ02G0032900.1*
AT3G61250.1	*AZ02G0032920.1*
AT2G46830.1	*AZ02G0037050.1*
AT5G52660.2	*AZ02G0037500.1*
AT5G16770.2	*AZ02G0043140.1*
AT2G16720.1	*AZ02G0044370.1*
AT4G38620.1	*AZ02G0044370.1*
AT3G23250.1	*AZ02G0046620.1*
AT2G46410.1	*AZ03G0051100.1*
AT4G01060.1	*AZ03G0051100.1*
AT5G53200.1	*AZ03G0051100.1*
AT2G18328.1	*AZ03G0056230.1*
AT3G27810.1	*AZ03G0056400.1*
AT3G01530.1	*AZ03G0056400.1*
AT4G05090.1	*AZ03G0059780.1*
AT4G21440.1	*AZ03G0059780.1*
AT5G54230.1	*AZ03G0059780.1*
AT4G26930.1	*AZ03G0062380.1*
AT5G55020.1	*AZ03G0062380.1*
AT3G49690.1	*AZ03G0073080.1*
AT4G37780.1	*AZ03G0073080.1*
AT5G65790.1	*AZ03G0073080.1*
AT1G26780.2	*AZ04G0082970.1*
AT1G69560.2	*AZ04G0082970.1*
AT3G29020.2	*AZ04G0082970.1*
AT3G47600.1	*AZ04G0083170.1*
AT5G15310.1	*AZ04G0083170.1*

AT1G14350.2	*AZ04G0083490.1*
AT2G02820.2	*AZ04G0083490.1*
AT1G34670.1	*AZ04G0084410.1*
AT5G10280.1	*AZ04G0084410.1*
AT5G65230.1	*AZ04G0084410.1*
AT1G01380.1	*AZ04G0091560.1*
AT2G46410.1	*AZ04G0091560.1*
AT4G01060.1	*AZ04G0091560.1*
AT5G53200.1	*AZ04G0091560.1*
AT1G09540.1	*AZ05G0097330.1*
AT4G01680.2	*AZ05G0097330.1*
AT5G26660.1	*AZ05G0097330.1*
AT2G47190.1	*AZ05G0098140.1*
AT3G06490.1	*AZ05G0098140.1*
AT5G49620.2	*AZ05G0098140.1*
AT2G47460.1	*AZ05G0098830.1*
AT5G49330.1	*AZ05G0098830.1*
AT5G57620.1	*AZ05G0101920.1*
AT5G57620.1	*AZ05G0107400.1*
AT5G67300.1	*AZ05G0109860.1*
AT4G01680.2	*AZ05G0122770.1*
AT5G58850.1	*AZ06G0123300.1*
AT2G38080.1	*AZ06G0123480.1*
AT5G58910.2	*AZ06G0123480.1*
AT5G01190.1	*AZ06G0123480.1*
AT2G39880.1	*AZ06G0123810.1*
AT3G09230.1	*AZ06G0123810.1*
AT3G55730.1	*AZ06G0123810.1*
AT3G09370.3	*AZ06G0124680.1*
AT5G02320.2	*AZ06G0124680.1*
AT5G59780.3	*AZ06G0125260.1*
AT2G47460.1	*AZ06G0137370.1*
AT3G62610.1	*AZ06G0137370.1*
AT1G69560.2	*AZ06G0138850.2*
AT2G40300.1	*AZ06G0140810.1*
AT3G11050.1	*AZ06G0140810.1*
AT5G01600.1	*AZ06G0140810.1*
AT5G02840.1	*AZ06G0141920.1*
AT3G46130.1	*AZ06G0142560.1*
AT5G59780.3	*AZ06G0142560.1*
AT5G57620.1	*AZ07G0145430.1*
AT3G50060.1	*AZ07G0150740.1*
AT5G67300.1	*AZ07G0150740.1*
AT2G46410.1	*AZ08G0163640.1*

AT4G01060.1	*AZ08G0163640.1*
AT5G53200.1	*AZ08G0163640.1*
AT1G75250.1	*AZ08G0167160.1*
AT3G27810.1	*AZ08G0167270.1*
AT5G55020.1	*AZ08G0171390.1*
AT5G17800.1	*AZ08G0172480.1*
AT1G79180.2	*AZ08G0174510.1*
AT3G49690.1	*AZ09G0179960.1*
AT4G37780.1	*AZ09G0179960.1*
AT5G65790.1	*AZ09G0179960.1*
AT5G65230.1	*AZ09G0187760.1*
AT5G10280.1	*AZ09G0187760.1*
AT1G74650.1	*AZ09G0189840.1*
AT3G47600.1	*AZ09G0189840.1*
AT1G26780.2	*AZ09G0190250.1*
AT1G19510.1	*AZ09G0193600.1*
AT1G75250.1	*AZ09G0193600.1*
AT2G18328.1	*AZ09G0193600.1*
AT4G36570.1	*AZ09G0193600.1*
AT4G39250.1	*AZ09G0193600.1*
AT5G47390.1	*AZ10G0203990.1*
AT3G02940.1	*AZ10G0205290.1*
AT4G17785.1	*AZ10G0205290.1*
AT5G16770.2	*AZ10G0205290.1*
AT2G16720.1	*AZ10G0206030.1*
AT4G38620.1	*AZ10G0206030.1*
AT4G34990.1	*AZ10G0206030.1*
AT2G16720.1	*AZ11G0214040.1*
AT4G34990.1	*AZ11G0214040.1*
AT4G38620.1	*AZ11G0214040.1*
AT5G23350.1	*AZ11G0217040.1*
AT5G52260.1	*AZ11G0217970.1*
AT3G23250.1	*AZ11G0220750.1*
AT5G14340.1	*AZ12G0227370.1*
AT5G16770.2	*AZ13G0242460.1*
AT4G34990.1	*AZ13G0246320.1*
AT4G38620.1	*AZ13G0246320.1*
AT4G00540.1	*AZ13G0248380.1*
AT3G23250.1	*AZ13G0250330.1*
AT3G46130.1	*AZ14G0259720.1*
AT5G59780.3	*AZ14G0259720.1*
AT3G09370.3	*AZ14G0260160.1*
AT5G02320.2	*AZ14G0260160.1*
AT2G28940.2	*AZ14G0260170.1*

AT2G37630.1	*AZ14G0260170.1*
AT2G39880.1	*AZ14G0260820.1*
AT3G55730.1	*AZ14G0260820.1*
AT2G38090.1	*AZ14G0261090.1*
AT5G01200.1	*AZ14G0261090.1*
AT5G58900.1	*AZ14G0261090.1*
AT5G58850.1	*AZ14G0261200.1*
AT5G15310.1	*AZ15G0263830.1*
AT1G69560.2	*AZ15G0263930.1*
AT1G26780.2	*AZ15G0263930.1*
AT3G29020.2	*AZ15G0263930.1*
AT1G74650.1	*AZ15G0264170.1*
AT3G47600.1	*AZ15G0264170.1*
AT5G65230.1	*AZ15G0265110.1*
AT5G10280.1	*AZ15G0265110.1*
AT3G49680.1	*AZ15G0266690.1*
AT4G37780.1	*AZ15G0266690.1*
AT5G65790.1	*AZ15G0266690.1*
AT2G47460.1	*AZ16G0276570.1*
AT3G62610.1	*AZ16G0276570.1*
AT5G49330.1	*AZ16G0276570.1*
AT2G47190.1	*AZ16G0277170.1*
AT3G06490.1	*AZ16G0277170.1*
AT2G28690.1	*AZ16G0279940.1*
AT3G46130.1	*AZ16G0279940.1*
AT5G59760.1	*AZ16G0279940.1*

Table S6. Synteny analysis of MYB gene family in *Oryza sativa* and *Cocos nucifera*.

Os Gene ID	Cn Gene ID
Os01t0863300-01	*AZ01G0003870.1*
Os05t0442400-01	*AZ01G0003870.1*
Os01t0874300-01	*AZ01G0010240.1*
Os11t0180900-00	*AZ01G0010240.1*
Os07t0629000-01	*AZ01G0016790.1*
Os07t0627300-01	*AZ01G0016910.1*
Os03t0388600-01	*AZ01G0017520.1*
Os06t0221000-00	*AZ01G0017520.1*
Os01t0128000-01	*AZ01G0019330.1*
Os02t0732600-01	*AZ01G0020080.1*
Os06t0258000-01	*AZ01G0020080.1*
Os01t0229000-00	*AZ01G0021730.1*
Os01t0619100-01	*AZ01G0022480.1*
Os01t0187900-01	*AZ01G0024000.1*
Os05t0195700-01	*AZ01G0024000.1*
Os01t0191900-01	*AZ01G0024190.1*
Os01t0722300-01	*AZ01G0024190.1*
Os04t0532800-01	*AZ02G0032900.1*
Os02t0641900-01	*AZ02G0032920.1*
Os02t0618400-02	*AZ02G0036210.1*
Os03t0142600-00	*AZ02G0036210.1*
Os04t0508500-01	*AZ02G0036210.1*
Os02t0680700-01	*AZ02G0037500.1*
Os08t0549000-01	*AZ02G0038740.1*
Os04t0594100-01	*AZ02G0043140.1*
Os10t0478300-01	*AZ02G0046620.1*
Os02t0624300-01	*AZ02G0046620.1*
Os04t0517100-02	*AZ02G0046620.1*
Os01t0709000-01	*AZ03G0049700.1*
Os01t0192300-01	*AZ03G0053570.1*
Os01t0603300-01	*AZ03G0053570.1*
Os05t0195700-01	*AZ03G0053570.1*
Os05t0589400-02	*AZ03G0053570.1*
Os01t0635000-00	*AZ03G0056230.1*
Os01t0663051-00	*AZ03G0056230.1*
Os05t0579600-01	*AZ03G0056230.1*
Os05t0567600-00	*AZ03G0056240.1*
Os01t0637800-00	*AZ03G0056400.1*
Os01t0702700-01	*AZ03G0058150.1*
Os05t0543600-01	*AZ03G0058150.1*
Os07t0558100-00	*AZ03G0059780.1*
Os03t0771100-01	*AZ03G0073080.1*

Os02t0732600-01	*AZ04G0079220.1*
Os06t0258000-01	*AZ04G0079220.1*
Os09t0401000-01	*AZ04G0079220.1*
Os08t0435700-00	*AZ04G0082970.1*
Os07t0629000-01	*AZ04G0083170.1*
Os08t0437300-00	*AZ04G0083170.1*
Os03t0388600-01	*AZ04G0084410.1*
Os06t0221000-00	*AZ04G0084410.1*
Os08t0486300-00	*AZ04G0084410.1*
Os02t0732600-01	*AZ04G0088200.1*
Os06t0258000-01	*AZ04G0088200.1*
Os09t0401000-01	*AZ04G0088200.1*
Os01t0229000-00	*AZ04G0090630.1*
Os02t0271900-01	*AZ04G0090630.1*
Os06t0162700-01	*AZ04G0090630.1*
Os01t0619100-01	*AZ04G0091560.1*
Os01t0187900-01	*AZ04G0093340.1*
Os05t0195700-01	*AZ04G0093340.1*
Os01t0191900-01	*AZ04G0093700.1*
Os01t0722300-01	*AZ04G0093700.1*
Os01t0192300-01	*AZ04G0093750.1*
Os05t0195700-01	*AZ04G0093750.1*
Os01t0285300-01	*AZ05G0097330.1*
Os05t0140100-01	*AZ05G0097330.1*
Os01t0298400-01	*AZ05G0098140.1*
Os11t0684000-01	*AZ05G0098140.1*
Os03t0315400-01	*AZ05G0098140.1*
Os01t0305900-01	*AZ05G0098830.1*
Os02t0786400-00	*AZ05G0101920.1*
Os08t0248700-01	*AZ05G0101920.1*
Os09t0431300-00	*AZ05G0101920.1*
Os02t0786400-00	*AZ05G0107400.1*
Os08t0248700-01	*AZ05G0107400.1*
Os02t0187700-00	*AZ05G0109860.1*
Os06t0637500-02	*AZ05G0109860.1*
Os09t0106700-01	*AZ05G0109860.1*
Os01t0874300-01	*AZ05G0119730.1*
Os11t0180900-00	*AZ05G0119730.1*
Os05t0429900-01	*AZ05G0119730.1*
Os01t0285300-01	*AZ05G0122770.1*
Os03t0371800-00	*AZ05G0122770.1*
Os05t0140100-01	*AZ05G0122770.1*
Os01t0855400-00	*AZ06G0123300.1*
Os01t0850400-01	*AZ06G0123810.1*

Os01t0841500-01	*AZ06G0124680.1*
Os05t0459000-01	*AZ06G0124680.1*
Os11t0700500-01	*AZ06G0125260.1*
Os01t0274800-01	*AZ06G0128770.1*
Os11t0128500-01	*AZ06G0128900.1*
Os12t0125000-01	*AZ06G0128900.1*
Os01t0812000-01	*AZ06G0129250.2*
Os05t0490600-00	*AZ06G0129250.2*
Os01t0305900-01	*AZ06G0137370.1*
Os01t0298400-01	*AZ06G0138250.1*
Os03t0315400-01	*AZ06G0138250.1*
Os11t0106700-02	*AZ06G0140810.1*
Os12t0106000-01	*AZ06G0140810.1*
Os05t0126300-01	*AZ06G0141580.2*
Os01t0156000-01	*AZ06G0141920.1*
Os02t0786400-00	*AZ07G0145430.1*
Os09t0431300-00	*AZ07G0145430.1*
Os02t0187700-00	*AZ07G0150740.1*
Os06t0637500-02	*AZ07G0150740.1*
Os09t0106700-01	*AZ07G0150740.1*
Os04t0470600-01	*AZ07G0152280.1*
Os01t0619100-01	*AZ08G0163640.1*
Os01t0191900-01	*AZ08G0165150.1*
Os01t0722300-01	*AZ08G0165150.1*
Os05t0553400-01	*AZ08G0165150.1*
Os01t0192300-01	*AZ08G0165210.1*
Os01t0603300-01	*AZ08G0165210.1*
Os01t0635000-00	*AZ08G0167160.1*
Os01t0663051-00	*AZ08G0167160.1*
Os05t0579600-01	*AZ08G0167160.1*
Os01t0637800-00	*AZ08G0167270.1*
Os01t0702700-01	*AZ08G0168470.1*
Os05t0543600-01	*AZ08G0168470.1*
Os03t0771100-01	*AZ09G0179960.1*
Os03t0388600-01	*AZ09G0187760.1*
Os06t0221000-00	*AZ09G0187760.1*
Os08t0486300-00	*AZ09G0187760.1*
Os04t0461000-01	*AZ09G0189840.1*
Os07t0629000-01	*AZ09G0189840.1*
Os08t0437300-00	*AZ09G0189840.1*
Os08t0435700-00	*AZ09G0190250.1*
Os03t0176300-00	*AZ09G0191600.1*
Os01t0635000-00	*AZ09G0193600.1*
Os02t0706400-01	*AZ09G0193600.1*

Os05t0579600-01	*AZ09G0193600.1*
Os02t0618400-02	*AZ10G0200550.1*
Os03t0142600-00	*AZ10G0200550.1*
Os04t0508500-01	*AZ10G0200550.1*
Os10t0561400-02	*AZ10G0203990.1*
Os04t0593200-01	*AZ10G0205290.1*
Os09t0538400-01	*AZ10G0206030.1*
Os09t0532900-04	*AZ11G0208510.1*
Os08t0144000-01	*AZ11G0211820.1*
Os08t0151300-02	*AZ11G0211900.1*
Os02t0680700-01	*AZ11G0212760.2*
Os06t0105800-02	*AZ11G0212760.2*
Os08t0549000-01	*AZ11G0214040.1*
Os09t0538400-01	*AZ11G0214040.1*
Os06t0728700-01	*AZ11G0215700.1*
Os02t0636600-00	*AZ11G0217040.1*
Os04t0676700-01	*AZ11G0217040.1*
Os04t0526800-01	*AZ11G0217040.1*
Os04t0532800-01	*AZ11G0217970.1*
Os10t0478300-01	*AZ11G0220750.1*
Os02t0624300-01	*AZ11G0220750.1*
Os04t0517100-02	*AZ11G0220750.1*
Os11t0207600-00	*AZ12G0231220.1*
Os01t0874300-01	*AZ12G0231930.1*
Os11t0180900-00	*AZ12G0231930.1*
Os05t0429900-01	*AZ12G0231930.1*
Os02t0732600-01	*AZ13G0238110.1*
Os06t0258000-01	*AZ13G0238110.1*
Os09t0401000-01	*AZ13G0238110.1*
Os06t0112700-00	*AZ13G0242460.1*
Os08t0151000-00	*AZ13G0244680.1*
Os02t0109200-01	*AZ13G0244820.1*
Os06t0109600-01	*AZ13G0245720.1*
Os08t0159000-01	*AZ13G0245720.1*
Os08t0549000-01	*AZ13G0246320.1*
Os09t0538400-01	*AZ13G0246320.1*
Os04t0532800-01	*AZ13G0248440.1*
Os10t0478300-01	*AZ13G0250330.1*
Os02t0624300-01	*AZ13G0250330.1*
Os04t0517100-02	*AZ13G0250330.1*
Os01t0812000-01	*AZ14G0257350.1*
Os05t0490600-00	*AZ14G0257350.1*
Os11t0128500-01	*AZ14G0257640.1*
Os12t0125000-01	*AZ14G0257640.1*

Os01t0274800-01	*AZ14G0257750.1*
Os11t0700500-01	*AZ14G0259720.1*
Os01t0841500-01	*AZ14G0260160.1*
Os05t0459000-01	*AZ14G0260160.1*
Os01t0850400-01	*AZ14G0260820.1*
Os01t0853700-01	*AZ14G0261090.1*
Os05t0449900-01	*AZ14G0261090.1*
Os01t0855400-00	*AZ14G0261200.1*
Os04t0461000-01	*AZ15G0263830.1*
Os07t0629000-01	*AZ15G0264170.1*
Os08t0437300-00	*AZ15G0264170.1*
Os03t0388600-01	*AZ15G0265110.1*
Os06t0221000-00	*AZ15G0265110.1*
Os08t0486300-00	*AZ15G0265110.1*
Os03t0231600-01	*AZ15G0266690.1*
Os01t0305900-01	*AZ16G0276570.1*
Os01t0298400-01	*AZ16G0277170.1*
Os03t0315400-01	*AZ16G0277170.1*
Os07t0688200-01	*AZ16G0277170.1*
Os01t0975300-01	*AZ16G0279940.1*
Os05t0115100-02	*AZ16G0279940.1*

Table S7. Synteny analysis of MYB gene family in *Ananas comosus* and *Cocos nucifera*.

Ac Gene ID	Cn Gene ID
Aco024560.1.v3	*AZ01G0003870.1*
Aco003309.1.v3	*AZ01G0009680.1*
Aco011242.1.v3	*AZ01G0010240.1*
Aco003262.1.v3	*AZ01G0010240.1*
Aco007902.1.v3	*AZ01G0010240.1*
Aco014685.1.v3	*AZ01G0016690.1*
Aco009605.1.v3	*AZ01G0016790.1*
Aco001113.1.v3	*AZ01G0016790.1*
Aco014702.1.v3	*AZ01G0016790.1*
Aco009598.1.v3	*AZ01G0016910.1*
Aco001218.1.v3	*AZ01G0017520.1*
Aco002802.1.v3	*AZ01G0017520.1*
Aco004081.1.v3	*AZ01G0017520.1*
Aco001378.1.v3	*AZ01G0019330.1*
Aco021231.1.v3	*AZ01G0019540.1*
Aco001045.1.v3	*AZ01G0020080.1*
Aco002989.1.v3	*AZ01G0020080.1*
Aco013437.1.v3	*AZ01G0020080.1*
Aco004416.1.v3	*AZ01G0021730.1*
Aco010782.1.v3	*AZ01G0021730.1*
Aco010709.1.v3	*AZ01G0022480.1*
Aco009969.1.v3	*AZ01G0024000.1*
Aco009968.1.v3	*AZ01G0024010.1*
Aco009947.1.v3	*AZ01G0024190.1*
Aco005966.1.v3	*AZ01G0024190.1*
Aco017254.1.v3	*AZ02G0026630.1*
Aco016649.1.v3	*AZ02G0029800.1*
Aco016295.1.v3	*AZ02G0032900.1*
Aco007619.1.v3	*AZ02G0032900.1*
Aco010277.1.v3	*AZ02G0032900.1*
Aco016293.1.v3	*AZ02G0032920.1*
Aco010058.1.v3	*AZ02G0034130.1*
Aco010195.1.v3	*AZ02G0034130.1*
Aco014614.1.v3	*AZ02G0036210.1*
Aco016649.1.v3	*AZ02G0037050.1*
Aco013228.1.v3	*AZ02G0037050.1*
Aco013238.1.v3	*AZ02G0037500.1*
Aco016984.1.v3	*AZ02G0043140.1*
Aco008865.1.v3	*AZ02G0044370.1*
Aco013105.1.v3	*AZ02G0044370.1*
Aco013937.1.v3	*AZ02G0046620.1*
Aco007733.1.v3	*AZ02G0046620.1*

Aco005761.1.v3	*AZ03G0049700.1*
Aco026820.1.v3	*AZ03G0049700.1*
Aco010709.1.v3	*AZ03G0051100.1*
Aco005652.1.v3	*AZ03G0053570.1*
Aco012769.1.v3	*AZ03G0056230.1*
Aco016401.1.v3	*AZ03G0056230.1*
Aco005371.1.v3	*AZ03G0056230.1*
Aco006193.1.v3	*AZ03G0056230.1*
Aco005389.1.v3	*AZ03G0056400.1*
Aco006187.1.v3	*AZ03G0056400.1*
Aco016520.1.v3	*AZ03G0058150.1*
Aco021134.1.v3	*AZ03G0058150.1*
Aco014419.1.v3	*AZ03G0059780.1*
Aco009029.1.v3	*AZ03G0062380.1*
Aco004978.1.v3	*AZ03G0073080.1*
Aco001045.1.v3	*AZ04G0079220.1*
Aco002989.1.v3	*AZ04G0079220.1*
Aco013389.1.v3	*AZ04G0080380.1*
Aco009478.1.v3	*AZ04G0081030.1*
Aco014685.1.v3	*AZ04G0082970.1*
Aco009605.1.v3	*AZ04G0083170.1*
Aco001113.1.v3	*AZ04G0083170.1*
Aco014702.1.v3	*AZ04G0083170.1*
Aco009598.1.v3	*AZ04G0083490.1*
Aco001218.1.v3	*AZ04G0084410.1*
Aco002802.1.v3	*AZ04G0084410.1*
Aco004081.1.v3	*AZ04G0084410.1*
Aco001045.1.v3	*AZ04G0088200.1*
Aco002989.1.v3	*AZ04G0088200.1*
Aco004416.1.v3	*AZ04G0090630.1*
Aco010782.1.v3	*AZ04G0090630.1*
Aco010709.1.v3	*AZ04G0091560.1*
Aco009969.1.v3	*AZ04G0093340.1*
Aco009968.1.v3	*AZ04G0093350.1*
Aco009947.1.v3	*AZ04G0093700.1*
Aco005966.1.v3	*AZ04G0093700.1*
Aco005652.1.v3	*AZ04G0093750.1*
Aco022092.1.v3	*AZ05G0097330.1*
Aco013641.1.v3	*AZ05G0097330.1*
Aco013653.1.v3	*AZ05G0097510.1*
Aco002582.1.v3	*AZ05G0098140.1*
Aco000681.1.v3	*AZ05G0098140.1*
Aco001748.1.v3	*AZ05G0098140.1*
Aco002526.1.v3	*AZ05G0098830.1*

Aco001802.1.v3	*AZ05G0098830.1*
Aco012129.1.v3	*AZ05G0101920.1*
Aco006517.1.v3	*AZ05G0101920.1*
Aco023818.1.v3	*AZ05G0101920.1*
Aco012129.1.v3	*AZ05G0107400.1*
Aco006517.1.v3	*AZ05G0107400.1*
Aco006402.1.v3	*AZ05G0109860.1*
Aco017875.1.v3	*AZ05G0115030.1*
Aco011242.1.v3	*AZ05G0119730.1*
Aco003262.1.v3	*AZ05G0119730.1*
Aco007902.1.v3	*AZ05G0119730.1*
Aco003309.1.v3	*AZ05G0120530.1*
Aco022092.1.v3	*AZ05G0122770.1*
Aco013641.1.v3	*AZ05G0122770.1*
Aco006386.1.v3	*AZ05G0122770.1*
Aco014104.1.v3	*AZ06G0123300.1*
Aco014114.1.v3	*AZ06G0123480.1*
Aco015411.1.v3	*AZ06G0123810.1*
Aco000514.1.v3	*AZ06G0124680.1*
Aco014182.1.v3	*AZ06G0124680.1*
Aco010976.1.v3	*AZ06G0125260.1*
Aco000536.1.v3	*AZ06G0125260.1*
Aco017649.1.v3	*AZ06G0125260.1*
Aco011071.1.v3	*AZ06G0126460.1*
Aco017900.1.v3	*AZ06G0128770.1*
Aco012607.1.v3	*AZ06G0128770.1*
Aco017875.1.v3	*AZ06G0128900.1*
Aco012621.1.v3	*AZ06G0128900.1*
Aco000590.1.v3	*AZ06G0129250.2*
Aco007204.1.v3	*AZ06G0129250.2*
Aco007210.1.v3	*AZ06G0129420.1*
Aco002526.1.v3	*AZ06G0137370.1*
Aco001802.1.v3	*AZ06G0137370.1*
Aco002582.1.v3	*AZ06G0138250.1*
Aco001748.1.v3	*AZ06G0138250.1*
Aco017900.1.v3	*AZ06G0138850.2*
Aco012607.1.v3	*AZ06G0138850.2*
Aco018658.1.v3	*AZ06G0138850.2*
Aco012492.1.v3	*AZ06G0140810.1*
Aco001590.1.v3	*AZ06G0140810.1*
Aco007079.1.v3	*AZ06G0140810.1*
Aco011071.1.v3	*AZ06G0141040.1*
Aco001552.1.v3	*AZ06G0141580.2*
Aco001524.1.v3	*AZ06G0141920.1*

Aco010976.1.v3	*AZ06G0142560.1*
Aco017649.1.v3	*AZ06G0142560.1*
Aco012129.1.v3	*AZ07G0145430.1*
Aco006517.1.v3	*AZ07G0145430.1*
Aco023818.1.v3	*AZ07G0145430.1*
Aco006402.1.v3	*AZ07G0150740.1*
Aco000847.1.v3	*AZ07G0152280.1*
Aco010709.1.v3	*AZ08G0163640.1*
Aco009947.1.v3	*AZ08G0165150.1*
Aco005966.1.v3	*AZ08G0165150.1*
Aco005652.1.v3	*AZ08G0165210.1*
Aco012769.1.v3	*AZ08G0167160.1*
Aco005371.1.v3	*AZ08G0167160.1*
Aco006193.1.v3	*AZ08G0167160.1*
Aco005389.1.v3	*AZ08G0167270.1*
Aco006187.1.v3	*AZ08G0167270.1*
Aco016520.1.v3	*AZ08G0168470.1*
Aco009029.1.v3	*AZ08G0171390.1*
Aco008987.1.v3	*AZ08G0172050.1*
Aco004978.1.v3	*AZ09G0179960.1*
Aco012266.1.v3	*AZ09G0184720.1*
Aco001218.1.v3	*AZ09G0187760.1*
Aco002802.1.v3	*AZ09G0187760.1*
Aco004081.1.v3	*AZ09G0187760.1*
Aco009605.1.v3	*AZ09G0189840.1*
Aco001113.1.v3	*AZ09G0189840.1*
Aco014702.1.v3	*AZ09G0189840.1*
Aco017900.1.v3	*AZ09G0190250.1*
Aco014685.1.v3	*AZ09G0190250.1*
Aco001084.1.v3	*AZ09G0190430.1*
Aco014678.1.v3	*AZ09G0190430.1*
Aco003952.1.v3	*AZ09G0191600.1*
Aco009478.1.v3	*AZ09G0191610.1*
Aco023267.1.v3	*AZ09G0191610.1*
Aco013389.1.v3	*AZ09G0192100.1*
Aco012769.1.v3	*AZ09G0193600.1*
Aco016401.1.v3	*AZ09G0193600.1*
Aco005371.1.v3	*AZ09G0193600.1*
Aco006193.1.v3	*AZ09G0193600.1*
Aco025308.1.v3	*AZ09G0194450.1*
Aco020311.1.v3	*AZ09G0194450.1*
Aco017254.1.v3	*AZ10G0195710.1*
Aco007619.1.v3	*AZ10G0197820.1*
Aco010058.1.v3	*AZ10G0198830.1*

Aco010195.1.v3	*AZ10G0198830.1*
Aco014614.1.v3	*AZ10G0200550.1*
Aco011681.1.v3	*AZ10G0203990.1*
Aco012950.1.v3	*AZ10G0205290.1*
Aco013198.1.v3	*AZ10G0205290.1*
Aco016984.1.v3	*AZ10G0205400.2*
Aco020863.1.v3	*AZ10G0205400.2*
Aco012950.1.v3	*AZ11G0208510.1*
Aco020874.1.v3	*AZ11G0208510.1*
Aco013198.1.v3	*AZ11G0208510.1*
Aco013133.1.v3	*AZ11G0208640.1*
Aco013304.1.v3	*AZ11G0211820.1*
Aco013297.1.v3	*AZ11G0211900.1*
Aco017509.1.v3	*AZ11G0212760.2*
Aco013238.1.v3	*AZ11G0212760.2*
Aco013105.1.v3	*AZ11G0214040.1*
Aco013133.1.v3	*AZ11G0214040.1*
Aco011903.1.v3	*AZ11G0215700.1*
Aco009204.1.v3	*AZ11G0215700.1*
Aco013011.1.v3	*AZ11G0215810.1*
Aco019298.1.v3	*AZ11G0217040.1*
Aco007675.1.v3	*AZ11G0217040.1*
Aco010322.1.v3	*AZ11G0217040.1*
Aco016294.1.v3	*AZ11G0217970.1*
Aco007619.1.v3	*AZ11G0217970.1*
Aco010274.1.v3	*AZ11G0217970.1*
Aco022792.1.v3	*AZ11G0217970.1*
Aco010195.1.v3	*AZ11G0219380.1*
Aco013937.1.v3	*AZ11G0220750.1*
Aco007733.1.v3	*AZ11G0220750.1*
Aco003309.1.v3	*AZ12G0231220.1*
Aco011242.1.v3	*AZ12G0231930.1*
Aco003262.1.v3	*AZ12G0231930.1*
Aco007902.1.v3	*AZ12G0231930.1*
Aco001045.1.v3	*AZ13G0238110.1*
Aco002989.1.v3	*AZ13G0238110.1*
Aco013437.1.v3	*AZ13G0238110.1*
Aco009478.1.v3	*AZ13G0239190.1*
Aco020874.1.v3	*AZ13G0242460.1*
Aco013198.1.v3	*AZ13G0242460.1*
Aco013304.1.v3	*AZ13G0244680.1*
Aco013297.1.v3	*AZ13G0244820.1*
Aco013228.1.v3	*AZ13G0245410.1*
Aco017481.1.v3	*AZ13G0245720.1*

Aco013213.1.v3	*AZ13G0245720.1*
Aco013105.1.v3	*AZ13G0246320.1*
Aco013133.1.v3	*AZ13G0246320.1*
Aco016294.1.v3	*AZ13G0248440.1*
Aco007619.1.v3	*AZ13G0248440.1*
Aco010274.1.v3	*AZ13G0248440.1*
Aco010195.1.v3	*AZ13G0249410.1*
Aco013937.1.v3	*AZ13G0250330.1*
Aco007733.1.v3	*AZ13G0250330.1*
Aco002582.1.v3	*AZ14G0250630.1*
Aco022092.1.v3	*AZ14G0251180.1*
Aco013641.1.v3	*AZ14G0251180.1*
Aco000590.1.v3	*AZ14G0257350.1*
Aco007204.1.v3	*AZ14G0257350.1*
Aco017875.1.v3	*AZ14G0257640.1*
Aco012621.1.v3	*AZ14G0257640.1*
Aco017900.1.v3	*AZ14G0257750.1*
Aco012607.1.v3	*AZ14G0257750.1*
Aco010976.1.v3	*AZ14G0259720.1*
Aco000514.1.v3	*AZ14G0260160.1*
Aco014182.1.v3	*AZ14G0260160.1*
Aco015454.1.v3	*AZ14G0260170.1*
Aco015411.1.v3	*AZ14G0260820.1*
Aco016874.1.v3	*AZ14G0261090.1*
Aco014113.1.v3	*AZ14G0261090.1*
Aco015386.1.v3	*AZ14G0261090.1*
Aco014104.1.v3	*AZ14G0261200.1*
Aco025308.1.v3	*AZ15G0261350.1*
Aco020311.1.v3	*AZ15G0261350.1*
Aco025559.1.v3	*AZ15G0262750.1*
Aco013389.1.v3	*AZ15G0262750.1*
Aco009478.1.v3	*AZ15G0263060.1*
Aco023267.1.v3	*AZ15G0263060.1*
Aco001084.1.v3	*AZ15G0263830.1*
Aco014678.1.v3	*AZ15G0263830.1*
Aco017900.1.v3	*AZ15G0263930.1*
Aco014685.1.v3	*AZ15G0263930.1*
Aco009605.1.v3	*AZ15G0264170.1*
Aco001113.1.v3	*AZ15G0264170.1*
Aco014702.1.v3	*AZ15G0264170.1*
Aco001218.1.v3	*AZ15G0265110.1*
Aco002802.1.v3	*AZ15G0265110.1*
Aco004081.1.v3	*AZ15G0265110.1*
Aco004978.1.v3	*AZ15G0266690.1*

Aco013356.1.v3	*AZ15G0266690.1*
Aco012266.1.v3	*AZ15G0270340.1*
Aco017875.1.v3	*AZ16G0272220.1*
Aco002526.1.v3	*AZ16G0276570.1*
Aco001802.1.v3	*AZ16G0276570.1*
Aco002582.1.v3	*AZ16G0277170.1*
Aco001748.1.v3	*AZ16G0277170.1*
Aco011071.1.v3	*AZ16G0278830.1*
Aco001552.1.v3	*AZ16G0279130.3*
Aco010978.1.v3	*AZ16G0279940.1*
Aco017649.1.v3	*AZ16G0279940.1*

Table S8. Synteny analysis of MYB gene family in *Elaeis guineensis* and *Cocos nucifera*.

Eg Gene ID	Cn Gene ID
LOC105039457	*AZ01G0022480.1*
LOC105037623	*AZ01G0009680.1*
LOC105036764	*AZ01G0010240.1*
LOC105038821	*AZ01G0007770.1*
LOC105038489	*AZ01G0009680.1*
LOC105038411	*AZ01G0010240.1*
LOC105041749	*AZ01G0016690.1*
LOC105041730	*AZ01G0016790.1*
LOC105041706	*AZ01G0016910.1*
LOC105041775	*AZ01G0017520.1*
LOC105041231	*AZ01G0019330.1*
LOC105041192	*AZ01G0019540.1*
LOC105041083	*AZ01G0020080.1*
LOC105040829	*AZ01G0021730.1*
LOC105040719	*AZ01G0022480.1*
LOC105040534	*AZ01G0024000.1*
LOC105040532	*AZ01G0024010.1*
LOC105040495	*AZ01G0024190.1*
LOC105047566	*AZ01G0022480.1*
LOC105047298	*AZ01G0024190.1*
LOC105048294	*AZ01G0021730.1*
LOC105048058	*AZ01G0024000.1*
LOC105048057	*AZ01G0024010.1*
LOC105048018	*AZ01G0024190.1*
LOC105048809	*AZ01G0016690.1*
LOC105048799	*AZ01G0016790.1*
LOC105048579	*AZ01G0016910.1*
LOC105048630	*AZ01G0017520.1*
LOC105048425	*AZ01G0019330.1*
LOC105048449	*AZ01G0019540.1*
LOC105048503	*AZ01G0020080.1*
LOC105051753	*AZ01G0009680.1*
LOC105051841	*AZ01G0010240.1*
LOC105053200	*AZ01G0020080.1*
LOC105056616	*AZ01G0016690.1*
LOC105056584	*AZ01G0016790.1*
LOC105058320	*AZ01G0016790.1*
LOC105061441	*AZ01G0003870.1*
LOC105061460	*AZ01G0003870.1*
LOC105043254	*AZ02G0037050.1*
LOC105043316	*AZ02G0037500.1*
LOC105043438	*AZ02G0038740.1*

LOC105043705	*AZ02G0041060.1*
LOC105044046	*AZ02G0044370.1*
LOC105043192	*AZ02G0036210.1*
LOC105042961	*AZ02G0034130.1*
LOC105042862	*AZ02G0032720.1*
LOC105044321	*AZ02G0046620.1*
LOC105054112	*AZ02G0038740.1*
LOC105054331	*AZ02G0041060.1*
LOC105053827	*AZ02G0043140.1*
LOC105053760	*AZ02G0044370.1*
LOC105054495	*AZ02G0046620.1*
LOC105053692	*AZ02G0036210.1*
LOC105054825	*AZ02G0032900.1*
LOC105055678	*AZ02G0046620.1*
LOC105055559	*AZ02G0034130.1*
LOC105054949	*AZ02G0037050.1*
LOC105054972	*AZ02G0037500.1*
LOC105059234	*AZ02G0034130.1*
LOC105058870	*AZ02G0029800.1*
LOC105059014	*AZ02G0032720.1*
LOC105059220	*AZ02G0032900.1*
LOC105059546	*AZ02G0037050.1*
LOC105059523	*AZ02G0037500.1*
LOC105059635	*AZ02G0044370.1*
LOC109504900	*AZ02G0032900.1*
LOC105032366	*AZ02G0032920.1*
LOC105033428	*AZ02G0046620.1*
LOC105042957	*AZ03G0053570.1*
LOC109506153	*AZ03G0056230.1*
LOC105048234	*AZ03G0056400.1*
LOC105046710	*AZ03G0057640.1*
LOC105046527	*AZ03G0057760.1*
LOC105046112	*AZ03G0058150.1*
LOC105051250	*AZ03G0062230.1*
LOC105051089	*AZ03G0062380.1*
LOC105038840	*AZ03G0049700.1*
LOC105061221	*AZ03G0073080.1*
LOC105039457	*AZ03G0051100.1*
LOC105032141	*AZ03G0059780.1*
LOC105033243	*AZ03G0071770.1*
LOC105040719	*AZ03G0051100.1*
LOC105040495	*AZ03G0053510.1*
LOC105040489	*AZ03G0053570.1*
LOC105047298	*AZ03G0053510.1*

LOC105047305	*AZ03G0053570.1*
LOC105046829	*AZ03G0062380.1*
LOC105046865	*AZ03G0062230.1*
LOC105047566	*AZ03G0051100.1*
LOC105047173	*AZ03G0056230.1*
LOC105047159	*AZ03G0056400.1*
LOC105047026	*AZ03G0058150.1*
LOC105046760	*AZ03G0071770.1*
LOC105048018	*AZ03G0053510.1*
LOC105052704	*AZ03G0059780.1*
LOC105056262	*AZ03G0056230.1*
LOC105055954	*AZ03G0073080.1*
LOC105058289	*AZ03G0073080.1*
LOC105058783	*AZ03G0058150.1*
LOC105042957	*AZ04G0093750.1*
LOC105039457	*AZ04G0091560.1*
LOC105041824	*AZ04G0081030.1*
LOC105041083	*AZ04G0079220.1*
LOC105041749	*AZ04G0082970.1*
LOC105041730	*AZ04G0083170.1*
LOC105041706	*AZ04G0083490.1*
LOC105041775	*AZ04G0084410.1*
LOC105041083	*AZ04G0088200.1*
LOC105040829	*AZ04G0090630.1*
LOC105040719	*AZ04G0091560.1*
LOC105040534	*AZ04G0093340.1*
LOC105040532	*AZ04G0093350.1*
LOC105040495	*AZ04G0093700.1*
LOC105040489	*AZ04G0093750.1*
LOC105041906	*AZ04G0082970.1*
LOC105041906	*AZ04G0080380.1*
LOC105047566	*AZ04G0091560.1*
LOC105047298	*AZ04G0093700.1*
LOC105047305	*AZ04G0093750.1*
LOC105048503	*AZ04G0079220.1*
LOC105048294	*AZ04G0090630.1*
LOC105048058	*AZ04G0093340.1*
LOC105048057	*AZ04G0093350.1*
LOC105048018	*AZ04G0093700.1*
LOC105048809	*AZ04G0082970.1*
LOC105048799	*AZ04G0083170.1*
LOC105048579	*AZ04G0083490.1*
LOC105048630	*AZ04G0084410.1*
LOC105048503	*AZ04G0088200.1*

LOC105053200	*AZ04G0088200.1*
LOC105053200	*AZ04G0079220.1*
LOC105053101	*AZ04G0081030.1*
LOC105053972	*AZ04G0093340.1*
LOC105056616	*AZ04G0082970.1*
LOC105056584	*AZ04G0083170.1*
LOC105056816	*AZ04G0084410.1*
LOC105056443	*AZ04G0081030.1*
LOC105056442	*AZ04G0081040.1*
LOC105058320	*AZ04G0083170.1*
LOC105058421	*AZ04G0084410.1*
LOC105058553	*AZ04G0082970.1*
LOC105058663	*AZ04G0080380.1*
LOC105038411	*AZ05G0119730.1*
LOC105038489	*AZ05G0120530.1*
LOC105040118	*AZ05G0122770.1*
LOC105036764	*AZ05G0119730.1*
LOC105037623	*AZ05G0120530.1*
LOC105034392	*AZ05G0121500.1*
LOC105039251	*AZ05G0107400.1*
LOC105040118	*AZ05G0097330.1*
LOC105040097	*AZ05G0097510.1*
LOC105039994	*AZ05G0098140.1*
LOC105039969	*AZ05G0098330.1*
LOC105039918	*AZ05G0098830.1*
LOC105039613	*AZ05G0101920.1*
LOC105039251	*AZ05G0101920.1*
LOC105039613	*AZ05G0107400.1*
LOC105044973	*AZ05G0098140.1*
LOC105045069	*AZ05G0098830.1*
LOC105046102	*AZ05G0115030.1*
LOC105050171	*AZ05G0109860.1*
LOC105050940	*AZ05G0101920.1*
LOC105050940	*AZ05G0107400.1*
LOC105051841	*AZ05G0119730.1*
LOC105051753	*AZ05G0120530.1*
LOC105052376	*AZ05G0115030.1*
LOC105057307	*AZ05G0098140.1*
LOC105057407	*AZ05G0098830.1*
LOC105059740	*AZ05G0097330.1*
LOC105059778	*AZ05G0098140.1*
LOC105060999	*AZ05G0109860.1*
LOC105061367	*AZ05G0115030.1*
LOC114913049	*AZ05G0096990.1*

LOC105039918	*AZ06G0137370.1*
LOC105046084	*AZ06G0138850.2*
LOC105045883	*AZ06G0140810.1*
LOC105045854	*AZ06G0141040.1*
LOC105045725	*AZ06G0142560.1*
LOC105044477	*AZ06G0125260.1*
LOC105044566	*AZ06G0126460.1*
LOC105044915	*AZ06G0128770.1*
LOC105044973	*AZ06G0138250.1*
LOC105046375	*AZ06G0123300.1*
LOC105046275	*AZ06G0123480.1*
LOC105046315	*AZ06G0123810.1*
LOC105045662	*AZ06G0124680.1*
LOC105045725	*AZ06G0125260.1*
LOC105045854	*AZ06G0126460.1*
LOC105046084	*AZ06G0128770.1*
LOC105046102	*AZ06G0128900.1*
LOC105046145	*AZ06G0129250.2*
LOC105045069	*AZ06G0137370.1*
LOC105044915	*AZ06G0138850.2*
LOC105045077	*AZ06G0140810.1*
LOC105044566	*AZ06G0141040.1*
LOC105044622	*AZ06G0141580.2*
LOC105044667	*AZ06G0141920.1*
LOC105044477	*AZ06G0142560.1*
LOC105045371	*AZ06G0134240.1*
LOC105045509	*AZ06G0129420.1*
LOC105052363	*AZ06G0128770.1*
LOC105052376	*AZ06G0128900.1*
LOC105052148	*AZ06G0123810.1*
LOC105052363	*AZ06G0138850.2*
LOC105052225	*AZ06G0124680.1*
LOC105056616	*AZ06G0138850.2*
LOC105056992	*AZ06G0142560.1*
LOC105057407	*AZ06G0137370.1*
LOC105057270	*AZ06G0128770.1*
LOC105057307	*AZ06G0138250.1*
LOC105057270	*AZ06G0138850.2*
LOC105057083	*AZ06G0141580.2*
LOC105056992	*AZ06G0125260.1*
LOC105032078	*AZ06G0129250.2*
LOC105032584	*AZ06G0123480.1*
LOC105032833	*AZ06G0142560.1*
LOC105032833	*AZ06G0125260.1*

LOC105039251	*AZ07G0145430.1*
LOC105039613	*AZ07G0145430.1*
LOC105050171	*AZ07G0150740.1*
LOC105049977	*AZ07G0152280.1*
LOC105050940	*AZ07G0145430.1*
LOC105060999	*AZ07G0150740.1*
LOC105061441	*AZ07G0156840.1*
LOC105061460	*AZ07G0156840.1*
LOC105042957	*AZ08G0165210.1*
LOC109506153	*AZ08G0167160.1*
LOC105048234	*AZ08G0167270.1*
LOC105046112	*AZ08G0168470.1*
LOC105051250	*AZ08G0171230.1*
LOC105051089	*AZ08G0171390.1*
LOC105039457	*AZ08G0163640.1*
LOC105033243	*AZ08G0174510.1*
LOC105040719	*AZ08G0163640.1*
LOC105040495	*AZ08G0165150.1*
LOC105040489	*AZ08G0165210.1*
LOC105047298	*AZ08G0165150.1*
LOC105047305	*AZ08G0165210.1*
LOC105046829	*AZ08G0171390.1*
LOC105046865	*AZ08G0171230.1*
LOC105047566	*AZ08G0163640.1*
LOC105047173	*AZ08G0167160.1*
LOC105047159	*AZ08G0167270.1*
LOC105047026	*AZ08G0168470.1*
LOC105046760	*AZ08G0174510.1*
LOC105046813	*AZ08G0172050.1*
LOC105048018	*AZ08G0165150.1*
LOC105056262	*AZ08G0167160.1*
LOC105058750	*AZ08G0167160.1*
LOC105061221	*AZ09G0179960.1*
LOC109506153	*AZ09G0193600.1*
LOC105046112	*AZ09G0194060.1*
LOC105041730	*AZ09G0189840.1*
LOC105041749	*AZ09G0190250.1*
LOC105041775	*AZ09G0187760.1*
LOC105041906	*AZ09G0192100.1*
LOC105041823	*AZ09G0191610.1*
LOC105046084	*AZ09G0190250.1*
LOC105044915	*AZ09G0190250.1*
LOC105047173	*AZ09G0193600.1*
LOC105048630	*AZ09G0187760.1*

LOC105048799	*AZ09G0189840.1*
LOC105048809	*AZ09G0190250.1*
LOC105053101	*AZ09G0191610.1*
LOC105056584	*AZ09G0189840.1*
LOC105056616	*AZ09G0190250.1*
LOC105056637	*AZ09G0190430.1*
LOC105055876	*AZ09G0179830.1*
LOC105055954	*AZ09G0179960.1*
LOC105056816	*AZ09G0187760.1*
LOC105056440	*AZ09G0191600.1*
LOC105056442	*AZ09G0191610.1*
LOC105056262	*AZ09G0193600.1*
LOC105056223	*AZ09G0194060.1*
LOC105058553	*AZ09G0190250.1*
LOC105058564	*AZ09G0190430.1*
LOC105058635	*AZ09G0191600.1*
LOC105058663	*AZ09G0192100.1*
LOC105058750	*AZ09G0193600.1*
LOC105058783	*AZ09G0194060.1*
LOC105058421	*AZ09G0187760.1*
LOC105058320	*AZ09G0189840.1*
LOC105058289	*AZ09G0179960.1*
LOC105060070	*AZ09G0179960.1*
LOC105033110	*AZ09G0184720.1*
LOC105040534	*AZ10G0203990.1*
LOC105043192	*AZ10G0200550.1*
LOC105043438	*AZ10G0202500.1*
LOC105043705	*AZ10G0204040.1*
LOC105044046	*AZ10G0206030.1*
LOC105054112	*AZ10G0202500.1*
LOC105053972	*AZ10G0203990.1*
LOC105054331	*AZ10G0204040.1*
LOC105053837	*AZ10G0205290.1*
LOC105053827	*AZ10G0205400.2*
LOC105053760	*AZ10G0206030.1*
LOC105053692	*AZ10G0200550.1*
LOC105054880	*AZ10G0197820.1*
LOC105055559	*AZ10G0198830.1*
LOC105055088	*AZ10G0206030.1*
LOC105054760	*AZ10G0203990.1*
LOC105059234	*AZ10G0198830.1*
LOC105059635	*AZ10G0206030.1*
LOC105061133	*AZ10G0198830.1*
LOC109504900	*AZ10G0197820.1*

LOC105033230	*AZ10G0195710.1*
LOC105034876	*AZ10G0205290.1*
LOC105042862	*AZ11G0217800.1*
LOC105042961	*AZ11G0219380.1*
LOC105044046	*AZ11G0214040.1*
LOC105043316	*AZ11G0212760.2*
LOC105054495	*AZ11G0220750.1*
LOC105053760	*AZ11G0214040.1*
LOC105053729	*AZ11G0217040.1*
LOC105054972	*AZ11G0212760.2*
LOC105055088	*AZ11G0214040.1*
LOC105055259	*AZ11G0215700.1*
LOC105055271	*AZ11G0215810.1*
LOC105055454	*AZ11G0217040.1*
LOC105055559	*AZ11G0219380.1*
LOC105055678	*AZ11G0220750.1*
LOC105054880	*AZ11G0217970.1*
LOC105054905	*AZ11G0217800.1*
LOC105054743	*AZ11G0211900.1*
LOC105054720	*AZ11G0212140.1*
LOC105054576	*AZ11G0208640.1*
LOC105059478	*AZ11G0211900.1*
LOC105059493	*AZ11G0212140.1*
LOC105059523	*AZ11G0212760.2*
LOC105059635	*AZ11G0214040.1*
LOC105058870	*AZ11G0215700.1*
LOC105059014	*AZ11G0217800.1*
LOC105059220	*AZ11G0217970.1*
LOC105059234	*AZ11G0219380.1*
LOC109504900	*AZ11G0217970.1*
LOC105033428	*AZ11G0220750.1*
LOC105034135	*AZ11G0208510.1*
LOC105037623	*AZ12G0231220.1*
LOC105036764	*AZ12G0231930.1*
LOC105038489	*AZ12G0231220.1*
LOC105038411	*AZ12G0231930.1*
LOC105038821	*AZ12G0227370.1*
LOC105051753	*AZ12G0231220.1*
LOC105051841	*AZ12G0231930.1*
LOC105041823	*AZ13G0239190.1*
LOC105041083	*AZ13G0238110.1*
LOC105042862	*AZ13G0248380.1*
LOC105043254	*AZ13G0245410.1*
LOC105042961	*AZ13G0249410.1*

LOC105044046	*AZ13G0246320.1*
LOC105044321	*AZ13G0250330.1*
LOC105048503	*AZ13G0238110.1*
LOC105053200	*AZ13G0238110.1*
LOC105053113	*AZ13G0239080.1*
LOC105053101	*AZ13G0239190.1*
LOC105052936	*AZ13G0240920.1*
LOC105054495	*AZ13G0250330.1*
LOC105053837	*AZ13G0242460.1*
LOC105053972	*AZ13G0244680.1*
LOC105053760	*AZ13G0246320.1*
LOC105054924	*AZ13G0245720.1*
LOC105055088	*AZ13G0246320.1*
LOC105055559	*AZ13G0249410.1*
LOC105055678	*AZ13G0250330.1*
LOC105054880	*AZ13G0248440.1*
LOC105054905	*AZ13G0248380.1*
LOC105054760	*AZ13G0244680.1*
LOC105054743	*AZ13G0244820.1*
LOC105054949	*AZ13G0245410.1*
LOC105056442	*AZ13G0239190.1*
LOC105059464	*AZ13G0244680.1*
LOC105059475	*AZ13G0244820.1*
LOC105059570	*AZ13G0245720.1*
LOC105059635	*AZ13G0246320.1*
LOC105059014	*AZ13G0248380.1*
LOC105059220	*AZ13G0248440.1*
LOC105059234	*AZ13G0249410.1*
LOC114914865	*AZ13G0249550.1*
LOC105059546	*AZ13G0245410.1*
LOC109504900	*AZ13G0248440.1*
LOC105033428	*AZ13G0250330.1*
LOC105052798	*AZ14G0255610.1*
LOC105046145	*AZ14G0257350.1*
LOC105046102	*AZ14G0257640.1*
LOC105046084	*AZ14G0257750.1*
LOC105045725	*AZ14G0259720.1*
LOC105045662	*AZ14G0260160.1*
LOC105045660	*AZ14G0260170.1*
LOC105046315	*AZ14G0260820.1*
LOC105046273	*AZ14G0261090.1*
LOC105046375	*AZ14G0261200.1*
LOC105044477	*AZ14G0259720.1*
LOC105044915	*AZ14G0257750.1*

LOC105046865	*AZ14G0255610.1*
LOC105052225	*AZ14G0260160.1*
LOC105052226	*AZ14G0260170.1*
LOC105052782	*AZ14G0255610.1*
LOC105052376	*AZ14G0257640.1*
LOC105052363	*AZ14G0257750.1*
LOC105052148	*AZ14G0260820.1*
LOC105052145	*AZ14G0261200.1*
LOC105056992	*AZ14G0259720.1*
LOC105057270	*AZ14G0257750.1*
LOC105059783	*AZ14G0250600.1*
LOC105059778	*AZ14G0250630.1*
LOC105059740	*AZ14G0251180.1*
LOC105061367	*AZ14G0257640.1*
LOC105032078	*AZ14G0257350.1*
LOC105032584	*AZ14G0261090.1*
LOC105032833	*AZ14G0259720.1*
LOC105061221	*AZ15G0266690.1*
LOC105041823	*AZ15G0263060.1*
LOC105041749	*AZ15G0263930.1*
LOC105041730	*AZ15G0264170.1*
LOC105041775	*AZ15G0265110.1*
LOC105041906	*AZ15G0262750.1*
LOC105044915	*AZ15G0263930.1*
LOC105048809	*AZ15G0263930.1*
LOC105048799	*AZ15G0264170.1*
LOC105048630	*AZ15G0265110.1*
LOC105053103	*AZ15G0263060.1*
LOC105053101	*AZ15G0263070.1*
LOC105056223	*AZ15G0261610.1*
LOC105055954	*AZ15G0266690.1*
LOC105056637	*AZ15G0263830.1*
LOC105056616	*AZ15G0263930.1*
LOC105056584	*AZ15G0264170.1*
LOC105056816	*AZ15G0265110.1*
LOC105056443	*AZ15G0263060.1*
LOC105056442	*AZ15G0263070.1*
LOC105058320	*AZ15G0264170.1*
LOC105058421	*AZ15G0265110.1*
LOC105058783	*AZ15G0261610.1*
LOC105058663	*AZ15G0262750.1*
LOC105058564	*AZ15G0263830.1*
LOC105058553	*AZ15G0263930.1*
LOC105058185	*AZ15G0266690.1*

LOC105039918	*AZ16G0276570.1*
LOC105039994	*AZ16G0277170.1*
LOC105045854	*AZ16G0278830.1*
LOC105045730	*AZ16G0279940.1*
LOC105045069	*AZ16G0276570.1*
LOC105044973	*AZ16G0277170.1*
LOC105044566	*AZ16G0278830.1*
LOC105044622	*AZ16G0279130.3*
LOC105044479	*AZ16G0279940.1*
LOC105052376	*AZ16G0272220.1*
LOC105056993	*AZ16G0279940.1*
LOC105057408	*AZ16G0276570.1*
LOC105057307	*AZ16G0277170.1*
LOC105057083	*AZ16G0279130.3*
LOC105059778	*AZ16G0277170.1*
LOC105061367	*AZ16G0272220.1*

Table S9. Secondary structure of MYB protein in *Cocos nucifera*.

Protein	Alpha helix (aa) (Proportion (%))	Extended strand (aa) (Proportion (%))	Beta turn (aa) (Proportion (%))	Random coil (aa) (Proportion (%))
CnMYB2	64 (35.75)	14 (7.82)	8 (4.47)	93 (51.96)
CnMYB70	82 (20.10)	1 (0.25)	13 (3.19)	312 (76.47)
CnMYB158	118 (22.65)	69 (13.24)	10 (1.92)	324 (62.19)

Table S10. Secondary structure of MYB protein in *Cocos nucifera*

Protein	Alpha helix (aa) (Proportion (%))	Extended strand (aa) (Proportion (%))	Beta turn (aa) (Proportion (%))	Random coil (aa) (Proportion (%))
CnMYB1	221 (26.15)	69 (8.17)	20 (2.37)	535 (63.31)
CnMYB2	64 (35.75)	14 (7.82)	8 (4.47)	93 (51.96)
CnMYB3	62 (22.88)	1 (0.37)	10 (3.69)	198 (73.06)
CnMYB4	68 (20.73)	5 (1.52)	10 (3.05)	245 (74.70)
CnMYB5	81 (32.02)	5 (1.98)	13 (5.14)	154 (60.87)
CnMYB6	90 (21.90)	7 (1.70)	9 (2.19)	305 (74.21)
CnMYB7	109 (35.39)	5 (1.62)	12 (3.90)	182 (59.09)
CnMYB8	133 (29.17)	5 (1.10)	8 (1.75)	310 (67.98)
CnMYB9	103 (31.79)	5 (1.54)	11 (3.40)	205 (63.27)
CnMYB10	64 (20.92)	4 (1.31)	10 (3.27)	228 (74.51)
CnMYB11	59 (24.79)	3 (1.26)	11 (4.62)	165 (69.33)
CnMYB12	70 (25.93)	1 (0.37)	11 (4.07)	188 (69.63)
CnMYB13	131 (12.49)	28 (2.67)	11 (1.05)	879 (83.79)
CnMYB14	36 (40.45)	4 (4.49)	4 (4.49)	45 (50.56)
CnMYB15	29 (8.31)	27 (7.74)	4 (1.15)	289 (82.81)
CnMYB16	61 (17.89)	2 (0.59)	13 (3.81)	265 (77.71)
CnMYB17	72 (25.00)	6 (2.08)	11 (3.82)	199 (69.10)

CnMYB18	57 (22.27)	5 (1.95)	14 (5.47)	180 (70.31)
CnMYB19	96 (16.13)	21 (3.53)	4 (0.67)	474 (79.66)
CnMYB20	110 (19.78)	0 (0.00)	14 (2.52)	432 (77.70)
CnMYB21	120 (25.42)	36 (7.63)	18 (3.81)	298 (63.14)
CnMYB22	96 (27.67)	3 (0.86)	12 (3.46)	236 (68.01)
CnMYB23	61 (18.37)	7 (2.11)	10 (3.01)	254 (76.51)
CnMYB24	89 (31.90)	33 (11.83)	15 (5.38)	142 (50.90)
CnMYB25	63 (21.95)	9 (3.14)	12 (4.18)	203 (70.73)
CnMYB26	130 (18.06)	17 (2.36)	8 (1.11)	565 (78.47)
CnMYB27	80 (25.32)	14 (4.43)	4 (1.27)	218 (68.99)
CnMYB28	93 (31.85)	0 (0.00)	12 (4.11)	187 (64.04)
CnMYB29	87 (29.69)	0 (0.00)	11 (3.75)	195 (66.55)
CnMYB30	62 (19.02)	2 (0.61)	10 (3.07)	252 (77.30)
CnMYB31	33 (10.41)	39 (12.30)	7 (2.21)	238 (75.08)
CnMYB32	82 (23.16)	5 (1.41)	9 (2.54)	258 (72.88)
CnMYB33	59 (25.00)	2 (0.85)	9 (3.81)	166 (70.34)
CnMYB34	68 (26.05)	1 (0.38)	9 (3.45)	183 (70.11)
CnMYB35	55 (28.65)	1 (0.52)	11 (5.73)	125 (65.10)
CnMYB36	44 (51.76)	4 (4.71)	5 (5.88)	32 (37.65)
CnMYB37	54 (24.77)	5 (2.29)	9 (4.13)	150 (68.81)
CnMYB38	61 (19.37)	6 (1.90)	10 (3.17)	238 (75.56)
CnMYB39	23 (8.61)	29 (10.86)	4 (1.50)	211 (79.03)
CnMYB40	48 (40.34)	0 (0.00)	2 (1.68)	69 (57.98)
CnMYB41	41 (53.25)	0 (0.00)	4 (5.19)	32 (41.56)
CnMYB42	41 (53.25)	0 (0.00)	4 (5.19)	32 (41.56)
CnMYB43	68 (33.03)	0 (0.00)	9 (4.48)	124 (61.69)
CnMYB44	59 (30.41)	0 (0.00)	10 (5.15)	125 (64.43)
CnMYB45	72 (25.09)	17 (5.92)	8 (2.79)	190 (66.20)
CnMYB46	129 (39.69)	0 (0.00)	11 (3.38)	185 (56.92)
CnMYB47	72 (22.57)	1 (0.31)	10 (3.13)	236 (73.98)
CnMYB48	279 (31.89)	97 (11.09)	55 (6.29)	444 (50.74)
CnMYB49	59 (41.84)	15 (10.64)	13 (9.22)	54 (38.30)
CnMYB50	104 (20.51)	0 (0.00)	11 (2.17)	392 (77.32)
CnMYB51	85 (21.46)	0 (0.00)	10 (2.53)	301 (76.01)
CnMYB52	55 (17.92)	5 (1.63)	9 (2.93)	238 (77.52)
CnMYB53	85 (29.01)	1 (0.34)	13 (4.44)	194 (66.21)
CnMYB54	44 (22.68)	6 (3.09)	6 (3.09)	138 (71.13)
CnMYB55	53 (23.25)	0 (0.00)	10 (4.39)	165 (72.37)
CnMYB56	78 (28.68)	1 (0.37)	10 (3.68)	183 (67.28)
CnMYB57	81 (31.52)	1 (0.39)	11 (4.28)	164 (63.81)
CnMYB58	87 (21.48)	5 (1.23)	9 (2.22)	304 (75.06)
CnMYB59	86 (28.10)	7 (2.29)	10 (3.27)	203 (66.34)
CnMYB60	116 (24.47)	16 (3.38)	9 (1.90)	333 (70.25)
CnMYB61	84 (26.17)	1 (0.31)	10 (3.12)	226 (70.40)

CnMYB62	58 (19.59)	3 (1.01)	11 (3.72)	224 (75.68)
CnMYB63	168 (15.33)	17 (1.55)	14 (1.28)	897 (81.84)
CnMYB64	79 (51.30)	9 (5.84)	7 (4.55)	59 (38.31)
CnMYB65	23 (6.53)	28 (7.95)	4 (1.14)	297 (84.38)
CnMYB66	74 (21.33)	0 (0.00)	10 (2.88)	263 (75.79)
CnMYB67	64 (22.22)	6 (2.08)	10 (3.47)	208 (72.22)
CnMYB68	23 (7.74)	27 (9.09)	4 (1.35)	243 (81.82)
CnMYB69	45 (22.17)	8 (3.94)	5 (2.46)	145 (71.43)
CnMYB70	82 (20.10)	1 (0.25)	13 (3.19)	312 (76.47)
CnMYB71	52 (50.98)	0 (0.00)	4 (3.92)	46 (45.10)
CnMYB72	76 (27.74)	3 (1.09)	11 (4.01)	184 (67.15)
CnMYB73	168 (55.63)	2 (0.66)	10 (3.31)	122 (40.40)
CnMYB74	600 (52.59)	65 (5.70)	30 (2.63)	446 (39.09)
CnMYB75	66 (20.31)	3 (0.92)	10 (3.08)	246 (75.69)
CnMYB76	64 (18.18)	4 (1.14)	11 (3.12)	273 (77.56)
CnMYB77	90 (28.04)	6 (1.87)	11 (3.43)	214 (66.67)
CnMYB78	77 (20.75)	8 (2.16)	6 (1.62)	280 (75.47)
CnMYB79	77 (24.44)	4 (1.27)	11 (3.49)	223 (70.79)
CnMYB80	63 (24.90)	11 (4.35)	10 (3.95)	169 (66.80)
CnMYB81	62 (20.20)	11 (3.58)	10 (3.26)	224 (72.96)
CnMYB82	129 (38.62)	3 (0.90)	8 (2.40)	194 (58.08)
CnMYB83	71 (16.14)	1 (0.23)	12 (2.73)	356 (80.91)
CnMYB84	100 (21.83)	6 (1.31)	9 (1.97)	343 (74.89)
CnMYB85	187 (20.09)	200 (21.48)	42 (4.51)	502 (53.92)
CnMYB86	81 (20.61)	6 (1.53)	9 (2.29)	297 (75.57)
CnMYB87	118 (20.10)	0 (0.00)	13 (2.21)	456 (77.68)
CnMYB88	78 (31.08)	0 (0.00)	11 (4.38)	162 (64.54)
CnMYB89	93 (38.91)	6 (2.51)	8 (3.35)	132 (55.23)
CnMYB90	73 (19.16)	5 (1.31)	10 (2.62)	293 (76.90)
CnMYB91	70 (20.71)	5 (1.48)	13 (3.85)	250 (73.96)
CnMYB92	99 (17.84)	4 (0.72)	12 (2.16)	440 (79.28)
CnMYB93	398 (60.30)	13 (1.97)	8 (1.21)	241 (36.52)
CnMYB94	42 (32.31)	0 (0.00)	4 (3.08)	84 (64.62)
CnMYB95	39 (37.14)	0 (0.00)	4 (3.81)	62 (59.05)
CnMYB96	39 (30.00)	0 (0.00)	4 (3.08)	87 (66.92)
CnMYB97	39 (30.00)	0 (0.00)	4 (3.08)	87 (66.92)
CnMYB98	41 (31.54)	0 (0.00)	4 (3.08)	85 (65.38)
CnMYB99	39 (31.97)	0 (0.00)	4 (3.28)	79 (64.75)
CnMYB100	88 (22.22)	6 (1.52)	10 (2.53)	292 (73.74)
CnMYB101	75 (25.86)	2 (0.69)	10 (3.45)	203 (70.00)
CnMYB102	63 (17.12)	10 (2.72)	10 (2.72)	285 (77.45)
CnMYB103	151 (25.25)	23 (3.85)	22 (3.68)	402 (67.22)
CnMYB104	77 (28.95)	17 (6.39)	8 (3.01)	164 (61.65)
CnMYB105	328 (19.62)	48 (2.87)	16 (0.96)	1280 (76.56)

CnMYB106	83 (24.85)	8 (2.40)	3 (0.90)	240 (71.86)
CnMYB107	59 (24.28)	4 (1.65)	13 (5.35)	167 (68.72)
CnMYB108	77 (23.19)	4 (1.20)	11 (3.31)	240 (72.29)
CnMYB109	84 (23.27)	6 (1.66)	6 (1.66)	265 (73.41)
CnMYB110	66 (19.94)	10 (3.02)	13 (3.93)	242 (73.11)
CnMYB111	63 (32.98)	17 (8.90)	6 (3.14)	105 (54.97)
CnMYB112	45 (47.87)	6 (6.38)	6 (6.38)	37 (39.36)
CnMYB113	42 (44.68)	4 (4.26)	6 (6.38)	42 (44.68)
CnMYB114	46 (56.10)	3 (3.66)	3 (3.66)	30 (36.59)
CnMYB115	60 (23.44)	4 (1.56)	9 (3.52)	183 (71.48)
CnMYB116	22 (7.80)	34 (12.06)	5 (1.77)	221 (78.37)
CnMYB117	39 (46.99)	2 (2.41)	4 (4.82)	38 (45.78)
CnMYB118	77 (38.89)	0 (0.00)	13 (6.57)	108 (54.55)
CnMYB119	65 (20.38)	1 (0.31)	10 (3.13)	243 (76.18)
CnMYB120	25 (36.76)	6 (8.82)	4 (5.88)	33 (48.53)
CnMYB121	102 (22.08)	0 (0.00)	12 (2.60)	348 (75.32)
CnMYB122	66 (20.12)	3 (0.91)	14 (4.27)	245 (74.70)
CnMYB123	352 (32.87)	22 (2.05)	26 (2.43)	671 (62.65)
CnMYB124	87 (30.74)	5 (1.77)	7 (2.47)	184 (65.02)
CnMYB125	74 (35.07)	11 (5.21)	4 (1.90)	122 (57.82)
CnMYB126	66 (20.25)	6 (1.84)	10 (3.07)	244 (74.85)
CnMYB127	71 (22.47)	0 (0.00)	11 (3.48)	234 (74.05)
CnMYB128	113 (34.66)	1 (0.31)	10 (3.07)	202 (61.96)
CnMYB129	97 (30.12)	4 (1.24)	9 (2.80)	212 (65.84)
CnMYB130	83 (20.96)	5 (1.26)	9 (2.27)	299 (75.51)
CnMYB131	110 (27.71)	3 (0.76)	12 (3.02)	272 (68.51)
CnMYB132	122 (15.74)	30 (3.87)	11 (1.42)	612 (78.97)
CnMYB133	75 (25.51)	1 (0.34)	11 (3.74)	207 (70.41)
CnMYB134	64 (27.12)	10 (4.24)	10 (4.24)	152 (64.41)
CnMYB135	48 (50.53)	0 (0.00)	6 (6.32)	41 (43.16)
CnMYB136	95 (23.28)	4 (0.98)	14 (3.43)	295 (72.30)
CnMYB137	81 (18.20)	3 (0.67)	9 (2.02)	352 (79.10)
CnMYB138	58 (22.48)	1 (0.39)	10 (3.88)	189 (73.26)
CnMYB139	55 (18.77)	1 (0.34)	10 (3.41)	227 (77.47)
CnMYB140	65 (22.81)	7 (2.46)	11 (3.86)	202 (70.88)
CnMYB141	75 (32.19)	0 (0.00)	12 (5.15)	146 (62.66)
CnMYB142	68 (23.69)	0 (0.00)	11 (3.83)	208 (72.47)
CnMYB143	29 (9.29)	26 (8.33)	4 (1.28)	253 (81.09)
CnMYB144	67 (19.82)	4 (1.18)	14 (4.14)	253 (74.85)
CnMYB145	106 (29.44)	4 (1.11)	10 (2.78)	240 (66.67)
CnMYB146	74 (20.22)	2 (0.55)	9 (2.46)	281 (76.78)
CnMYB147	65 (29.68)	6 (2.74)	9 (4.11)	139 (63.47)
CnMYB148	121 (33.4)	6 (1.66)	11 (3.04)	224 (61.88)
CnMYB149	58 (23.67)	1 (0.41)	10 (4.08)	176 (71.84)

CnMYB150	42 (14.05)	34 (11.37)	4 (1.34)	219 (73.24)
CnMYB151	59 (19.41)	10 (3.29)	11 (3.62)	224 (73.68)
CnMYB152	173 (29.37)	32 (5.43)	19 (3.23)	365 (61.97)
CnMYB153	88 (25.00)	10 (2.84)	3 (0.85)	251 (71.31)
CnMYB154	128 (16.62)	23 (2.99)	10 (1.30)	609 (79.09)
CnMYB155	60 (25.32)	4 (1.69)	12 (5.06)	161 (67.93)
CnMYB156	74 (14.48)	22 (4.31)	4 (0.78)	411 (80.43)
CnMYB157	96 (20.96)	28 (6.11)	12 (2.62)	322 (70.31)
CnMYB158	118 (22.65)	69 (13.24)	10 (1.92)	324 (62.19)
CnMYB159	102 (18.85)	2 (0.37)	12 (2.22)	425 (78.56)
CnMYB160	57 (19.59)	6 (2.06)	11 (3.78)	217 (74.57)
CnMYB161	68 (22.67)	11 (3.67)	11 (3.67)	210 (70.00)
CnMYB162	67 (24.91)	1 (0.37)	9 (3.35)	192 (71.38)
CnMYB163	70 (39.11)	3 (1.68)	6 (3.35)	100 (55.87)
CnMYB164	60 (22.30)	1 (0.37)	10 (3.72)	198 (73.61)
CnMYB165	98 (19.14)	23 (4.49)	17 (3.32)	374 (73.05)
CnMYB166	62 (24.22)	0 (0.00)	9 (3.52)	185 (72.27)
CnMYB167	65 (22.34)	3 (1.03)	10 (3.44)	213 (73.20)
CnMYB168	316 (28.01)	15 (1.33)	19 (1.68)	778 (68.97)
CnMYB169	71 (25.63)	2 (0.72)	10 (3.61)	194 (70.04)
CnMYB170	74 (27.11)	0 (0.00)	11 (4.03)	188 (68.86)
CnMYB171	92 (37.70)	14 (5.74)	7 (2.87)	131 (53.69)
CnMYB172	56 (18.67)	3 (1.00)	11 (3.67)	230 (76.67)
CnMYB173	97 (28.70)	3 (0.89)	12 (3.55)	226 (66.86)
CnMYB174	27 (8.54)	29 (9.18)	4 (1.27)	256 (81.01)
CnMYB175	278 (42.97)	26 (4.02)	20 (3.09)	323 (49.92)
CnMYB176	139 (18.48)	12 (1.60)	8 (1.06)	593 (78.86)
CnMYB177	334 (56.23)	41 (6.90)	20 (3.37)	199 (33.50)
CnMYB178	59 (25.65)	7 (3.04)	11 (4.78)	153 (66.52)
CnMYB179	96 (15.92)	0 (0.00)	12 (1.99)	495 (82.09)
CnMYB180	73 (22.74)	7 (2.18)	11 (3.43)	230 (71.65)
CnMYB181	61 (20.33)	5 (1.67)	10 (3.33)	224 (74.67)
CnMYB182	53 (37.06)	17 (11.89)	6 (4.20)	67 (46.85)
CnMYB183	63 (23.16)	1 (0.37)	9 (3.31)	199 (73.16)
CnMYB184	79 (18.63)	2 (0.47)	12 (2.83)	331 (78.07)
CnMYB185	60 (22.99)	0 (0.00)	12 (4.60)	189 (72.41)
CnMYB186	80 (19.56)	1 (0.24)	13 (3.18)	315 (77.02)
CnMYB187	75 (30.24)	3 (1.21)	10 (4.03)	160 (64.52)
CnMYB188	101 (18.67)	1 (0.18)	12 (2.22)	427 (78.93)
CnMYB189	89 (27.38)	3 (0.92)	13 (4.00)	220 (67.69)
CnMYB190	68 (18.13)	10 (2.67)	10 (2.67)	287 (76.53)
CnMYB191	63 (25.01)	2 (0.80)	9 (3.59)	177 (70.52)
CnMYB192	132 (22.45)	6 (1.02)	14 (2.38)	436 (74.15)
CnMYB193	315 (39.33)	76 (9.49)	39 (4.87)	371 (46.32)

CnMYB194	83 (21.45)	8 (2.07)	6 (1.55)	290 (74.94)
CnMYB195	70 (23.03)	11 (3.62)	6 (1.97)	217 (71.38)
CnMYB196	105 (23.08)	6 (1.32)	11 (2.42)	333 (73.19)
CnMYB197	102 (23.39)	3 (0.69)	9 (2.06)	322 (73.85)
CnMYB198	69 (21.84)	1 (0.32)	11 (3.48)	235 (74.37)
CnMYB199	86 (24.36)	12 (3.40)	10 (2.83)	245 (69.41)
CnMYB200	54 (18.24)	1 (0.34)	10 (3.38)	231 (78.04)
CnMYB201	77 (33.77)	0 (0.00)	10 (4.39)	141 (61.84)
CnMYB202	113 (29.58)	3 (0.79)	11 (2.88)	255 (66.75)
CnMYB203	83 (21.07)	10 (2.54)	10 (2.54)	291 (73.86)
CnMYB204	95 (30.16)	3 (0.95)	10 (3.17)	207 (65.71)
CnMYB205	102 (31.29)	1 (0.31)	10 (3.07)	213 (65.34)
CnMYB206	262 (26.84)	171 (17.52)	75 (7.68)	468 (47.95)
CnMYB207	90 (28.39)	4 (1.26)	13 (4.10)	210 (66.25)
CnMYB208	97 (32.88)	3 (1.02)	9 (3.05)	186 (63.05)
CnMYB209	124 (27.56)	12 (2.67)	12 (2.67)	302 (67.11)
CnMYB210	69 (23.55)	4 (1.37)	11 (3.75)	209 (71.33)
CnMYB211	83 (22.87)	11 (3.03)	10 (2.75)	259 (71.35)
CnMYB212	82 (29.50)	16 (5.76)	8 (2.88)	172 (61.87)
CnMYB213	320 (19.06)	37 (2.20)	15 (0.89)	1307 (77.84)
CnMYB214	220 (36.48)	51 (8.46)	32 (5.31)	300 (49.75)
